# Delayed diagnostic evaluation of symptomatic breast cancer in sub-Saharan Africa: A qualitative study of Tanzanian women

**DOI:** 10.1371/journal.pone.0275639

**Published:** 2022-10-06

**Authors:** Lulu Lunogelo Sakafu, Godfrey Sama Philipo, Christina V. Malichewe, Lulu S. Fundikira, Flora A. Lwakatare, Katherine Van Loon, Beatrice P. Mushi, Rebecca J. DeBoer, Stella A. Bialous, Amie Y. Lee

**Affiliations:** 1 Department of Radiology, Muhimbili National Hospital, Dar es Salaam, Tanzania; 2 Muhimbili University of Health and Allied Sciences, Dar es Salaam, Tanzania; 3 Division of Hematology/Oncology, Department of Medicine, University of California San Francisco (UCSF), San Francisco, California, United States of America; 4 Department of Social and Behavioral Sciences, University of California San Francisco (UCSF), San Francisco, California, United States of America; 5 Department of Radiology and Biomedical Imaging, University of California San Francisco (UCSF), San Francisco, California, United States of America; Gulu University Faculty of Medicine, UGANDA

## Abstract

**Background:**

Women with breast cancer in sub-Saharan Africa are commonly diagnosed at advanced stages. In Tanzania, more than 80% of women are diagnosed with stage III or IV disease, and mortality rates are high. This study explored factors contributing to delayed diagnostic evaluation among women with breast cancer in Tanzania.

**Methods:**

A qualitative study was performed at Muhimbili National Hospital in Dar es Salaam, Tanzania. Twelve women with symptomatic pathologically proven breast cancer were recruited. In-depth, semi-structured interviews were conducted in Swahili. Interviews explored the women’s journey from symptom recognition to diagnosis, including the influence of breast cancer knowledge and pre-conceptions, health seeking behaviors, psychosocial factors, preference for alternative treatments, and the contribution of culture and norms. Audio-recorded interviews were transcribed and translated into English. Thematic analysis was facilitated by a cloud-based qualitative analysis software.

**Results:**

All women reported that their first breast symptom was a self-identified lump or swelling. Major themes for factors contributing to delayed diagnostic presentation of breast cancer included lack of basic knowledge and awareness of breast cancer and misconceptions about the disease. Participants faced barriers with their local primary healthcare providers, including symptom mismanagement and delayed referrals for diagnostic evaluation. Other barriers included financial hardships, fear and stigma of cancer, and use of traditional medicine. The advice and influence of family members and friends played key roles in healthcare-seeking behaviors, serving as both facilitators and barriers.

**Conclusion:**

Lack of basic knowledge and awareness of breast cancer, stigma, financial barriers, and local healthcare system barriers were common factors contributing to delayed diagnostic presentation of breast cancer. The influence of friends and family also played key roles as both facilitators and barriers. This information will inform the development of educational intervention strategies to address these barriers and improve earlier diagnosis of symptomatic breast cancer in Tanzania.

## Introduction

Breast cancer is a disease that affects women and men worldwide, contributing to a substantial public health burden. It is estimated that 2.3 million new cases of breast cancer are diagnosed each year [1]. Breast cancer has surpassed lung cancer as the most commonly diagnosed malignancy, accounting for 12% of all cancers diagnosed in 2020. It is the leading cause of cancer death in women worldwide [1]. Although breast cancer incidence is higher in high-income countries, it is increasing rapidly in low- and middle-income countries (LMICs) [2]. Disadvantaged communities face the heaviest disease burden in terms of mortality and morbidity associated with a breast cancer diagnosis [3, 4].

Sub-Saharan Africa is experiencing a growing cancer burden [5, 6]. Cancer of the breast is the second most common cause of cancer among women in Tanzania and is the second highest cause of cancer mortality among women in the country [7, 8]. It is predicted that from 2012 to 2030 the number of new breast cancer cases in Tanzania will increase by 82% and the number of breast cancer deaths will increase by 80% [9].

The mortality-to-incidence ratio (MIR) for breast cancer in Tanzania is 0.5, indicating that half of all women diagnosed with breast cancer will succumb to the disease [7]. While causes of high MIR are complex and multifactorial, partly contributing may be the fact that a majority of Tanzanian women with breast cancer are diagnosed when the disease has reached an advanced stage, when treatments are less effective and survival is poor. Studies at Muhimbili National Hospital (MNH) and Tumani Hospital in Dar es Salaam, Tanzania found that more than 80% of patients with breast cancer presented with at least stage III disease [9, 10]. A study performed in 2013 found that most patients were stage III (59%) or stage IV (32%) upon arrival to Ocean Road Cancer Institute (ORCI), the largest cancer center in Tanzania [11]. Addressing the causes of delayed breast cancer diagnosis in Tanzania will be a critical component to improving outcomes.

There is currently a lack of studies investigating the factors contributing to delayed diagnosis of breast cancer among Tanzanian women. A few survey-based studies have evaluated breast cancer knowledge and beliefs among Tanzanian women, the vast majority (>97%) of whom had no personal history of breast cancer [12, 13]. To our knowledge, there are few studies to date that have specifically explored the journey and perspective of Tanzanian women with breast cancer. We sought to explore barriers and facilitators to timely diagnostic presentation of patients with symptomatic breast cancer in Tanzania.

## Methods

### Study design

This qualitative investigation used an interpretive phenomenology design [14–16] to explore the lived experience of Tanzanian women with breast cancer, investigating the participants’ journey from symptom recognition to breast cancer diagnosis. Data were collected through in-depth semi-structured interviews. This qualitative approach was utilized since little is presently known about factors contributing to delayed diagnostic presentation of breast cancer in Tanzania. The study was approved by the institutional review boards of Muhimbili University of Health and Allied Sciences (MUHAS; Research and Publications Committee) and the University of California, San Francisco (UCSF). Permission to conduct the study was obtained from Muhimbili National Hospital (MNH).

In this study, we took rigorous steps to ensure the trustworthiness and validity of our work. A detailed description of the inclusion and exclusion criteria, working environment, data collection process, and data analysis is provided below. All interviews were conducted by the same researcher who had dedicated in-depth coursework training in qualitative research, ensuring fidelity to the methodology.

### Study setting

This study was conducted at MNH in Dar es Salaam, Tanzania. MNH is the teaching hospital affiliated with MUHAS. It is a national referral hospital with a 1,500 bed capacity and provides care for up to 2,000 outpatients per day. MNH is the largest referral hospital for cancer diagnosis in the country, equipped with a state-of-the-art radiology department with diagnostic breast imaging capabilities, and well-established medical, surgical, and pathology departments.

### Participants and recruitment procedures

Participants were recruited from June to September 2019. Patients with a histologically confirmed diagnosis of breast cancer who were actively undergoing surgical/oncologic management were recruited from the Radiology or Oncology Departments at MNH.

All eligible participants were approached by the research assistant. Purposive sampling techniques were used to invite participants whereby all eligible participants visiting the clinic during the study period were consecutively recruited. Individuals who were too ill to participate in the interview were excluded. Patients who met the inclusion criteria and completed the written informed consent were enrolled. Participants were briefed about the study and were informed that their participation was voluntary with the right to withdraw at any time. Participants consented to participate in the study and for their interview to be recorded. Recruitment was done concurrently with data analysis and stopped once saturation was reached on key themes, with saturation defined as no longer finding new information adding to our understanding of the categories or coming to a point in the data collection where no new categories emerge [14, 17, 18].

### Data collection

Interviews were conducted in a quiet private room at the hospital premises. Interviews were conducted in Swahili, the national language of Tanzania, and were all performed by a single research assistant with formal training in qualitative research methodology. A semi-structured interview guide was developed by the research team and piloted on three women before formal recruitment started. Interview questions focused on exploring the participant’s journey from the first recognition of breast signs and symptoms to diagnosis of breast cancer, including barriers and facilitators to timely diagnosis. Interviews delved into factors such as pre-existing breast cancer knowledge and pre-conceptions, psychosocial factors, health-seeking behavior, preference for alternative treatment, and the contribution of culture and norms.

Demographic data of participants were also collected, including age, highest level of education, marital status, number of children, place of permanent residence, and distance and method of transportation to the local healthcare provider. Key medical information collected from the patients included the breast signs and symptoms, who detected the signs and symptoms, time between symptom onset and seeking of medical care, use of traditional medicine, breast imaging evaluations performed prior to being referred to MNH, and breast cancer risk factors, such as family history of breast cancer.

Interviews were audio-recorded to ensure that all information as reported by participants was captured. The recorded interviews were then transcribed verbatim in Swahili and translated into English after each interview by a single research assistant (an intern physician). These transcribed interviews, both the Swahili and English versions, were subsequently reviewed for accuracy by the interviewer. Both individuals, the interviewer and the transcriber, had advanced proficiency in both Swahili (native language) and English. Interviews were performed until saturation was achieved on key themes (N = 12). This theoretical saturation point was defined as no longer finding new information adding to our understanding of the categories and no new categories emerging. In other words, we stopped data collection at the point where the women’s experiences became repetitive/redundant and further data collection became unnecessary [14, 17, 18].

### Data analysis

Patient characteristics, including socio-demographic data and breast symptoms were summarized with descriptive statistics. Qualitative analysis was performed using Dedoose, a cloud-based qualitative analysis coding application software. Transcripts in English were reviewed concurrently with the data collection to explore initial emerging issues to follow up in subsequent interviews, until saturation was achieved. Data were coded and analyzed using a thematic approach [17, 19, 20]. The interview transcripts were imported into the coding application software, where they were read and re-read by the investigators. A codebook was established based on issues emerging inductively from the data. Three investigators (AL, LS, and GS) independently coded the transcripts, blinded to one another. Through an iterative process, the coding schedule was revised; divergent applications were discussed, definitions were revised, and new codes were added through discussion and consensus. The final revised coding schedule was applied to all 12 transcripts by the investigators. The frequencies and agreement of the final coded excerpts were analyzed, reviewed, and again discussed as a group. Themes were inducted from the codes, and these were categorized into barriers and facilitators to breast cancer diagnosis. Quotes that best described the themes and best represented what was said by participants were chosen and presented in italics.

## Results

### Description of participants

Semi-structured interviews were conducted in a total of 12 women with histologically proven breast cancer. Each interview lasted for approximately 30 minutes. Mean age was 50 years (range 40–65). The most commonly reported highest level of education was primary school (42%) and secondary school (42%). The most common occupations were petty trader (25%) and peasant (25%). Most participants were married (58%) or widowed (17%). One participant had no children; the remaining 11 participants had children.

All participants reported that their first breast symptom was a self-identified lump or swelling. Additionally, three women had associated breast pain and one woman had associated bloody discharge. Study participants’ characteristics are summarized in Table 1.

**Table 1:** 

Study participants’ characteristics (N = 12)	
**Age**	Mean 50 years (range 40–65)
**Highest education level**	
Primary school	5 (42%)
Secondary school	5 (42%)
University	1 (8%)
No formal education	1 (8%)
**Marital status**	
Married	7 (58%)
Single	3 (25%)
Widowed	2 (17%)
**Number of children**	Mean 3 (range 0–5)
**Occupation**	
Petty trader	3 (25%)
Peasant/farmer	3 (25%)
Primary school teacher	1 (8%)
Hair salon	1 (8%)
Electric company employee	1 (8%)
Secretary/Office assistant	1 (8%)
Civil servant	1 (8%)
“Various businesses”	1 (8%)
**Presenting breast signs or symptoms**	
Lump/Swelling	12 (100%)
Lump/Swelling + pain	3 (25%)
Lump/Swelling + bloody discharge	1 (8%)
**Who first identified the symptoms?**	
Self-identified	12 (100%)
**Time from symptom onset to diagnostic evaluation**	Mean 10 months (range 4–24)
**Method of transport of local healthcare provider**	
Foot or paid taxi/bajaji/motorbike	4 (33%)
Foot only	3 (25%)
Bus only	2 (17%)
Foot or bus	1 (8%)
Pedicab	1 (8%)
Unknown/no response	1 (8%)

### Factors contributing to delayed diagnosis of breast cancer

The main emergent themes from the participants’ accounts of their journeys from symptom recognition to breast cancer diagnosis were grouped as barriers and facilitators to timely diagnosis. These are summarized in Table 2.

**Table 2:** 

Themes	Subthemes
Barriers	• Lack of cancer knowledge • Financial hardships • Local health system or health provider • Use of traditional or alternative treatments • Cancer fear or stigma • Relatives and friends
Facilitators	• Relatives and friends • Prior education on breast cancer

#### Barriers

Participants’ responses revealed six common barriers that contributed to delayed diagnosis.

#### Lack of breast cancer knowledge

All participants admitted to recognizing a symptom, such as a lump or swelling in the breast, but most didn’t understand that it could be a sign of cancer. Many were initially not worried about the symptoms and decided to ignore it, believing it was not a serious condition.

“*I thought that it was just a normal condition for a woman to feel a little problem*. *I had never thought the disease of cancer in my mind*. *I didn’t think that I can get such a thing…Probably it is a communicable disease*, *so getting such a disease would be difficult*.*” (P01*, *age 62)*

“*I wasn’t knowledgeable when I saw the swelling and didn’t understand it*.*” (P10*, *age 57)*

“*I never noticed it was cancer because I only saw something hard…So you are sick but you keep on working regarding it as a normal condition*.*” (P12*, *age 65)*

Others were reassured by the absence of pain, unaware that breast cancers commonly present as painless palpable lumps.

“*We didn’t take it seriously because it wasn’t painful*. *I continued doing my activities*, *you know how we neglect things … without knowing that the more we delay the more the problem worsens*.*” (P11*, *age 42)*

“*I didn’t understand what it was*, *I didn’t understand it without the kids telling me to go to the hospital! I was just asking myself why was the area hard however there was no pain*. *Only later did I decide to get to the hospital*.*” (P03*, *age 42)*

“*The problem is the knowledge*, *not being aware*, *not understanding something…You start questioning what it is and if it has no pain you might just ignore it*.*” (P07*, *age 56)*

Participants reported seeking health care four months to two years later, after symptoms had progressed to more advanced stages. One participant was aware of self-breast examination, noticed a lump, and shared the information with her husband. Together they agreed to wait several months before seeking medical care.

#### Financial hardships

Participants described their struggle to get money so they could access health care, contributing to delays in diagnosis. After being seen by a primary health care provider, several participants had to mobilize to collect funds, through efforts such as family fundraising, to get enough money to receive the necessary diagnostic services. Some had to travel to zonal and referral hospitals for necessary diagnostic investigations and needed to find funds for travel and temporary accommodations.

“*The doctor told me to have an ultrasound or x-ray so that they can give me a transfer to come [to MNH] to know what it is*. *Unfortunately*, *I didn’t have enough money so I had to return home… I knew the problem of cancer is a disease that is so scary in Tanzania or the world*. *It is the disease that is most feared*, *but to be honest*, *money was the greatest challenge”*. *(P11*, *age 42)*

“*Since I was poor*, *I couldn’t manage the costs*. *I struggled to get money for the investigations*.*” (P09)*

“*It was expensive because I was paying*. *So*, *there were high costs indeed*. *Moreover*, *taking into account the transport costs and food costs when you are away*, *in fact the expenses are high…You consider the fare from Tanga to Moshi*, *how to live there and you don’t have relatives there*, *now you can see*. *I was so late such that I was in bad condition*. *So the expenses to enable me to get the services there*, *to stay at a lodge*, *was a challenge*.*” (P01*, *age 62)*

“*It took about one month and three weeks when my sister had all the money needed*. *Then I came to Muhimbili” (P04*, *age 41)*

“*What are other reasons for the delay? Financial crisis*, *money has been a problem*. *Especially this time*, *it didn’t rain*, *so few was harvested*. *There was an option of selling cows*, *but they are not good-looking to sell due to lack of pasture and are sold at a cheaper price*. *And this place is too far*, *especially for two people it is really expensive*.*” (P08*, *age 54)*

These struggles to gather funds for investigations contributed to delays in breast cancer diagnosis. Some expressed a sense of inevitability that those who are poor will suffer.

“*The factors that lead to the delay*, *for instance for us peasants*, *it’s the monetary factor…They are just there*, *hopelessly waiting for God’s call*, *waiting for their death*.*” (P04*, *age 51)*

#### Local health system or health provider barriers

When participants did seek medical attention for their breast symptom, several reported further delays in diagnosis due to barriers within the local health system. For example, some participants managed to get to their primary health care provider in a timely manner, but they were assured that the symptom was nothing worrisome.

“*I noticed a small swelling*. *A small one here on the breast*. *So I went to the hospital*, *and the doctor that was there told me that it wasn’t a problem*… *Yes*, *so you become less worried and you proceed with your works and mind you it is a medical doctor who told me that it wasn’t a problem and I didn’t go to a traditional healer*, *I only went to the hospital for that*.*” (P10*, *age 57)*

“*I felt like there is a swelling inside…When I arrived [at the hospital]*, *the doctor examined me and told me that the breast doesn’t have threatening symptoms*.*” (P02*, *age 44)*

Two participants visited their local healthcare provider and were given acetaminophen tablets for their breast lumps. Others were given antibiotics, further delaying diagnosis.

“*I went to the district hospital and told them*. *They gave me medications*, *antibiotics*, *I took them but the situation didn’t change*. *It was still painful*. *I went again after I completed the dose*, *and they changed the dose they gave me another one*.*” (P09*, *age 40)*

“*At the regional hospital they told me that it was an abscess*, *and because it didn’t show where it originated from then they decided to give me antibiotics*. *They gave me the medications*, *and after sometime I didn’t notice it turning out to be an abscess*, *so I went to Dodoma*.*” (P06*, *age 41)*

Improper management and delay in diagnostic work-up may reflect to lack of knowledge among primary health care providers in the recognition and appropriate evaluation of breast symptoms. Further, most primary health care facilities lack biopsy capabilities, imaging equipment, and pathology services necessary to establish a breast cancer diagnosis. This necessitates referrals to zonal or referral hospitals for a diagnosis to be made. In many cases, participants reported difficulties in obtaining referrals, which further contributed to delays.

“*There is a challenge how to get a referral*. *I don’t know whether it is that the doctor tried to treat and see what he will achieve but as a patient*, *you are suffering*. *You as a patient*, *you are deteriorating*. *I prefer if there were enough investigations*, *many hospitals and specialists*, *it would be helpful*.*” (P01*, *age 62)*

“*There are areas where the health care providers did wrong…those doctors from the regional hospital*, *if they know that it is not their field*, *then they should not tamper with things that could cost somebody’s life*, *and they should make the referral system more official*.*” (P06*, *age 41)*

#### Use of traditional or alternative treatments

Some participants faced delays in cancer diagnosis due to first seeking care from a traditional healer. One participant believed she was being bewitched so she went to a traditional healer first. Most who sought traditional medicine for their breast symptoms reported spending more than three months using these alternative treatments. Upon realizing that their symptoms were worsening, they later sought care at a medical facility.

“*I was given local herbs that once I urinate the swelling would go*, *but there was no response so I went to the hospital and I was told that it is cancer*.*” (P08*, *age 54)*

“*So*, *there was a medication that I was applying on that area*, *[and] as I apply it there it continues to dig deeper and deeper*. *What he was telling me was the way it digs is how it eats away cancer*, *so let the meat be eaten away okay*. *So… sometimes I bleed about half a bucket*, *I get deficit of blood*, *and it continues that way…Later on I saw that it was abnormal and not good*.*” (P09*, *age 40)*

In addition, while some participants benefited from prayers as a source of comfort, others believed they could only be healed by prayers. Thus, they spent significant time with prayers alone until they recognized that the disease was still progressing.

“*I used to go to prayers*. *I went*, *I prayed*, *they prayed for me*. *The condition was not improving*. *That is why I decided to come to the hospital*.*” (P03*, *age 42)*

#### Cancer fears and stigma

Societal stigmatization of cancer led some participants to fear revealing their symptoms to others, contributing to delay in seeking medical care. Others reported fear of physical deformity from surgery, that cancer was an incurable disease, or that going to the hospital was a death sentence.

“*I told the professor*, *‘If my breast was cut*, *I would die*. *If that is the case*, *then just leave me to die*.*” (P01*, *age 62)*

“*In the environment that I live*, *I didn’t want anyone to know that I have the problem*. *In the end of the day*, *I think it is what made me delay to come to the hospital…There is no helping rather than laughing at one another*. *They mostly come and say I have seen so and so*, *she has this terrible disease that cannot be cured*. *So*, *people tell you it is better to have AIDS*, *you will be cured rather than this disease*. *There is when you get the fear*.*” (P11*, *age 42)*

#### Family or friends as barrier

Relatives and friends had a substantial influence on how participants acted on their breast symptoms. They sometimes advised the participant to wait rather than seek prompt medical attention.

“*I just used to show my close friends*, *the swelling and all that*, *they are the ones who advised me that we should observe it first*.*” (P05*, *age 41)*

“*I told my husband*, *‘I can feel as if there is a swelling starting here’*. *I told him*, *yes*. *Then he decided to wait a bit*, *we stayed for around four months*.*” (P03*, *age 42)*

Relatives also relayed fears of the hospital to the participant, equating cancer diagnosis and treatment with death.

“*I went home and told my relatives that if I delay it will develop into cancer*. *My sister*, *who has studied politics*, *said no*, *‘you shouldn’t be operated to cut the breast because if it’s cut it will spread in the whole-body system’*.*” (P04*, *age 51)*

“*The role of my few relatives is that they were giving me advice*, *but a few of them told me if I had my breast removed and I would die and they won’t be involved in it*.*” (P01*, *age 62)*

Further, some relatives were the main advisors in encouraging participants to first seek traditional healers. Two participants reported consulting with their husbands who did not take their concerns about the symptoms seriously. In addition, participants reported struggling to balance their own welfare with the needs of their family, which led to delays in seeking health care. Participants had to attend to societal and familial obligations, such as organizing a relative’s burial ceremony or taking care of sick family members, prior to attending to their own medical care.

### Facilitators

In contrast to these many barriers, participants reported far fewer facilitators to timely breast cancer diagnosis. These included family and friends’ encouragement and prior breast cancer knowledge.

#### Family or friends as facilitators

While sometimes a barrier, in other cases relatives and friends were critical in facilitating and expediting the diagnostic process. Some relatives were the source of financial support for medical care. In other cases, friends and relatives advised that participants seek prompt medical attention and encouraged them throughout the whole process.

“*The ones who pushed me are my brothers*. *To be honest*, *after I showed them the swelling and for everyday that the swelling was growing and hardening*, *my brothers advised me to go to the hospital*, *and they are the ones who challenged me*, *and I thank them for where we have reached*.*” (P11*, *age 42)*

“*There was a lump that I was wondering about*. *I then called my daughter who is a nurse working in Korogwe; I asked her about the lump in my breast*. *I also told my other daughter who is also a nurse but working in Iringa*, *she advised to go to the hospital…The relatives are the ones helping me*, *my kids*. *They were the ones who were putting pressure on me*.*” (P07*, *age 56)*

#### Prior education on breast cancer

Two participants were knowledgeable about the signs and symptoms of breast cancer, prompting them to seek diagnostic evaluation. They had received information from television and radio programs. One participant received prior education as part of her maternal health services.

“*I heard about the disease through the radio*, *RTD station*, *and I came to hear about it in the year 2009 after being pregnant when I was attending the reproductive health center*. *They informed us about the disease*, *so*, *I was aware with that*. *They taught us as part of maternal health…So after I noticed the lump*, *I went to the hospital"*. *(P06*, *age 41)*

“*I have heard it on the radio*. *Whenever I watch the TV I see them announcing that there is breast cancer*. *They explain explicitly that one can know that they have breast cancer by going to see a doctor so that they get investigated*.*" (P01*, *age 62)*

Nevertheless, knowledge of breast cancer alone was not sufficient to overcome the other barriers and even these patients experienced delays in diagnosis.

## Discussion

This study explored the factors contributing to delayed diagnostic evaluation of symptomatic breast cancer among Tanzanian women. As in most sub-Saharan African countries, population-based mammographic screening programs have not yet been established in Tanzania. Therefore, diagnoses are almost exclusively dependent on the recognition of breast cancer signs and symptoms. It is well established that delay between onset of breast symptoms and the diagnosis of breast cancer is detrimental to survival [21]. This qualitative study outlines key contributors to delayed breast cancer diagnosis in Tanzania from the perspective of patients currently undergoing therapy for breast cancer. Barriers and facilitators to timely diagnostic evaluation of symptomatic breast cancers in Tanzanian women were complex and multifactorial, encompassing individual, interpersonal, sociocultural, and health system factors.

At the individual-level, lack of knowledge and misconceptions about the signs and symptoms of breast cancer was a key contributor to delayed diagnosis. All participants admitted to self-detection of a new breast lump or swelling, but most were unaware that the symptom could be due to breast cancer, especially in the absence of associated pain. Some participants reported a misconception that cancer was a communicable disease; since they had no known close contact with a breast cancer patient, they erroneously believed they were unlikely to contract the disease. Most decided to ignore their symptom and did not seek medical attention immediately. This lack of breast cancer awareness has been similarly described in other countries in sub-Saharan Africa, including Rwanda, Kenya, Ethiopia, Ghana, Nigeria, and Malawi [22–27] as a major factor in delayed breast cancer diagnosis. A recent multi-country African Breast Cancer Disparities Outcomes study (ABC-DO) found that poor cancer awareness and education was significantly associated with more advanced stages of breast cancer at diagnosis [28]. These findings highlight the importance of increasing breast cancer awareness in the community and educating women in recognizing signs and symptoms of malignancy.

Our study suggests that development of educational efforts to promote breast cancer awareness should extend beyond the patient level to the health provider level. Several participants reported that when they did seek medical attention for their symptoms, they faced barriers within their local healthcare system. For example, participants received false reassurance from their primary health care provider that their symptom was nothing worrisome. Others were sent home with analgesics or antibiotics. Participants reported difficulties in obtaining referrals from their local providers to higher-level facilities with cancer diagnostic capabilities. Our findings are in keeping with other studies conducted in sub-Saharan Africa which similarly demonstrated health system-related barriers with inappropriate appraisal and management of breast symptoms by local health providers [25, 26, 29]. Strategies are needed to ensure providers at primary care facilities are trained in breast symptom evaluation and the clinical breast exam. This may reduce misdiagnoses and accelerate referrals of suspected cancers to appropriate levels where a diagnosis can be made. Focused training of local health aids, such as midwives and nurse assistants, in the evaluation of cancer signs and symptoms has been demonstrated to be an effective intervention to down-stage breast cancers in rural Tanzania [30]. Additionally, ongoing expansion of healthcare services in Tanzania by opening new health centers could increase availability and accessibility of healthcare. Healthcare delivery improvement, and improved coordination and facilitation between primary care facilities and referral hospitals may help address the delay participants faced due to bureaucracy in the referral systems.

However, even after referrals were obtained, participants reported financial barriers to prompt diagnostic evaluation. Many described struggles to gather funds for recommended tests, further delaying their diagnosis. In addition to medical costs, participants reported challenges in obtaining money for travel and temporary accommodation near the referral hospitals. It should be noted that most of the Tanzanian community do not have health insurance, necessitating out-of-pocket payments for medical services. As a result, after being referred many had to return home to raise money. The contribution of financial constraints to delayed diagnosis of breast cancer and possibly other cancers is seen in several other low to middle-income countries especially in sub-Saharan Africa [23, 31]. It is also seen among low-income communities even in affluent societies. The government should aim to improve breast cancer patients’ access to health care by making cancer services, including diagnostic services, financially accessible to all. Tanzania has been making efforts toward universal health coverage, but many challenges remain [32]. Continued expansion of health insurance coverage with preferential targeting of the poor will help increase equitable access and health outcomes [33].

Sociocultural barriers to timely breast cancer diagnosis included use of alternative treatments rather than medical evaluation. Some participants reported first seeking care with a traditional healer. In Tanzania, such services are often widely available and relatively affordable with promises of immediate relief. In addition, while many participants benefited from prayer and religion as a source of comfort, some believed that their breast symptoms could be healed by prayers alone, further delaying diagnostic medical evaluation. Studies in other sub-Saharan African countries have found that alternative therapies, such as the use of traditional healers, were a common contributor to delayed diagnosis of breast cancer [22, 27]. In addition to educating women on the importance of proper medical evaluation of breast symptoms, working with churches and traditional healers to raise awareness about the symptoms of breast cancer and the importance of early diagnosis may benefit women who seek alternative care.

Similar to other studies from sub-Saharan Africa, a fatalistic view of cancer, fear of cancer diagnosis, and fear of public stigma were barriers to seeking evaluation and diagnosis [23, 34, 35]. Participants were afraid to be given a cancer diagnosis and feared undergoing the recommended surgeries to confirm malignancy. Participants were scared to be labeled as having cancer in their communities, fearing gossip and negative comments from the public. Historically poor cancer outcomes in Tanzania have instilled a fear that it is an incurable disease and many equate it with being given a death sentence. Education and awareness in the community that breast cancer can be a curable disease with recent improvements in cancer care services and that earlier diagnosis enhances the chances of survival is essential to encourage patients to seek timely medical evaluation without fear of being stigmatized. This information may be used to inform the government and other stakeholders to develop strategies to reduce stigmatization of not only breast cancer but also all other cancers in Tanzania.

Lastly, we found that the advice and influence of family members and friends played a major impact in participants’ healthcare-seeking behaviors, serving both as facilitators and as barriers to timely cancer diagnosis. Family and friends were often the sole source of social and financial support. Given the impactful role of family and friends, breast cancer awareness and educational strategies should not simply target women at risk of developing breast cancer, but campaigns highlighting the importance of early breast cancer diagnosis should be disseminated to the Tanzanian population as a whole.

**Limitations** of this research should be noted. First, this was a small exploratory study with a sample limited to those receiving care at a national referral hospital in Dar es Salaam, Tanzania. Many Tanzanian women with breast cancer may never reach this national referral hospital due to myriad barriers to breast cancer care; as the city of Dar es Salaam is not easily accessible by the majority rural population of Tanzania, our results may not be generalizable. Thus, this report may reflect some selection bias. In addition, participants were diagnosed months to years prior to being interviewed; therefore, data may be subject to some recall bias, and discussions of historic events may be affected by the current knowledge of their breast cancer diagnosis. For the purposes of this this qualitative study, the primary source of data was from the interviews. Quantitative review of the medical records was not conducted; details of clinical and surgical cancer stage, treatments, and outcomes were not collected. Although the results of this study may not be generalizable to all Tanzanian women, our results were similar to studies carried out in other sub-Saharan African countries and the key findings may be used to improve cancer diagnostic care more broadly.

Our study was qualitative, as we aimed to deeply explore the experiences and journeys of Tanzanian women with breast cancer. To our knowledge, while similar studies have been conducted in other countries in sub-Saharan Africa, there is very little research on the causes of delayed diagnosis of breast cancer in Tanzania [36]. The results of this qualitative study offer hypotheses that could be tested in larger multicenter quantitative studies to assess the generalizability of our findings.

In summary, lack of basic knowledge and awareness of breast cancer, fear and stigma, financial barriers, and local healthcare system barriers were common factors which contributed to delayed diagnostic presentation of breast cancer. The influence of friends and family also played key roles as both facilitators and barriers.

## Conclusions

Our study highlights key areas of focus to guide future research, practice, and policies. We aim to use this information to inform and develop strategies to improve earlier diagnosis of symptomatic breast cancer in Tanzania. Specifically, increasing breast cancer awareness, reducing stigma, and educating individuals on the recognition of breast signs and symptoms, for example through community outreach and educational campaigns, may empower women and their primary healthcare providers to diagnose cancers early and improve survival. In addition, given the strong influence family and friends, these educational and outreach endeavors should target the Tanzanian population as a whole, not just those at risk of developing breast cancer.

## Supporting information

S1 File Interview transcripts. (ZIP)
