# Supplementary material for: Delayed diagnostic evaluation of symptomatic breast cancer in sub-Saharan Africa: A qualitative study of Tanzanian women
Source: PLoS One. 2022 Oct 6;17(10):e0275639. doi: 10.1371/journal.pone.0275639 (PMC9536581; doi:10.1371/journal.pone.0275639)
Supplement: S1 File — (ZIP) [file pone.0275639.s001.zip › Interview Guide_ENGLISH.docx]

**Evaluation of factors related to delay in presentation for imaging of women with breast cancer in Tanzania**

## **Interview guide**

Recorder _______________________Language ______________________

Time: Start ___________ End ______________

Information about the study

Informed consent

Turn on the recorder

1. **Background Information**
   1. Date of birth…………………………..
   2. Gender …………………..
   3. Region of residence………………………
   4. Marital status……………………………..
   5. Number of children……………………….
   6. Level of education……………………………..

What is your highest completed level of education: (Primary? Secondary? High School? University Undergraduate? Etc.)

- 1. Occupation
- Do you have an occupation? If so, what is your occupation?
  1. Time/distance to the nearest health care provider,
- How far is the nearest health care provider from your home/residence? How many miles? How much time does it take to travel?
  1. Method of transportation to the nearest health care,

How do you travel to the nearest health center? Walking? Automobile? Bus? Airplane?

How did you travel to MNH? Walking? Automobile? Bus? Airplane?

- 1. Any computer/internet access,

Do you have access to a computer or phone?

Do you have regular internet access?

- 1. What were the patient’s breast signs/symptoms?

What kinds of breast symptoms did you initially have? Lump? Pain? Nipple discharge? Skin changes (warm, red, itchy, scaly, **dimpling**, ulcer or wound? Other?)

- 1. Who detected the signs/symptoms,

Who first noticed the breast problem? Yourself? Your partner/husband? Health care provider? MEWATA Campaign? Other?

- 1. Any use of traditional medicine prior to seeking medical care.
     (type of medical care was first sort)…..traditional, conventional, alternative, etc)

What kind of health provider did you go to first for this problem? Traditional healer? GP? Nurse? Other? What hospital/clinic?

If the first provider was a traditional healer (or NOT a nurse or doctor), how long after noticing the breast problem did you go to see a nurse or doctor?

- 1. time between symptom onset and seeking of medical care,

When did you first notice the symptom or problem with your breast? How many days/weeks/moths/years ago. (or do you remember the approximate date?)

Approximately how long after noticing the problem did you seek medical care or go to a health care provider?

- 1. time to breast imaging evaluation,
- How much time after noticing your symptoms did it take imaging evaluation?
- What imaging investigation did you do?
- Did you have mammography or breast ultrasound to evaluate your breast problem? If so, when?
  1. any past personal history of breast cancer,
- Have you ever been treated for breast cancer in the past before this diagnosis?
- If so, when?
  1. Any family history of breast cancer,
- Do you have any history of breast cancer in your biological family members? If so, who? Grandparents, biological parents, siblings, uncles and aunties? Cousins?
  1. What were you told about the stage of breast cancer at the time of diagnosis and initiation of treatment (or record from case notes)

1. **factors leading to delay imaging presentation**

- In your opinion, what time lapse would you consider late in getting breast cancer diagnostic results and initiation of treatment?
- What were the factors that prevented you from seeking medical care sooner?

(probe:

1. personal – lack of awareness, beliefs on alternative type of healing, stigma, time, economic status, family support, lack of power to make decision {needing permission from spouse}
2. healthcare system (capacity of healthcare workers to recognize early symptoms, availability of diagnostic tools etc.)
3. referral system (timeline for overall referral process)

(After the patient responds to the above question, interviewer can probe further by asking the following questions below) Did any of the following prevent you from seeking medical care at a hospital/clinic sooner?

1. To assess the psychosocial factors associated with late presentation to imaging services

*Probes* – *fear, stigma, need for permission to seek medical care*

1. To determine how breast cancer information and perception contributes to late imaging

*Probes – sources of breast cancer information, awareness and knowledge on risk factors, knowledge on breast mammography and ultrasound*

- Do you know how to examine your breasts for lumps?
- Before you developed this problem, did you ever check your breasts for lumps?
- Before you developed this problem, had you ever had a breast exam by a health provider (including MEWATA campaign)?
- Do you see a health care provider regularly?
- What was your knowledge of breast cancer prior to developing this problem?
- How did you learn about breast cancer? (If patient unable to answer, ask: TV, Internet, health provider, friend/family, newspaper, traditional healer, radio, doctor/nurse, minister?)

1. To assess how health seeking behaviour, preference for alternative treatment, culture and norms contributes to late breast cancer imaging (**probe barriers and facilitators)

*Probes – accessing health facility, treatment preferences tradition medicine vs medical care*

-Did you know needed to see a doctor?

-Did you fear being examined by a health provider/doctor?

-Did you think it would go away?

-Were you too busy at home or at your job?

-Fear of breast cancer diagnosis? (I did not want to know if I had cancer)

-Fear or treatments (Fear of chemotherapy? Fear of surgery/losing your breast?)

-Fear of dying from treatments?

-I did not want anyone else to know I had this breast problem

-Delay due to using a traditional healer or oriental medicine (or minister for prayer)

-I was afraid hospital bills would be too expensive

-It was too far to go to the hospital/clinic

-It was too expensive to travel to the hospital/clinic

-Did you have to wait long for authorization/commission/permission for medical care? (How long did you wait for permission)

1. To assess patients’ satisfaction with health providers knowledge and skills in breast cancer imaging breast cancer at different levels

*Probes – At which level were you referred for imaging, where?*

We have now come to the end of our interview. Thank you so much for your time.

Do you have anything else that you would want to add as the causes of delay for you or other women to seek health care?

Thank you
