# Supplementary material for: Delayed diagnostic evaluation of symptomatic breast cancer in sub-Saharan Africa: A qualitative study of Tanzanian women
Source: PLoS One. 2022 Oct 6;17(10):e0275639. doi: 10.1371/journal.pone.0275639 (PMC9536581; doi:10.1371/journal.pone.0275639)
Supplement: S1 File — (ZIP) [file pone.0275639.s001.zip › Interview Guide_SWAHILI.docx]

## **Muongozo wa Majadiliano**

Recorder _______________________Language ______________________

Time: Start ___________ End ______________

Information about the study

Informed consent

Turn on the recorder

1. **Taarifa za mwanzo**
   1. Tarehe ya kuzaliwa …………………………...
   2. Jinsia …………………...
   3. Mkoa unapoishi………………………
   4. Hali ya ndoa……………………………...
   5. Idadi ya watoto……………………….
   6. Kiwango cha elimu……………………………...
   7. Ni kiwango gani cha juu cha elimu ulichofikia? (*Elimu ya msingi, kidato cha nne, kidato cha sita, chuo kikuu? Etc*)
   8. Umeajiriwa/una kazi? Kama ndio, ni kazi gani?
   9. Kutoka mahali unapoishi, ni muda unatumia au ni umbali gani kufika kituo cha karibu cha afya? *(Dodosa: Umbali* ***kwa maili/kilomita*** *na* ***mda gani*** *kufika kituo cha afya cha karibu?)*
   10. Ni aina gani ya usafiri itumikayo kukufikisha kituo cha afya cha karibu? *(Dodosa: aina ya usafiri – kutembea, baiskeli, pikipiki, gari, meli, ndege nk)*
       - Ulisafiri vipi kufika MNH?
   11. Mawasiliano: Uwepo wa kompyuta au upatikanaji wa mtandao

*- Una kompyuta au simu? na Je unapata mtandao muda wote?*

*- Kama ndio, je unafahamu kuwa unaweza kupata taarifa za afya (saratani) kwa kupitia mtandao wa simu?*

*- Maoni juu ya upatikanaji wa taarifa za afya kwa kupitia mtandao?*

- 1. Je unaweza kunisimulia jinsi ilivyokuwa, hadi unagundua kuwa una satratani ya titi?
     Je ulikuwa/uliona na dalili gani za kwanza za ugonjwa? *(Uvimbe? Maumivu, chuchu kutoa maji/damu? Mabadiliko kwenye ngozi ya titi kama joto, kubadilika rangi, mibonyeo/kudumbukia, kuwasha, kutoa magamba, kidonda n.k.).*
  2. Je ni nani alikuwa wa kwanza kugundua dalili hizo za tatizo la titi lako? (*Dodoso: Wewe mwenyewe? Mwenzi/mume wako, mhudumu wa afya? Kupitia kampeni za Madaktari wa MEWATA, nk)*
  3. Ni lini uligundua kuwa na dalili za ugonjwa kwenye titi/matiti yako? (Ni siku/wiki/miezi/miaka mingapi iliyopita? (unaweza kukadiria ni tarehe ngapi?)

1. Kwa kukadiria, ilichukua mda gani tangu ulipogondua tatizo la titi na ulipoamua kufuata huduma ya afya au kumuona mhudumu wa afya?

*(Ikiwa mgonjwa hataweza kutoa jibu Dodosa kama ni <wiki 1, <mwezi 1, kati ya mwezi 1-3, kati ya miezi 6-12, kati ya miezi 12 hadi 18, kati ya miezi 18 hadi 24, Zaidi ya miezi 24 iliyopita) AU <wiki 1, <mwezi 1, kati ya mwezi 1-3, kati ya miezi 6-12, kati ya miezi 12 hadi 18, kati ya miezi 18 hadi 24, Zaidi ya miezi 24 iliyopita)*

1. Huduma gani ya kwanza ya tiba uliyoipata baada ya kuona/gundua dalili za ugonjwa? (aina ya huduma uliyopata kwanza) ……waganga wa kienyeji, tiba asilia, tiba mbadala, tiba za hospitali)
   1. Je ulishawahi kufanyiwa uchunguzi wa picha (mamograph au ultrasound)

- Baada ya kugundua dalili, ni muda gani ulipita hadi kufanyiwa uchunguzi wa picha kwa tatizo lako la titi?
- Ni vipimo gani vya picha ulivyofanyiwa (*Dodoso: mammograph? au ultrasound?*) Kama ndio, lini?
- Ulisafiri kupata kipimo hichi?
  1. Utumiaji wa dawa asilia au tiba mbadala kabla ya kuanza kufuatilia matibabu ya hospital
- Kama hapana, kwa nini?
- Je ni nani ulimuona kwanza kupata huduma hili tatizo lilipo kuanza? Mganga wa jadi, Daktari, Muuguzi, au wengine (taja)?
- Hospitali au kliniki gani?
- Kama mtu wa kwanza kumuona ni mganga wa jadi, ni mda gani umechukua tangu umegundua tatizo la titi/matiti hadi kumuona daktari au muuguzi?
  1. Historia binafsi ya saratani ya titi.
- Umewahi kutibiwa saratani ya titi hapo awali? Kabla ya ugunduzi huu wa sasa?
- Kama ndio, lini?
  1. Hatua ya saratani ya matiti kipindi inagundulika na kuanzishiwa matibabu
     (kutoka kwenye taarifa za matibabu)
     - Je ulitaarifiwa hatua ya saratani ya titi uliyo nayo?
  2. Historia ya saratani ya matiti katika familia
- Una ndugu yoyote wa damu aliyewahi kuwa au mwenye saratani ya matiti? Kama ndio, nani? *(uhusiano; babu, bibi, baba, mama, kaka, Dada? Binamu? Mjomba, Shangazi? n.k)*

1. **Sababu zinazopelekea kuchelewa kufika kwa uchunguzi wa picha**
   - - Kwa maoni yako ni muda gani ukipita, utasema umechelewa kupata majibu ya kuhakiki kuwa una saratani ya matiti na kuanza matibabu?
     - Ni vitu gani vilipelekea kuchelewa kufika na kupata huduma za matibabu hospitalini?
       *(Dodosa:*
2. *Sababu binafsi – kutokuwa na uelewa, imani za tiba mbadala, unyanyapaa, mda, uwezo wa kifedha, ukosefu wa msaada wa familia, kutokuwa na uwezo wa kutoa maamuzi {ruhusa kutoka kwa mume})*
3. *Mfumo wa huduma za afya - Uwezo wa kitaaluma wa wahudumu wa afya kugundua dalili za mwanzo, uwepo wa vifaa vya ugunduzi wa picha nk)*
4. *Mfumo wa rufaa (muda kiujumla hadi kupata rufaa nk.)*
   - - Ni vitu gani vilikupa motisha au kukusaidia kufuata huduma za afya mapema?

(Mgonjwa akijibu swali hapo juu dodosa Zaidi kwa kuuliza maswali yafuatayo) Kuna chochote kati ya hizi kilikufanya usifike hospitali mapema?

1. Kutathmini sababu za kisaikolojia zinazoambatana na kuchelewa kufika kwa ajili ya uchunguzi wa picha

*Dodosao* – woga/hofu, unyanyapaa, kuhitaji ruhusa kwenda hospitali

1. Kutambua jinsi taarifa na mtazamo juu ya sartatini ya matiti vinachangia kuchelewa kufanya uchunguzi wa picha

*Dodosa – vyanzo vya taarifa juu ya saratani ya matiti, uelewa na ufahamu juu ya visababishi vya saratani ya matiti, uelewa juu ya mammography na ultrasound*

- *Unajua namna ya kujichunguza uvimbe kwenye matiti?*
- *Kabla ya kupata hili tatizo, uliwahi kuchunguza matiti yako kama kuna uvimbe*? *Kama ndio, ulichunguza vipi?*
- *Kabla hujapata hili tatizo, ulishawahi kufanyiwa uchunguzi na mhudumu wa afya (pamoja na madaktari wa MEWATA)*?
- *Je huwa unawaona wahudumu wa afya mara kwa mara*?
- Ufahamu/uelewa wako juu ya saratani ya matiti ulikuwaje kabla ya kupata tatizo hili?
- Ulijifunza vipi juu ya saratani ya matiti?
  Dodosa: TV, mtandao, magazeti, redio, muhudumu wa afya, mganga wa jadi, daktari au muuguzi, mchungaji n.k

1. Kutathmini namna tabia ya kufuatilia huduma za afya, tiba mbadala, utamaduni na kanuni vinachangia kuchelewa kupata huduma ya uchunguzi kwa picha

*Dodoso – upatikanaji wa kituo cha afya, mapendekezo ya huduma kati ya tiba mbadala/ya jadi na hospitalinie*

- je ulijua kuwa unahitaji kumuona daktari?
- Je ulikuwa ukiogopa kuchunguzwa na mhudumu wa afya au daktari?
- Je ulifikiri dalili/uvimbe ungeisha wenyewe?
- Ulikuwa na majukumu/mambo mengi nyumbani au kazini?
- Uliogopa kujua kuwa una saratani ya matiti? (Sikutaka kujua kama nina saratani)
- Uoga wa matibabu (Uoga wa kemo? Uoga wa kufanyiwa upasuaji/kupoteza titi?)
- Uoga wa kufa kutokana na matibabu?
- Sikutaka mtu mwingine yoyote kujua kuwa nina tatizo hili la matiti
- Nilichelewa kwasababu nilikua Napata tiba asilia (au maombi)
- Nilikua naogopa gharama ya matibabu itakua juu
- Ilikua mbali sana kufika hospitalini/kliniki kupata matibabu
- Ilikua gharama kubwa sana kusafiri kufika hospitalini/kliniki
- Ulihitaji kusubiri mda mrefu kupata kibali/ruhusa/msamaha kupata huduma ya afya? (Ulichukua mda gani kupata ruhusa)

1. Kutathmini uridhishwaji wa mgonjwa juu ya uelewa na ujuzi wa watoa huduma ya afya katika ngazi mbali, juu ya vipimo vya picha vya saratani ya matiti
   (*Dodosa – Katika ngazi ipi ya afya ulipelekwa kufanya kipimo cha picha ya matiti; ulifanyia kipimo hicho wapi?*

Sasa tumefikia mwisho wa mahojiano yetu. Nashukuru kwa kunipa muda wako ili nipate maoni kuhusiana na sababu zinazopelekea wagonjwa kuchelewa kupata huduma za afya (ugunduzi wa ugonjwa pamoja na tiba).

**Je una kitu kingine chochote cha kuongezea kama sababu kuu zinazowapelekea kina mama wengi au wewe mwenyewe kuchelewa kuanza kupata matibabu ya hospitali?**

**Aksante!**
