# Supplementary material for: Delayed diagnostic evaluation of symptomatic breast cancer in sub-Saharan Africa: A qualitative study of Tanzanian women
Source: PLoS One. 2022 Oct 6;17(10):e0275639. doi: 10.1371/journal.pone.0275639 (PMC9536581; doi:10.1371/journal.pone.0275639)
Supplement: S1 File — (ZIP) [file pone.0275639.s001.zip › Minimal Data-Interview Transcripts.docx]

**CASE 1**

I: Madam, so what is your name?

R: My name is -----.

I: And when were you born?

R: I was born on the -----.

I: And where do you live?

R: I live in -----.

I: Alright -----, are you married?

R: yes.

I: you have a husband and how many children?

R: I have four children.

I: Alright.

R: My husband is dead.

I: I am sorry.

R: Thank you.

I: Did he die a long time ago or a bit recently?

R: He died in March last year.

I: Alright. So -----, what is your highest level of education?

R: I reached form four of secondary education

I: Form four? ,,,So are you working?

R: I was working but I have retired.

I: Alright, what was your job?

R: I was a teacher.

I: A secondary school or primary school teacher?

R: A primary school teacher.

I: Alright, so ----- the objective of this study is to know as to why woman are late to seek and start the treatment of breast cancer. So I would like to know first of all, what is the distance between your home and the nearest health cenntre?

R: Truly I am not very far away from the hospital because I live in ----- and the ----- hospital is near around here.

I: Are you near ----- hospital?

R: Yeah, I am near ----- hospital.

I: Alright

R: Yes.

I: Also do you have any means of communications, such as mobile phone?

I: And do you have such equipment as television or radio at your home?

R: I have the television and the radio as well.

I: So have you ever heard about breast cancer?

R: Yes, I have ever heard.

I: Where have you heard it?

R: I have heard it on the radio. Whenever I watch the TV I see them announcing that there is breast cancer. They explain explicitly that one can know that they have breast cancer by going to see a doctor so that they get investigated. Another way you can detect that you have breast cancer is when you notice that your breast has a difference, it is bigger than the other, it is bigger than it was initially, and then maybe you can notice it is bleeding on the nipple or presence of an ulcer.

I: For you, what was your first symptom?

R: My first symptom which I experienced is that I was feeling pricking sensation on my breast. It went on and I thought may it was just normal and that was when I had stopped breastfeeding when I had the symptoms. Later on I noticed that breast had stroke on the left side.

I: It got stroke?

R: Yes, for the time being I was at the hospital but later on I noticed that the nipple was also bleeding. At that very moment of stroke, I noticed that my cloth which I was wearing it was soaked, why was it soaked? Just to realize it is blood aah! I asked the doctor, ‘why am I bleeding on the breast?’ he asked me if I was breastfeeding, I told him, ‘I have even stopped menstruating’. He told me that they would do investigation, it is when they detected that I had breast cancer.

I: Did you see the first signs while you were sick already or you experienced them even before being sick?

R: Before knowing that it was breast cancer, I didn’t know, I was just feeling pricking sensation on my breast but was unable to check myself.

I: Do you know how to check yourself?

R: I don't know. I was not knowing absolutely anything.

I: About checking yourself?

R: Yeah, I didn’t know.

I:, what did you do when you noticed blood?

R: When I noticed that blood I rushed to the hospital.

I: Before that, you told me that you had pricking sensation.

R: Yeah, I had pricking pain but it was not continuous, it was an on and off pricking sensation, therefore I thought that it was not a very harmful disease but there was swelling inside.

I: And were you able to touch the swelling?

R: I was not examining myself. you know the breast is big, so you cannot be knowing if there is something inside. I was not quick to examine myself there inside I therefore thought that it was just saw it to be a normal condition for a woman to feel a little problem and then it is off, but then there was a growing swelling inside without me knowing. In the long run, the symptom which led me to know that I was sick is after noticing blood on the nipple.

I: And how long was it from the moment you started feeling that pricking pain initially?

R: Actually, it might have taken a year, and it is not that it was there all days, no. it was happening a little two or three days and then it was stopping. You stay for a while then it appears another day. So, I was not quick enough to notice that it was a very harmful issue.

I: So, the one who discovered it for the first time when found it out, did you meet a doctor or?

R: I met a doctor at ----- hospital

I: Didn’t you pursue other services such as treatment?

R: Frankly speaking, I didn’t get traditional services, I didn’t participate. I was not quick to agree on that.

I: But you were told that there are such kind of treatment, right?

R: I had been advised a lot that there are traditional treatments. I have even been advised by my relatives but me I thought it is better to get investigation-based treatment.

I: From hospital. ….

R: From hospital, rather than going to the tradition, indeed that thing….

I: When did you meet your relatives and tell them that you have such a problem?

R: When the breast was bleeding.

I: That is when you told them?

R: It is when it old them, ‘my fellows that my breast was bleeding’. They said, ahh maybe it is a swelling in the breast, I told them that I don't know because it was equal to the other one. But when I examined it, I noticed that the nipple was retracted, totally embedded inside but the other one the nipple was just in good condition. But this one is bleeding and the nipple is retracted. I discovered that it was different it is when I said that I had to to rush to the hospital.

I: So, okay….?

R: Frankly speaking, I was participating much in prayers than in any other advice. I was only getting prayers and hospital, but I didn’t take part in any traditional medicine, no. I must be sincere.

I; When you started your treatment, did you get any imaging investigations, like ultrasound?

R: yes.

I: How long did it take from the moment you had the symptoms initially to when you got the investigation?

R: It is about one month, because I was going to hospital and I was late to take action because I had a heart problem. So all my mind was directed towards the heart problem, I thought probably it was the heart problem which had contributed to the bleeding of the nipple. So I was very hesitant to investigate why was the nipple bleeding, I thought no, probably it is due to my blood pressure. Because since my childhood they were telling me that I had cardiac problems. Therefore, I thought may be the heart has caused this problem, maybe it is the holes which were repaired that is why blood was coming out here. Those were my thoughts which contradicted me such that without having investigations done. Later on I said I should go and have investigations at the hospital.

I: So, it took time a bit, right?

R: Indeed, it took time.

I: Like how long?

R: It reached even three months.

I: Okay, do you think you were late a bit to come to get treatment?

R: I was very late, not a little.

I: When do you think you would have come for treatments?

R: The moment I was feeling the pricking pain I was supposed to take action. I should have thought, what are these for? But for took it on my own, to treat myself by using my own mind, it is when I was very late. I was thinking maybe it was the heart, maybe it was just a simple condition that a woman should passing through, indeed this maybe led me to come late. I was diagnosed to have cancer already at stage four.

I: Stage four? ….

R: Yeah, it is likely that my on and off illness was due to the cancer, but I had never thought of it. Actually I had never thought the diseases of cancer in my mind

I: I wanted to ask you also about that, did you know anything about breast cancer?

R: I didn’t know even gastric cancer.

I: You told me about the radio you listen to.

R: I listen to the radio but I didn’t think that I can get such a thing…. I don't know why I was so such stupid.

I: You didn’t think about….

R: To think that one can get it. Because I was knowing probably it is a communicable disease and I had no patient having such a disease,….. so getting such a disease would be difficulty. But then it is not a communicable disease, it just happens.

I: Did you ever have a relative who happened to have such a disease before?

R: Truly I have not confirmed that one has a certain disease and it is a cancer. Because most of them just died, you are told, ‘that one had this disease, was vomiting, was vomiting blood, so and so’, my mother was among them. I am last born of my mother but I was living with my mother for seven years. Her problem is that she was her uterus was removed because she was bleeding heavily.

I: Was her uterus removed?

R: Her uterus was removed, it was removed but I was still young I didn’t know the problem I was just knowing it was abdominal disease of a woman. Later on my mother went on with the problem of abdominal pain, but they didn’t say it to be cancer……

I: To be a cancer.

R: To be a cancer, I was not knowing that. The late moments of my mother’s severe illness was severe pain till she was vomiting blood. My mother vomited a lot of blood.

I: Before death.

R: Before her death. And that was her end, she died. Now one day I was watching a TV, I heard that you might be having a swelling the uterus and that it is better to remove the uterus and start treatments early. It is when I therefore realized and thought that maybe even my mother had cancer.

I: Was it after you had started being sick?

R: No, it was not yet.

I: It was not yet, right?

R: Aaah, about being sick, I was being sick already. But I had not realized that I might be having it… I didn’t notice the symptoms like those of my mother, those of abdomen, no no…. I didn’t notice that. Therefore, I didn’t know that I can get it from my mother, neither did I knew that it is not transmissible disease. But as far as my mother is concerned, the symptoms are likely it was a cancer.

I: ----- why didn’t you go to hospital, for testing when you had the pricking pain, were you getting other kind of services, like traditional treatment?

R: I didn’t have any other services, I did not have traditional treatment, except that I was getting treatment for heart. That is how my thoughts were. My thoughts I knew it was the problem of cardiac failure was the one which led me to bleed on the breast, that is what led me to be late.

I: So where did you do the ultrasound investigation?

R: I did it at Bombo hospital, they told me I had a swelling in my breast.

I: What did they say later on?

R: They said it had already been big. But thereafter they said they would take a biopsy for investigation.

I: Was it done there at -----?

R: No, it was not done at -----. I told you that I have a heart problem, then I had operation on my left breast. I had a negative belief that if my breast is removed I can die. You see another problem.

I: Was it only you were thinking that or even your relatives as well?

R: I was thinking it myself. Then my relatives, there contribution is that they told me it is better they sell a cow so that they take me to a certain traditional healer. The Idea of traditional healer I didn’t want it I was much against it. But I gave priority to my idea that, I have heart disease then I should have an operation, no. so I remained with that condition, being afraid, I went on wasting time. After I was in bad condition, I even lost strength, I had little blood, I said now I want to go to -----. Because I know there is a doctor called Professor ----- who deals with heart issues. So I went there for heart investigations.

I: There at -----?

R: -----, I didn’t go there for breast investigations. arriving there, professor ----- told me,’mum you don't have heart problem, your heart is still health’. I told him, ‘doctor, I have taken medications since I was a child, a girl studying primary school.

I: Didn’t they investigate you on the breast there?

R: Listen now, I tell you how it was. Because I went in a bad condition, I was admitted due to heart problem. I was admitted at ICU. I was attended to and I got better, later on I said no, my breast is bleeding. That moment is when they started breast investigation. They told me that they had to look for the problem in my breast. They did ultrasound then tissue and I waited for three months for the results or to choose to be taken blood for investigation.

I: How long did they did they say it will take to results?

R: They said the result would delay for three months. So, I told them, ‘should I wait for three months while at the same time I am still bleeding and I am such tired, will I reach the three

months?’ I took blood instead for investigation, tested it and said it is true it is a cancer at stage four.

I: And at that juncture was it that you had started noticing swelling in the armpits?

R: There was like lymph node swelling.

I: what then after?

R: Thereafter, the professor advised to have my breast removed. I told the professor, ‘If my breast was cut because I would die. If that is the case, then just leave me to die’. I was with my daughter, they told me, ‘mum, according to the condition you are in now, after two weeks your condition might be worse and threaten your life, so we plead you mum to agree to have your breast removed for your safety.’ She advised me well, saying, ‘he has an intention of making you better, death is a promise. The doctor is not treating you in order to kill you” so my daughter, advised me to agree.’

I: Is she your child?

R: Yeah, is my child. ‘agree if there is a possibility of breast to be removed so as to prolong life. Because if we leave this, the cancer will spread’. My daughter told me, ‘if you leave the breast you will die, if you have the operation done you die, so agree the treatment, at least you add sometime’. So, I agreed and had my breast removed.

I: I am very sorry.

R: thank you.

I: Maybe I would like to know the role of your relatives; probably why didn’t they advise you early like your daughter. Why didn’t they tell you needed treatment the moment you said you had pricking pain?

R: The role of my few relatives is that they were giving me advice, but a few of them told me if I had my breast removed and I would die and they won’t be involved in it, so the relatives themselves were contradicting each other.

I: So, the relatives were not ready, right?

R: They were not ready. I was left with my only one relative whom I am with her here till now. She told me, ‘my young sister let us go, death is a promise, there is no a corps which rots inside, you will be buried.’

I: How long did it take that you decided at to at least start of treatment?

R: I think it took some time. It took time because after knowing that I had cancer, we stayed about two months later.

I: While you were waiting to know…

R: While I was waiting what kind of treatment.

I: during that moment you were waiting, did you have any kind of treatment?

R: I had no any treatment.

I: During that moment, in the midway?

R: I had no any kind of treatment

I: How about the medical costs, do you think it has got a role also?

R: Medical costs for cancer frankly speaking, as far as I am concerned, it was not costly because I had gone there at Ocean Road where I was being treated for free. Except that when I was at ----- it was expensive because I was paying.

I: At -----?

R: Yeah.

I: How about -----?

R: At ----- also I was paying at the moment. So, there were high costs indeed. Moreover, taking into account the transport costs, food costs when you are away, in fact the expenses are high.

I: You said you had heart problem, right?

R: Mmmh

I: How about it? were you following up the treatment….

R: I was following the treatment but professor ----- told me to stop all the medications which I was claiming to have cardiac failure. He told me that I did not have a heart problem. He himself is a heart surgeon.

I: Was that before you had breast disease or before?

R: Yeah, I already had the breast disease and I was in intensive investigations.

I: I wanted to know because patients with heart failure have the habit of going to hospital now and then.

R: Yes, I was attending clinic.

I: How was it to you?

R: I used to go to clinic.

I: Where is the clinic?

R: It is at ----- hospital.

I: Then why didn’t you explain to your doctor about the new problem?

R: He knew it, I told him the problem of bleeding. It is when he told me to go and do the ultrasound so that I can see what is the problem, what is inside the breast.

I: but you didn’t tell him initially during the moment you had pricking pain, right?

R: No, actually I didn’t tell him. Me myself I was thinking that it is a heart disease, that is the problem of mine.

I: And when was it when you learned more about the disease of cancer, about knowing how to examine yourself, and where did you get such information?

R: I knew how to examine myself while I was at -----.

I: At the moment had the disease already, right?

R: I had the disease already, being admitted at -----. And now I was admitted at ----- for treatment. It is when they said, ‘if you want to detect a swelling in a breast, lie on your back and then try to do this way, you will find a hard thing inside, otherwise go and have regular checkups to the specialists at the hospital.

I: Ok R, I think we are about to end our talk, but I would like to know, you told me that you think you are late to come to get the treatment.

R: Yes

I: And it is due to the fact that you didn’t have thorough understanding, right?

R: I had no education.

I: Maybe, do you think there is any other thing which led you to come late to hospital?

R: yes. Another thing which led me to be late is about income.

I: Income?

R: Because if you consider the fare from ----- to -----, how to live there and you don't have relative there, now you can see. Then you put into consideration, according to the condition I had, I was unable to go there on my own, it was a must I have more than one assistant, how will they live, what will they eat. That was a very big challenge. It was a must that I go with people according to the condition I had reached that time, I had to go with two people, one to hold me and another to carry the luggage we went with, such as bags. According to my condition, one person was not enough. I was in bad condition; I was so late such that I was in bad condition. So the expenses to enable me to get the services there it was a challenge, to stay at a lodge.

I: Were you staying at a hotel, a lodge?

R: My assistants had to stay there, I was admitted.

I: At the moment you were at -----, right?

R: I was at -----. From ----- to ----- I had to be admitted as well, where would my assistant stay? It was a challenge.

I: And according to your experience, from the moment you got sick, I think you told me that you have received treatment at different places, such as -----, -----, you came up to -----, -----. Maybe here in the midway in the health services, is there anything that made you to be late, in the health system? In provision of service, healthcare providers, things like that?

R: One thing which made me to be late is when I was at ----- hospital, if they had taken the investigation early, I would have been known early.

I: Why didn’t they take the investigation early?

R: I don't know the reason; I think because they don't have the equipment. I think they don't have the equipment. Because as for the issue of heart, you are being told that I was using medications for heart disease but in reality, there was nothing like that. It is better if they had the equipment, they would do detailed investigations I would not have taken such medication for that moment. Another thing is that they would discover this problem early. My ribs were very aching, they told me that I had opacification in my lungs which led to bleeding on the. It is better if they had rapid investigations, it would have helped. But I stayed long at the hospital, I was treated at various hospital and many different diseases, this and that, but then it was just a cancer.

I: And about having referral from one hospital to another, how about those referrals?

R: There is a challenge how to get a referral. I don't know whether it is that the doctor tried to treat and see what he will achieve but as a patient you are suffering. You as a patient you are deteriorating. I prefer if there were enough investigations, many hospitals and specialists, it would be helpful.

I: About the relatives, do they allow you to get the hospital services or are there others who want you to go elsewhere

R: No, nowadays they have understanding.

I: we have talked about your understanding, now your understanding has improved about this problem, right?

R: Currently, I desire to be an advisor to my fellow women, even men. Whenever they see the problem has started they shouldn’t say this stuff has no problem, go and have investigations done. Myself I have played a role to my delay to the hospital, my relatives have played a role, the lack of equipment at the hospital has played a role and economy has also played a role.

I: how about distance?

R: The distance to hospital with investigation has also played a role.

I: Which near hospital had the investigations which at least you got?

R: the hospital where I got investigations is -----, it is where they did all the expertise till I had the operation, at -----.

I: Alright.

R: But in fact, these issues were even supposed to be done even at -----.

I: Earlier on.

R: Early at the moment. Maybe currently they have the investigations, but during that moment it was a challenge. I have been sick till now it is ten years plus how many?

I: From the moment you started to be sick?

R: From the moment my breast was removed it is twelve years.

I: Aaah, when was it removed?

R: In -----.

I: In -----?

R: Yeah, it is a long time, not now. Currently there are specialists at -----. In that there are those people for testing breast cancer, this and that. It was not the case when I started to be sick, that is what played a role. I am talking about the issues of ten years and before.

I: Now, what else would you like to talk about? Thank you, you have talked a lot, I have learnt many things from you. Maybe what else would you like to say which you think is a cause for many women to be late to go to hospital so as to get investigations, especially the imaging investigation?

R: I had suggestions. I would like that when the women go to clinic, they would get a hint about cancer.

I: Which clinic?

R: When she takes her baby to clinic or when pregnant. They should be given a hint about testing for cancer. It should not be until one has got the problem. Because if one gets advice now and then, the mind becomes open and they realize that this can happen, it is not transmissible. That will give one an idea that on seeing these symptoms, like what I have ever heard that it can happen you bleed for a long time, that can be a cancer, calling for further testing. But one can think it is just normal to have periods monthly and even when the days prolong, they just prolong up to more than seven days, they don't realize anything, knowing that it is just normal for women. But in fact there is a problem inside. And when a person stops menstruation, while the time to stop is not yet, they should be alerted, they should not say it is just normal. This is the main thing I advise is to give education.

I: Earlier on.

R: People should be given education on cancer disease. Information on AIDS for example, has been well provided here in Tanzania. Many people know about AIDS even in villages, even the little children, even at school we have taught about AIDS, even me myself I taught.

I: But there is no subject about cancer.

R: But there is no cancer subject. So, my main advice people should be educated, from the moment still they are at school, people should be educated even at the villages, they should summon meetings and bring a specialist to give information about cancer. They should send specialists to go and give information on testing cancer, they are given education, it would save many. Actually, when I was coming at ----- to be admitted there were not so many patients as they are now. When I go to -----, I wonder the crowd of people who have cancer as compared when I stared initially, I say is it communicable again? Has it been infectious or what? And indeed, you find people in bad condition. They have reached the stage in which I was, and others more than that. All this is because of delaying to take action. Cancer is a curable disease. When you delay is when there is a problem like what happens to me. If it were stage one, stage two, at least it is cured. But stage three and four, it is difficulty. You are treated just to reduce suffering but it is not completely. Imagine a person like me, it was in ----- when I had my breast removed, how many years have passed?

I: About twelve.

R: Twelve but look the stage I am in now, in fact I was supposed to say that I have been treated there and cured. I have received chemo for six months I have received bone radiations, I went back for pills, I have come back again for intravenous chemotherapy, but look at my condition, why was I late to start treatment. So, I advise, in fact I even desire even to look for a microphone to announce, ‘colleagues be early to go to investigate cancer’. The sufferings I pass through, I am sick, I have severe suffering, I don't want even my child to pass through such suffering.

I: sorry for that…

R: And if it is inheritable disease, I don't know what should I do. I want to get advice, so and so.

I: So, thank you mum, I am grateful we have talked, I have learnt a lot.

R: Thank you, I don't know if I have answered you correctly.

I: You have indeed answered a lot.

**CASE 2**

I: Y, what year were you born? [00:00:04-0](http://localhost:2300/file=C:/Users/USER/Desktop/EXTRAS/godfrey/180206_1440.mp3time=4000)

R: ----- [00:00:07-1](http://localhost:2300/file=C:/Users/USER/Desktop/EXTRAS/godf)

I: Can you remember the date? [00:00:08-6](%22http)

R: ----- [00:00:16-6](http://localhost:2300/file=C:/Users/USER/Desktop/EXTRAS/godfrey/180206_1440.mp3time=16600)

I: And where do you live? [00:00:19-0](http://localhost:2300/file=C:/Users/USER/Desktop/EXTRAS/godfrey/180206_1440.mp3time=19000)

R: ----- [00:00:19-9](http://localhost:2300/file=C:/Users/USER/Desktop/EXTRAS/godfrey/180206_1440.mp3time=19900)

I: -----? [00:00:21-6](http://localhost:2300/file=C:/Users/USER/De)

R: Yes. [00:00:22-4](http://local)

I: Are you married, and do you live with your husband? [00:00:25-5](http://localhost:2300/file=C:/Users/USER/Desktop/EXTRAS/godfrey/180206_1440.mp3time=25500)

R: Yes [00:00:26-0](http://localhost:2300/file=C:/Users/USER/Desktop/EXTRAS/godfrey/180206_1440.mp3time=26000)

I: How many children do you have? [00:00:29-1](http://localhost:2300/file=C:/Users/USER/Desktop/EXTRAS/godfrey/180206_1440.mp3time=29100)

R: I have three children. [00:00:32-0](http://localhost:2300/file=C:/Users/USER/)

I: Okay. And what is your highest education level? [00:00:37-6](http://localhost:2300/file=C:/Users/USER/Desktop/EXTRAS/godfrey/180206_1440.mp3time=37600)

R: Standard seven. [00:00:37-6](http://localhost:2300/file=C:/Users/USER/Desktop/EXTRAS/godfrey/180206_1440.mp3time=37600)

I: OK… [00:00:38-3](http://localhost:2300/file=C:/Users/USER/Desktop/EXTRAS/godfrey/180206_1440.mp3time=38300)

R: Yes [00:00:38-9](http://localhost:2300/file=C:/Users/USER/Desktop/EXTRAS/godfr)

I: What about your job, are you employed? [00:00:42-5](http://localhost:2300/file=C:/Users/USER/Desktop/EXTRAS/godfrey/180206_1440.mp3time=42500)

R: I am a peasant. [00:00:45-3](http://localhost:2300/file=C:/Users/USER/Desktop/EXTRAS/godfrey/180206_1440.mp3time=45300)

I: Y, can you approximate the distance from your area of residence to the nearest hospital? [00:00:55-9](http://localhost:2300/file=C:/Users/USER/Desktop/EXTRAS/godfrey/180206_1440.mp3time=55900)

R: From? [00:00:56-9](http://localhost:2300/file=C:/Users/USER/Desktop/EXTRAS/godfrey/180206_1440.mp3time=56900)

I: From where you live to the nearest health centre, how far can it be, what is the distance? [00:01:06-2](http://localhost:2300/file=C:/Users/USER/Desktop/EXTRAS/godfrey/180206_1440)

R: Any center? [00:01:07-5](http://localhost:2300/file=C:/Users)

I: Yes, the one that is closest to you. [00:01:09-8](http://localhost:2300/file=C:/Users/USER/Desktop/EXTRAS/godfrey/180206_1440.mp3time=69800)

R: oh, not so far, it is so close such that I can walk on foot to the hospital. [00:01:19-8](http://localhost:2300/file=C:/Users/USER/Desktop/EXTRAS/godfrey/180206_1440.mp3time=79800)

I: Does that mean you do not use any transport? [00:01:23-4](http://localhost:2300/file=C:/Users/USER/Desktop/EX)

R: Not really, I sometimes use a motorbike for one thousand shillings [00:01:29-4](http://localhost:2300/file=C:/Users/USER/Desktop/EXTRAS/godfrey/180206_1440.mp3time=89400)

I: Did you use any means of transport upon coming here to -----? [00:01:37-7](http://localhost:2300/file=C:/Users/USER/Desktop/EXTRAS/godfrey/180206_1440.mp3time=97700)

R: A bus [00:01:38-8](http://localhost:2300/file=C:/Users/USER/Desktop/EXTRAS/godfrey/180206_1440.mp3time=98800)

I: And do you access network where you live? [00:01:42-9](http://localhost:2300/file=C:/Users/USER/Desktop/EXTRAS/godfrey/180206_1440.mp3time=102900)

R: Yes. [00:01:44-2](http://localhost:2300/file=C:/Users/USER/Desktop/EXTRAS/godfrey/180206_1440.mp3time=104200)

I: So you can access internet network [00:01:47-4](http://localhost:2300/file=C:/Users/USER/Desktop/EXTRAS/godfrey/180206_1440.mp3time=107400)

R: Yes [00:01:48-3](http://localhost:2300/file=C:/Users/USER/Desktop/EXTRAS/godfrey/180206_1440.mp3time=108300)

I: Do you use computer or any device that uses network? [00:01:52-7](http://localhost:2300/file=C:/Users/USER/Desktop/EXTRAS/godfrey/180206_1440.mp3time=112700)

R: No, I only have a phone. [00:01:54-6](http://localhost:2300/file=C:/Users/USER/Desktop/EXTRAS/godfrey/180206_1440.mp3t)

I: Does your phone have network access? [00:01:58-8](http://localhos)

R: Yes. [00:01:59-5](http://localhost:2300/file=C:/Users/USER/Desktop/EXTRAS/godfrey/180206_1440.mp3time=119500)

I: Do you get network full time or there are times when network is inaccessible? [00:02:04-4](http://localhost:2300/file=C:/Users/USER/Desktop/EXTRAS/godfrey/180206_1440.mp3time=124400)

R: Aaah! I am not sure if at all there are times it becomes inaccessible but all the times that I try, it is accessible. [00:02:11-7](http://localhost:2300/file=C:/Users/USER/Desktop/EXTRAS/godfrey/180206_1440.mp3time=131700)

I: Alright. So, when did you first realize the symptoms you had? [00:02:19-3](http://localhost:2300/file=C:/Users/USER/Desktop/EXTRAS/godfrey/180206_1440.mp3time=139300)

R: What? [00:02:19-6](http://localhost:2300/file=C:/Users/USER/Desktop/EXTRAS/go)

I: The breast symptoms that made you come here. [00:02:26-5](http://localhost:2300/file=C:/Users/USER/Desktop/EXTRAS/godfrey/180206_1440.mp3time=146500)

R: I have been seeing the breast symptoms since December. In November I only felt the pain in the breast. While I was contemplating on the cause of the symptoms, I had my periods. When the period was over the breast was no longer paining. When it came to December [00:02:55-7](http://localhost:2300/file=C:/Users/USER/Desktop/EXTRAS/godfrey/180206_1440.mp3time=175700)

I: was it last year? [00:02:56-7](http://localhost:2300/file=C:/Users/USER/Desktop/EXTRAS/godfrey/180206_1440.mp3time=176700)

R: Last year. It then came a time when the breast pain began again. At that time I was not in my periods. So I went to ----- hospital. 00:03:06-9

I: so, how long did it take from the time you first noticed the symptoms to when you went to the hospital? [00:03:13-4](http://localhost:2300/file=C:/Users/USER/Desktop/EXTRAS/godfrey/180206_1440.mp3time=193400)

R: I didn’t take long. I was tolerating the pains and but then together with the pain, I felt like there is a swelling inside. There is when I went to ----- hospital. When I arrived there, the doctor examined me and told me that the breast doesn’t have threatening symptoms it is only minor infections. He then asked me of any other problems I had. I told him sometimes I had fever. He then told me to go and test for UTI. I went to test and they noted I had UTI. He gave me some medications, and told me to use the medications for some time. Said the UTI medications will help relieve the breast symptoms too. I used the medications. I got better after using them for some time. As a matter of fact, I am not sure whether it was Panadol or the pain killers or the other medications that worked. The breast was no longer paining, I got better, then I travelled. When I was in ----- the breast pain started again. I tolerated the pains, and thought to myself that I cannot start the treatment processes after all I did not have my health insurance card with me. I tolerated and said to myself that I will go to the hospital once I am in -----. When I arrived in ----- I came directly to the hospital. [00:04:46-6](http://localhost:2300/file=C:/Users/USER/Desktop/EXTRAS/godfrey/180206_1440.mp3time=286600)

I: So, the first person you saw was the doctor or you had previously gone to other means such as traditional doctors.? [00:04:55-5](http://localhost:2300/file=C:/Users/USER/Desktop/EXTRAS/godfrey/180206_1440.mp3time=295500)

R: No. I am not used to going to the traditional doctor. I went straight to ----- hospital. There is where the doctor examined my breast. [00:05:04-2](http://localhost:2300/file=C:/Users/USER/Desktop/EXTRAS/godfrey/180206_1440.mp3time=304200)

I: And what where the first symptom/s that you saw? You have told me of the breast pain. [00:05:08-3](http://localhost:2300/file=C:/Users/USER/Desktop/EXTRAS/godfrey/180206_1440.mp3time=308300)

R: Yes, breast pain. [00:05:09-7](http://localhost:2300/file=C:/Users/USER/Desktop/EXTRAS/godfrey/180206_1440.mp3time=309700)

I: Only that? [00:05:10-5](http://localhost:2300/file=C:/Users/USER/Desktop/EXTRAS/godfrey/180206_1440.mp3time=310500)

R: Eeeh [00:05:11-0](http://localhost:2300/file=C:/Users/USER/Desktop/EXTRAS/go)

I: What about the swelling? [00:05:12-1](%22http)

R: There was pain and a lump followed after all over the breast to the nipple. [00:05:20-7](http://localhost:2300/file=C:/Users/USER/Desktop/EXTRAS/godfrey/180206_1440.mp3time=320700)

I: How long did it take for you to see the doctor since when you first felt the symptoms? [00:05:30-7](http://localhost:2300/file=C:/Users/USER/Desktop/EXTRAS/godfrey/180206_1440.mp3time=330700)

R: All that time since November and December until currently. [00:05:37-7](http://localhost:2300/file=C:/Users/USER/Desktop/EXTRAS/godfrey/180206_1440.mp3time=337700)

I: So, I want to know for instance today you experienced the symptoms and maybe the day you went to see the doctor took maybe one week, two weeks or a month? [00:05:50-2](http://localhost:2300/file=C:/Users/USER/Desktop/EXTRAS/godfrey/180206_1440.mp3time=350200)

R: When I saw the symptoms I thought maybe I am approaching my periods, but then I didn’t see my periods. I then stayed for a week before going to the hospital. After seeing that I was not getting my periods and that there is something like a lump on my breast there is when I decided to go to the hospital. [00:06:08-9](http://localhost:2300/fi)

I: Okay. Since the beginning of symptoms until when you decided to go for ultrasound and mammography, how long did it take until you decided to do such investigations? [00:06:26-9](http://localhost:2300/file=C:/Users/USER/Desktop/EXTRAS/godfrey/180206_1440.mp3time=38)

R: two weeks have passed since I came here. I did the radiographs on -----, and I saw the doctor. He prescribed me some medications which I am still using till date. [00:06:52-8](http://localhost:2300/file=C:/Users/USER/Desktop/EXTRAS/godfrey/180206_1440.mp3time=412800)

I: Okay. Have you ever had any breast illness before? [00:06:59-5](http://localhost:2300/file=C:/Users/USER/Desktop/EXTRAS/godfrey/180206_1440.mp3time=419500)

R: No. [00:07:00-4](http://localhost:2300/file=C:/Users/USER/Desktop/EXTRAS/godfrey/180206_1440.mp3time=420400)

I: And is there anyone in your family who had ever suffered from breast cancer? [00:07:07-4](http://localhost:2300/file=C:/Users/USER/Desktop/EXTRAS/godfrey/180206_1440.mp3time=427400)

R: None. [00:07:12-1](http://localhost:2300/file=C:/Users/USER/Desktop/EXTRAS/godfrey/180206_1440.mp3time=432100)

I: Okay, I would also like to know, because we want to know some of the things that contributed to the delay of you being screened and to be seen by the doctor for this issue. What are the things that have greatly contributed to the delay such that you didn’t get treatment earlier enough? [00:07:38-2](http://localhost:2300/file=C:/Users/USER/Desktop/EXTRAS/godfrey/180206_1440.mp3time=458200)

R: As for me, I haven’t delayed because if I had pain in body, I can never delay to see the doctor. When I saw the problem, I came quickly to the hospital. [00:07:51-9](%22h)

I: Weren’t you scared at first when you saw the symptoms? Weren’t you afraid? Because such things happen to most people and some are scared to know that they are suffering from cancer. Aren’t you scared? [00:08:10-5](http://localhost:2300/file=C)

R: Actually I am not scared because it is good to know the things troubling you so that you may know how to face them. [00:08:24-1](http://localhost:2300/file=C:/Users/USER/Desktop/EXTRAS/godfrey/180206_1440.mp3time=504100)

I: Where did you get the information concerning breast cancer? [00:08:31-2](http://localhost:2300/file=C:/Users/USER/Desktop/EX)

R: I heard that from the radio as well as from somebody who had suffered from breast cancer. [00:08:40-7](http://localhost:2300/file=C:/Users/USER/Desktop/EXTRAS/godfrey/180206_1440.mp3time=520700)

I: What is your understanding on this illness? What do you know about breast cancer.? [00:08:52-7](http://localhost:2300/file=C:/Users/USER/Desktop/EXTRAS/godfrey/18020)

R: I really don’t know much. [00:08:53-6](http://localhost:2300/file=C:/Users/USER/Desktop/EXTRAS/godfrey/180206_1440.mp3time=533600)

I: Do you know how to examine yourself for a lump on the breast? [00:08:59-5](http://localhost:2300/file=C:/Users/USER/Desktop/EXTRAS/godfrey/180206_1440.mp3time=539500)

R: I don’t know how to examine myself. [00:09:03-2](http://localhost:2300/file=C:/Users/USER/Desktop/EXTRAS/godfrey/180206_1440.mp3time=543200)

I: How did you know that you have a lump? [00:09:07-3](http://localhost:2300/fi)

R: I came to know when the pain started. All the other days my breast was okay, but right after the pain started, and I was in so much pain, such that when I touch my breast I feel like this area pains the most. So, when I touched there I felt as if there is something like that… [00:09:34-3](http://localhost:2300/file=C:/Users/USER/Desktop/EXTRAS/godfrey/180206_1440.mp3time=574300)

…. But as for this breast since when I was a girl in my youth, it was different. Even my children never used to breastfeed on it. [00:09:54-9](http://localhost:2300/file=C:/Users/USER/Desktop/EXTRAS/godfrey/180206_1440.mp3t)

I: I see..... [00:09:56-1](http://localhost:2300/file=C:/Users/USER/Desktop/EXTRAS/godfrey/180206_1440.mp3time=596100)

R: Yes. So, my biggest concern is that I used to see this breast bigger than the other which is always small. So, I used to believe that it was small because my children suck it often and more than the other…. [00:10:14-1](http://loc)

I: Do you get to see the health providers often? For instance, if you have a health issue, do you have that habit of seeing the health providers? [00:10:28-3](http://localhost:2300/file=C:/Users/USER/Desktop/EXTRAS/godfrey/180206_1440.mp3time=628300)

R: Yes, if I have a problem, when I fall sick. It depends with what I am suffering from. [00:10:36-2](http://localhost:2300/file=C:/Users/USER/Desktop/EXTRAS/godfrey/180206_1440.mp3time=636200)

I: About the breast issue, have you ever been examined by the doctor before or any hospital health provider? [00:10:43-9](http://localhost:2300/file=C:/Users/USER/Desktop/EXTRAS/godfrey/180206_1440.mp3time=643900)

R: About the breast problem, the examination that was done, was done here not any other place, only at Mloganzila. [00:10:54-1](http://localhost:2300/file=C:/Users/USER/Desktop/EXTRAS/godfrey/180206_1440.mp3time=654100)

I: Okay. How was your level of breast cancer understanding before you had the symptoms? [00:11:06-0](http://localhost:2300/file=C:/Users/USER/Desktop/EXTRAS/go)

R: My understanding on breast cancer, what I used to know about it was that if one gets breast cancer, they die, that is what I know. [00:11:18-0](http://localhost:2300/file=C:/Users/USER/Desktop/EXTRAS/godfrey/180206_1440.mp3time=678000)

I: Is that what you told me that you heard from the radio? [00:11:23-3](http://localhost:2300/file=C:/Users/USER/Desktop/EXTRAS/godfre)

R: Yes, from the radio. I heard it from the radio that when you see a lump on the breast, then there are symptoms of cancer, that’s all. [00:11:42-3](http://localhost:2300/file=C:/Users/USER/Desktop/EXTRAS/godfrey/180206_1440.mp3time=702300)

I: After knowing that you have a lump, were you aware that you needed to see the doctor earlier? [00:11:56-7](http://localhost:2300/file=)

R: Yes. [00:11:57-9](http://localhost:2300/file=C:/Users/USER/Desktop/EXTRAS/godfrey/180206_1440.mp3time=717900)

I: Or is there anyone who told you? [00:11:59-5](http://localhost:2300/file=C:/Users/USER/Desktop/EXTRAS/godfrey/180206_1440.mp3time=719500)

R: No one told me, its I myself. [00:12:03-4](http://localhost:2300/file=C:/User)

I: And you have told me…, have you never been scared of being examined by the doctor and if at all he identified the disease? [00:12:14-8](http://localhost:2300/file=C:/Users/USER/Desktop/EXTRAS/godfrey/180206_1440.mp3time=734800)

R: How can I be scared doctor, because it is the same as a pregnant mother who is scared to go to the clinic. Enh! How are you going to deliver if you are afraid of the doctor? [00:12:26-1](http://localhost:2300/file=C:/Users/USER/Desktop/EXTRAS/godfrey/18020)

I: Alright, are there no issues in the family, let’s say customs and traditions that prohibit people from seeking health care ? [00:12:37-7](http://localhost:2300/file=C:/Users/USER/Desktop/EXTRAS/godfrey/180206_1440.mp3time=757700)

R: I have never seen it on my side. [00:12:48-2](http://localhost:2300/file=C:/Users/USER/Desktop/EXTRAS/godfrey/180206_1440.mp3time=768200)

I: Okay, you told me that you did the tests at -----hospital? [00:12:55-7](http://localhost:2300/file=C:/)

R: Aaah aah I was not tested at -----. He only touched me and thereafter he told me he saw the lump but it should be a normal problem, he prescribed me some medications that I had to take. He said it was an infection. I was given medications for UTI which he said will help me with the breast lump as well. [00:13:19-6](http://localhost:2300/file=C:/Users/USER/Desktop/EXTRAS/godfrey/180206_1440.mp3time=799600)

I: Alright. Thank you for your thoughts and explanations, and for you time to interview with me. [00:13:34-0](http://localhost:2300/file=C:/Users/USER/Desktop/EXTRAS/godfrey/180206_1440.mp3t)

R: Okay. [00:13:47-4](http://localhost:2300/file=C:/Users/USER/Desktop/EXTRAS/godfrey/180206_1440.mp3time=827400)

I: Thank you very much. [00:13:48-8](http://localhost:2300/file=C:/Users/USER/Desktop/EXTRAS/godfrey/180206_1440.mp3time=828800)

R: Alright. [00:13:50-0](http://localhost:2300/file=C:/Users/USER/Desktop/EXTRAS/godfrey/180206_1440.mp3time=830000)

**CASE 3**

I: H, when were you born? In terms of date, month and year?

R: -----.

I: which date?

R: On the -----

I: Ok, and where do you live?

R: At -----

I: Are you married….?

R: Yes I am married.

I: Do you live with your husband?

R: Yes.

I: And how many children do you have?

R: We have three children.

I: And, what highest level of education did you attain?

R: I didn’t reach university

I: Up to what education level did you study?

R: I attained grade three of primary school.

I: so you didn’t complete primary school... And what is your occupation?

R: Yes, I do petty business.

I: Okay, I want to know the distance from where you live to the nearest health center?

R: The nearest centre is -----.

I: -----?

R: yeah.

I: In what distance is it from your home?

R: it is not very far, let say from -----, I don't know it is like from ----- because I don't really know its location, I mean its distance. Let say it is the same as from ----- or a little behind.

I: but from home up to the health centre, do you walk or travel by car?

R: you can walk on foot or by car. It depends on oneself.

I: it is approximately how many kilometers?

R: mhh, now that I cannot truly know…

I: And when you take a car, how much do you pay?

R: five hundred (500TShs).

I: so it is just a short distance.

R: Yeah

I: Ok, H, do you use any network? Do you access internet?

R: No.

I; Do you use computer?

R: No.

I: And how about mobile phone, do you use it?

R: I had a phone, but a day before yesterday when I was doing my business it fell into water. So currently I don't have one.

I: were you able to access internet using your phone?

R: yeah.

I: ----- what are first symptoms that you noticed?

R: Those which I noticed?

I: yeah, those which led you to come to hospital or go to seek for treatment.

R: I found a small swelling like this way, that is why I went to hospital.

I: And how long did it take since you noticed that swelling till you went to the doctor or to a physician?

R: it took some time.

I: Like months or years?

R: No, no, it was months.

I: how many months was it?

R: So far I have forgotten because it was a year before last year when it started. So I don't know how long it took because when it just started, there is my brother-in-law called ----- who works at Amana told me to go with him for a checkup. When we went there they checked me and said, ‘because it is this problem, let us operate her’. They operated me but they didn’t do medical investigation.

I: When was that?

R: The year before last year. It was in December the tenth.

I: so that was the year before last year in December, however when did you notice the symptoms.

R: It was the same year. The year before last year is the very same year when I noticed the symptoms.

**I:** Around which month? I want to know, for example , you had the operation in December, when did you notice that there is a swelling?

R: in fact, that year I was doing like this (palpating her breast), I noticed as if there are particles. I said, ‘I can feel a swelling’. I told my husband, ‘I can feel as if there is a swelling is starting here’. He said, ‘a swelling?’. I told him, yes. Then we decided to wait a bit, we stayed for around four months, the fifth month I had operation. they removed something like this, it was solid. That is how the problem started. I stayed for a while then it has appeared again.

I: okay

R: yeah

I: And who was the first person to see in your efforts to for seek treatment? Have been able to attend these alternative medicines and then came to hospital or where did you start? I want to know also..

R: I have never used herbal medicine.

I: Did you go to any traditional healer?.

R: No, no. I only went for prayers.

I: So you went for prayers. Was it before the operation or after?

R: before and after the operation…...

I: Alright.

R: I have never gone anywhere. It is that, I was told to come to ----- to have the medical investigation so that I can know what is the problem before the operation was done and without it was over but it has recurred again.

I: And what were the initial symptoms that caused you to fear what was the problem in your breast?

R: Symptoms??

I: Yeah.

R: In fact I noticed three particles which started again. Just above the site which was operated.

I: What do you mean when you say particles?

R: indeed they are like let say seeds.

I: seeds??

R: Yeah, three of them, small, then they came to join. They were three later on they became one big swelling...

I: Ok, and who detected them, was it you or?

R: it was myself. When I detected them I told him, and he touched them and said, ‘here are they”.

I: Whom did you show?

R: My husband.

I: So your husband was the first to see them, after you right?

R: yeah

I: And do you know how to examine yourself?

R: Yeah

I: How do you examine a swelling on the breast?

R: so the swellings are here, then I check this way, I touch this way, I apply pressure this way. Only that.(Impression: it was a great way of self-examination).

I: Ok. Excuse me, so you are telling me you had an operation on your breast in December the year before last year, it was done the operation. When did this one start?...

R: from the year before last year, when I went to burry my dad, when was that year? It was last year, when I went to burry my dad, when I was in up country there, I started to feel as if am not okay…

I: in which month was that?

R: I don't know what was the month, I have forgotten.

I: Or are you just telling me the same story as for the current?

R: No, no, I am telling this another story, this new one. This is a new one.

I: And when it started, this new one, you said when was the moth, by approximation? How many months have passed till now or how many weeks?

R: Mmmm, now it is about a year.

I: A year?

R: Yeah.

I: And what has happened in the meanwhile? What kind of treatment have you sought before coming here at hospital?

R: ok, after that, my brother in law told me to go to ----- referral hospital, the doctor advised me, ‘since you have already had the operation, go and take the big medical test so that we know what is the problem’. So that is when he wrote me a referral not come here then I will send the result there.

I: Alright, so for the first one, were you told if it was a cancer or not.

R: No, no. because they didn’t do medical investigation for sure. The investigation I think they didn’t do it, because they just did the operation.

I: Alright, so -----, is there any one in your family who has the history of breast swelling, or who has been told that she has breast cancer?

R: No.

I: Ok, I would like to know something, you told me it is a year since you noticed the symptoms till you have started to receive treatment now, what happened in the between such that you were late? Because I think one year is a long time. Somehow.

R: I used to go to prayers. I went, I prayed, they prayed for me. The condition was not improving. That is why I decided to come to hospital.

I: was there also any fear of knowing what might be the problem in that swelling? Maybe it is just abnormal swelling or a cancer. And is there any stigma if someone is recognized to have the problem? Have all these contributed you to arrive late?

R: No, my husband does not behave like that way.

I: How about relatives?

R: They don’t.

I: How about you? Do you have any fear?

R: maybe.

I: Ok, tell me a little about your understanding on the problem of breast cancer. How is your understanding. What do you know about cancer? If someone tells you about breast cancer, what do you know?

R: The disease is bad indeed, it is terrible.

I: And where do you get these information? The information about the disease of cancer.

R: On the TV everyday they talk. Even that announcement we heard it on the news on TV.

I: Which announcement?

R: about free testing, there was a time when they said that they are testing for free. There was a moment in the meanwhile they were testing freely, right?

I: yeah.

R: Alright, so that also they advertised on TV, it was ITV, TBC I don't know.

I: Do you think another thing which led you to be late, are the expenses likely to be high?

R: No. but since there was a time I was away, I went to my young sister, she was very sick at home. I stayed with her for several months, I attended her till she got better then I came back. So that moment I would have come to seek treatment I would not be just staying without treatment.

I: So there is a lot of issues, right?

R: indeed. Moreover, there was my mother in law was sick, so I was unable to leave her alone. We took her from there, and brought her here to get treatment and then we took her home. I stayed there for a while then i went back to my business. So this also has played a role, I cannot hide information, because I will be hiding at my own risky. So these are what contributed to my delay. Because I had to stay and care for my mother in law for four months till she passed away. the second time I left my young sister was sick., considering that we don't have parents. And nowadays relatives are not like those of the past times, they cannot take care of your family, indeed it is not like the former times. We have been in trouble indeed. In fact, if your father and mother have passed away, you will have formidable trouble. It is better at least one parent is alive, if both are dead, you are in trouble.

I: Do you have the habit of getting heath service/checkup or seeing the doctor frequently? Do you go to meet a doctor now and then,? Is that your practice?

R: if I am sick. At one time a high blood pressure was disturbing me, was gong frequently. Do you see that tall man sitting over there? That is my brother in law, he works at -----. Usually he takes us frequently for checkup but now I am busy due to the two issues I told you. If you don't have your father or your mother in the family, then you ought to have studied indeed. Then you will see its advantage. But if you didn’t even study then it is a problem.

I: However, H, I can see there are many kind of health services, traditional medicine, alternative medicine. if someone is found with a problem of cancer or has a swelling or even a normal disease they go, don't you have the opportunity to meet these people?

R: Actually I have never taken any medication. maybe when I have headache, I take Panadol. But these traditional ones, I have never used them at all. Truly speaking.

I: Why is that?

R: I just don't. what should I say…, maybe I am not used to that, I don't know.

I: Alright.

R: yeah, maybe. I don't know. I have never taken traditional medicine. I have never used. Maybe I start now. In fact, I have never used.

I: Have you been having fear to come to hospital?

R: No.

I: have you ever been investigated by the doctor when you get the swelling?

R: That of the first time?

I: Yeah

R: the doctor at ----- hospital checked me.

I: Ok, so when it was detected you went to the doctor, right?

R: Yeah, the problem is maybe they didn’t do the medical investigation, let say like this one I am doing now.

I: Alright, at least I have got a picture a bit, because one year is a long time.

R: It is long, but it is because I had left I would not stay such long, moreover, there where I was married there is a distance from hospital.

I: You mean where you live there is a long distance?

R: I am referring to the upcountry where I went.

I: But how about here where you live?

R: In -----?

I: yeah

R: here it is just near.

I: how long does it take to arrive at the health center?

R: I told you maybe a half an hour or a quarter an hour. Because it is as far as -----, if you are going this way.

I: And how long has it taken you to do the imaging investigation?

R: I have not taken a long time because the doctor and my brother in law know each other. indeed, I have not taken long time, frankly speaking.

I: Alright, thank you -----. I wanted to know at least that information will give us a certain picture so that we can see where we can improve our breast cancer care provision.

R: alright

I: thank you.

R: Ok, I am grateful.

**CASE 4**

I: So, A… when where you born? [00:00:06-8](http://localhost:2300/file=C:/Users/Judith/Desktop/180427_1147.mp3time=6800)

R: -----. [00:00:08-5](http://localhost:2300/file=C:/Users/Judith/Desktop/180427_1147.mp3time=8500)

I: How about the year you were born? [00:00:09-8](http://localhost:2300/file=C:/Users/Judith/Desktop/180427_1147.mp3time=9800)

R: ----- [00:00:18-7](http://localhost:2300/file=C:/Users/Judith/Desktop/180427_1147.mp3time=18700)

I: You said you live in -----?[00:00:00-0](http://localhost:2300/file=C:/Users/Judith/Desktop/180427_1147.mp3time=0)

R: Yes but I studied at ----- [00:00:25-2](http://localhost:2300/file=C:/Users/Judith/Desktop/180427_1147.mp3time=25200)

I: Are you married? Do you live with your husband? [00:00:29-8](http://localhost:2300/file=C:/Users/Judith/Desktop/180427_1147.mp3time=29800)

R: Yes [00:00:31-3](http://localhost:2300/file=C:/Users/Judith/Desktop/180427_1147.mp3time=31300)

I: What is your highest education level? [00:00:35-7](http://localhost:2300/file=C:/Users/Judith/Desktop/180427_1147.mp3time=35700)

R: I studied up to standard seven [00:00:37-7](http://localhost:2300/file=C:/Users/Judith/Desktop/180427_1147.mp3time=37700)

I: What is your occupation, A? [00:00:43-7](http://localhost:2300/file=C:/Users/Judith/Desktop/180427_1147.mp3time=43700)

R: I am a peasant [00:00:44-4](http://localhost:2300/file=C:/Users/Judith/Desktop/180427_1147.mp3time=44400)

I: Okay... what is the distance from your home place to the nearest health center? I mean the nearest health center closest to you? [00:00:54-8](http://localhost:2300/file=C:/Users/Judith/Desktop/180427_1147.mp3time=54800)

R: it’s so near [00:00:58-7](http://localhost:2300/file=C:/Users/Judith/Desktop/180427_1147.mp3time=58700)

I: Do you walk on foot, or use any kind of transport? [00:01:04-0](http://localhost:2300/file=C:/Users/Judith/Desktop/180427_1147.mp3time=64000)

R: it is on foot. The hospital is close by; it is approximately fifteen minutes from here to there. [00:02:13-7](http://localhost:2300/file=C:/Users/Judith/Desktop/180427_1147.mp3time=133700)

I: Okay. What kind of mobile network do you use specifically? [00:01:15-1](http://localhost:2300/file=C:/Users/Judith/Desktop/180427_1147.mp3time=75100)

R: Vodacom [00:01:15-8](http://localhost:2300/file=C:/Users/Judith/Desktop/180427_1147.mp3time=75800)

I: Do you have access to internet through the phone that you use? [00:01:24-7](http://localhost:2300/file=C:/Users/Judith/Desktop/180427_1147.mp3time=84700)

R: Yes, there is. [00:01:27-3](http://localhost:2300/file=C:/Users/Judith/Desktop/180427_1147.mp3time=87300)

I: do you get health information through the phone you use? [00:01:37-1](http://localhost:2300/file=C:/Users/Judith/Desktop/180427_1147.mp3time=97100)

R: Yes [00:01:36-2](http://localhost:2300/file=C:/Users/Judith/Desktop/180427_1147.mp3time=96200)

I: Okay, how did you know about breast cancer? [00:02:24-8](http://localhost:2300/file=C:/Users/Judith/Desktop/180427_1147.mp3time=144800)

R: there is a certain place that I used to work before… then one day I went to the hospital and asked the nurse that why both of my breasts swell. I began going there when both breasts were swollen without decreasing. I continued going there until one month passed and still they were the same, no changes. Later on, one started to decrease in size but the other one did not. A month after I finished my periods, and on following month there was a lump on the nipples. I stayed for a while then later on I went back to the hospital and told the nurse about my situation and asked her why are they still enlarging?[00:03:55-1](http://localhost:2300/file=C:/Users/Judith/Desktop/180427_1147.mp3time=235100)

I: Then... [00:03:59-5](http://localhost:2300/file=C:/Users/Judith/Desktop/180427_1147.mp3time=239500)

R: she told me that, recently there is a disease, you shouldn’t waste your money, go to -----hospital and for testing... ----- is my homeplace, but I was there on work mission. I went to ----- center, told the health workers that my breast has swollen but it is not increasing. They gave me Panadol. [00:04:38-4](http://localhost:2300/file=C:/Users/Judith/Desktop/180427_1147.mp3time=278400)

I: Ever since you experienced the symptoms until when you went to ----- hospital to see the doctor, how long did it take? [00:04:44-7](http://localhost:2300/file=C:/Users/Judith/Desktop/180427_1147.mp3time=284700)

R: about two months. [00:04:47-9](http://localhost:2300/file=C:/Users/Judith/Desktop/180427_1147.mp3time=287900)

I: the first time that you detected the symptoms, was it by self-examining that you noticed that you had a lump or was it someone who examined you and identified the mass? [00:05:01-8](http://localhost:2300/file=C:/Users/Judith/Desktop/180427_1147.mp3time=301800)

R: I used to tell my partner everyday, that I am sick, and I asked him if it is a boil or not. He said that he cannot know saying… “let me buy for you some medicine that you can take and we observe how it will go”. When he bought the medications, I took them the swelling didn’t increase. I bought them once again but no changes. So, the third time is when the nurse advised me to go to ----- hospital. [00:05:44-8](http://localhost:2300/file=C:/Users/Judith/Desktop/180427_1147.mp3time=344800)

I: then what happened. [00:05:41-5](http://localhost:2300/file=C:/Users/Judith/Desktop/180427_1147.mp3time=341500)

R: when I went there, they gave me some medications to take, and to go back if the lump didn’t disappear. I took the medications until when the dose was over. Then when I went back, the nurses in that hospital told me to go to ----- because there were no any investigations they could do at their hospital. [00:06:22-0](http://localhost:2300/file=C:/Users/Judith/Desktop/180427_1147.mp3time=382000)

I: what test did they do first? [00:06:48-3](http://localhost:2300/file=C:/Users/Judith/Desktop/180427_1147.mp3time=408300)

R: I went to ----- (regional hospital), when I reached, there were no investigations too, so I was told to attend cardiac clinic, since I also have cardiac problems, I went. They told me that I should remove the lump when it is still small. [00:07:12-4](http://localhost:2300/file=C:/Users/Judith/Desktop/180427_1147.mp3time=432400)

I: did the doctor tell you so? [00:07:13-5](http://localhost:2300/file=C:/Users/Judith/Desktop/180427_1147.mp3time=433500)

R: yes, at -----. They told me if it is removed while it is still small there will be no complications but if I delay it will be a cancer. I went home and told my relatives that if I delay it will develop into cancer. My sister, who has studied politics, said no, “you shouldn’t be operated to cut the breast because if its cut it will spread in the whole-body system, let me find some money then you will go for investigations”. After she found some money, I went to ----- where the whitemen were coming to ----- hospital. [00:08:03-6](http://localhost:2300/file=C:/Users/Judith/Desktop/180427_1147.mp3time=483600)

I: were the whitemen doctors? [00:08:07-9](http://localhost:2300/file=C:/Users/Judith/Desktop/180427_1147.mp3time=487900)

R: yes, so, after I went there, they however came for heart problems testing, not for breast cancer screening. One of the doctors told me that the symptoms and signs are of breast cancer. So, because you have come unprepared, then you should go, prepare yourself so that you can go to ----- to be investigated because if you continue like this, the disease will spread. This is still at the first stages and it will heal. He told me there is stage one, two and three, mine was at first stage and it can resolve with treatments. When I went to see my sister I told her everything, she told me to go then on the fourth day she will call me to come to her place. [00:09:32-0](http://localhost:2300/file=C:/Users/Judith/Desktop/180427_1147.mp3time=572000)

I: Do you live with your sister? [00:09:35-0](http://localhost:2300/file=C:/Users/Judith/Desktop/180427_1147.mp3time=575000)

R: No, she works at ----- [00:09:40-9](http://localhost:2300/file=C:/Users/Judith/Desktop/180427_1147.mp3time=580900)

I: So, after you telling her for the second time, did she agree that you should start treatment? [00:09:47-2](http://localhost:2300/file=C:/Users/Judith/Desktop/180427_1147.mp3time=587200)

R: Yes, she looked for money and she gave me about seven hundred thousand (700,000Tshs), then I came to -----. I dropped at ----- center where my host resides and they are the ones who brought me to the hospital. I stayed here up to two months duration. [00:10:15-2](http://localhost:2300/file=C:/Users/Judith/Desktop/180427_1147.mp3time=615200)

I: without being treated? [00:10:18-0](http://localhost:2300/file=C:/Users/Judith/Desktop/180427_1147.mp3time=618000)

R: Yes, I used to come and go. [00:10:22-3](http://localhost:2300/file=C:/Users/Judith/Desktop/180427_1147.mp3time=622300)

I: when you were coming here, did you get any investigations done [00:10:24-1](http://localhost:2300/file=C:/Users/Judith/Desktop/180427_1147.mp3time=624100)

R: I did all investigations, about six investigations. [00:10:28-3](http://localhost:2300/file=C:/Users/Judith/Desktop/180427_1147.mp3time=628300)

I: Did you do any imaging investigations? [00:10:29-9](http://localhost:2300/file=C:/Users/Judith/Desktop/180427_1147.mp3time=629900)

R: I did [00:10:30-4](http://localhost:2300/file=C:/Users/Judith/Desktop/180427_1147.mp3time=630400)

I: how long did it take, since you started experiencing the symptoms to when you got investigations? [00:10:35-5](http://localhost:2300/file=C:/Users/Judith/Desktop/180427_1147.mp3time=635500)

R: I came around November, in the same month I did a chest x ray. [00:10:57-5](http://localhost:2300/file=C:/Users/Judith/Desktop/180427_1147.mp3time=657500)

I: which month did you notice the disease for the first time? [00:11:05-4](http://localhost:2300/file=C:/Users/Judith/Desktop/180427_1147.mp3time=665400)

R: they did a chest x ray and they said that they have not yet come into conclusion, I came back and they took blood for investigations and they said again they have not come to conclusion then lastly, they took a tissue, so I came by and they took a tissue. [00:11:33-4](http://localhost:2300/file=C:/Users/Judith/Desktop/180427_1147.mp3time=693400)

I: where did you do the ultrasound investigation? [00:11:38-7](http://localhost:2300/file=C:/Users/Judith/Desktop/180427_1147.mp3time=698700)

R: I did it at ----- [00:11:42-8](http://localhost:2300/file=C:/Users/Judith/Desktop/180427_1147.mp3time=702800)

I: so, how long did it take since you started being sick? [00:11:47-5](http://localhost:2300/file=C:/Users/Judith/Desktop/180427_1147.mp3time=707500)

R: I completed two months. [00:11:49-4](http://localhost:2300/file=C:/Users/Judith/Desktop/180427_1147.mp3time=709400)

I: when you were still in -----? [00:11:54-4](http://localhost:2300/file=C:/Users/Judith/Desktop/180427_1147.mp3time=714400)

R: yes [00:11:51-7](http://localhost:2300/file=C:/Users/Judith/Desktop/180427_1147.mp3time=711700)

I: what about when you were still at home, how long did it take ever since you experienced the symptoms? [00:11:54-4](http://localhost:2300/file=C:/Users/Judith/Desktop/180427_1147.mp3time=714400)

R: after I came from home, it took about one month and three weeks there is when my sister had all the money needed, then I came to -----. [00:12:25-8](http://localhost:2300/file=C:/Users/Judith/Desktop/180427_1147.mp3time=745800)

I: okay [00:12:36-9](http://localhost:2300/file=C:/Users/Judith/Desktop/180427_1147.mp3time=756900)

R: so after the investigations, I was told to come back on ----- for surgery, do you consent? my relatives asked, I said yes. They asked me do you really agree to this? I said yes. I don’t know what happened after I went, I don’t know what came into my mind, and I had money in my simcard, and I lost my phone, I didn’t know what more I could do. I then thought if I tell her I have lost the phone and that she should send me another amount, it will be some kind of disturbance. [00:13:30-6](http://localhost:2300/file=C:/Users/Judith/Desktop/180427_1147.mp3time=810600)

I: in the course of treatment who did you see first, for example to the traditional doctors first or maybe… [00:13:58-9](http://localhost:2300/file=C:/Users/Judith/Desktop/180427_1147.mp3time=838900)

R: they told me to start with the traditional doctor, but my sister was against that, and she said that this kind of illness cannot be cured by the traditional doctors. They will just trick me and waste my time and money. [00:14:10-0](http://localhost:2300/file=C:/Users/Judith/Desktop/180427_1147.mp3time=850000)

I: Was that after you started treatment at the hospital or? [00:14:12-3](http://localhost:2300/file=C:/Users/Judith/Desktop/180427_1147.mp3time=852300)

R: yes. I had already started treatment but they told me that there are some traditional medications that can cure this kind of illness. But my relatives were strictly against that, they said that the disease have grown the disease was still there. It cannot be cured by traditional medicines. [00:14:38-9](http://localhost:2300/file=C:/Users/Judith/Desktop/180427_1147.mp3time=878900)

I: throughout your life, have you heard anyone in your family who has ever suffered from breast cancer? [00:14:46-4](http://localhost:2300/file=C:/Users/Judith/Desktop/180427_1147.mp3time=886400)

R: no, I have not heard. [00:14:49-1](http://localhost:2300/file=C:/Users/Judith/Desktop/180427_1147.mp3time=889100)

I: what about you, have you ever suffered from any breast disease in the past? [00:14:59-3](http://localhost:2300/file=C:/Users/Judith/Desktop/180427_1147.mp3time=899300)

R: yes, I have ever. [00:15:01-8](http://localhost:2300/file=C:/Users/Judith/Desktop/180427_1147.mp3time=901800)

I: when? Was it before this time? [00:15:04-7](http://localhost:2300/file=C:/Users/Judith/Desktop/180427_1147.mp3time=904700)

R: yes, before I gave birth to my child, I had ever suffered from breast disease. [00:15:09-4](http://localhost:2300/file=C:/Users/Judith/Desktop/180427_1147.mp3time=909400)

I: how bad was it? [00:15:08-0](http://localhost:2300/file=C:/Users/Judith/Desktop/180427_1147.mp3time=908000)

R: it was like they were burnt, then later on they were discharging some watery discharge. It delayed to heal, it took like ten years when I was still a girl, then later on they healed. Then I got married, I gave birth to my first child, and they were healed. [00:15:43-2](http://localhost:2300/file=C:/Users/Judith/Desktop/180427_1147.mp3time=943200)

I: okay. I still want to know the things/factors that predispose our patients to delay seeking hospital treatment as well as delay in getting the investigations done, especially the radiological investigations because it is the highly preferred investigations which will determine the modality of treatment. I want to learn from you, what do you think are the causes to the delay [00:16:08-9](http://localhost:2300/file=C:/Users/Judith/Desktop/180427_1147.mp3time=968900)

R: factors that lead to the delay, for instance for us peasants, it’s the monetary factor. Not only I but also there are others out there. There is somebody who had cancer of the mouth, the other one cancer of the private region, and they are just there, hopelessly waiting for Gods call, waiting for their death. [00:16:38-7](http://localhost:2300/file=C:/Users/Judith/Desktop/180427_1147.mp3time=998700)

I: do you think there are no other factors like being scared of knowing that they have cancer. Do you think that doesn’t contribute to the delay of coming to the hospital? [00:16:52-9](http://localhost:2300/file=C:/Users/Judith/Desktop/180427_1147.mp3time=1012900)

R: no, because as for me I used to see it as the normal disease. I didn’t know it is this big, and can cause death. So, my relatives attend some sessions where they are taught how cancer starts, they are given exercises to examine themselves after they wake up. [00:17:21-4](http://localhost:2300/file=C:/Users/Judith/Desktop/180427_1147.mp3time=1041400)

I: did you also happen to know how to examine yourself? [00:17:31-4](http://localhost:2300/file=C:/Users/Judith/Desktop/180427_1147.mp3time=1051400)

R: no, I came to learn when I already had the disease. I was told that, even in the radios they say about this. When you wake up and examine yourself, once you feel any swelling, you go to the hospital early. They remove it while it is still immature. [00:17:58-9](http://localhost:2300/file=C:/Users/Judith/Desktop/180427_1147.mp3time=1078900)

I: what was your relatives’ perspective on this disease? Do you think by any means they might have contributed to the delay of treatment? [00:18:11-6](http://localhost:2300/file=C:/Users/Judith/Desktop/180427_1147.mp3time=1091600)

R: My relative is educated and is optimistic. [00:18:20-6](http://localhost:2300/file=C:/Users/Judith/Desktop/180427_1147.mp3time=1100600)

I: so, you think she is the one who has helped you much until you came early to the hospital? [00:18:26-6](http://localhost:2300/file=C:/Users/Judith/Desktop/180427_1147.mp3time=1106600)

R: Yes [00:18:28-1](http://localhost:2300/file=C:/Users/Judith/Desktop/180427_1147.mp3time=1108100)

I: But you were still late. [00:18:18-3](http://localhost:2300/file=C:/Users/Judith/Desktop/180427_1147.mp3time=1098300)

R: I have used a total of four months. [00:18:18-3](http://localhost:2300/file=C:/Users/Judith/Desktop/180427_1147.mp3time=1098300)

I: do you have a routine of seeking health services frequently? [00:18:46-3](http://localhost:2300/file=C:/Users/Judith/Desktop/180427_1147.mp3time=1126300)

R: yes [00:18:43-8](http://localhost:2300/file=C:/Users/Judith/Desktop/180427_1147.mp3time=1123800)

I: what do you do? [00:18:51-5](http://localhost:2300/file=C:/Users/Judith/Desktop/180427_1147.mp3time=1131500)

R: what I mean by saying that is, we are all Christians at home, we do not give our first thoughts on being bewitched or something like that. That is why the first time that I was told that I am sick, I was able to believe that. [00:19:08-3](http://localhost:2300/file=C:/Users/Judith/Desktop/180427_1147.mp3time=1148300)

I: did you say there are costs, for example transport to take you to the hospital? Why didn’t you start from ----- in getting your treatment? [00:19:27-7](http://localhost:2300/file=C:/Users/Judith/Desktop/180427_1147.mp3time=1167700)

R: I would have started from -----, but they say that there is some bureaucracy, it takes time. [00:19:36-5](http://localhost:2300/file=C:/Users/Judith/Desktop/180427_1147.mp3time=1176500)

I: bureaucracy in what terms? What do you mean? [00:19:40-0](http://localhost:2300/file=C:/Users/Judith/Desktop/180427_1147.mp3time=1180000)

R: you can stay for over six months or even four months without getting treatment. [00:19:48-6](http://localhost:2300/file=C:/Users/Judith/Desktop/180427_1147.mp3time=1188600)

I: from all the hospitals you have been through, starting from ----- how available were the screening tools? Because I see it could be possible to do the radiological investigation earlier to know whether it is cancer or not cancer. [00:20:07-0](http://localhost:2300/file=C:/Users/Judith/Desktop/180427_1147.mp3time=1207000)

R: At ----- they test using x ray [00:20:07-7](http://localhost:2300/file=C:/Users/Judith/Desktop/180427_1147.mp3time=1207700)

I: Don’t they have ultrasound? [00:20:14-0](http://localhost:2300/file=C:/Users/Judith/Desktop/180427_1147.mp3time=1214000)

R: they don’t, if they do, then maybe it is of recent. [00:20:18-3](http://localhost:2300/file=C:/Users/Judith/Desktop/180427_1147.mp3time=1218300)

I: Okay, thank you so much. Do you have anything to add apart from the reasons you told me of which the first one is about monetary issues, what about knowledge on this disease [00:20:36-7](http://localhost:2300/file=C:/Users/Judith/Desktop/180427_1147.mp3time=1236700)

R: most people lack that knowledge on the disease, most of them will say they are bewitched. And the money they get are taken to the traditional doctors for nothing. Once they come into their senses, the disease is already advanced. [00:20:58-2](http://localhost:2300/file=C:/Users/Judith/Desktop/180427_1147.mp3time=1258200)

I: what about your husband, how did he receive the information concerning the illness? [00:21:05-0](http://localhost:2300/file=C:/Users/Judith/Desktop/180427_1147.mp3time=1265000)

R: he mostly wanted me to go to the traditional doctors, but my relatives did not agree to that. [00:21:13-3](http://localhost:2300/file=C:/Users/Judith/Desktop/180427_1147.mp3time=1273300)

I: what about family duties and responsibilities…you told me that you are a peasant, do you think you have a lot of duties? [00:21:22-5](http://localhost:2300/file=C:/Users/Judith/Desktop/180427_1147.mp3time=1282500)

R: yes, my husband is here with me, but concerning payment issues, my sister is the one responsible for that. [00:21:29-0](http://localhost:2300/file=C:/Users/Judith/Desktop/180427_1147.mp3time=1289000)

I: okay -----... thank you [00:21:32-5](http://localhost:2300/file=C:/Users/Judith/Desktop/180427_1147.mp3time=1292500)

R: Okay, thankyou [00:21:34-9](http://localhost:2300/file=C:/Users/Judith/Desktop/180427_1147.mp3time=1294900)

**CASE 5**

I: What is your name? [00:00:05-1](http://localhost:2300/file=C:/Users/Lenovo%20Core%20i5/Desktop/180510_1203_01.mp3time=5100)

R: My name is ----- ] .[00:00:05-1](http://localhost:2300/file=C:/Users/Lenovo%20Core%20i5/Desktop/180510_1203_01.mp3time=5100)

I: And what’s your birth date? [00:00:08-5](http://localhost:2300/file=C:/Users/Lenovo%20Core%20i5/Desktop/180510_1203_01.mp3time=8500)

R: I was born on -----. [00:00:11-4](http://localhost:2300/file=C:/Users/Lenovo%20Core%20i5/Desktop/180510_1203_01.mp3time=11400)

I: Which year? [00:00:13-6](http://localhost:2300/file=C:/Users/Lenovo%20Core%20i5/Desktop/180510_1203_01.mp3time=13600)

R: -----. [00:00:14-3](http://localhost:2300/file=C:/Users/Lenovo%20Core%20i5/Desktop/180510_1203_01.mp3time=14300)

I: And where do you live -----? [00:00:16-6](http://localhost:2300/file=C:/Users/Lenovo%20Core%20i5/Desktop/180510_1203_01.mp3time=16600)

R: -----. [00:00:20-7](http://localhost:2300/file=C:/Users/Lenovo%20Core%20i5/Desktop/180510_1203_01.mp3time=20700)

I: -----? [00:00:19-3](http://localhost:2300/file=C:/Users/Lenovo%20Core%20i5/Desktop/180510_1203_01.mp3time=19300)

R: Yeah. [00:00:19-3](http://localhost:2300/file=C:/Users/Lenovo%20Core%20i5/Desktop/180510_1203_01.mp3time=19300)

I: And do you have a husband and/or kids maybe? [00:00:23-2](http://localhost:2300/file=C:/Users/Lenovo%20Core%20i5/Desktop/180510_1203_01.mp3time=23200)

R: Yeah, I have three kids. [00:00:24-5](http://localhost:2300/file=C:/Users/Lenovo%20Core%20i5/Desktop/180510_1203_01.mp3time=24500)

I: And a husband? [00:00:28-6](http://localhost:2300/file=C:/Users/Lenovo%20Core%20i5/Desktop/180510_1203_01.mp3time=28600)

R: Yeah. [00:00:28-6](http://localhost:2300/file=C:/Users/Lenovo%20Core%20i5/Desktop/180510_1203_01.mp3time=28600)

I: What is the highest level of education that you have attained? [00:00:29-5](http://localhost:2300/file=C:/Users/Lenovo%20Core%20i5/Desktop/180510_1203_01.mp3time=29500)

R: Only standard seven. [00:00:34-3](http://localhost:2300/file=C:/Users/Lenovo%20Core%20i5/Desktop/180510_1203_01.mp3time=34300)

I: Standard seven? [00:00:32-7](http://localhost:2300/file=C:/Users/Lenovo%20Core%20i5/Desktop/180510_1203_01.mp3time=32700)

R: Yeah. [00:00:32-7](http://localhost:2300/file=C:/Users/Lenovo%20Core%20i5/Desktop/180510_1203_01.mp3time=32700)

I: what is your job/occupation? [00:00:37-5](http://localhost:2300/file=C:/Users/Lenovo%20Core%20i5/Desktop/180510_1203_01.mp3time=37500)

R: I used to work in a hair salon, mmh…. [00:00:42-5](http://localhost:2300/file=C:/Users/Lenovo%20Core%20i5/Desktop/180510_1203_01.mp3time=42500)

I: And now? [00:00:42-5](http://localhost:2300/file=C:/Users/Lenovo%20Core%20i5/Desktop/180510_1203_01.mp3time=42500)

R: For the time being, since these problems started, I have stopped. [00:00:45-5](http://localhost:2300/file=C:/Users/Lenovo%20Core%20i5/Desktop/180510_1203_01.mp3time=45500)

I: When did your problems start? [00:00:51-4](http://localhost:2300/file=C:/Users/Lenovo%20Core%20i5/Desktop/180510_1203_01.mp3time=51400)

R: My problems? [00:00:52-7](http://localhost:2300/file=C:/Users/Lenovo%20Core%20i5/Desktop/180510_1203_01.mp3time=52700)

I: Mmmh. [00:00:52-7](http://localhost:2300/file=C:/Users/Lenovo%20Core%20i5/Desktop/180510_1203_01.mp3time=52700)

R: They started in the last year after I felt down and broke my leg. Now, after my leg was broken, by the time I was staying indoors with the plaster of Paris, I touched here (*showing on the breast*) and noticed that there was like a sort swelling. I told my friends that I had a swelling and they said that it was probably because I felt down. Now as days went on, the swelling was increasing in size, I stayed with it for about a year but I noted that it was still growing, then people told me to go to the hospital, advisors as you know women, we do meet up with friends maybe, they told me “Go to the hospital, go to the hospital” [00:01:39-8](http://localhost:2300/file=C:/Users/Lenovo%20Core%20i5/Desktop/180510_1203_01.mp3time=99800)

I: I see, and what is the distance from where you live to..... [00:01:41-4](http://localhost:2300/file=C:/Users/Lenovo%20Core%20i5/Desktop/180510_1203_01.mp3time=101400)

R: To? [00:01:41-4](http://localhost:2300/file=C:/Users/Lenovo%20Core%20i5/Desktop/180510_1203_01.mp3time=101400)

I: To the hospital, I mean from your residential area to the hospital, what is the distance? Any nearby hospital. [00:01:51-2](http://localhost:2300/file=C:/Users/Lenovo%20Core%20i5/Desktop/180510_1203_01.mp3time=111200)

R: Is not a far distance. [00:01:50-6](http://localhost:2300/file=C:/Users/Lenovo%20Core%20i5/Desktop/180510_1203_01.mp3time=110600)

I: Do you walk or take a bus? [00:01:53-2](http://localhost:2300/file=C:/Users/Lenovo%20Core%20i5/Desktop/180510_1203_01.mp3time=113200)

R: I take a bus. [00:01:53-2](http://localhost:2300/file=C:/Users/Lenovo%20Core%20i5/Desktop/180510_1203_01.mp3time=113200)

I: Do you pay bus fare? [00:01:56-3](http://localhost:2300/file=C:/Users/Lenovo%20Core%20i5/Desktop/180510_1203_01.mp3time=116300)

R: Yeah. [00:01:55-7](http://localhost:2300/file=C:/Users/Lenovo%20Core%20i5/Desktop/180510_1203_01.mp3time=115700)

I: Like how much? [00:01:57-3](http://localhost:2300/file=C:/Users/Lenovo%20Core%20i5/Desktop/180510_1203_01.mp3time=117300)

R: For the normal hospitals or? [00:01:59-8](http://localhost:2300/file=C:/Users/Lenovo%20Core%20i5/Desktop/180510_1203_01.mp3time=119800)

I: Yeah, the one that is nearby and that you can easily reach from your home. [00:02:04-6](http://localhost:2300/file=C:/Users/Lenovo%20Core%20i5/Desktop/180510_1203_01.mp3time=124600)

R: Only three hundred shillings. [00:02:04-6](http://localhost:2300/file=C:/Users/Lenovo%20Core%20i5/Desktop/180510_1203_01.mp3time=124600)

I: Ooh, which hospital is that? [00:02:08-2](http://localhost:2300/file=C:/Users/Lenovo%20Core%20i5/Desktop/180510_1203_01.mp3time=128200)

R: ----- [00:02:08-2](http://localhost:2300/file=C:/Users/Lenovo%20Core%20i5/Desktop/180510_1203_01.mp3time=128200)

I: Ahaa, alright…. and do you use a mobile phone or a computer? [00:02:17-7](http://localhost:2300/file=C:/Users/Lenovo%20Core%20i5/Desktop/180510_1203_01.mp3time=137700)

R: Mobile phone. [00:02:21-1](http://localhost:2300/file=C:/Users/Lenovo%20Core%20i5/Desktop/180510_1203_01.mp3time=141100)

I: Mobile phone. I want to know from where mostly did you get the information about breast cancer. [00:02:23-8](http://localhost:2300/file=C:/Users/Lenovo%20Core%20i5/Desktop/180510_1203_01.mp3time=143800)

R: Mm, I just noticed it myself, I didn’t even know. [00:02:26-7](http://localhost:2300/file=C:/Users/Lenovo%20Core%20i5/Desktop/180510_1203_01.mp3time=146700)

I: Haven’t you ever heard, maybe from the radio or from the mobile phone applications? [00:02:30-7](http://localhost:2300/file=C:/Users/Lenovo%20Core%20i5/Desktop/180510_1203_01.mp3time=150700)

R: I have been hearing, I have heard from the radio, TV sessions, mm. [00:02:35-5](http://localhost:2300/file=C:/Users/Lenovo%20Core%20i5/Desktop/180510_1203_01.mp3time=155500)

I: What do you know about breast cancer? [00:02:43-0](http://localhost:2300/file=C:/Users/Lenovo%20Core%20i5/Desktop/180510_1203_01.mp3time=163000)

R: Perhaps I have not understood you, explain to me openly. [00:02:43-6](http://localhost:2300/file=C:/Users/Lenovo%20Core%20i5/Desktop/180510_1203_01.mp3time=163600)

I: I mean, you said you got information maybe the radio or TV. [00:02:46-6](http://localhost:2300/file=C:/Users/Lenovo%20Core%20i5/Desktop/180510_1203_01.mp3time=166600)

R: Mm. [00:02:46-6](http://localhost:2300/file=C:/Users/Lenovo%20Core%20i5/Desktop/180510_1203_01.mp3time=166600)

I: Now I would like to know what do you know about this problem of breast cancer.

[00:02:56-9](http://localhost:2300/file=C:/Users/Lenovo%20Core%20i5/Desktop/180510_1203_01.mp3time=176900)

R: I know, the cancer is like a swelling, but most of the people believe that it is just a normal swelling, but currently most of the swellings turn into cancer.[00:03:08-5](http://localhost:2300/file=C:/Users/Lenovo%20Core%20i5/Desktop/180510_1203_01.mp3time=188500)

I: And what first symptom did you see to say that you had a problem in your breast? [00:03:13-3](http://localhost:2300/file=C:/Users/Lenovo%20Core%20i5/Desktop/180510_1203_01.mp3time=193300)

R: It was swelling. [00:03:13-3](http://localhost:2300/file=C:/Users/Lenovo%20Core%20i5/Desktop/180510_1203_01.mp3time=193300)

I: Swelling? [00:03:13-3](http://localhost:2300/file=C:/Users/Lenovo%20Core%20i5/Desktop/180510_1203_01.mp3time=193300)

R: Mm. [00:03:13-3](http://localhost:2300/file=C:/Users/Lenovo%20Core%20i5/Desktop/180510_1203_01.mp3time=193300)

I: Did you discover it by yourself or somebody else did? [00:03:16-6](http://localhost:2300/file=C:/Users/Lenovo%20Core%20i5/Desktop/180510_1203_01.mp3time=196600)

R: No, it was discovered in the hospital. I considered it as a normal swelling, I went to the hospital and it was removed surgically; I mean I showed a doctor, at Temeke hospital. The doctor told me that it is a swelling that needed to be operated so that it can be taken for further investigations. [00:03:39-5](http://localhost:2300/file=C:/Users/Lenovo%20Core%20i5/Desktop/180510_1203_01.mp3time=219500)

I: Ahaa, and before seeing the doctor, how long did you take since you noticed the symptoms until when you saw the doctor? [00:03:45-5](http://localhost:2300/file=C:/Users/Lenovo%20Core%20i5/Desktop/180510_1203_01.mp3time=225500)

R: It took a day. [00:03:45-5](http://localhost:2300/file=C:/Users/Lenovo%20Core%20i5/Desktop/180510_1203_01.mp3time=225500)

I: Mm. [00:03:47-0](http://localhost:2300/file=C:/Users/Lenovo%20Core%20i5/Desktop/180510_1203_01.mp3time=227000)

R: As I told you, it started slowly, so as it grew up I was getting worried. [00:03:52-3](http://localhost:2300/file=C:/Users/Lenovo%20Core%20i5/Desktop/180510_1203_01.mp3time=232300)

I: So how long did you take until you went to the hospital from the time it started as a small swelling, did you take a year maybe..? [00:03:58-3](http://localhost:2300/file=C:/Users/Lenovo%20Core%20i5/Desktop/180510_1203_01.mp3time=238300)

R: So, I thought I had fallen on that part, it reached a year. [00:04:01-2](http://localhost:2300/file=C:/Users/Lenovo%20Core%20i5/Desktop/180510_1203_01.mp3time=241200)

I: It took a year? [00:04:01-5](http://localhost:2300/file=C:/Users/Lenovo%20Core%20i5/Desktop/180510_1203_01.mp3time=241500)

R: Mm (head nodding). [00:04:02-8](http://localhost:2300/file=C:/Users/Lenovo%20Core%20i5/Desktop/180510_1203_01.mp3time=242800)

I: Okay, and you said that you noticed it by yourself, do you know how to examine yourself to know if you have a swelling in the breasts? [00:04:11-9](http://localhost:2300/file=C:/Users/Lenovo%20Core%20i5/Desktop/180510_1203_01.mp3time=251900)

R: Yeah, I know. [00:04:14-2](http://localhost:2300/file=C:/Users/Lenovo%20Core%20i5/Desktop/180510_1203_01.mp3time=254200)

I: Where did you know from? [00:04:15-7](http://localhost:2300/file=C:/Users/Lenovo%20Core%20i5/Desktop/180510_1203_01.mp3time=255700)

R: I mean, it is like that, we are being instructed in the hospital to….when we wake up every morning, we should lie on the back, and search by using your fingers. [00:04:31-1](http://localhost:2300/file=C:/Users/Lenovo%20Core%20i5/Desktop/180510_1203_01.mp3time=271100)

I: Alright, and what made you late to go see the doctor early after you noticed it, why maybe? [00:04:36-4](http://localhost:2300/file=C:/Users/Lenovo%20Core%20i5/Desktop/180510_1203_01.mp3time=276400)

R: I assumed that it was due to the sliding and falling, because I felt on that side, so straight away I thought maybe it was just that pain of falling. [00:04:46-4](http://localhost:2300/file=C:/Users/Lenovo%20Core%20i5/Desktop/180510_1203_01.mp3time=286400)

I: Pain in the breast? [00:04:46-4](http://localhost:2300/file=C:/Users/Lenovo%20Core%20i5/Desktop/180510_1203_01.mp3time=286400)

R: From falling. [00:04:48-6](http://localhost:2300/file=C:/Users/Lenovo%20Core%20i5/Desktop/180510_1203_01.mp3time=288600)

I: From falling. And when it started, despite knowing that it was just the pain, did you get any treatment before going to the doctor? Maybe from the local doctors? [00:05:06-4](http://localhost:2300/file=C:/Users/Lenovo%20Core%20i5/Desktop/180510_1203_01.mp3time=306400)

R: No. [00:05:05-2](http://localhost:2300/file=C:/Users/Lenovo%20Core%20i5/Desktop/180510_1203_01.mp3time=305200)

I: Okay...... [00:05:07-1](http://localhost:2300/file=C:/Users/Lenovo%20Core%20i5/Desktop/180510_1203_01.mp3time=307100)

R: Mm, I did not go anywhere, I just assumed that it’s because I have fallen down and it is going to disappear. [00:05:11-8](http://localhost:2300/file=C:/Users/Lenovo%20Core%20i5/Desktop/180510_1203_01.mp3time=311800)

I: Mm, whom did you inform about that problem at the beginning? [00:05:15-1](http://localhost:2300/file=C:/Users/Lenovo%20Core%20i5/Desktop/180510_1203_01.mp3time=315100)

R: My friend. [00:05:18-3](http://localhost:2300/file=C:/Users/Lenovo%20Core%20i5/Desktop/180510_1203_01.mp3time=318300)

I: You told me that you have a husband, did you tell him? [00:05:21-9](http://localhost:2300/file=C:/Users/Lenovo%20Core%20i5/Desktop/180510_1203_01.mp3time=321900)

R: Mm? [00:05:21-1](http://localhost:2300/file=C:/Users/Lenovo%20Core%20i5/Desktop/180510_1203_01.mp3time=321100)

I: So your husband knows? [00:05:24-1](http://localhost:2300/file=C:/Users/Lenovo%20Core%20i5/Desktop/180510_1203_01.mp3time=324100)

R: He knew, yeah, but as you know you men, you always take things easy from us (women)…. [00:05:30-3](http://localhost:2300/file=C:/Users/Lenovo%20Core%20i5/Desktop/180510_1203_01.mp3time=330300)

I: How did he consider your problem when you told him that you have a swelling? [00:05:32-6](http://localhost:2300/file=C:/Users/Lenovo%20Core%20i5/Desktop/180510_1203_01.mp3time=332600)

R: At first, he just considered it as a normal thing, mm. [00:05:36-9](http://localhost:2300/file=C:/Users/Lenovo%20Core%20i5/Desktop/180510_1203_01.mp3time=336900)

I: Why do you say that he considered it as a normal thing? What did he say? [00:05:36-9](http://localhost:2300/file=C:/Users/Lenovo%20Core%20i5/Desktop/180510_1203_01.mp3time=336900)

R: Aaah, I told him that since I had a leg accident, it has been like this; he said ooh, maybe it could be because of that because I felt on a bad place. Yeah. [00:05:53-1](http://localhost:2300/file=C:/Users/Lenovo%20Core%20i5/Desktop/180510_1203_01.mp3time=353100)

I: Alright, and when you went to the hospital, did they do an imaging investigation? For example ultrasound. You told me you went and the doctor told me that you were supposed to be operated. [00:06:07-9](http://localhost:2300/file=C:/Users/Lenovo%20Core%20i5/Desktop/180510_1203_01.mp3time=367900)

R: Mm. yes [00:06:07-9](http://localhost:2300/file=C:/Users/Lenovo%20Core%20i5/Desktop/180510_1203_01.mp3time=367900)

I: Did you do any investigations before the operation? [00:06:13-0](http://localhost:2300/file=C:/Users/Lenovo%20Core%20i5/Desktop/180510_1203_01.mp3time=373000)

R: Aaah, I did… I was operated, I did not do any investigations apart from…. aaah, they took blood samples, when they took blood sample the results were normal. [00:06:21-4](http://localhost:2300/file=C:/Users/Lenovo%20Core%20i5/Desktop/180510_1203_01.mp3time=381400)

I: How about ultrasound? [00:06:24-9](http://localhost:2300/file=C:/Users/Lenovo%20Core%20i5/Desktop/180510_1203_01.mp3time=384900)

R: Ultrasound, after I got the problem.[00:06:27-5](http://localhost:2300/file=C:/Users/Lenovo%20Core%20i5/Desktop/180510_1203_01.mp3time=387500)

I: You did? [00:06:27-5](http://localhost:2300/file=C:/Users/Lenovo%20Core%20i5/Desktop/180510_1203_01.mp3time=387500)

R: Yeah. [00:06:27-5](http://localhost:2300/file=C:/Users/Lenovo%20Core%20i5/Desktop/180510_1203_01.mp3time=387500)

I: How long did it take after you got the problem to when you did that investigation? [00:06:31-0](http://localhost:2300/file=C:/Users/Lenovo%20Core%20i5/Desktop/180510_1203_01.mp3time=391000)

R: I did the investigation when I came to start…. So that I could begin treatment. [00:06:35-6](http://localhost:2300/file=C:/Users/Lenovo%20Core%20i5/Desktop/180510_1203_01.mp3time=395600)

I: Which was after a year now? [00:06:37-8](http://localhost:2300/file=C:/Users/Lenovo%20Core%20i5/Desktop/180510_1203_01.mp3time=397800)

R: Enhe. [00:06:37-8](http://localhost:2300/file=C:/Users/Lenovo%20Core%20i5/Desktop/180510_1203_01.mp3time=397800)

I: Alright, and have you ever used traditional medicines maybe for this problem? [00:06:48-4](http://localhost:2300/file=C:/Users/Lenovo%20Core%20i5/Desktop/180510_1203_01.mp3time=408400)

R: Mm. [00:06:47-1](http://localhost:2300/file=C:/Users/Lenovo%20Core%20i5/Desktop/180510_1203_01.mp3time=407100)

I: Didn’t you go for prayer services? I mean which other services apart from the hospital services did you use? [00:06:54-8](http://localhost:2300/file=C:/Users/Lenovo%20Core%20i5/Desktop/180510_1203_01.mp3time=414800)

R: I have never went for prayers, to be honest. [00:06:58-5](http://localhost:2300/file=C:/Users/Lenovo%20Core%20i5/Desktop/180510_1203_01.mp3time=418500)

I: Which other alternative treatment did you use? [00:06:58-5](http://localhost:2300/file=C:/Users/Lenovo%20Core%20i5/Desktop/180510_1203_01.mp3time=418500)

R: No any. [00:06:58-5](http://localhost:2300/file=C:/Users/Lenovo%20Core%20i5/Desktop/180510_1203_01.mp3time=418500)

I: In your family…, is/was there any relative who has/had the problem of cancer? [00:07:26-3](http://localhost:2300/file=C:/Users/Lenovo%20Core%20i5/Desktop/180510_1203_01.mp3time=446300)

R: Aaa no. [00:07:25-0](http://localhost:2300/file=C:/Users/Lenovo%20Core%20i5/Desktop/180510_1203_01.mp3time=445000)

I: No? and have you never heard maybe of anyone in your family with breast cancer? [00:07:31-6](http://localhost:2300/file=C:/Users/Lenovo%20Core%20i5/Desktop/180510_1203_01.mp3time=451600)

R: You know, old people used to hide such a problem because we were young, and you see a person having such a problem hiding and all that.[00:07:40-6](http://localhost:2300/file=C:/Users/Lenovo%20Core%20i5/Desktop/180510_1203_01.mp3time=460600)

I: Mmmh, and have you ever had a problem with the breast before, or is this the first time? [00:07:46-9](http://localhost:2300/file=C:/Users/Lenovo%20Core%20i5/Desktop/180510_1203_01.mp3time=466900)

R: This is the first time. [00:07:50-9](http://localhost:2300/file=C:/Users/Lenovo%20Core%20i5/Desktop/180510_1203_01.mp3time=470900)

I: And, by the time it discovered to be cancer were you told in which stage was breast cancer? [00:08:03-9](http://localhost:2300/file=C:/Users/Lenovo%20Core%20i5/Desktop/180510_1203_01.mp3time=483900)

R: Mmmh, they just told me my stage is good, mm, it had not gone very far.[00:08:09-2](http://localhost:2300/file=C:/Users/Lenovo%20Core%20i5/Desktop/180510_1203_01.mp3time=489200)

I: Alright, and the other thing maybe I would like to know the reasons which mainly made you delay to come for treatment of your disease. Because as you know this is a disease which needs early treatment so as to have good outcomes. Do you personally think that you delayed to go to the hospital? [00:08:33-9](http://localhost:2300/file=C:/Users/Lenovo%20Core%20i5/Desktop/180510_1203_01.mp3time=513900)

R: yes I was late to go…. [00:08:37-0](http://localhost:2300/file=C:/Users/Lenovo%20Core%20i5/Desktop/180510_1203_01.mp3time=517000)

I: And what do you think was the main reason that made you delay? [00:08:43-3](http://localhost:2300/file=C:/Users/Lenovo%20Core%20i5/Desktop/180510_1203_01.mp3time=523300)

R: I had already told you the reason doctor, i thought it was because I felt down. [00:08:46-3](http://localhost:2300/file=C:/Users/Lenovo%20Core%20i5/Desktop/180510_1203_01.mp3time=526300)

I: And didn’t you even ask any person at the beginning that the swelling was growing? [00:08:57-6](http://localhost:2300/file=C:/Users/Lenovo%20Core%20i5/Desktop/180510_1203_01.mp3time=537600)

R: Mm [00:08:57-6](http://localhost:2300/file=C:/Users/Lenovo%20Core%20i5/Desktop/180510_1203_01.mp3time=537600)

I: No? [00:08:57-6](http://localhost:2300/file=C:/Users/Lenovo%20Core%20i5/Desktop/180510_1203_01.mp3time=537600)

R: I just used to show my close friends, the swelling and all that, they are the ones who advised me that we should observe it first. [00:09:07-0](http://localhost:2300/file=C:/Users/Lenovo%20Core%20i5/Desktop/180510_1203_01.mp3time=547000)

I: So they told you to observe first ..... [00:09:08-6](http://localhost:2300/file=C:/Users/Lenovo%20Core%20i5/Desktop/180510_1203_01.mp3time=548600)

R: Yes... If it was going to persist, because we all knew that perhaps it was due to the falling. [00:09:15-9](http://localhost:2300/file=C:/Users/Lenovo%20Core%20i5/Desktop/180510_1203_01.mp3time=555900)

I: Was the swelling increasing? [00:09:16-3](http://localhost:2300/file=C:/Users/Lenovo%20Core%20i5/Desktop/180510_1203_01.mp3time=556300)

R: At times it was decreasing, at times it was increasing, so it was confusing me, so I believed that it was going to disappear. [00:09:27-0](http://localhost:2300/file=C:/Users/Lenovo%20Core%20i5/Desktop/180510_1203_01.mp3time=567000)

I: And during all that time, weren’t you in any treatment? [00:09:27-0](http://localhost:2300/file=C:/Users/Lenovo%20Core%20i5/Desktop/180510_1203_01.mp3time=567000)

R: Mm. [00:09:32-6](http://localhost:2300/file=C:/Users/Lenovo%20Core%20i5/Desktop/180510_1203_01.mp3time=572600)

I: you maybe didn’t consider it as a big problem? [00:09:31-1](http://localhost:2300/file=C:/Users/Lenovo%20Core%20i5/Desktop/180510_1203_01.mp3time=571100)

R: Yeah. [00:09:35-2](http://localhost:2300/file=C:/Users/Lenovo%20Core%20i5/Desktop/180510_1203_01.mp3time=575200)

I: And maybe were your perception of treatment expenses made you not to come…? [00:09:39-4](http://localhost:2300/file=C:/Users/Lenovo%20Core%20i5/Desktop/180510_1203_01.mp3time=579400)

R: No. [00:09:43-3](http://localhost:2300/file=C:/Users/Lenovo%20Core%20i5/Desktop/180510_1203_01.mp3time=583300)

I: Do you have health insurance? [00:09:41-9](http://localhost:2300/file=C:/Users/Lenovo%20Core%20i5/Desktop/180510_1203_01.mp3time=581900)

R: Yes I don’t have insurance. [00:09:42-0](http://localhost:2300/file=C:/Users/Lenovo%20Core%20i5/Desktop/180510_1203_01.mp3time=582000)

I: So you pay cash, don’t you think that could also be a reason that made you delay to go for treatment? [00:09:53-4](http://localhost:2300/file=C:/Users/Lenovo%20Core%20i5/Desktop/180510_1203_01.mp3time=593400)

R: That could also be a reason, say for we poor people, due to the problem of expenses; medications and its services are expensive. [00:10:07-4](http://localhost:2300/file=C:/Users/Lenovo%20Core%20i5/Desktop/180510_1203_01.mp3time=607400)

I: Did people you saw at the beginning tell you that the treatment is expensive or? [00:10:14-4](http://localhost:2300/file=C:/Users/Lenovo%20Core%20i5/Desktop/180510_1203_01.mp3time=614400)

R: Aaah, that’s what we hear, you know we mislead each other in the streets. [00:10:18-4](http://localhost:2300/file=C:/Users/Lenovo%20Core%20i5/Desktop/180510_1203_01.mp3time=618400)

I: What misleading things did people say? For example which ones that you heard which made you not to come early… [00:10:26-1](http://localhost:2300/file=C:/Users/Lenovo%20Core%20i5/Desktop/180510_1203_01.mp3time=626100)

R: Like someone may tell you that, those are rich people’s diseases, poor people like us will just suffer. [00:10:38-8](http://localhost:2300/file=C:/Users/Lenovo%20Core%20i5/Desktop/180510_1203_01.mp3time=638800)

I: okay…..? [00:10:37-4](http://localhost:2300/file=C:/Users/Lenovo%20Core%20i5/Desktop/180510_1203_01.mp3time=637400)

R: yeah, I however did not stay with it because of the expenses, the reason is like how I told you and If I knew earlier I wouldn’t have wanted to stay all those days. Nobody likes to kill herself. [00:10:55-4](http://localhost:2300/file=C:/Users/Lenovo%20Core%20i5/Desktop/180510_1203_01.mp3time=655400)

I: Alright… After knowing that you have this problem, before starting treatment, did you get any fear that made you not to go? [00:11:09-1](http://localhost:2300/file=C:/Users/Lenovo%20Core%20i5/Desktop/180510_1203_01.mp3time=669100)

R: Ah, at the beginning I was afraid. [00:11:14-9](http://localhost:2300/file=C:/Users/Lenovo%20Core%20i5/Desktop/180510_1203_01.mp3time=674900)

I: And what about the health system itself in general, maybe availability of services and referral …. you know that not all hospitals offer definitive treatment, mainly big hospitals such as ----- or -----

How was it for you until reaching to these hospitals? [00:11:27-0](http://localhost:2300/file=C:/Users/Lenovo%20Core%20i5/Desktop/180510_1203_01.mp3time=687000)

R: Reaching to these hospitals? [00:11:33-8](http://localhost:2300/file=C:/Users/Lenovo%20Core%20i5/Desktop/180510_1203_01.mp3time=693800)

I: Yes Getting a referrals maybe… reaching to ----- for example. [00:11:37-6](http://localhost:2300/file=C:/Users/Lenovo%20Core%20i5/Desktop/180510_1203_01.mp3time=697600)

R: I told you that I went to Temeke, when I went there and after I it was discovered, the doctor told me to go and get money for taking off the breast. [00:11:49-2](http://localhost:2300/file=C:/Users/Lenovo%20Core%20i5/Desktop/180510_1203_01.mp3time=709200)

I: So you underwent mastectomy at -----? [00:11:53-0](http://localhost:2300/file=C:/Users/Lenovo%20Core%20i5/Desktop/180510_1203_01.mp3time=713000)

R: Yes it was done there but now that doctor told me to bring a lot of money. [00:11:54-3](http://localhost:2300/file=C:/Users/Lenovo%20Core%20i5/Desktop/180510_1203_01.mp3time=714300)

I: At Temeke? [00:11:57-5](http://localhost:2300/file=C:/Users/Lenovo%20Core%20i5/Desktop/180510_1203_01.mp3time=717500)

R: Yeah. [00:11:55-9](http://localhost:2300/file=C:/Users/Lenovo%20Core%20i5/Desktop/180510_1203_01.mp3time=715900)

I: Ahaa, [00:11:59-2](http://localhost:2300/file=C:/Users/Lenovo%20Core%20i5/Desktop/180510_1203_01.mp3time=719200)

R: So it was so expensive for me, a lot of money. I had to…I also did not have the idea of coming to ----- I just thought of looking for the money and going to -----. But as I kept staying…. you know getting money is difficult…., so as I kept looking for money, one of my relative…., I mean my friend whose friend had a similar problem as mine, advised, “Why are you stressed?” she told her that, “I am stressed, I have my relative ….” Because that friend of mine is like my relative, “I am stressed, my relative is sick, she is suffering from this and that, but we have been told to have this amount of money” [00:12:44-6](http://localhost:2300/file=C:/Users/Lenovo%20Core%20i5/Desktop/180510_1203_01.mp3time=764600)

I: What amount were you told? [00:12:48-1](http://localhost:2300/file=C:/Users/Lenovo%20Core%20i5/Desktop/180510_1203_01.mp3time=768100)

R: (laughs) that is… because if we say, doctors will see that we are complaining of high expenses or we speak bad about them, I do not like that. [00:12:56-1](http://localhost:2300/file=C:/Users/Lenovo%20Core%20i5/Desktop/180510_1203_01.mp3time=776100)

I: No, we want to know so that we can see where the problem is and how to solve it, and as I told you we just want to get that information but we do not want to know the person. [00:13:09-8](http://localhost:2300/file=C:/Users/Lenovo%20Core%20i5/Desktop/180510_1203_01.mp3time=789800)

R: So will you not mention even the information that am giving? [00:13:11-5](http://localhost:2300/file=C:/Users/Lenovo%20Core%20i5/Desktop/180510_1203_01.mp3time=791500)

I: No, I will not mention personal information [00:13:15-9](http://localhost:2300/file=C:/Users/Lenovo%20Core%20i5/Desktop/180510_1203_01.mp3time=795900)

R: (laughs) [00:13:16-0](http://localhost:2300/file=C:/Users/Lenovo%20Core%20i5/Desktop/180510_1203_01.mp3time=796000)

I: How was the cost at -----? [00:13:20-3](http://localhost:2300/file=C:/Users/Lenovo%20Core%20i5/Desktop/180510_1203_01.mp3time=800300)

R: Here at ----- the cost is not high, they are just normal, because of technology, aids, that is it. [00:13:28-9](http://localhost:2300/file=C:/Users/Lenovo%20Core%20i5/Desktop/180510_1203_01.mp3time=808900)

I: Why don’t you use insurance services while it may be helpful, why don’t you use it?

[00:13:35-6](http://localhost:2300/file=C:/Users/Lenovo%20Core%20i5/Desktop/180510_1203_01.mp3time=815600)

R: Me? Because of my income, I had gone to look for health insurance card when I already had the problem, so I was supposed to pay for the higher one, the one costing a Million Tanzanian shilings. So by the time am processing it is when that sister told me that I will delay if I have to follow all the processes, it’s just better to go. [00:13:58-0](http://localhost:2300/file=C:/Users/Lenovo%20Core%20i5/Desktop/180510_1203_01.mp3time=838000)

I: Is that another sister your relative or? [00:13:58-0](http://localhost:2300/file=C:/Users/Lenovo%20Core%20i5/Desktop/180510_1203_01.mp3time=838000)

R: Yeah. [00:13:58-0](http://localhost:2300/file=C:/Users/Lenovo%20Core%20i5/Desktop/180510_1203_01.mp3time=838000)

I: Did you inform her at the beginning when this problem started? [00:14:02-1](http://localhost:2300/file=C:/Users/Lenovo%20Core%20i5/Desktop/180510_1203_01.mp3time=842100)

R: My friend is the one who was telling the other sister who had a similar problem like mine, she told her “*I have my relative having this and this”*, she is the one who told me to go directly to -----. [00:14:12-9](http://localhost:2300/file=C:/Users/Lenovo%20Core%20i5/Desktop/180510_1203_01.mp3time=852900)

She is the one who suffered. [00:14:16-0](http://localhost:2300/file=C:/Users/Lenovo%20Core%20i5/Desktop/180510_1203_01.mp3time=856000)

I: Okay. [00:14:18-7](http://localhost:2300/file=C:/Users/Lenovo%20Core%20i5/Desktop/180510_1203_01.mp3time=858700)

R: My friend’s friend, her breast was already removed, so she is the one who said that we should just go to ----- directly, follow some process and it will be cut. [00:14:28-3](http://localhost:2300/file=C:/Users/Lenovo%20Core%20i5/Desktop/180510_1203_01.mp3time=868300)

I: Please tell me your experience after meeting up with a person who had a problem like yours and was treated, do you feel like it changed your thoughts? [00:14:41-2](http://localhost:2300/file=C:/Users/Lenovo%20Core%20i5/Desktop/180510_1203_01.mp3time=881200)

R: So much. [00:14:41-2](http://localhost:2300/file=C:/Users/Lenovo%20Core%20i5/Desktop/180510_1203_01.mp3time=881200)

I: How? [00:14:47-0](http://localhost:2300/file=C:/Users/Lenovo%20Core%20i5/Desktop/180510_1203_01.mp3time=887000)

R: After seeing her, that she had the same problem as mine, she advised me…. and she is on medications. She told me that this disease is now treatable because there are medications, “it is the same to me, my breast was cut and I am using medications, and am okay, I do my activities as usual, so do not have any worries”….[00:15:04-0](http://localhost:2300/file=C:/Users/Lenovo%20Core%20i5/Desktop/180510_1203_01.mp3time=904000)

I: Do you think the information that you got from the patient herself compared to that you got from relatives, doctors, or people who have never suffered from that problem had any influence? [00:15:15-6](http://localhost:2300/file=C:/Users/Lenovo%20Core%20i5/Desktop/180510_1203_01.mp3time=915600)

R: Yeah, that has changed me a lot, it gave me light, am really thankful, she helped me to some extents. [00:15:25-9](http://localhost:2300/file=C:/Users/Lenovo%20Core%20i5/Desktop/180510_1203_01.mp3time=925900)

I: So, how is your perception towards this disease of cancer? In terms of treatment for example do you think it is a curable disease, or… what are your perceptions toward this disease? [00:15:43-3](http://localhost:2300/file=C:/Users/Lenovo%20Core%20i5/Desktop/180510_1203_01.mp3time=943300)

R: Currently it is treatable, because in the past people who suffered from this problem were just in bad condition… but now, I see myself… God is making it easy for me. [00:15:57-6](http://localhost:2300/file=C:/Users/Lenovo%20Core%20i5/Desktop/180510_1203_01.mp3time=957600)

I: Your current understanding after you started treatment and before, do you see any difference? [00:16:04-8](http://localhost:2300/file=C:/Users/Lenovo%20Core%20i5/Desktop/180510_1203_01.mp3time=964800)

R: So much. [00:16:04-8](http://localhost:2300/file=C:/Users/Lenovo%20Core%20i5/Desktop/180510_1203_01.mp3time=964800)

I: Which difference? [00:16:09-0](http://localhost:2300/file=C:/Users/Lenovo%20Core%20i5/Desktop/180510_1203_01.mp3time=969000)

R: (laughs) the difference is, as you come to the hospital you keep expanding your thoughts, as you come to see patients, meaning that currently there are many patients, you will find one saying that I had the same problem but am now healed, and others give testimonies, so I also get a belief that I will be fine. [00:16:29-8](http://localhost:2300/file=C:/Users/Lenovo%20Core%20i5/Desktop/180510_1203_01.mp3time=989800)

I: What about stigma, would be stigmatized by relatives…how do you see this in your problem? [00:16:46-1](http://localhost:2300/file=C:/Users/Lenovo%20Core%20i5/Desktop/180510_1203_01.mp3time=1006100)

R: Relatives have not stigmatized me for sure. [00:16:50-2](http://localhost:2300/file=C:/Users/Lenovo%20Core%20i5/Desktop/180510_1203_01.mp3time=1010200)

I: Do you get a good support from relatives? [00:16:50-1](http://localhost:2300/file=C:/Users/Lenovo%20Core%20i5/Desktop/180510_1203_01.mp3time=1010100)

R: Yeah. [00:16:54-4](http://localhost:2300/file=C:/Users/Lenovo%20Core%20i5/Desktop/180510_1203_01.mp3time=1014400)

I: And, have you ever attended health events for example the ones organized by -----, do you know -----? [00:17:03-7](http://localhost:2300/file=C:/Users/Lenovo%20Core%20i5/Desktop/180510_1203_01.mp3time=1023700)

R: No. [00:17:03-7](http://localhost:2300/file=C:/Users/Lenovo%20Core%20i5/Desktop/180510_1203_01.mp3time=1023700)

I: MEWATA, doctors come and provide the services of breast examination? [00:17:12-0](http://localhost:2300/file=C:/Users/Lenovo%20Core%20i5/Desktop/180510_1203_01.mp3time=1032000)

R: I…. okay, I went like two years ago or one month and a half, they announced in ----- that women should go for checking cervical cancer for free, I went for the checkup. [00:17:24-1](http://localhost:2300/file=C:/Users/Lenovo%20Core%20i5/Desktop/180510_1203_01.mp3time=1044100)

I: Was it only cervical cancer or also…[00:17:26-7](http://localhost:2300/file=C:/Users/Lenovo%20Core%20i5/Desktop/180510_1203_01.mp3time=1046700)

R: Aah, only cervical cancer. [00:17:28-9](http://localhost:2300/file=C:/Users/Lenovo%20Core%20i5/Desktop/180510_1203_01.mp3time=1048900)

I: By that time… had that swelling started by then? [00:17:29-6](http://localhost:2300/file=C:/Users/Lenovo%20Core%20i5/Desktop/180510_1203_01.mp3time=1049600)

R: Not yet. [00:17:30-3](http://localhost:2300/file=C:/Users/Lenovo%20Core%20i5/Desktop/180510_1203_01.mp3time=1050300)

I: It had not yet started? [00:17:33-2](http://localhost:2300/file=C:/Users/Lenovo%20Core%20i5/Desktop/180510_1203_01.mp3time=1053200)

R: Mm [00:17:33-2](http://localhost:2300/file=C:/Users/Lenovo%20Core%20i5/Desktop/180510_1203_01.mp3time=1053200)

I: And besides that, do you think there are other reasons maybe to you personally or someone else that you have ever met, that you think are the reasons which make them delay to come for investigations and treatment? [00:18:08-7](http://localhost:2300/file=C:/Users/Lenovo%20Core%20i5/Desktop/180510_1203_01.mp3time=1088700)

R: Mmh, some have witchcraft beliefs, a person may get such a problem and start staying this and that,.. you know, everybody has his/her own faith. [00:18:21-1](http://localhost:2300/file=C:/Users/Lenovo%20Core%20i5/Desktop/180510_1203_01.mp3time=1101100)

I: Yes, and did you think of that personally at some point? [00:18:23-7](http://localhost:2300/file=C:/Users/Lenovo%20Core%20i5/Desktop/180510_1203_01.mp3time=1103700)

R: No, I did not have that belief. [00:18:23-6](http://localhost:2300/file=C:/Users/Lenovo%20Core%20i5/Desktop/180510_1203_01.mp3time=1103600)

I: Your relatives may be? [00:18:29-0](http://localhost:2300/file=C:/Users/Lenovo%20Core%20i5/Desktop/180510_1203_01.mp3time=1109000)

R: Mm no. [00:18:29-0](http://localhost:2300/file=C:/Users/Lenovo%20Core%20i5/Desktop/180510_1203_01.mp3time=1109000)

I: Okay sister, thank you, I have understood, maybe if there is any other thing that you would like to add. [00:18:40-6](http://localhost:2300/file=C:/Users/Lenovo%20Core%20i5/Desktop/180510_1203_01.mp3time=1120600)

R: I am just advising my fellow women, when they get a problem they should not stay home, everything is possible to Allah. [00:18:50-3](http://localhost:2300/file=C:/Users/Lenovo%20Core%20i5/Desktop/180510_1203_01.mp3time=1130300)

I: Alright. [00:18:49-1](http://localhost:2300/file=C:/Users/Lenovo%20Core%20i5/Desktop/180510_1203_01.mp3time=1129100)

R: And currently there are so many researches, people are really doing well, people with this problem and on medications are doing better day by day, a lot is being done about it, it is given a priority. [00:19:09-0](http://localhost:2300/file=C:/Users/Lenovo%20Core%20i5/Desktop/180510_1203_01.mp3time=1149000)

I: And, on the hospital side, what do you think if once improved it will enable most of the women to go early for treatment? [00:19:22-5](http://localhost:2300/file=C:/Users/Lenovo%20Core%20i5/Desktop/180510_1203_01.mp3time=1162500)

R: Ooh, there are things which needs improvement, such as medications, yeah. [00:19:29-5](http://localhost:2300/file=C:/Users/Lenovo%20Core%20i5/Desktop/180510_1203_01.mp3time=1169500)

I: Medications? [00:19:29-5](http://localhost:2300/file=C:/Users/Lenovo%20Core%20i5/Desktop/180510_1203_01.mp3time=1169500)

R: Medications which are being used, they say that they reduce the rate….[00:19:35-9](http://localhost:2300/file=C:/Users/Lenovo%20Core%20i5/Desktop/180510_1203_01.mp3time=1175900)

I: Okay. [00:19:40-0](http://localhost:2300/file=C:/Users/Lenovo%20Core%20i5/Desktop/180510_1203_01.mp3time=1180000)

R: So that is it. [00:19:40-0](http://localhost:2300/file=C:/Users/Lenovo%20Core%20i5/Desktop/180510_1203_01.mp3time=1180000)

I: Okay, thank you then. [00:19:44-3](http://localhost:2300/file=C:/Users/Lenovo%20Core%20i5/Desktop/180510_1203_01.mp3time=1184300)

**CASE 6**

I: Hello sister, what is your name? [00:00:02-9](http://localhost:2300/file=C:/Users/Hp/Desktop/OTONDE/180603_1929.mp3time=2900)

R: My name is ----- [00:00:05-4](http://localhost:2300/file=C:/Users/Hp/Desktop/OTONDE/180603_1929.mp3time=5400)

I: Where are you from? [00:00:06-6](http://localhost:2300/file=C:/Users/Hp/Desktop/OTONDE/180603_1929.mp3time=6600)

R: I am from ----- region,… ----- Municipal [00:00:12-7](http://localhost:2300/file=C:/Users/Hp/Desktop/OTONDE/180603_1929.mp3time=12700)

I: -----, what is your highest level of education? [00:00:16-5](http://localhost:2300/file=C:/Users/Hp/Desktop/OTONDE/180603_1929.mp3time=16500)

R: Diploma [00:00:19-1](http://localhost:2300/file=C:/Users/Hp/Desktop/OTONDE/180603_1929.mp3time=19100)

I: Okay… and what do you do? [00:00:22-4](http://localhost:2300/file=C:/Users/Hp/Desktop/OTONDE/180603_1929.mp3time=22400)

R: I am an entrepreneur; I sell and buy goods, farm products. [00:00:34-2](http://localhost:2300/file=C:/Users/Hp/Desktop/OTONDE/180603_1929.mp3time=34200)

I: Are you married? [00:00:40-3](http://localhost:2300/file=C:/Users/Hp/Desktop/OTONDE/180603_1929.mp3time=40300)

R:.It is complicated… [00:00:42-2](http://localhost:2300/file=C:/Users/Hp/Desktop/OTONDE/180603_1929.mp3time=42200)

I: Ok, and how about children? [00:00:43-3](http://localhost:2300/file=C:/Users/Hp/Desktop/OTONDE/180603_1929.mp3time=43300)

R: I have children [00:00:45-3](http://localhost:2300/file=C:/Users/Hp/Desktop/OTONDE/180603_1929.mp3time=45300)

I: How many children do you have? [00:00:46-6](http://localhost:2300/file=C:/Users/Hp/Desktop/OTONDE/180603_1929.mp3time=46600)

R: I have two children; one is twelve years old and the other one is two years old. [00:00:56-4](http://localhost:2300/file=C:/Users/Hp/Desktop/OTONDE/180603_1929.mp3time=56400)

I: And ----- how far is the nearest health centre from your place, how far is it, is it a walking distance or …? [00:01:10-5](http://localhost:2300/file=C:/Users/Hp/Desktop/OTONDE/180603_1929.mp3time=70500)

R: Aaaa...for the place where I live, since I work at an institute that deals with safety issues, there is a certain dispensary, I think it is a primary one. And the distance is approximately one kilometer from where I live to the largest health centre which is a regional hospital. [00:01:37-6](http://localhost:2300/file=C:/Users/Hp/Desktop/OTONDE/180603_1929.mp3time=97600)

I: So, for you to reach there you need to board a bus? [00:01:41-2](http://localhost:2300/file=C:/Users/Hp/Desktop/OTONDE/180603_1929.mp3time=101200)

R: I can walk or I can take a car, but it is a walkable distance [00:01:47-0](http://localhost:2300/file=C:/Users/Hp/Desktop/OTONDE/180603_1929.mp3time=107000)

I: Ok, and what transport did you use to -----? [00:01:55-0](http://localhost:2300/file=C:/Users/Hp/Desktop/OTONDE/180603_1929.mp3time=115000)

R: I used a car [00:02:03-1](http://localhost:2300/file=C:/Users/Hp/Desktop/OTONDE/180603_1929.mp3time=123100)

I: Okay, do you use a smart phone? I want to know how you get the information aboout breast cancer [00:02:12-2](http://localhost:2300/file=C:/Users/Hp/Desktop/OTONDE/180603_1929.mp3time=132200)

R: I got breast disease in the year ----- but by then I was young. I heard about the disease through the radio, RTD station, and I came to hear about it in the year ----- after being pregnant when I was attending the reproductive health centre. They informed us about the disease, so, I was aware with that. They taught us as part of maternal health. [00:02:51-0](http://localhost:2300/file=C:/Users/Hp/Desktop/OTONDE/180603_1929.mp3time=171000)

I: Okay… [00:02:52-3](http://localhost:2300/file=C:/Users/Hp/Desktop/OTONDE/180603_1929.mp3time=172300)

R: They taught us on the symptoms, and then later on when I was working, I heard an announcement from the -----, that they were doing outreach programs in different regions in the country. It was in 2016, I therefore attended the sessions and I was screened. [00:03:12-8](http://localhost:2300/file=C:/Users/Hp/Desktop/OTONDE/180603_1929.mp3time=192800)

I: Okay [00:03:14-4](http://localhost:2300/file=C:/Users/Hp/Desktop/OTONDE/180603_1929.mp3time=194400)

R: And from that I knew certain things concerning breast cancer, cervical cancer, throat cancer, lung in these public meetings. [00:03:29-8](http://localhost:2300/file=C:/Users/Hp/Desktop/OTONDE/180603_1929.mp3time=209800)

I: Mmmh [00:03:31-2](http://localhost:2300/file=C:/Users/Hp/Desktop/OTONDE/180603_1929.mp3time=211200)

R: Also, I got fliers which are very recent, there is where I got the knowledge from. [00:03:44-4](http://localhost:2300/file=C:/Users/Hp/Desktop/OTONDE/180603_1929.mp3time=224400)

I: So what do you know about breast cancer? [00:03:46-4](http://localhost:2300/file=C:/Users/Hp/Desktop/OTONDE/180603_1929.mp3time=226400)

R: Breast cancer is the accumulation of cells, which are dead cells, which when they stay for long end up forming a lump, and when they mature, they transform into cancer which continue to multiply until they become matured, (she laughs), that is my understanding. [00:04:15-6](http://localhost:2300/file=C:/Users/Hp/Desktop/OTONDE/180603_1929.mp3time=255600)

I: Okay..... [00:04:16-6](http://localhost:2300/file=C:/Users/Hp/Desktop/OTONDE/180603_1929.mp3time=256600)

R: But it is a result of lifestyle [00:04:21-8](http://localhost:2300/file=C:/Users/Hp/Desktop/OTONDE/180603_1929.mp3time=261800)

I: What was the first symptom you experienced? [00:04:25-2](http://localhost:2300/file=C:/Users/Hp/Desktop/OTONDE/180603_1929.mp3time=265200)

R: The first thing I noticed…, I was breastfeeding, I got a lump in the breast. It was painless. So after I noticed it, I went to the hospital. [00:04:40-6](http://localhost:2300/file=C:/Users/Hp/Desktop/OTONDE/180603_1929.mp3time=280600)

I: Mmmh [00:04:43-2](http://localhost:2300/file=C:/Users/Hp/Desktop/OTONDE/180603_1929.mp3time=283200)

R: At the regional hospital they told me that it was an abscess, and because it didn’t show where it originated from then they decided to give me antibiotics. They gave me the medications, and after sometime, I didn’t notice it turning out to be an abscess, so I went to -----. [00:05:09-7](http://localhost:2300/file=C:/Users/Hp/Desktop/OTONDE/180603_1929.mp3time=309700)

I: Mmmh [00:05:10-1](http://localhost:2300/file=C:/Users/Hp/Desktop/OTONDE/180603_1929.mp3time=310100)

R: The hospital is called ----- Hospital. I heard that there are specialists in that hospital who are capable of such things. So, I decided to go there, when I reached there, they also said that the swelling was an abscess [00:05:30-1](http://localhost:2300/file=C:/Users/Hp/Desktop/OTONDE/180603_1929.mp3time=330100)

I: Okay… [00:05:31-0](http://localhost:2300/file=C:/Users/Hp/Desktop/OTONDE/180603_1929.mp3time=331000)

R: We agreed on the way forward, to extract it but it didn’t work out. [00:05:35-2](http://localhost:2300/file=C:/Users/Hp/Desktop/OTONDE/180603_1929.mp3time=335200)

I: At -----kapa? [00:05:38-1](http://localhost:2300/file=C:/Users/Hp/Desktop/OTONDE/180603_1929.mp3time=338100)

R: Yeah, because they were delaying, and by that time I had already began to experience pain. [00:05:43-2](http://localhost:2300/file=C:/Users/Hp/Desktop/OTONDE/180603_1929.mp3time=343200)

I: Were you still breastfeeding by then? [00:05:45-3](http://localhost:2300/file=C:/Users/Hp/Desktop/OTONDE/180603_1929.mp3time=345300)

R: No, I had stopped breastfeeding after knowing that I had the lump. [00:05:50-7](http://localhost:2300/file=C:/Users/Hp/Desktop/OTONDE/180603_1929.mp3time=350700)

I: But how long did it take for you to go to the hospital from the first day you noticed the lump? [00:06:01-2](http://localhost:2300/file=C:/Users/Hp/Desktop/OTONDE/180603_1929.mp3time=361200)

R: I went to the hospital just on the next day after noticing the lump [00:06:04-8](http://localhost:2300/file=C:/Users/Hp/Desktop/OTONDE/180603_1929.mp3time=364800)

I: How did you notice it; did you notice it yourself or somebody did…. [00:06:07-4](http://localhost:2300/file=C:/Users/Hp/Desktop/OTONDE/180603_1929.mp3time=367400)

R: As I was touching myself, I felt there is something. Then I communicated with a certain man who works at a private hospital, and he told me to go to the hospital on the following day, and he insisted not to go to such hospitals but rather go to the regional hospital because they screen for such issues. [00:06:28-0](http://localhost:2300/file=C:/Users/Hp/Desktop/OTONDE/180603_1929.mp3time=388000)

I: Mmmh [00:06:29-1](http://localhost:2300/file=C:/Users/Hp/Desktop/OTONDE/180603_1929.mp3time=389100)

R: So, I went there but I wasn’t satisfied with the results they gave me, so I decided to go to ----- where I was told that I had a lump and it was an abscess, but then I saw that the situation is also not good, because it had already began to be painful, there is when I requested for a referral letter from the Regional hospital, and they gave me the referral [00:06:58-1](http://localhost:2300/file=C:/Users/Hp/Desktop/OTONDE/180603_1929.mp3time=418100)

I: referral to...... [00:06:58-9](http://localhost:2300/file=C:/Users/Hp/Desktop/OTONDE/180603_1929.mp3time=418900)

R: To come to -----. When I came to -----, it was still small but still inside, and when they touch it, it slipped away. Then they said they should take a biopsy so that they can test and know what it was. [00:07:19-1](http://localhost:2300/file=C:/Users/Hp/Desktop/OTONDE/180603_1929.mp3time=439100)

I: Yes… [00:07:20-8](http://localhost:2300/file=C:/Users/Hp/Desktop/OTONDE/180603_1929.mp3time=440800)

R: They took the biopsy, told me to wait for the results within one month. Within one month the breast lump was increasing in size, and having some growths here and there. Therefore, I had to go back to the hospital before the thirty one days were due, and I told them that my condition had worsened a lot.[00:07:45-1](http://localhost:2300/file=C:/Users/Hp/Desktop/OTONDE/180603_1929.mp3time=465100)

I: Before getting the results? [00:07:47-2](http://localhost:2300/file=C:/Users/Hp/Desktop/OTONDE/180603_1929.mp3time=467200)

R: Before getting the results. I was given some medications to relieve pain and was given a date to return to the hospital. I came and they gave me the results and referred me to ----- for chemotherapy. At ----- I underwent seven cycles of two types of medications. [00:08:09-0](http://localhost:2300/file=C:/Users/Hp/Desktop/OTONDE/180603_1929.mp3time=489000)

I: Ok … [00:08:10-5](http://localhost:2300/file=C:/Users/Hp/Desktop/OTONDE/180603_1929.mp3time=490500)

R: It began to shrink, and the hardness disappeared, and the bleeding was stopped. I stayed at ----- for sometime and they referred me back here (at -----) for surgery. [00:08:27-1](http://localhost:2300/file=C:/Users/Hp/Desktop/OTONDE/180603_1929.mp3time=507100)

I: You said that biopsy was done, did you do any imaging investigations like ultrasound? [00:08:33-3](http://localhost:2300/file=C:/Users/Hp/Desktop/OTONDE/180603_1929.mp3time=513300)

R: I did the Ultrasound here at ----- and -----, after every one month. [00:08:41-1](http://localhost:2300/file=C:/Users/Hp/Desktop/OTONDE/180603_1929.mp3time=521100)

I: What about at the first hospitals that you attended? [00:08:49-2](http://localhost:2300/file=C:/Users/Hp/Desktop/OTONDE/180603_1929.mp3time=529200)

R: At -----, I did ultrasound and x-ray, as well as mammography [00:08:58-3](http://localhost:2300/file=C:/Users/Hp/Desktop/OTONDE/180603_1929.mp3time=538300)

I: How long did it take to do the imaging from the day you experienced the symptoms to the time you did those imaging, the mammography and ultrasound? [00:09:08-3](http://localhost:2300/file=C:/Users/Hp/Desktop/OTONDE/180603_1929.mp3time=548300)

R: Four months [00:09:11-8](http://localhost:2300/file=C:/Users/Hp/Desktop/OTONDE/180603_1929.mp3time=551800)

I: Personally, do you think you have delayed to get the medical attention? [00:09:17-7](http://localhost:2300/file=C:/Users/Hp/Desktop/OTONDE/180603_1929.mp3time=557700)

R: I cannot say by how much percent, but there is a time where I did wrong, and there are areas where the health care providers did wrong. For example, the doctors at the regional hospital, they were not specialists in these things, they were not supposed to mess with something they are not specialized with, they should refer the patient immediately. [00:09:40-8](http://localhost:2300/file=C:/Users/Hp/Desktop/OTONDE/180603_1929.mp3time=580800)

I: Mmmh [00:09:42-4](http://localhost:2300/file=C:/Users/Hp/Desktop/OTONDE/180603_1929.mp3time=582400)

R: I cannot just wake up from nowhere and say that this is cancer when I do not know much about it. [00:09:48-0](http://localhost:2300/file=C:/Users/Hp/Desktop/OTONDE/180603_1929.mp3time=588000)

I: Where there any difficulties in getting the referral? [00:09:54-0](http://localhost:2300/file=C:/Users/Hp/Desktop/OTONDE/180603_1929.mp3time=594000)

R: Because they were confident that it was an abscess. [00:09:55-4](http://localhost:2300/file=C:/Users/Hp/Desktop/OTONDE/180603_1929.mp3time=595400)

I: Okay… [00:09:56-8](http://localhost:2300/file=C:/Users/Hp/Desktop/OTONDE/180603_1929.mp3time=596800)

R: Sometime they said the milk in my breast had gone bad…. I was still in maternity [00:10:06-6](http://localhost:2300/file=C:/Users/Hp/Desktop/OTONDE/180603_1929.mp3time=606600)

I: And you also said that you had personal reasons, what are these reasons that made delay? [00:10:13-4](http://localhost:2300/file=C:/Users/Hp/Desktop/OTONDE/180603_1929.mp3time=613400)

R: If I knew earlier, the day I was told to go to the government hospital, I wish I had gone to ----- straight or come to ----- straight, meaning that they would have operated me earlier and I would have continued with chemo. [00:10:30-5](http://localhost:2300/file=C:/Users/Hp/Desktop/OTONDE/180603_1929.mp3time=630500)

I: Was there any difficulty in going directly? [00:10:32-4](http://localhost:2300/file=C:/Users/Hp/Desktop/OTONDE/180603_1929.mp3time=632400)

R: I wasn’t aware at all, I was blind, I had all those options but… There are specialists at ----- and -----, but they had different levels. So, when I came here, I found that, I could have come earlier, and gone to ocean road and things would have been better… [00:11:13-7](http://localhost:2300/file=C:/Users/Hp/Desktop/OTONDE/180603_1929.mp3time=673700)

I: Are there any treatments you underwent before going to the hospital, such as traditional ways of treatment, or prayers, did you do such things before going to the hospital? [00:11:25-2](http://localhost:2300/file=C:/Users/Hp/Desktop/OTONDE/180603_1929.mp3time=685200)

R: No, I do pray every day, I do not pray because I got the disease. [00:11:33-7](http://localhost:2300/file=C:/Users/Hp/Desktop/OTONDE/180603_1929.mp3time=693700)

I: is it usual for you to say your prayers? [00:11:39-9](http://localhost:2300/file=C:/Users/Hp/Desktop/OTONDE/180603_1929.mp3time=699900)

R: I do not pray because I have cancer problem, that is my daily routine [00:11:49-9](http://localhost:2300/file=C:/Users/Hp/Desktop/OTONDE/180603_1929.mp3time=709900)

I: Okay [00:11:51-7](http://localhost:2300/file=C:/Users/Hp/Desktop/OTONDE/180603_1929.mp3time=711700)

R: And I don’t go to traditional healers, but there are certain people who I am sure that they have cancer and they are doing such things of going to the traditional healers [00:12:05-9](http://localhost:2300/file=C:/Users/Hp/Desktop/OTONDE/180603_1929.mp3time=725900)

I: What about family support, did it contribute to the delay of coming to the hospital? [00:12:22-0](http://localhost:2300/file=C:/Users/Hp/Desktop/OTONDE/180603_1929.mp3time=742000)

R: No, by the way I do not live with my family, I am alone with my kids, therefore, I plan myself, and about the issue of being scared, I am not scared about hospital issues because if at all I was I wouldn’t have the guts to go to the regional hospital where I went. [00:12:54-4](http://localhost:2300/file=C:/Users/Hp/Desktop/OTONDE/180603_1929.mp3time=774400)

I: As per your knowledge, is there anyone from your family who had ever had cancer? [00:13:06-3](http://localhost:2300/file=C:/Users/Hp/Desktop/OTONDE/180603_1929.mp3time=786300)

R: On my father’s side there is one of his relatives who died of esophageal cancer, but he was a doctor who smoked a lot and drank strong alcohols. But I don’t know if that relates with this. [00:13:20-9](http://localhost:2300/file=C:/Users/Hp/Desktop/OTONDE/180603_1929.mp3time=800900)

I: Esophageal cancer? [00:13:21-9](http://localhost:2300/file=C:/Users/Hp/Desktop/OTONDE/180603_1929.mp3time=801900)

R: Yeah, he was swollen on the neck though I didn’t know if that was throat cancer or not. [00:13:39-4](http://localhost:2300/file=C:/Users/Hp/Desktop/OTONDE/180603_1929.mp3time=819400)

I: Okay. -----, do you use health insurance services? [00:13:43-7](http://localhost:2300/file=C:/Users/Hp/Desktop/OTONDE/180603_1929.mp3time=823700)

R: No, I don’t. [00:13:44-8](http://localhost:2300/file=C:/Users/Hp/Desktop/OTONDE/180603_1929.mp3time=824800)

I: So you pay cash? [00:13:46-2](http://localhost:2300/file=C:/Users/Hp/Desktop/OTONDE/180603_1929.mp3time=826200)

R: I pay cash [00:13:52-1](http://localhost:2300/file=C:/Users/Hp/Desktop/OTONDE/180603_1929.mp3time=832100)

I: Is that a barrier to.... [00:13:53-5](http://localhost:2300/file=C:/Users/Hp/Desktop/OTONDE/180603_1929.mp3time=833500)

R: It is a barrier but I have no way out, I must recover, I should look for money here and there [00:14:02-6](http://localhost:2300/file=C:/Users/Hp/Desktop/OTONDE/180603_1929.mp3time=842600)

I: But in your process of treatment or follow up, do you think that may also be one of the personal reasons for delay? [00:14:12-3](http://localhost:2300/file=C:/Users/Hp/Desktop/OTONDE/180603_1929.mp3time=852300)

R: No [00:14:14-9](http://localhost:2300/file=C:/Users/Hp/Desktop/OTONDE/180603_1929.mp3time=854900)

I: no? [00:14:15-7](http://localhost:2300/file=C:/Users/Hp/Desktop/OTONDE/180603_1929.mp3time=855700)

R: By that time, I had a lot of cash since I was still working, so it wasn’t a reason to be honest. Maybe if I wouldn’t have wanted to come to the hospital in the near days that could be another factor that would have made me stop the treatment processes, but money is not the reason. [00:14:37-2](http://localhost:2300/file=C:/Users/Hp/Desktop/OTONDE/180603_1929.mp3time=877200)

I: Mmmh [00:14:39-3](http://localhost:2300/file=C:/Users/Hp/Desktop/OTONDE/180603_1929.mp3time=879300)

R: Because government institutions can lend me money for treatment and I would come to pay later on. [00:14:52-1](http://localhost:2300/file=C:/Users/Hp/Desktop/OTONDE/180603_1929.mp3time=892100)

I: So, what are the main reasons that have led to this? [00:15:01-5](http://localhost:2300/file=C:/Users/Hp/Desktop/OTONDE/180603_1929.mp3time=901500)

R: The reason is those doctors from the regional hospital, if they know that it is not their field, then they should not temper with things that could cost somebody’s life, and they should make the referral system more official. [00:15:15-4](http://localhost:2300/file=C:/Users/Hp/Desktop/OTONDE/180603_1929.mp3time=915400)

I: Getting the referral? [00:15:17-1](http://localhost:2300/file=C:/Users/Hp/Desktop/OTONDE/180603_1929.mp3time=917100)

R: Yeah [00:15:18-4](http://localhost:2300/file=C:/Users/Hp/Desktop/OTONDE/180603_1929.mp3time=918400)

I: Ahaaa [00:15:20-0](http://localhost:2300/file=C:/Users/Hp/Desktop/OTONDE/180603_1929.mp3time=920000)

R: They have a lot of bureaucracy, for instance I got my referral after two days and it was after I over reacted, and at the end they gave me referral [00:15:33-3](http://localhost:2300/file=C:/Users/Hp/Desktop/OTONDE/180603_1929.mp3time=933300)

I: so, there is not only treatment but also referral? [00:15:38-5](http://localhost:2300/file=C:/Users/Hp/Desktop/OTONDE/180603_1929.mp3time=938500)

R: Referral, because if you are seen by the surgeon, then you are supposed to give a referral because you know that I have no ability to cure this patient, so she should go to see another surgeon and report so that they can start another process. He/she is only supposed to write a letter, it is then typed and signed, but even that is so difficult to deal with. [00:16:10-3](http://localhost:2300/file=C:/Users/Hp/Desktop/OTONDE/180603_1929.mp3time=970300)

I: How many days did it take until you got the referral letter? [00:16:10-6](http://localhost:2300/file=C:/Users/Hp/Desktop/OTONDE/180603_1929.mp3time=970600)

R: It took me two and a half days but it was after I told them. I was told he is in the operating room, but I asked “will he be there for the whole day?”. So, you find that sometimes there are unnecessary procedures, they follow outdated protocols. Because currently all hospitals have a computer, he would have put it in a soft copy and they give it to me, off I go. [00:16:38-7](http://localhost:2300/file=C:/Users/Hp/Desktop/OTONDE/180603_1929.mp3time=998700)

I: Mmmh [00:16:40-7](http://localhost:2300/file=C:/Users/Hp/Desktop/OTONDE/180603_1929.mp3time=1000700)

R: Within seven minutes everything is done, so, I would like to advise these government employees that they should use the technology they have for the aim of simplifying treatment to patients, not because they feel happy when patients come. But also as an institute they are supposed to visit us at our home places/communities and see how live, what we eat to prevent these strange diseases. [00:17:11-9](http://localhost:2300/file=C:/Users/Hp/Desktop/OTONDE/180603_1929.mp3time=1031900)

I: It is true. [00:17:12-8](http://localhost:2300/file=C:/Users/Hp/Desktop/OTONDE/180603_1929.mp3time=1032800)

R: But they like staying in the offices for us to follow them [00:17:16-7](http://localhost:2300/file=C:/Users/Hp/Desktop/OTONDE/180603_1929.mp3time=1036700)

I: So, you mean that there are more treatment alone? [00:17:26-0](http://localhost:2300/file=C:/Users/Hp/Desktop/OTONDE/180603_1929.mp3time=1046000)

R: Yeah [00:17:29-6](http://localhost:2300/file=C:/Users/Hp/Desktop/OTONDE/180603_1929.mp3time=1049600)

I: Were you not scared of cancer treatments? Maybe to undergo surgery or receive strong medication? [00:17:40-7](http://localhost:2300/file=C:/Users/Hp/Desktop/OTONDE/180603_1929.mp3time=1060700)

R: They didn’t scare me, because after knowing that I have cancer, I said Thank you God for I have known it earlier. And about the surgery, it isn’t my first operation, and I think it won’t be the last one, others will also come and they will all pass.[00:18:00-0](http://localhost:2300/file=C:/Users/Hp/Desktop/OTONDE/180603_1929.mp3time=1080000)

I: Mmmh [00:18:01-1](http://localhost:2300/file=C:/Users/Hp/Desktop/OTONDE/180603_1929.mp3time=1081100)

R: So I am not scared because of the operation and about the treatment…. Those chemotherapy scared me a bit after seeing some changes that came around in the first place, they were so rapid, and I didn’t expect that it will be this and this, I was thinking a lot, I knew that there are going to be some skin changes, hair loss and changes in my bone composition.[00:18:33-1](http://localhost:2300/file=C:/Users/Hp/Desktop/OTONDE/180603_1929.mp3time=1113100)

I: Mmmh [00:18:34-7](http://localhost:2300/file=C:/Users/Hp/Desktop/OTONDE/180603_1929.mp3time=1114700)

R: But also, there are other changes that I didn’t know about, for instance about drop down of the CD4, when it goes low you change, you get skin rashes all over, but I continued to have my good diets normally and I recovered. So, I saw it as a normal thing.[00:18:55-4](http://localhost:2300/file=C:/Users/Hp/Desktop/OTONDE/180603_1929.mp3time=1135400)

I: Are your relatives aware of this? [00:18:59-8](http://localhost:2300/file=C:/Users/Hp/Desktop/OTONDE/180603_1929.mp3time=1139800)

R: Being aware of? [00:19:02-1](http://localhost:2300/file=C:/Users/Hp/Desktop/OTONDE/180603_1929.mp3time=1142100)

I: Knowing that you have this problem [00:19:05-3](http://localhost:2300/file=C:/Users/Hp/Desktop/OTONDE/180603_1929.mp3time=1145300)

R: A few [00:19:06-3](http://localhost:2300/file=C:/Users/Hp/Desktop/OTONDE/180603_1929.mp3time=1146300)

I: Few… why? [00:19:07-7](http://localhost:2300/file=C:/Users/Hp/Desktop/OTONDE/180603_1929.mp3time=1147700)

R: That is how I am, I don’t like to disclose my personal matters to my relatives [00:19:11-6](http://localhost:2300/file=C:/Users/Hp/Desktop/OTONDE/180603_1929.mp3time=1151600)

I: but not that you think they will may be discriminate you?... [00:19:17-4](http://localhost:2300/file=C:/Users/Hp/Desktop/OTONDE/180603_1929.mp3time=1157400)

R: There is no one to discriminate me, because there are some of them that I have never share anything with them and they are my elders, others are my younger siblings. I don’t have that time. [00:19:34-1](http://localhost:2300/file=C:/Users/Hp/Desktop/OTONDE/180603_1929.mp3time=1174100)

I: So, it is not because of the disease, but it is in all matters.[00:19:36-6](http://localhost:2300/file=C:/Users/Hp/Desktop/OTONDE/180603_1929.mp3time=1176600)

R: All matters pertaining to life [00:19:40-7](http://localhost:2300/file=C:/Users/Hp/Desktop/OTONDE/180603_1929.mp3time=1180700)

I: Did you also think of the swelling when they told you that it will resolve at some point on its own? [00:19:51-1](http://localhost:2300/file=C:/Users/Hp/Desktop/OTONDE/180603_1929.mp3time=1191100)

R: No [00:19:51-8](http://localhost:2300/file=C:/Users/Hp/Desktop/OTONDE/180603_1929.mp3time=1191800)

I: You didn’t think of such a thing? [00:19:54-8](http://localhost:2300/file=C:/Users/Hp/Desktop/OTONDE/180603_1929.mp3time=1194800)

R: Mmmh [00:19:56-2](http://localhost:2300/file=C:/Users/Hp/Desktop/OTONDE/180603_1929.mp3time=1196200)

I: Okay V, what are your recommendations so as to help people seek medical treatment early? [00:20:11-5](http://localhost:2300/file=C:/Users/Hp/Desktop/OTONDE/180603_1929.mp3time=1211500)

R: The first thing, I would like to ask the government through their administration to make sure that such information about cancer, TB and other diseases, should be also be told to those living in the villages for example, you may find that many women who have cancer of the cervix but do not know that they have cancer of the cervix, but when they tell you that I feel this and that, you just know t is the same thing.[00:20:41-8](http://localhost:2300/file=C:/Users/Hp/Desktop/OTONDE/180603_1929.mp3time=1241800)

I: Okay… [00:20:44-9](http://localhost:2300/file=C:/Users/Hp/Desktop/OTONDE/180603_1929.mp3time=1244900)

R: And they use local herbs to treat themselves, they destroy themselves and worsen the condition because they put when it is fulminant, they are told to squat over a bucket with very hot water, which burns her. [00:21:00-5](http://localhost:2300/file=C:/Users/Hp/Desktop/OTONDE/180603_1929.mp3time=1260500)

I: Ahaa, what places are these? Because you know traditional ways of handling diseases differ from society to society.[00:21:11-0](http://localhost:2300/file=C:/Users/Hp/Desktop/OTONDE/180603_1929.mp3time=1271000)

R: I am speaking referring to Iringa region where most of the times I do my business and I work in different villages. That is what they do, or you find that somebody else is severely sick, so she ends up saying that she is bewitched, and she is going to the witch doctor, in the end she looses blood and she dies and then she complains that somebody has bewitched her. [00:21:33-8](http://localhost:2300/file=C:/Users/Hp/Desktop/OTONDE/180603_1929.mp3time=1293800)

I: Yes [00:21:35-3](http://localhost:2300/file=C:/Users/Hp/Desktop/OTONDE/180603_1929.mp3time=1295300)

R: Others say that people are jealous of her, because my child has built a house and they believe. [00:21:40-6](http://localhost:2300/file=C:/Users/Hp/Desktop/OTONDE/180603_1929.mp3time=1300600)

I: I see, everything is different… [00:21:42-5](http://localhost:2300/file=C:/Users/Hp/Desktop/OTONDE/180603_1929.mp3time=1302500)

R: Eeeh, and there are others who have studied and they are very religious, they go to church and they know that today I will be healed. But the bible says, “My people are perishing because they lack knowledge”. God had put the doctors knowing that a human’s body will in one way or the other get tired so they must get medical treatment.[00:22:07-6](http://localhost:2300/file=C:/Users/Hp/Desktop/OTONDE/180603_1929.mp3time=1327600)

I: Okay .... [00:22:09-5](http://localhost:2300/file=C:/Users/Hp/Desktop/OTONDE/180603_1929.mp3time=1329500)

R: And that is why he had put doctors and gave them the ability to know that when you mix this tree and that tree you will get a certain kind of medicine. And some of them have studied so much but instead of going to the specialized areas they go to ….I don’t disagree that it is his faith that guides somebody, but most of them will perish [00:22:36-2](http://localhost:2300/file=C:/Users/Hp/Desktop/OTONDE/180603_1929.mp3time=1356200)

I: Because they do not know [00:22:38-2](http://localhost:2300/file=C:/Users/Hp/Desktop/OTONDE/180603_1929.mp3time=1358200)

R: Yes, and another thing, I would like to ask the government to get rid of these doctors who identify themselves as doctor ----- from Nigeria, or Korea, I cure cancer. [00:22:53-9](http://localhost:2300/file=C:/Users/Hp/Desktop/OTONDE/180603_1929.mp3time=1373900)

I: Are there so many people who follow them? [00:22:54-8](http://localhost:2300/file=C:/Users/Hp/Desktop/OTONDE/180603_1929.mp3time=1374800)

R: Most of them follow them and that medicine for cancer for what I believe, and cancer cannot be totally removed in the body of a human being. It was there before you know it, and even after they do surgery it remains there, it cannot be totally eradicated. [00:23:14-3](http://localhost:2300/file=C:/Users/Hp/Desktop/OTONDE/180603_1929.mp3time=1394300)

I: It is completely eradicated [00:23:15-8](http://localhost:2300/file=C:/Users/Hp/Desktop/OTONDE/180603_1929.mp3time=1395800)

R: You see, so they keep lying to people saying that they cure all cancers, and when they go there, the cancer spreads faster and a person is gone, you waste your money, its better if you would have come to these government hospitals, and -----and ----- in particular. She will incur less charges.[00:23:36-6](http://localhost:2300/file=C:/Users/Hp/Desktop/OTONDE/180603_1929.mp3time=1416600)

I: Mmmh [00:23:38-3](http://localhost:2300/file=C:/Users/Hp/Desktop/OTONDE/180603_1929.mp3time=1418300)

R: Those are the second people, their licenses of doing business should be confiscated because they are the ones who contribute in misleading people. They give them medications, supplements, supplement is okay but not that you say it cures cancer by hundred percent because their advertisements say that they cure cancer by hundred percent. [00:23:59-4](http://localhost:2300/file=C:/Users/Hp/Desktop/OTONDE/180603_1929.mp3time=1439400)

I: They cure by hundred percent? [00:24:00-2](http://localhost:2300/file=C:/Users/Hp/Desktop/OTONDE/180603_1929.mp3time=1440200)

R: Yes, they say they cure by hundred percent, and that is very bad. You find that somebody announces that much, why shouldn’t these hospitals do as how they do. So, somebody who can sit down and ponder about the news will be saved but other people cannot do that and in the end they go there and that becomes the end of her. [00:24:20-4](http://localhost:2300/file=C:/Users/Hp/Desktop/OTONDE/180603_1929.mp3time=1460400)

I: Have you ever attended the treatment centre for these people who use alternative medication, the ones who advertise their business?[00:24:30-9](http://localhost:2300/file=C:/Users/Hp/Desktop/OTONDE/180603_1929.mp3time=1470900)

R: No [00:24:31-8](http://localhost:2300/file=C:/Users/Hp/Desktop/OTONDE/180603_1929.mp3time=1471800)

I: You have never? [00:24:34-4](http://localhost:2300/file=C:/Users/Hp/Desktop/OTONDE/180603_1929.mp3time=1474400)

R: I have never fallen sick to an extent that I decide to go to a traditional healer. The last time I fell sick was ten years ago, so all others were due to pregnancy, flue, but the greatest thing that had strike me is this, cancer. [00:24:52-6](http://localhost:2300/file=C:/Users/Hp/Desktop/OTONDE/180603_1929.mp3time=1492600)

I: Okay … [00:24:53-8](http://localhost:2300/file=C:/Users/Hp/Desktop/OTONDE/180603_1929.mp3time=1493800)

R: But in all other days in the past I have never fallen sick. [00:24:57-6](http://localhost:2300/file=C:/Users/Hp/Desktop/OTONDE/180603_1929.mp3time=1497600)

I: V, I know that there is this investigation …. you told me that among the first investigations you did was mammography and ultrasound and they are among the first investigations that most people do, are there any difficulties in getting access to these? [00:25:10-1](http://localhost:2300/file=C:/Users/Hp/Desktop/OTONDE/180603_1929.mp3time=1510100)

R: Yeah, for instance, at ----- hospital, it takes two days for you to get the mammography results. [00:25:23-3](http://localhost:2300/file=C:/Users/Hp/Desktop/OTONDE/180603_1929.mp3time=1523300)

I: Two days? [00:25:25-0](http://localhost:2300/file=C:/Users/Hp/Desktop/OTONDE/180603_1929.mp3time=1525000)

R: You are told that, come tomorrow for the results of the ultrasound you did today. But from what I know, it is simple, electronic devices, you read results, you send it to the doctor, he gets the results, if you want a copy, he prints it out. [00:25:39-8](http://localhost:2300/file=C:/Users/Hp/Desktop/OTONDE/180603_1929.mp3time=1539800)

I: Mmmh [00:25:40-7](http://localhost:2300/file=C:/Users/Hp/Desktop/OTONDE/180603_1929.mp3time=1540700)

R: But then they tell you come tomorrow, it means that, he is not sure of what he is doing, he is not sure what he is doing in that screen, so he needs to tell his fellows and ask them of what is seen in the ultrasound. [00:25:58-7](http://localhost:2300/file=C:/Users/Hp/Desktop/OTONDE/180603_1929.mp3time=1558700)

I: But what about the availability...... [00:26:01-9](http://localhost:2300/file=C:/Users/Hp/Desktop/OTONDE/180603_1929.mp3time=1561900)

R: it is difficult to access it here, even at the national hospital, they need to add more facilities, you cannot call it a national hospital with one … [00:26:17-9](http://localhost:2300/file=C:/Users/Hp/Desktop/OTONDE/180603_1929.mp3time=1577900)

I: Mammography machine [00:26:19-1](http://localhost:2300/file=C:/Users/Hp/Desktop/OTONDE/180603_1929.mp3time=1579100)

R: Not mammography, the ultrasound, it is very small, a big room such as this is required, and ten doctors enter in the room all at once, they attend ten patients, then others follow, because otherwise there is a long queue, you make an appointment, what if I am going to die today [00:26:40-4](http://localhost:2300/file=C:/Users/Hp/Desktop/OTONDE/180603_1929.mp3time=1600400)

I: And you have not yet got the results?... [00:26:42-9](http://localhost:2300/file=C:/Users/Hp/Desktop/OTONDE/180603_1929.mp3time=1602900)

R: I haven’t got the results, and probably the results would have helped me today because doctors are there for us and we here for them, and for example, I have got the results today at one in the afternoon and I went to tell the doctor about the results, it was possible that she should be given the painkillers first then drip then pother things follow and you will not die anymore [00:27:05-8](http://localhost:2300/file=C:/Users/Hp/Desktop/OTONDE/180603_1929.mp3time=1625800)

I: Hmmm [00:27:06-7](http://localhost:2300/file=C:/Users/Hp/Desktop/OTONDE/180603_1929.mp3time=1626700)

R: Yes, that is the reality, you know the other thing that we government people are not capable of compared to those of private is that those in the private hospitals have got many facilities. [00:27:20-9](http://localhost:2300/file=C:/Users/Hp/Desktop/OTONDE/180603_1929.mp3time=1640900)

I: The private hospitals? [00:27:24-7](http://localhost:2300/file=C:/Users/Hp/Desktop/OTONDE/180603_1929.mp3time=1644700)

R: Yeah, they have the facilities, three ultrasound machines, maybe two x-ray machines because they are well off and they have people who are available all the time that is why there is no bureaucracy. You go there, you do your investigations and you get your results faster. [00:27:42-4](http://localhost:2300/file=C:/Users/Hp/Desktop/OTONDE/180603_1929.mp3time=1662400)

I: That is fast… [00:27:44-2](http://localhost:2300/file=C:/Users/Hp/Desktop/OTONDE/180603_1929.mp3time=1664200)

R: You get your results faster, off you go, there is no wastage of time, that is why it becomes easy, and good. I am not saying that they are bad, they are good but they delay [00:28:01-8](http://localhost:2300/file=C:/Users/Hp/Desktop/OTONDE/180603_1929.mp3time=1681800)

I: They delay? [00:28:04-2](http://localhost:2300/file=C:/Users/Hp/Desktop/OTONDE/180603_1929.mp3time=1684200)

R: And sometimes those who do the x-ray tell you that you will get your results on the day of the appointment, but sometimes it is the system itself. For example, those who do the x-ray tell you that you will get the results on the day of the appointment, so I ask myself, those results usually delay, what do they do with them? [00:28:34-1](http://localhost:2300/file=C:/Users/Hp/Desktop/OTONDE/180603_1929.mp3time=1714100)

I: Ahaa… [00:28:44-5](http://localhost:2300/file=C:/Users/Hp/Desktop/OTONDE/180603_1929.mp3time=1724500)

R: To those patients, because everyone wants to see the other recovering, for example, for cancer patients, there are certain foods when they are told to eat, if it is in any stage, it helps in reducing the cancer spread, I am sure. [00:29:10-0](http://localhost:2300/file=C:/Users/Hp/Desktop/OTONDE/180603_1929.mp3time=1750000)

I: Things about food [00:29:11-1](http://localhost:2300/file=C:/Users/Hp/Desktop/OTONDE/180603_1929.mp3time=1751100)

R: Things about food, the anti-cancer, anti-oxidants foods. Doctors responsible for that or responsible for cancer issues, there should be a department of counselling and advise. Somebody starts the dose, there is nobody to advise her that you should eat certain foods, when you come it is only chemotherapy, they only do the blood investigations, they check if your heart is well, the lungs, the kidneys, then you get your chemo. They don’t tell you what kind of foods you are supposed to eat, so there should be a specific place. [00:29:53-5](http://localhost:2300/file=C:/Users/Hp/Desktop/OTONDE/180603_1929.mp3time=1793500)

I: Mmmh [00:29:54-4](http://localhost:2300/file=C:/Users/Hp/Desktop/OTONDE/180603_1929.mp3time=1794400)

R: There should be a department which advises her on what kinds of foods she should take, for example fruits, what kind of fruits is she supposed to take which have high antioxidant ability, what kind of fruits should I eat and when should I eat them, that helps a lot. Not that all people like eating fruits but if they do that to the citizens, it will help them in getting the natural healing and their body will generate every day because they eat many natural stuffs. And the naturality leads to death of unwanted bacteria, and if you add up chemo to that, you will have completed everything. [00:30:39-6](http://localhost:2300/file=C:/Users/Hp/Desktop/OTONDE/180603_1929.mp3time=1839600)

I: Mmmh [00:30:40-0](http://localhost:2300/file=C:/Users/Hp/Desktop/OTONDE/180603_1929.mp3time=1840000)

R: You see, now things are going perfectly well uncle, but the issue of leaving someone to eat rice everyday [00:30:46-0](http://localhost:2300/file=C:/Users/Hp/Desktop/OTONDE/180603_1929.mp3time=1846000)

I: Then she gets the medication, and because the medications are very strong [00:30:50-5](http://localhost:2300/file=C:/Users/Hp/Desktop/OTONDE/180603_1929.mp3time=1850500)

R: Leave alone these medications, I am not saying these in a bad way but I ask you to go and sit over there, those medications are evil, they make you loose your own self, when you get the medications, within seven days, you are not you. [00:31:07-9](http://localhost:2300/file=C:/Users/Hp/Desktop/OTONDE/180603_1929.mp3time=1867900)

I: Therefore there is a need to know ... [00:31:11-3](http://localhost:2300/file=C:/Users/Hp/Desktop/OTONDE/180603_1929.mp3time=1871300)

R: What kind of things to eat [00:31:12-7](http://localhost:2300/file=C:/Users/Hp/Desktop/OTONDE/180603_1929.mp3time=1872700)

I: Other things apart from.... [00:31:14-6](http://localhost:2300/file=C:/Users/Hp/Desktop/OTONDE/180603_1929.mp3time=1874600)

R: Even those who lose their lives, for example, I had a friend who I met at -----. She did mastectomy before starting chemo. I was so happy because she told me she had her whole breast removed and I have not yet started chemo, I am scared. I told her, don’t worry because right now you are in another stage and chemo doesn’t have much effects but I asked her, “what do you normally eat?” do you take lemon juice or do you take tomatoes? “what for?” she told me, “I don’t eat any of those” Then I told her because next week I have chemo, I do not feel well, I will call you next week and I will write to you what things you should eat. [00:32:01-0](http://localhost:2300/file=C:/Users/Hp/Desktop/OTONDE/180603_1929.mp3time=1921000)

I: Mmmh [00:32:01-4](http://localhost:2300/file=C:/Users/Hp/Desktop/OTONDE/180603_1929.mp3time=1921400)

R: I called her brother and he told me that she had passed away. [00:32:04-0](http://localhost:2300/file=C:/Users/Hp/Desktop/OTONDE/180603_1929.mp3time=1924000)

I: It didn’t take long? [00:32:05-2](http://localhost:2300/file=C:/Users/Hp/Desktop/OTONDE/180603_1929.mp3time=1925200)

R: It didn’t take long; she was weak and she was I cannot say that she had blood issues because all people with cancer must have a problem with the blood but she was very pale. So I said to myself that this is the result of not knowing about things. [00:32:23-2](http://localhost:2300/file=C:/Users/Hp/Desktop/OTONDE/180603_1929.mp3time=1943200)

I: maybe in terms of educating the society, helping people to get treatment early. Do you think when we do this as doctors but we do this in collaboration with the patients who have already been through the whole process of treatment and you too say something to them.[00:32:43-3](http://localhost:2300/file=C:/Users/Hp/Desktop/OTONDE/180603_1929.mp3time=1963300)

R: Yeah, it becomes a nice case study because they see the reality, they can say, mmh, she too had cancer, and you take the previous photos of her before she fell sick, when she fell sick, the process that she was going through and then you say to them that I was this way and this way. This helps and you doctors stress on that, educate them and then you stay with them in the villages. For example, as what the NGO does, not all go to the grass root [00:33:18-1](http://localhost:2300/file=C:/Users/Hp/Desktop/OTONDE/180603_1929.mp3time=1998100)

I: Mmmh [00:33:18-5](http://localhost:2300/file=C:/Users/Hp/Desktop/OTONDE/180603_1929.mp3time=1998500)

R: You find that others take those in the NGO, and the workers are the ones going there, their boss stays back in the office. But for a day when both the boss and the employer are there and maybe they choose somebody from another place to go and show them about the farming system of watermelon and they are shown directly, they are taken for study tour but you do not take them there you bring that person to them. It will help and it will help to change people’s mindsets especially those who believe in such people, the witch doctors.

[00:33:50-5](http://localhost:2300/file=C:/Users/Hp/Desktop/OTONDE/180603_1929.mp3time=2030500)

I: Hmmm [00:33:51-1](http://localhost:2300/file=C:/Users/Hp/Desktop/OTONDE/180603_1929.mp3time=2031100)

R: We are not saying that local herbs are bad but they are good if taken in correct proportions and knowing that they are the ones you should take. Most people stick on eating soursop fruit, I mean leaves from the soursop tee but what I know is that the soursop leaves are poisonous. So, knowing which one is safe which one is poisonous [00:34:21-7](http://localhost:2300/file=C:/Users/Hp/Desktop/OTONDE/180603_1929.mp3time=2061700)

I: So, people use that mostly? [00:34:24-3](http://localhost:2300/file=C:/Users/Hp/Desktop/OTONDE/180603_1929.mp3time=2064300)

R: Most use that [00:34:25-6](http://localhost:2300/file=C:/Users/Hp/Desktop/OTONDE/180603_1929.mp3time=2065600)

I: And it doesn’t help them? [00:34:26-3](http://localhost:2300/file=C:/Users/Hp/Desktop/OTONDE/180603_1929.mp3time=2066300)

R: But me I don’t know if it's helping them or not, and I was also told about that but I did my research on the soursop fruits and there are lots of variety, now which one is safe and which one is poisonous? [00:34:38-2](http://localhost:2300/file=C:/Users/Hp/Desktop/OTONDE/180603_1929.mp3time=2078200)

I: Okay.. [00:34:38-7](http://localhost:2300/file=C:/Users/Hp/Desktop/OTONDE/180603_1929.mp3time=2078700)

R: It is hard to tell, so people must be educated. They are not bad but they should take something that is natural such as tomatoes, garlic, grapes and things that are anti-oxidants. If they get that and two ripe bananas with fresh water. When they come to get ugali they continue to pursue life. [00:35:07-7](http://localhost:2300/file=C:/Users/Hp/Desktop/OTONDE/180603_1929.mp3time=2107700)

I: Yes [00:35:08-4](http://localhost:2300/file=C:/Users/Hp/Desktop/OTONDE/180603_1929.mp3time=2108400)

R: Eee [00:35:09-1](http://localhost:2300/file=C:/Users/Hp/Desktop/OTONDE/180603_1929.mp3time=2109100)

I: Do you have anything else to add? [00:35:11-1](http://localhost:2300/file=C:/Users/Hp/Desktop/OTONDE/180603_1929.mp3time=2111100)

R: No, I don’t have any. Thank you very much. [00:35:13-7](http://localhost:2300/file=C:/Users/Hp/Desktop/OTONDE/180603_1929.mp3time=2113700)

I: thank you too [00:35:15-0](http://localhost:2300/file=C:/Users/Hp/Desktop/OTONDE/180603_1929.mp3time=2115000)

R: Eee, can you tell those people, what department you are from, what kind of research you are doing. You just stay in towns visit the village people too. Go in the villages, go to the councils and they commit you to the ward executives. You walk around with the ward executives, there are women who are on bed, stinking, and others are putting a blame on fistula.... [00:35:42-1](http://localhost:2300/file=C:/Users/Hp/Desktop/OTONDE/180603_1929.mp3time=2142100)

I: Is it the known disease? [00:35:44-6](http://localhost:2300/file=C:/Users/Hp/Desktop/OTONDE/180603_1929.mp3time=2144600)

R: yes that it is fistula, and I tell them stop your nonsense, your politics because in the company that I was working in the early ----- we were educating people on HIV and so forth, but if you see this woman she doesn’t have AIDS they tell you she has fistula. [00:36:07-3](http://localhost:2300/file=C:/Users/Hp/Desktop/OTONDE/180603_1929.mp3time=2167300)

I: While it is something else [00:36:09-8](http://localhost:2300/file=C:/Users/Hp/Desktop/OTONDE/180603_1929.mp3time=2169800)

R: While she has cancer, we have come out with many people from the villages and brought them to the hospital. Until another company had to be responsible for the cost because it was owned by the whites, and later on the company died and people went on separate ways. so, there are some of the women who recovered through the association and what are the challenges that we found, what challenges do those people face because we began looking for the challenges maybe it is with the poor irrigation system why don’t mothers reproduce, others have fistula, and a lot of women have fistula but in real sense that is not fistula, they just don’t know that it is other diseases [00:36:51-5](http://localhost:2300/file=C:/Users/Hp/Desktop/OTONDE/180603_1929.mp3time=2211500)

I: Thank you -----. [00:36:56-4](http://localhost:2300/file=C:/Users/Hp/Desktop/OTONDE/180603_1929.mp3time=2216400)

R: yes do some research to those in the interior, they do not know about things and they will be the ones to tell you more, I just told you in brief, they are the ones who will tell you, those who will cry will cry and they will tell you that it is too painful. Go and record and do some documentaries about it. [00:37:16-7](http://localhost:2300/file=C:/Users/Hp/Desktop/OTONDE/180603_1929.mp3time=2236700)

I: Okay [00:37:17-4](http://localhost:2300/file=C:/Users/Hp/Desktop/OTONDE/180603_1929.mp3time=2237400)

R: yes, you will help them and it will help you in your researches, you will get funds form outside to do the research. [00:37:23-4](http://localhost:2300/file=C:/Users/Hp/Desktop/OTONDE/180603_1929.mp3time=2243400)

I: Okay [00:37:28-2](http://localhost:2300/file=C:/Users/Hp/Desktop/OTONDE/180603_1929.mp3time=2248200)

**CASE 7**

I: What is your name mam? 00: 00: 04-1

R: I'm -----. 00: 00: 07-0

I: And where do you come from? 00: 00: 08-3

R: I come from -----. 00: 00: 10-1

I: In what region is -----? 00: 00: 12-9

R: -----. 00: 00: 13-9

I: Are you married? 00: 00: 17-5 00: 00: 21-0

R: Yeah, I'm married. 00: 00: 19-3

I: How many children do you have? 00: 00: 20-1

R: Two children. 00: 00: 21-7

I: And what about your highest education level, how far have you studied? 00: 00: 27-1

R: I studied until seventh grade, after which I progressed to form four; did the exam but I failed. 00: 00: 36-7

I: Okay, so what work do you do now? 00: 00: 40-5

R: I'm retired; I used to work in the court. 00: 00: 45-7

I: At the court; who were you working as? 00: 00: 47-5

R: I was hired as a secretary; an office assistant. 00: 00: 53-4

I: All right, you have told me that you live in -----, what’s the distance from where you live to a health centre, whether you walk or use a car? 00: 01: 05-9

R: It is not too far, from my home to the district hospital. 00: 01: 12-4

I: Okay, so do you ride a car or just walk on foot? 00: 01: 16-5

R: I can go with a pedicab but for a person who is healthy they can just go on foot. 00: 01: 23-2

I: And what about traveling from home to -----, what transport have you used? 00: 01: 31-8

R: I used a passenger’s bus from home to here. 00: 01: 37-8

I: I want to know in what way do you know about breast cancer? 00: 01: 52-1

R: What happened is, I suddenly noticed that I have a swelling after palpating myself, there was a lump that I was wondering about it, I then called my daughter who is a nurse working in -----; I asked her about the lump in my breast. I also told my other daughter who is also a nurse but working in Iringa, she advised to go to hospital then I went to ----- hospital. I saw a doctor at ----- hospital who referred me to another doctor, the second doctor told me that was breast cancer and we have to get rid of it soon, that was around -----. 00: 02: 43-1

I: March; this year? 00: 02: 45-0

R: This year 00: 02: 45-2

I: And how long has it been since was that since you saw those symptoms? 00: 02: 53-5

R: I think it had been a month or two, I went to the hospital after seeing that the swelling was worsening, the doctor told me that I’ll have to undergo surgery where they will take a sample and send it for further investigations. I was admitted in the hospital and the operation was done, after taking out the sample I’m not sure whether they sent it to -----. 00: 03: 24-5

I: Didn’t they tell you where they sent it? 00: 03: 27-1

R: Hmm, they told me to leave the phone number that they would call me after the results came out but they didn't call me, later in noticed that the incision site was becoming hard, I first thought maybe it was because of the sutures but I doubted and went back to hospital around July for that and for the follow up of my results. 00: 03: 53-5

I: In which month was the surgery? 00: 03: 55-9

R: March 00: 03: 57-1

I: So you went back in July? 00: 03: 59-8

R: Yeah, when I got there he said to me, since the swelling has recurred then we have to operate on you again, I had to consult my family and my children stopped me. I first asked on how the results were and they told me that the results show the signs of cancer. 00: 04: 21-9

I: So the results came out after how long? 00: 04: 24-7

R: I don't know because he told me only after going to ask for them. 00: 04: 28-5

I: After going for the second time? 00: 04: 29-4

R: Hmm, but they still haven't phoned me, on that day he arranged for me to be admitted again for surgery, when we came home and consulted my children, they said that I should not have another surgery. Why should he operate on you again if the signs he sees are really suggestive of cancer? You should go and ask him to give you the results and go with them to -----. I actually went again the next day and told him that I am not ready to undergo surgery, I asked for the results to go with them to ----- which he printed and gave them to me. It was on Tuesday, on Wednesday of ----- I got on the bus and came here, and on the morning of Thursday I came to see the doctors who started to process the tests on me. 00: 05: 31-0

I: At -----? 00: 05: 32-1

R: Yes, I met doctor ----- who examined me and said, “why did this doctor hide you from telling you that it was cancer?” I told him I don't know, I'm not a professional. It was cancer, he told me there are three options of treatment, he told me about all of them but concluded that it will all depend on how the test results will come out to be. If we see the possibility of just removing the affected part ...... what shall we do ...... but if from the test results, we find that most of the breast has been affect we’ll remove all of it. 00: 06: 12-8

I: All right. 00: 06: 13-8

R: So we took the tests, they wrote to me to go to -----, I went for a test and a CD was returned, I came here on 13^th^, I was told to meet the anaesthesiologists ready for the operation, I was then told to come back here on the 18^th^ to be admitted for the operation. 00: 06: 44-1

I: All right, what did you say were the first symptoms that you noticed? 00: 06: 51-7

R: There was nothing else besides the swelling. 00: 06: 56-0

I: And you said you took two months until you went to the hospital, why did you wait for the two months maybe? 00: 07: 04-2

R: Firstly, I didn't understand what it was, I didn't understand it without the kids telling me to go to the hospital! I was just asking myself why was the area hard however there was no pain, only later did I decide to get to the hospital. 00: 07: 21-3

I: And had you ever heard anything about breast cancer? 00: 07: 27-0

R: There are times when I hear the ads, they broadcast on radio 00: 07: 32-2

I: On the radio, haven’t you ever seen other services for example -----? 00: 07: 36-7

R: I've never. 00: 07: 37-6

I: You have never, and you also told me you were always coming back and consulting your family, do you think they were deciding whether or not you should be treated? 00: 07: 54-2

R: Well, they were insisting me to come to the hospital. 00: 07: 58-8

I: All right 00: 08: 00-0

 R: And they were the ones who were encouraging me, that even if your breast will be removed it will be safer than staying with such stuff. 00: 08: 09-5

I: And what tests were done on you while you were there? 00: 08: 14-4

R: Where, in -----? The only tests there were just preparing me for surgery, just a blood tests and maybe HIV testing. 00: 08: 31-0

 I: Did you do a test for this, for example ultrasound? 00: 08: 34-0

 R: They did, they tested me. 00: 08: 35-3

 I: Where? 00: 08: 37-3

 R: At -----, they tested me. 00: 08: 39-2

 I: Ultrasound? 00: 08: 40-6

 R: Yes. 00: 08: 41-6

 I: And mammography, have you ever done it? as another imaging investigation? 00: 08: 46-2

 R: What is it? 00: 08: 48-3

 I: Mammography 00: 08: 52-1

 R: I am not sure if it is the one they did for me, mammogram 00: 09: 02-5

 I: So you told me you did an ultrasound at -----, and other tests here? 00: 09: 07-5

R: I have done a lot of tests here, the ultrasound, they did another test to check on the heart, they did a test on my abdomen, they did an x-ray 00: 09: 29-8

 I: At ----- they did the ultrasound.00: 09: 32-4

 R: Yes, they did it. 00: 09: 34-5

 I: Did they do it in the first two months or? 00: 09: 35-0

 R: Initially, after I went on that day I was told to go for it, I got tested, the results were sent to the doctor and they decided to operate on me. 00: 09: 57-3.

 I: Do you feel a little late for health care or were you on time? 00: 09: 59-3

 R: I can't tell if I was late or early, because I was just walking around without knowing what the thing really was, so I was just doing it on my own. I said let me go to the hospital and ask what this is, if I see something I don’t understand I just go to the hospital. 00: 10: 18-2

 I: And there are treatments that we see, we hear about; alternative treatments, did you undergo any other services before you came for hospital services? 00: 10: 30-5

 R: Concerning this breast? 00: 10: 30-9

 I: Hmm! 00: 10: 32-3

 R: No, I haven't done any alternative therapy, it's only hospital. 00: 10: 41-5

 I: Have you ever used traditional medicines? 00: 10: 44-2

 R: For this breast? 00: 10: 46-7

 I: Hmm! 00: 10: 47-4

 R: No. 00: 10: 49-0

 I: Don't you believe in traditional medicine? 00: 10: 50-5

 R: I've never had someone who told me if the ones for treatment of cancer are there. The reason is that I only came to confirm that it was cancer lately; at the end of July. I was scared when I was told that there were signs of cancer, I saw it was better if I come here and in case of treatment I should get them from here as well. 00:00 to 23:23. 9

 I: And have you ever had any relative who had cancer 00: 11: 27-8

 R: I've never heard of it in a family 00: 11: 36-2

 I: What are some of the things that have prompted you to start hospital therapy instead of going to other treatments, what do you think has motivated to pursue hospital care? 00: 11: 45-7

 R: Because there are people who have told me, there are my relatives who know the people who have had breast cancer, they told me to better come to the hospital than letting it spread which will make it a problem. And if you make it early and they remove the breast, there are people living without breasts, their breasts have been removed and they are active. So through their words, I was encouraged. I thought it was better to come earlier to the hospital, get treated and recover than staying with the problem making it worse. Because we lost one of our relative last year as he had cancer as well, they used to say that he had gastric ulcers, but it was cancer 00: 12: 29-9.

 I: Was it breast cancer? 00: 12: 30-6

R: I think it was gastric cancer, they diagnosed it when he was to -----, it had already spread and they could not help him, he was brought back and died later on. 00: 12: 39-8

 I: And when you were diagnosed with cancer, did the doctor tell you what stage was it? Did they tell you the stage of the cancer? 00: 12: 49-5

 R: I don't know; I don't remember if he told me 00: 12: 53-7

 I: Personally; when they told you that you would have surgery, did you not get scared? 00: 13: 01-8

 R: As a human being, fear is a must, you first ask yourself many questions. Because it is not my first time to undergo surgery, so I must be scared because I’ve once been there. For instance, I have had two surgeries for the same thing in this year example this year. The first time they did it they said they took out a sample for investigation, this is the second time; they completely removed the breast. In human nature, fear is there but you just be strong because it's the cure. 00: 13: 36-1

 I: And would there be no relatives who would stigmatize you because you have cancer maybe? 00: 13: 43-0

R: The relatives are the ones helping me, my kids. They were the ones who were putting pressure on me, to be treated early, so that if I could recover I should do so because they still need me 00: 13: 56-6

 I: You said that one of your relatives is a nurse? 00: 13: 59-4

 R: My daughter 00: 14: 01-3

 I: Your daughter, do you feel that it has contributed a lot because she was understanding? 00: 14: 05-9

R: Yeah it has also helped because we have three daughters who are all nurses so they all stood up to advise me and encourage me that this is something normal and if I get treated early I will still live 00: 14: 20-5

I: What about the economic issue in pursuing your treatment? 00: 14: 24-7

R: The economic situation is known to be difficult, we have been trying hard, what has helped us most is the health insurance. 00: 14: 34-4.

 I: Do you have health insurance? 00: 14: 35-5

 R: Hmm I do. 00: 14: 35-7

 I: In all that sequence; in terms of the hospitals, are there any challenges that you have gone through that you think have delayed you in one way or another to start treatment, to achieve treatment? 00: 14: 59-0

 R: I am really in awe of God, firstly, I have not taken long. I came here on the 30th day, I started the treatment on the following day and the treatment was good enough that I was not delayed to get this service. It is just the schedule because a lot of patients get admitted as well, they scheduled me as usual 00: 15: 24-1

 I: Is that at -----? 00: 15: 25-6

 R: Yes, and on the day I was scheduled I would find the services were as we were directed until I was admitted, it is not like they have bothered around that much. 00: 15: 36-8

 I: And what about the fact that since you have been sick from ----- to -----, I feel like a long time has passed from March and it is now August? 00: 15: 51-0

 R: You mean what happened until I reached this point? 00: 15: 53-6

 I: Hmm! 00: 15: 53-9

R: I am now not sure of the language I should use because when a doctor tells the patient to leave the phone number, we will let you know when we get the results and they do monitor their activities every day on who should get what service. Because I was just staying at home knowing that the results are not yet out but in reality they already were and I was not phoned until when I had to follow them myself. 00: 16: 21-7

I: And how far is it from where you live? 00: 16: 24-3

 R: From ----- to ----- is as far as 25 miles. 00: 16: 28-8

 I: 25 miles? 00: 16: 30-2

 R: Hmm. 00: 16: 30-7

I: So do think that you could track them faster if the place was closer? 00: 16: 37-5

 R: Hmm, and I was confident that they would be careful to follow up on my results. If they had told me to do it myself I wouldn’t have been late because when they give me a date to come back to hospital, I usually do so. I would have done it myself if they had told me to do a follow up on them. 00: 16: 55-0

 I: All right, and before you personally got this problem did you ever hear someone else or a close friend or relative had breast cancer? 00: 17: 12-2

R: Hmm No! 00: 17: 14-5

 I: You never did? 00: 17: 15-3

 R: To someone I know, I've never heard of it but I only got information from the people who were comforting me, they were just telling about different people who had undergone the same situation and they are ok but I’ve never seen my close relative having been removed her breast. 00: 17: 35-9

 I: Are people you were told that they were not treated and their outcome was not good, are there such people? 00: 17: 45-0

R: Hmm! 00: 17: 45-6

I: Maybe those who died for lack of treatment? 00: 17: 49-1

 R: Breast cancer? 00: 17: 51-8

 I: Hmm? 00: 17: 51-8

R: I haven’t heard 00: 17: 53-5

I: And among the others, did all of them get health care or are there others? 00: 17: 59-0

 R: The ones I was told about got the hospital services, getting their breasts removed in the hospital. 00: 18: 05-5

 I: All right 00: 18: 05-8

R: Yes 00: 18: 07-1

I: Well mom I'm sorry, we just wanted to know the sequence until someone arrives too late at the hospital, in your opinion, are there some others you know who come with their cancer already spread, where do you think the problem lies? 00: 18: 29-7

 R: The problem is the knowledge, not being aware, not understanding something. You know for a rare disease may see someone with a swollen finger in the first day and start questioning what it is and if it has no pain you might just ignore it. I was shocked that the swelling was there whenever I palpated myself, I was usually telling my husband and later decided to go to hospital. 00: 19: 01-0

 I: What did your husband do, did he say anything about this issue? 00: 19: 05-8

R: Hmm, he was just wondering as I was then after questioning ourselves he said we should go to hospital, so he took me. 00: 19: 14-6

 I: In terms of relatives in your clan, were they offering good support? 00: 19: 22-0

 R: Yeah, especially the kids; they are the ones who have stood on this, they even me connected me with the doctors because they know them. They introduce me as their mother, they said, take her to the hospital urgently and I received urgent care 00: 19: 42-2

 I: Okay, what are some of the things that you personally would advise the mothers you have heard that they have tested for cancer but have delayed to go to the hospital, what do you think are the causes and maybe what is your opinion? 00: 19: 59-3

R: The first thing is that a person my get a fever that they don’t know about, end up wondering what it is until they get delayed at is, the second thing might be the economy.00: 20: 13-0

 A: Economy? 00: 20: 14-6

 R: And financially as well, it would have been hard for me too if my children and relatives did not contribute. I am just thankful that my relatives contributed and my husband also had a little reservoir. Even if you use health insurance you might not get food. Well, where we have come to stay is far; ----- as it is where my sister lives. I have been moving from ----- to here and to -----, there are times that we had to hire a car so that we can make it early to do the tests. It's expensive, I've spent about eight hundred thousand since I came here. 00: 21: 02-8

 I: All that despite the fact that you have a health insurance? 00: 21: 05-9

 R: Yeah, that's just my own consumption of food, I've spent about eight hundred thousand and I don't know how long I will stay until I complete the treatment because when I get discharged home today I have to come back for the clinic. I do not know when they will discharge me, I have not been told what is going on. And as you know I still need food supplies, transportation as after were discharged home we will have to hire a car up to ----- 00: 21: 40-8

 I: -----. 00: 21: 41-7

R: We cannot take the public transport; people are suffering because of all those expenses. Someone may be wishing to go to the hospital but he is helpless, so he ends up suffering until death strikes him. 00: 21: 58-9

I: All right. 00: 21: 59-8

 R: Most families live in poverty, difficult life 00: 22: 04-8

 I: But are there no other treatments people are getting, because there are people who may see a small swelling and it keeps growing but maybe they get other treatments until they delay to come to the hospital? 00: 22: 22-8

 R: Ah! There are others who are using these alternative remedies as they are being advertised, do you usually hear them advertising? 00: 22: 31-9

I: On the radio? 00: 22: 32-7

 R: The ones who sell usually advertise that their medicines can treat cancer, others say that soursop’s leaves are used to treat all types of cancers. 00: 22: 47-7.

I: You personally couldn't? 00: 22: 50-0

R: I have never drunk. 00: 22: 51-2

 I: You have never tried? 00: 22: 52-3

R: Hmm. 00: 22: 52-5

I: Why do you personally think so? 00: 22: 55-9

 R: I am often in disbelief, I first go to the hospital and when I know that the hospital has failed to do anything about it that is when I can turn to the other side. 00: 23: 08-8

I: Well, mom, thank you, that is all from me. I’ve learnt a lot from you, do you have something else that you want to add maybe? 00: 23: 23-7

 R: Hmm, you are the ones who should try to educate people so that they can know about this or if they see any swelling they should not just stay at home but go to the hospital without starting local medications. 00: 23: 37-9

I: But we are seeing these services broadcasted on the radio, phones and on televisions. 00: 23: 46-0

 R: One can stay at home all day without watching the TV or even listening to the news, people are just out there struggling with the hardship of life, one can tell you that he hasn’t even sat in the sitting room. 24:00: 00-2

I: Although he has a TV? 00: 24: 02-1

R: Yes, he may have a TV but is because life. Few have the opportunity to listen to. 00: 24: 10-2

I: All right, what do you think is the best way to educate these people that we thought TV and Radio were probably the best way to reach them, and if they don't what do you think would be the best ways to reach people? 00: 24: 27-1

R: Hmm, I'm not sure; you could use the speakers to announce, but even on TVs others would hear, those who are busy are missing out things but when they get a chance they do so. 00: 24: 40-5.

  I: All right, thank you ----- for your time. 00: 24: 43-8

**CASE 8**

I: What is your name [00:00:02-2](http://localhost:2300/file=C:/Users/admin/Desktop/NSAJIGWA/180826_1211_Sado.mp3time=2200)

R: ----- [00:00:03-1](http://localhost:2300/file=C:/Users/admin/Desktop/NSAJIGWA/180826_1211_Sado.mp3time=3100)

I: What year were you born? [00:00:06-3](http://localhost:2300/file=C:/Users/admin/Desktop/NSAJIGWA/180826_1211_Sado.mp3time=6300)

R: ----- [00:00:10-7](http://localhost:2300/file=C:/Users/admin/Desktop/NSAJIGWA/180826_1211_Sado.mp3time=10700)

I: Oh [00:00:15-0](http://localhost:2300/file=C:/Users/admin/Desktop/NSAJIGWA/180826_1211_Sado.mp3time=15000)

R: Yes [00:00:16-0](http://localhost:2300/file=C:/Users/admin/Desktop/NSAJIGWA/180826_1211_Sado.mp3time=16000)

I: And, where do you live [00:00:17-5](http://localhost:2300/file=C:/Users/admin/Desktop/NSAJIGWA/180826_1211_Sado.mp3time=17500)

R: I live in ----- [00:00:20-0](http://localhost:2300/file=C:/Users/admin/Desktop/NSAJIGWA/180826_1211_Sado.mp3time=20000)

I: Pardon [00:00:21-3](http://localhost:2300/file=C:/Users/admin/Desktop/NSAJIGWA/180826_1211_Sado.mp3time=21300)

R: ----- [00:00:22-3](http://localhost:2300/file=C:/Users/admin/Desktop/NSAJIGWA/180826_1211_Sado.mp3time=22300)

I: ----- in ----- [00:00:23-4](http://localhost:2300/file=C:/Users/admin/Desktop/NSAJIGWA/180826_1211_Sado.mp3time=23400)

R: Yes, ----- District [00:00:24-7](http://localhost:2300/file=C:/Users/admin/Desktop/NSAJIGWA/180826_1211_Sado.mp3time=24700)

I: How many children do you have [00:00:26-7](http://localhost:2300/file=C:/Users/admin/Desktop/NSAJIGWA/180826_1211_Sado.mp3time=26700)

R: I have five children [00:00:29-3](http://localhost:2300/file=C:/Users/admin/Desktop/NSAJIGWA/180826_1211_Sado.mp3time=29300)

I: Are you married, do you live with your husband [00:00:30-4](http://localhost:2300/file=C:/Users/admin/Desktop/NSAJIGWA/180826_1211_Sado.mp3time=30400)

R: Yes, but my husband is deceased [00:00:34-8](http://localhost:2300/file=C:/Users/admin/Desktop/NSAJIGWA/180826_1211_Sado.mp3time=34800)

I: Oh sorry. What education level have you attained [00:00:38-0](http://localhost:2300/file=C:/Users/admin/Desktop/NSAJIGWA/180826_1211_Sado.mp3time=38000)

R: I have not gone to school [00:00:41-1](http://localhost:2300/file=C:/Users/admin/Desktop/NSAJIGWA/180826_1211_Sado.mp3time=41100)

I: You haven’t gone to school… [00:00:42-8](http://localhost:2300/file=C:/Users/admin/Desktop/NSAJIGWA/180826_1211_Sado.mp3time=42800)

R: Yes [00:00:43-1](http://localhost:2300/file=C:/Users/admin/Desktop/NSAJIGWA/180826_1211_Sado.mp3time=43100)

I: What is your occupation? [00:00:43-7](http://localhost:2300/file=C:/Users/admin/Desktop/NSAJIGWA/180826_1211_Sado.mp3time=43700)

R: I am just a farmer [00:00:45-7](http://localhost:2300/file=C:/Users/admin/Desktop/NSAJIGWA/180826_1211_Sado.mp3time=45700)

I: Okay, and what is the distance from where you live at ----- to a nearest health centre [00:00:53-8](http://localhost:2300/file=C:/Users/admin/Desktop/NSAJIGWA/180826_1211_Sado.mp3time=53800)

R: Distance? [00:01:04-4](http://localhost:2300/file=C:/Users/admin/Desktop/NSAJIGWA/180826_1211_Sado.mp3time=64400)

I: Yes [00:01:05-8](http://localhost:2300/file=C:/Users/admin/Desktop/NSAJIGWA/180826_1211_Sado.mp3time=65800)

R: I am only seated [00:01:06-4](http://localhost:2300/file=C:/Users/admin/Desktop/NSAJIGWA/180826_1211_Sado.mp3time=66400)

I: From your home to hospital, do you go on foot or board a car [00:01:09-8](http://localhost:2300/file=C:/Users/admin/Desktop/NSAJIGWA/180826_1211_Sado.mp3time=69800)

R: Coming to this place? [00:01:16-4](http://localhost:2300/file=C:/Users/admin/Desktop/NSAJIGWA/180826_1211_Sado.mp3time=76400)

I: Going to any hospital nearby your home [00:01:17-7](http://localhost:2300/file=C:/Users/admin/Desktop/NSAJIGWA/180826_1211_Sado.mp3time=77700)

R: I go on foot [00:01:20-6](http://localhost:2300/file=C:/Users/admin/Desktop/NSAJIGWA/180826_1211_Sado.mp3time=80600)

I: What hospital is nearby your place [00:01:21-7](http://localhost:2300/file=C:/Users/admin/Desktop/NSAJIGWA/180826_1211_Sado.mp3time=81700)

R: The one of ----- District [00:01:24-2](http://localhost:2300/file=C:/Users/admin/Desktop/NSAJIGWA/180826_1211_Sado.mp3time=84200)

I: Do you walk to reach there [00:01:32-5](http://localhost:2300/file=C:/Users/admin/Desktop/NSAJIGWA/180826_1211_Sado.mp3time=92500)

R: Yes, or I can board a bus , ----- [00:01:34-1](http://localhost:2300/file=C:/Users/admin/Desktop/NSAJIGWA/180826_1211_Sado.mp3time=94100)

I: Oh [00:01:36-2](http://localhost:2300/file=C:/Users/admin/Desktop/NSAJIGWA/180826_1211_Sado.mp3time=96200)

R: Yes, [00:01:36-8](http://localhost:2300/file=C:/Users/admin/Desktop/NSAJIGWA/180826_1211_Sado.mp3time=96800)

I: How much does it cost when by bus [00:01:38-9](http://localhost:2300/file=C:/Users/admin/Desktop/NSAJIGWA/180826_1211_Sado.mp3time=98900)

R: Tshs2500 [00:01:41-6](http://localhost:2300/file=C:/Users/admin/Desktop/NSAJIGWA/180826_1211_Sado.mp3time=101600)

I: Mmh, [00:01:42-7](http://localhost:2300/file=C:/Users/admin/Desktop/NSAJIGWA/180826_1211_Sado.mp3time=102700)

R: From ----- [00:01:43-5](http://localhost:2300/file=C:/Users/admin/Desktop/NSAJIGWA/180826_1211_Sado.mp3time=103500)

I: And from there to -----, what means of transport did you use? [00:01:50-6](http://localhost:2300/file=C:/Users/admin/Desktop/NSAJIGWA/180826_1211_Sado.mp3time=110600)

R: I came by bus that come from ----- [00:01:52-2](http://localhost:2300/file=C:/Users/admin/Desktop/NSAJIGWA/180826_1211_Sado.mp3time=112200)

I: Long distance trucks from -----? [00:01:55-9](http://localhost:2300/file=C:/Users/admin/Desktop/NSAJIGWA/180826_1211_Sado.mp3time=115900)

R: Yes [00:01:56-8](http://localhost:2300/file=C:/Users/admin/Desktop/NSAJIGWA/180826_1211_Sado.mp3time=116800)

I: So you boarded a lorry and not a bus [00:01:58-0](http://localhost:2300/file=C:/Users/admin/Desktop/NSAJIGWA/180826_1211_Sado.mp3time=118000)

R: Yes, long distance trucks [00:02:00-1](http://localhost:2300/file=C:/Users/admin/Desktop/NSAJIGWA/180826_1211_Sado.mp3time=120100)

I: Oh [00:02:02-2](http://localhost:2300/file=C:/Users/admin/Desktop/NSAJIGWA/180826_1211_Sado.mp3time=122200)

R: Yes [00:02:02-9](http://localhost:2300/file=C:/Users/admin/Desktop/NSAJIGWA/180826_1211_Sado.mp3time=122900)

I: Okay [00:02:03-3](http://localhost:2300/file=C:/Users/admin/Desktop/NSAJIGWA/180826_1211_Sado.mp3time=123300)

I: So can you tell to me how you started falling sick till today that you are here [00:02:09-3](http://localhost:2300/file=C:/Users/admin/Desktop/NSAJIGWA/180826_1211_Sado.mp3time=129300)

R: It started last year October but this is the second time, the first my breast was swollen then came I to -----, got treated and was transferred to ----- for chemotherapy, I used the cycles and completed then returned back home [00:02:18-1](http://localhost:2300/file=C:/Users/admin/Desktop/NSAJIGWA/180826_1211_Sado.mp3time=138100)

I: What year was that [00:02:44-3](http://localhost:2300/file=C:/Users/admin/Desktop/NSAJIGWA/180826_1211_Sado.mp3time=164300)

R: ----- [00:02:45-6](http://localhost:2300/file=C:/Users/admin/Desktop/NSAJIGWA/180826_1211_Sado.mp3time=165600)

I: -----? [00:02:46-6](http://localhost:2300/file=C:/Users/admin/Desktop/NSAJIGWA/180826_1211_Sado.mp3time=166600)

R: Yes [00:02:47-0](http://localhost:2300/file=C:/Users/admin/Desktop/NSAJIGWA/180826_1211_Sado.mp3time=167000)

I: Is that when it was swollen for the first time [00:02:48-6](http://localhost:2300/file=C:/Users/admin/Desktop/NSAJIGWA/180826_1211_Sado.mp3time=168600)

R: Yes [00:02:52-3](http://localhost:2300/file=C:/Users/admin/Desktop/NSAJIGWA/180826_1211_Sado.mp3time=172300)

I: Before starting treatment what symptoms did you see [00:02:52-9](http://localhost:2300/file=C:/Users/admin/Desktop/NSAJIGWA/180826_1211_Sado.mp3time=172900)

R: For last year or [00:02:58-7](http://localhost:2300/file=C:/Users/admin/Desktop/NSAJIGWA/180826_1211_Sado.mp3time=178700)

I: No, for the year that you first saw the swelling [00:03:02-3](http://localhost:2300/file=C:/Users/admin/Desktop/NSAJIGWA/180826_1211_Sado.mp3time=182300)

R: Can you pardon because I have problems with swahili [00:03:08-4](http://localhost:2300/file=C:/Users/admin/Desktop/NSAJIGWA/180826_1211_Sado.mp3time=188400)

I: What did you first see on the breast that you decided to go to hospital [00:03:14-0](http://localhost:2300/file=C:/Users/admin/Desktop/NSAJIGWA/180826_1211_Sado.mp3time=194000)

R: There was a small swelling inside the breast and once you hold it was too small, I was given local herbs that once I urinate the swelling would go but there was no response so I went to hospital and I was told that it is cancer [00:03:19-7](http://localhost:2300/file=C:/Users/admin/Desktop/NSAJIGWA/180826_1211_Sado.mp3time=199700)

I: So with the herbs that you were given they told you that you will urinate the disease out? [00:04:03-8](http://localhost:2300/file=C:/Users/admin/Desktop/NSAJIGWA/180826_1211_Sado.mp3time=243800)

R: Yes [00:04:09-4](http://localhost:2300/file=C:/Users/admin/Desktop/NSAJIGWA/180826_1211_Sado.mp3time=249400)

I: Where do they place the local herbs [00:04:10-9](http://localhost:2300/file=C:/Users/admin/Desktop/NSAJIGWA/180826_1211_Sado.mp3time=250900)

R: Pardon [00:04:11-6](http://localhost:2300/file=C:/Users/admin/Desktop/NSAJIGWA/180826_1211_Sado.mp3time=251600)

I: Do they give you to drink or [00:04:12-2](http://localhost:2300/file=C:/Users/admin/Desktop/NSAJIGWA/180826_1211_Sado.mp3time=252200)

R: I drink [00:04:15-2](http://localhost:2300/file=C:/Users/admin/Desktop/NSAJIGWA/180826_1211_Sado.mp3time=255200)

I: The local herbs [00:04:16-5](http://localhost:2300/file=C:/Users/admin/Desktop/NSAJIGWA/180826_1211_Sado.mp3time=256500)

R: Yes [00:04:17-0](http://localhost:2300/file=C:/Users/admin/Desktop/NSAJIGWA/180826_1211_Sado.mp3time=257000)

I: Who gave them to you [00:04:17-7](http://localhost:2300/file=C:/Users/admin/Desktop/NSAJIGWA/180826_1211_Sado.mp3time=257700)

R: Witch Doctor [00:04:18-9](http://localhost:2300/file=C:/Users/admin/Desktop/NSAJIGWA/180826_1211_Sado.mp3time=258900)

I: Okay, so for how long where you treated him [00:04:25-4](http://localhost:2300/file=C:/Users/admin/Desktop/NSAJIGWA/180826_1211_Sado.mp3time=265400)

R: The first time when I noticed the swelling, I stopped using the local herbs and I went to hospital, I was told that its cancer and I should go to ----- for treatment. That’s when I came to ----- in year ----- [00:04:30-0](http://localhost:2300/file=C:/Users/admin/Desktop/NSAJIGWA/180826_1211_Sado.mp3time=270000)

I: Oh [00:04:52-3](http://localhost:2300/file=C:/Users/admin/Desktop/NSAJIGWA/180826_1211_Sado.mp3time=292300)

R: Yes [00:04:53-2](http://localhost:2300/file=C:/Users/admin/Desktop/NSAJIGWA/180826_1211_Sado.mp3time=293200)

I: So the first care provider you had visited a witch doctor [00:04:53-9](http://localhost:2300/file=C:/Users/admin/Desktop/NSAJIGWA/180826_1211_Sado.mp3time=293900)

R: Yes [00:05:04-7](http://localhost:2300/file=C:/Users/admin/Desktop/NSAJIGWA/180826_1211_Sado.mp3time=304700)

I; And for how long did he treat you [00:05:04-8](http://localhost:2300/file=C:/Users/admin/Desktop/NSAJIGWA/180826_1211_Sado.mp3time=304800)

R: Just a short while [00:05:06-8](http://localhost:2300/file=C:/Users/admin/Desktop/NSAJIGWA/180826_1211_Sado.mp3time=306800)

I: And did you get better [00:05:10-2](http://localhost:2300/file=C:/Users/admin/Desktop/NSAJIGWA/180826_1211_Sado.mp3time=310200)

R: No [00:05:11-3](http://localhost:2300/file=C:/Users/admin/Desktop/NSAJIGWA/180826_1211_Sado.mp3time=311300)

I: Were you paying money [00:05:12-4](http://localhost:2300/file=C:/Users/admin/Desktop/NSAJIGWA/180826_1211_Sado.mp3time=312400)

R: No, it was for free and he was saying ‘let me try to treat you’ so he tried then he stopped after treatment failure then I also stopped and proceeded with hospital treatment [00:05:14-6](http://localhost:2300/file=C:/Users/admin/Desktop/NSAJIGWA/180826_1211_Sado.mp3time=314600)

I: Was he your relative [00:05:25-8](http://localhost:2300/file=C:/Users/admin/Desktop/NSAJIGWA/180826_1211_Sado.mp3time=325800)

R: No [00:05:27-1](http://localhost:2300/file=C:/Users/admin/Desktop/NSAJIGWA/180826_1211_Sado.mp3time=327100)

R: He only knew me, he is a neighbor who knows about local herbs [00:05:27-9](http://localhost:2300/file=C:/Users/admin/Desktop/NSAJIGWA/180826_1211_Sado.mp3time=327900)

I: Do you know anything about breast cancer [00:05:33-5](http://localhost:2300/file=C:/Users/admin/Desktop/NSAJIGWA/180826_1211_Sado.mp3time=333500)

R: Yes [00:05:37-9](http://localhost:2300/file=C:/Users/admin/Desktop/NSAJIGWA/180826_1211_Sado.mp3time=337900)

I: What do you know about breast cancer [00:05:38-4](http://localhost:2300/file=C:/Users/admin/Desktop/NSAJIGWA/180826_1211_Sado.mp3time=338400)

R; I don’t really know, I only see it [00:05:42-1](http://localhost:2300/file=C:/Users/admin/Desktop/NSAJIGWA/180826_1211_Sado.mp3time=342100)

I: you didn’t know about it? [00:05:43-4](http://localhost:2300/file=C:/Users/admin/Desktop/NSAJIGWA/180826_1211_Sado.mp3time=343400)

R:I only see it [00:05:44-6](http://localhost:2300/file=C:/Users/admin/Desktop/NSAJIGWA/180826_1211_Sado.mp3time=344600)

I: Where have you seen it? [00:05:45-9](http://localhost:2300/file=C:/Users/admin/Desktop/NSAJIGWA/180826_1211_Sado.mp3time=345900)

R: I have seen it after being ill [00:05:47-3](http://localhost:2300/file=C:/Users/admin/Desktop/NSAJIGWA/180826_1211_Sado.mp3time=347300)

I: What was the response from home after you were told that you have breast cancer [00:05:52-1](http://localhost:2300/file=C:/Users/admin/Desktop/NSAJIGWA/180826_1211_Sado.mp3time=352100)

R: Pardon [00:05:58-7](http://localhost:2300/file=C:/Users/admin/Desktop/NSAJIGWA/180826_1211_Sado.mp3time=358700)

I: Concerning starting treatment [00:06:00-4](http://localhost:2300/file=C:/Users/admin/Desktop/NSAJIGWA/180826_1211_Sado.mp3time=360400)

R: You mean my parents and other relatives? [00:06:05-5](http://localhost:2300/file=C:/Users/admin/Desktop/NSAJIGWA/180826_1211_Sado.mp3time=365500)

I: Yes, what was your response? [00:06:09-9](http://localhost:2300/file=C:/Users/admin/Desktop/NSAJIGWA/180826_1211_Sado.mp3time=369900)

R: They said that they should take me to hospital [00:06:11-6](http://localhost:2300/file=C:/Users/admin/Desktop/NSAJIGWA/180826_1211_Sado.mp3time=371600)

I: Do they live here in -----? [00:06:14-7](http://localhost:2300/file=C:/Users/admin/Desktop/NSAJIGWA/180826_1211_Sado.mp3time=374700)

R: No, they live in -----, I don’t have relatives here in ----- I only came to hospital for treatment [00:06:17-0](http://localhost:2300/file=C:/Users/admin/Desktop/NSAJIGWA/180826_1211_Sado.mp3time=377000)

I: Did you come alone here at the hospital? [00:06:24-1](http://localhost:2300/file=C:/Users/admin/Desktop/NSAJIGWA/180826_1211_Sado.mp3time=384100)

R: I came with my brother [00:06:25-5](http://localhost:2300/file=C:/Users/admin/Desktop/NSAJIGWA/180826_1211_Sado.mp3time=385500)

I: Oh [00:06:26-6](http://localhost:2300/file=C:/Users/admin/Desktop/NSAJIGWA/180826_1211_Sado.mp3time=386600)

R:Yes [00:06:26-9](http://localhost:2300/file=C:/Users/admin/Desktop/NSAJIGWA/180826_1211_Sado.mp3time=386900)

I: Do you normally go to hospital for regular check-up? [00:06:30-7](http://localhost:2300/file=C:/Users/admin/Desktop/NSAJIGWA/180826_1211_Sado.mp3time=390700)

R: Yes, they told me to go to ----- to for treatment [00:06:38-8](http://localhost:2300/file=C:/Users/admin/Desktop/NSAJIGWA/180826_1211_Sado.mp3time=398800)

I; Do you also use local herbs? [00:06:58-7](http://localhost:2300/file=C:/Users/admin/Desktop/NSAJIGWA/180826_1211_Sado.mp3time=418700)

R: What medication [00:07:02-0](http://localhost:2300/file=C:/Users/admin/Desktop/NSAJIGWA/180826_1211_Sado.mp3time=422000)

I: The ones from the witch doctor? [00:07:03-3](http://localhost:2300/file=C:/Users/admin/Desktop/NSAJIGWA/180826_1211_Sado.mp3time=423300)

R; No, I stopped using them a while ago [00:07:05-2](http://localhost:2300/file=C:/Users/admin/Desktop/NSAJIGWA/180826_1211_Sado.mp3time=425200)

I: So for now you are not using? [00:07:05-5](http://localhost:2300/file=C:/Users/admin/Desktop/NSAJIGWA/180826_1211_Sado.mp3time=425500)

R: No, I stopped using them a while ago [00:07:06-9](http://localhost:2300/file=C:/Users/admin/Desktop/NSAJIGWA/180826_1211_Sado.mp3time=426900)

I: Do you have a health insurance card or do you pay cash? [00:07:15-1](http://localhost:2300/file=C:/Users/admin/Desktop/NSAJIGWA/180826_1211_Sado.mp3time=435100)

R: I do pay cash [00:07:31-8](http://localhost:2300/file=C:/Users/admin/Desktop/NSAJIGWA/180826_1211_Sado.mp3time=451800)

I: Have you done any investigation like ultrasound or mammograph? [00:07:52-6](http://localhost:2300/file=C:/Users/admin/Desktop/NSAJIGWA/180826_1211_Sado.mp3time=472600)

R: Chest X-ray [00:08:03-0](http://localhost:2300/file=C:/Users/admin/Desktop/NSAJIGWA/180826_1211_Sado.mp3time=483000)

I:Yes [00:08:03-7](http://localhost:2300/file=C:/Users/admin/Desktop/NSAJIGWA/180826_1211_Sado.mp3time=483700)

R: Today I did a chest x-ray [00:08:04-5](http://localhost:2300/file=C:/Users/admin/Desktop/NSAJIGWA/180826_1211_Sado.mp3time=484500)

I: So, what investigations did you do at -----? [00:08:07-2](http://localhost:2300/file=C:/Users/admin/Desktop/NSAJIGWA/180826_1211_Sado.mp3time=487200)

R: No investigation that was done at ----- [00:08:12-8](http://localhost:2300/file=C:/Users/admin/Desktop/NSAJIGWA/180826_1211_Sado.mp3time=492800)

I: You did not do any investigation? [00:08:14-3](http://localhost:2300/file=C:/Users/admin/Desktop/NSAJIGWA/180826_1211_Sado.mp3time=494300)

R: Yes, I didn’t [00:08:15-1](http://localhost:2300/file=C:/Users/admin/Desktop/NSAJIGWA/180826_1211_Sado.mp3time=495100)

I: So, who told you to come to Dar es Salaam from -----? [00:08:15-8](http://localhost:2300/file=C:/Users/admin/Desktop/NSAJIGWA/180826_1211_Sado.mp3time=495800)

R:A doctor from ----- [00:08:20-1](http://localhost:2300/file=C:/Users/admin/Desktop/NSAJIGWA/180826_1211_Sado.mp3time=500100)

I; Have you ever seen a person with a similar disease at your place? [00:08:59-5](http://localhost:2300/file=C:/Users/admin/Desktop/NSAJIGWA/180826_1211_Sado.mp3time=539500)

R: Breast disease [00:09:03-0](http://localhost:2300/file=C:/Users/admin/Desktop/NSAJIGWA/180826_1211_Sado.mp3time=543000)

I: Yes [00:09:04-0](http://localhost:2300/file=C:/Users/admin/Desktop/NSAJIGWA/180826_1211_Sado.mp3time=544000)

R: I have never seen it even from my relatives [00:09:05-1](http://localhost:2300/file=C:/Users/admin/Desktop/NSAJIGWA/180826_1211_Sado.mp3time=545100)

I: What about neighbors? [00:09:17-1](http://localhost:2300/file=C:/Users/admin/Desktop/NSAJIGWA/180826_1211_Sado.mp3time=557100)

R: No, I have never seen [00:09:18-6](http://localhost:2300/file=C:/Users/admin/Desktop/NSAJIGWA/180826_1211_Sado.mp3time=558600)

I: So how did the traditional healers know that they will treat you by drinking the local herbs, have they ever treated someone before or are you the first person they ever saw? [00:09:20-9](http://localhost:2300/file=C:/Users/admin/Desktop/NSAJIGWA/180826_1211_Sado.mp3time=560900)

R: They have never seen someone before [00:10:00-5](http://localhost:2300/file=C:/Users/admin/Desktop/NSAJIGWA/180826_1211_Sado.mp3time=600500)

I: Okay, apart from traditional healers is there any other treatment regime that you are taking? [00:10:05-0](http://localhost:2300/file=C:/Users/admin/Desktop/NSAJIGWA/180826_1211_Sado.mp3time=605000)

R: No [00:10:18-5](http://localhost:2300/file=C:/Users/admin/Desktop/NSAJIGWA/180826_1211_Sado.mp3time=618500)

I: So, is it only here at the hospital? [00:10:19-4](http://localhost:2300/file=C:/Users/admin/Desktop/NSAJIGWA/180826_1211_Sado.mp3time=619400)

R: Yes [00:10:20-6](http://localhost:2300/file=C:/Users/admin/Desktop/NSAJIGWA/180826_1211_Sado.mp3time=620600)

I: So, how do you see it, are you late coming to hospital for medical help or you came early? [00:10:21-1](http://localhost:2300/file=C:/Users/admin/Desktop/NSAJIGWA/180826_1211_Sado.mp3time=621100)

R: I came early [00:10:31-6](http://localhost:2300/file=C:/Users/admin/Desktop/NSAJIGWA/180826_1211_Sado.mp3time=631600)

I: What stage did they tell you your disease was? [00:10:32-8](http://localhost:2300/file=C:/Users/admin/Desktop/NSAJIGWA/180826_1211_Sado.mp3time=632800)

R: Here at the hospital [00:10:37-1](http://localhost:2300/file=C:/Users/admin/Desktop/NSAJIGWA/180826_1211_Sado.mp3time=637100)

I:Yes [00:10:39-1](http://localhost:2300/file=C:/Users/admin/Desktop/NSAJIGWA/180826_1211_Sado.mp3time=639100)

R: I am not informed [00:10:39-7](http://localhost:2300/file=C:/Users/admin/Desktop/NSAJIGWA/180826_1211_Sado.mp3time=639700)

I: But how do you see the illness do you see it as advanced already or still at early stage? [00:10:41-6](http://localhost:2300/file=C:/Users/admin/Desktop/NSAJIGWA/180826_1211_Sado.mp3time=641600)

R: It was a small wound [00:10:47-8](http://localhost:2300/file=C:/Users/admin/Desktop/NSAJIGWA/180826_1211_Sado.mp3time=647800)

I: Oh [00:10:50-5](http://localhost:2300/file=C:/Users/admin/Desktop/NSAJIGWA/180826_1211_Sado.mp3time=650500)

R: Yes [00:10:51-3](http://localhost:2300/file=C:/Users/admin/Desktop/NSAJIGWA/180826_1211_Sado.mp3time=651300)

I: So, you were late coming to hospital? [00:10:52-1](http://localhost:2300/file=C:/Users/admin/Desktop/NSAJIGWA/180826_1211_Sado.mp3time=652100)

R: Yes, I delayed coming to hospital [00:10:53-9](http://localhost:2300/file=C:/Users/admin/Desktop/NSAJIGWA/180826_1211_Sado.mp3time=653900)

I: What are other reasons apart from attending to a traditional healer that made you delay starting medical treatment? [00:10:56-6](http://localhost:2300/file=C:/Users/admin/Desktop/NSAJIGWA/180826_1211_Sado.mp3time=656600)

R: Mmmh? [00:11:06-4](http://localhost:2300/file=C:/Users/admin/Desktop/NSAJIGWA/180826_1211_Sado.mp3time=666400)

I; What are the other reasons that made you delay? [00:11:07-1](http://localhost:2300/file=C:/Users/admin/Desktop/NSAJIGWA/180826_1211_Sado.mp3time=667100)

R: Financial crisis, money has been a problem especially this time that it didn’t rain so few was harvested and there was an option of selling cows but they are not good looking to sell due to lack of pasture so even when sold are sold at a cheaper price and this place is too far especially for two people it’s really expensive [00:11:10-4](http://localhost:2300/file=C:/Users/admin/Desktop/NSAJIGWA/180826_1211_Sado.mp3time=670400)

I: So, you came only the two of you, you and your brother? [00:11:41-4](http://localhost:2300/file=C:/Users/admin/Desktop/NSAJIGWA/180826_1211_Sado.mp3time=701400)

R: Yes [00:11:46-7](http://localhost:2300/file=C:/Users/admin/Desktop/NSAJIGWA/180826_1211_Sado.mp3time=706700)

I: So, where does he stay? [00:11:49-1](http://localhost:2300/file=C:/Users/admin/Desktop/NSAJIGWA/180826_1211_Sado.mp3time=709100)

R: He stays and sleeps at the bus stand near the hospital gate. We don’t have relatives here or money to pay for a hotel. [00:11:51-5](http://localhost:2300/file=C:/Users/admin/Desktop/NSAJIGWA/180826_1211_Sado.mp3time=711500)

I: He stays here around the hospital? [00:11:56-6](http://localhost:2300/file=C:/Users/admin/Desktop/NSAJIGWA/180826_1211_Sado.mp3time=716600)

R:Yes [00:11:58-0](http://localhost:2300/file=C:/Users/admin/Desktop/NSAJIGWA/180826_1211_Sado.mp3time=718000)

I: Is there any other challenge apart from finance? [00:12:04-0](http://localhost:2300/file=C:/Users/admin/Desktop/NSAJIGWA/180826_1211_Sado.mp3time=724000)

R: Another challenge is I want to be treated [00:12:22-9](http://localhost:2300/file=C:/Users/admin/Desktop/NSAJIGWA/180826_1211_Sado.mp3time=742900)

I: Is there any treatment pertaining breast that you received at ----- Hospital? [00:12:32-4](http://localhost:2300/file=C:/Users/admin/Desktop/NSAJIGWA/180826_1211_Sado.mp3time=752400)

R: No [00:12:40-0](http://localhost:2300/file=C:/Users/admin/Desktop/NSAJIGWA/180826_1211_Sado.mp3time=760000)

I: Why so, what did they say? [00:12:40-7](http://localhost:2300/file=C:/Users/admin/Desktop/NSAJIGWA/180826_1211_Sado.mp3time=760700)

R: They said that they can’t treat the disease [00:13:02-9](http://localhost:2300/file=C:/Users/admin/Desktop/NSAJIGWA/180826_1211_Sado.mp3time=782900)

I: Why can’t they treat it? [00:13:08-1](http://localhost:2300/file=C:/Users/admin/Desktop/NSAJIGWA/180826_1211_Sado.mp3time=788100)

R: I don’t know [00:13:10-9](http://localhost:2300/file=C:/Users/admin/Desktop/NSAJIGWA/180826_1211_Sado.mp3time=790900)

I: Are there no medical equipment or no investigations? [00:13:12-0](http://localhost:2300/file=C:/Users/admin/Desktop/NSAJIGWA/180826_1211_Sado.mp3time=792000)

R: I don’t know [00:13:14-3](http://localhost:2300/file=C:/Users/admin/Desktop/NSAJIGWA/180826_1211_Sado.mp3time=794300)

I: Okay [00:13:17-1](http://localhost:2300/file=C:/Users/admin/Desktop/NSAJIGWA/180826_1211_Sado.mp3time=797100)

I: Do your relatives stigmatize you when you tell them that you are suffering from this disease? [00:13:21-3](http://localhost:2300/file=C:/Users/admin/Desktop/NSAJIGWA/180826_1211_Sado.mp3time=801300)

R: No, they don’t stigmatize me [00:13:30-1](http://localhost:2300/file=C:/Users/admin/Desktop/NSAJIGWA/180826_1211_Sado.mp3time=810100)

I: So, what is the other challenge? [00:13:36-3](http://localhost:2300/file=C:/Users/admin/Desktop/NSAJIGWA/180826_1211_Sado.mp3time=816300)

R: Its only investigations, they need to be done investigations [00:13:39-5](http://localhost:2300/file=C:/Users/admin/Desktop/NSAJIGWA/180826_1211_Sado.mp3time=819500)

I: Weren’t you scared to undergo chemotherapy and radiotherapy at -----? [00:13:50-8](http://localhost:2300/file=C:/Users/admin/Desktop/NSAJIGWA/180826_1211_Sado.mp3time=830800)

R: No, at first I underwent chemotherapy and I completed the cycle [00:14:02-2](http://localhost:2300/file=C:/Users/admin/Desktop/NSAJIGWA/180826_1211_Sado.mp3time=842200)

I: Were you not you scared? [00:14:08-9](http://localhost:2300/file=C:/Users/admin/Desktop/NSAJIGWA/180826_1211_Sado.mp3time=848900)

R:No, [00:14:10-1](http://localhost:2300/file=C:/Users/admin/Desktop/NSAJIGWA/180826_1211_Sado.mp3time=850100)

I: At -----, were you also paying cash? [00:14:11-2](http://localhost:2300/file=C:/Users/admin/Desktop/NSAJIGWA/180826_1211_Sado.mp3time=851200)

R:No, I didn’t pay [00:14:15-9](http://localhost:2300/file=C:/Users/admin/Desktop/NSAJIGWA/180826_1211_Sado.mp3time=855900)

I: Do you know about health insurance scheme, the one that you pay and you show your card and get treated for free? [00:14:18-6](http://localhost:2300/file=C:/Users/admin/Desktop/NSAJIGWA/180826_1211_Sado.mp3time=858600)

R:No [00:14:43-6](http://localhost:2300/file=C:/Users/admin/Desktop/NSAJIGWA/180826_1211_Sado.mp3time=883600)

I: So now do you know well about this disease? [00:14:47-6](http://localhost:2300/file=C:/Users/admin/Desktop/NSAJIGWA/180826_1211_Sado.mp3time=887600)

R: No, I don’t [00:15:00-7](http://localhost:2300/file=C:/Users/admin/Desktop/NSAJIGWA/180826_1211_Sado.mp3time=900700)

I: Don’t you know about this disease? [00:15:01-9](http://localhost:2300/file=C:/Users/admin/Desktop/NSAJIGWA/180826_1211_Sado.mp3time=901900)

R: Yes I don’t [00:15:02-9](http://localhost:2300/file=C:/Users/admin/Desktop/NSAJIGWA/180826_1211_Sado.mp3time=902900)

I: If someone asks you what you are suffering from can you tell him what you are suffering from? [00:15:04-2](http://localhost:2300/file=C:/Users/admin/Desktop/NSAJIGWA/180826_1211_Sado.mp3time=904200)

R: Am suffering from breast disease [00:15:10-6](http://localhost:2300/file=C:/Users/admin/Desktop/NSAJIGWA/180826_1211_Sado.mp3time=910600)

I: Suppose there comes your relative with similar symptoms will you be able tell her what she is suffering from? Aren’t you taught about this disease [00:15:53-5](http://localhost:2300/file=C:/Users/admin/Desktop/NSAJIGWA/180826_1211_Sado.mp3time=953500)

R: Yes [00:15:55-7](http://localhost:2300/file=C:/Users/admin/Desktop/NSAJIGWA/180826_1211_Sado.mp3time=955700)

I: Okay, so do you have a television or radio at home? [00:15:59-3](http://localhost:2300/file=C:/Users/admin/Desktop/NSAJIGWA/180826_1211_Sado.mp3time=959300)

R: No, [00:16:04-5](http://localhost:2300/file=C:/Users/admin/Desktop/NSAJIGWA/180826_1211_Sado.mp3time=964500)

I: What is the work of your other relatives? [00:16:13-1](http://localhost:2300/file=C:/Users/admin/Desktop/NSAJIGWA/180826_1211_Sado.mp3time=973100)

R: They are not working [00:16:14-9](http://localhost:2300/file=C:/Users/admin/Desktop/NSAJIGWA/180826_1211_Sado.mp3time=974900)

I: You told me that you have children [00:17:04-8](http://localhost:2300/file=C:/Users/admin/Desktop/NSAJIGWA/180826_1211_Sado.mp3time=1024800)

R: Yes, I do [00:17:07-0](http://localhost:2300/file=C:/Users/admin/Desktop/NSAJIGWA/180826_1211_Sado.mp3time=1027000)

I: Do you live with your kids? [00:17:08-0](http://localhost:2300/file=C:/Users/admin/Desktop/NSAJIGWA/180826_1211_Sado.mp3time=1028000)

R: Others are married [00:17:09-9](http://localhost:2300/file=C:/Users/admin/Desktop/NSAJIGWA/180826_1211_Sado.mp3time=1029900)

I: How have they helped you to reach here? [00:17:13-1](http://localhost:2300/file=C:/Users/admin/Desktop/NSAJIGWA/180826_1211_Sado.mp3time=1033100)

R: they only contribute money [00:17:15-4](http://localhost:2300/file=C:/Users/admin/Desktop/NSAJIGWA/180826_1211_Sado.mp3time=1035400)

I: Haven’t they told you to visit a traditional healer? [00:17:18-2](http://localhost:2300/file=C:/Users/admin/Desktop/NSAJIGWA/180826_1211_Sado.mp3time=1038200)

R: No, [00:17:31-0](http://localhost:2300/file=C:/Users/admin/Desktop/NSAJIGWA/180826_1211_Sado.mp3time=1051000)

I: So, what are other reasons that can make you delay reach hospital for treatment? [00:17:40-7](http://localhost:2300/file=C:/Users/admin/Desktop/NSAJIGWA/180826_1211_Sado.mp3time=1060700)

R: It’s only finances [00:18:00-6](http://localhost:2300/file=C:/Users/admin/Desktop/NSAJIGWA/180826_1211_Sado.mp3time=1080600)

I: Can you please tell me so that I may learn about how traditional healers to treat? [00:18:23-4](http://localhost:2300/file=C:/Users/admin/Desktop/NSAJIGWA/180826_1211_Sado.mp3time=1103400)

R: He used to give me local herbs to drink so that I urinate [00:18:37-1](http://localhost:2300/file=C:/Users/admin/Desktop/NSAJIGWA/180826_1211_Sado.mp3time=1117100)

I: Did it help? [00:18:51-9](http://localhost:2300/file=C:/Users/admin/Desktop/NSAJIGWA/180826_1211_Sado.mp3time=1131900)

R: No [00:18:53-2](http://localhost:2300/file=C:/Users/admin/Desktop/NSAJIGWA/180826_1211_Sado.mp3time=1133200)

I: And did he tell you the cause of the swelling? [00:18:59-3](http://localhost:2300/file=C:/Users/admin/Desktop/NSAJIGWA/180826_1211_Sado.mp3time=1139300)

R: No, he used to only treat me [00:19:02-4](http://localhost:2300/file=C:/Users/admin/Desktop/NSAJIGWA/180826_1211_Sado.mp3time=1142400)

I: He never told you the cause of the swelling? [00:19:07-1](http://localhost:2300/file=C:/Users/admin/Desktop/NSAJIGWA/180826_1211_Sado.mp3time=1147100)

R: They say call it “Mabale” in ----- language [00:19:10-8](http://localhost:2300/file=C:/Users/admin/Desktop/NSAJIGWA/180826_1211_Sado.mp3time=1150800)

I: “mabale”? [00:19:19-7](http://localhost:2300/file=C:/Users/admin/Desktop/NSAJIGWA/180826_1211_Sado.mp3time=1159700)

R: Yes [00:19:20-2](http://localhost:2300/file=C:/Users/admin/Desktop/NSAJIGWA/180826_1211_Sado.mp3time=1160200)

I: What is Mabale? [00:19:20-7](http://localhost:2300/file=C:/Users/admin/Desktop/NSAJIGWA/180826_1211_Sado.mp3time=1160700)

R: It’s a disease [00:19:21-7](http://localhost:2300/file=C:/Users/admin/Desktop/NSAJIGWA/180826_1211_Sado.mp3time=1161700)

I: So, what else did he tell is the cause? [00:19:25-8](http://localhost:2300/file=C:/Users/admin/Desktop/NSAJIGWA/180826_1211_Sado.mp3time=1165800)

R: They only say that’s the cause [00:19:33-1](http://localhost:2300/file=C:/Users/admin/Desktop/NSAJIGWA/180826_1211_Sado.mp3time=1173100)

I: and did the herbs help you? [00:19:36-8](http://localhost:2300/file=C:/Users/admin/Desktop/NSAJIGWA/180826_1211_Sado.mp3time=1176800)

R: No [00:19:39-6](http://localhost:2300/file=C:/Users/admin/Desktop/NSAJIGWA/180826_1211_Sado.mp3time=1179600)

I: Don’t you get relief? [00:19:40-3](http://localhost:2300/file=C:/Users/admin/Desktop/NSAJIGWA/180826_1211_Sado.mp3time=1180300)

R: Yes [00:19:41-5](http://localhost:2300/file=C:/Users/admin/Desktop/NSAJIGWA/180826_1211_Sado.mp3time=1181500)

I: Do you also do prayers? [00:19:44-8](http://localhost:2300/file=C:/Users/admin/Desktop/NSAJIGWA/180826_1211_Sado.mp3time=1184800)

R: Church prayers [00:19:47-7](http://localhost:2300/file=C:/Users/admin/Desktop/NSAJIGWA/180826_1211_Sado.mp3time=1187700)

I: Yes [00:19:48-3](http://localhost:2300/file=C:/Users/admin/Desktop/NSAJIGWA/180826_1211_Sado.mp3time=1188300)

R: I do pray [00:19:49-0](http://localhost:2300/file=C:/Users/admin/Desktop/NSAJIGWA/180826_1211_Sado.mp3time=1189000)

I: Have you gone for prayers? [00:19:51-0](http://localhost:2300/file=C:/Users/admin/Desktop/NSAJIGWA/180826_1211_Sado.mp3time=1191000)

R: Yes [00:19:52-7](http://localhost:2300/file=C:/Users/admin/Desktop/NSAJIGWA/180826_1211_Sado.mp3time=1192700)

I: So, when there was no good results that’s when you went to hospital? [00:19:53-8](http://localhost:2300/file=C:/Users/admin/Desktop/NSAJIGWA/180826_1211_Sado.mp3time=1193800)

R: Yes, I do pray in church [00:19:57-4](http://localhost:2300/file=C:/Users/admin/Desktop/NSAJIGWA/180826_1211_Sado.mp3time=1197400)

I: Okay, thank you so much. That’s all I wanted to know. [00:20:04-8](http://localhost:2300/file=C:/Users/admin/Desktop/NSAJIGWA/180826_1211_Sado.mp3time=1204800)

**CASE 9**

I: Mmmh What is your name my sister, your name? [00:00:03-4](http://localhost:2300/file=C:/Users/Hp/Desktop/OTONDE/180829_1547_Anna.mp3time=3400)

R: My name is ----- [00:00:07-1](http://localhost:2300/file=C:/Users/Hp/Desktop/OTONDE/180829_1547_Anna.mp3time=7100)

I: A where are you from? [00:00:08-6](http://localhost:2300/file=C:/Users/Hp/Desktop)

R: ----- [00:00:09-9](http://localhost:2300/file=)

I: -----, and how old are you? [00:00:12-0](http://localhost:2300/file=C:/Users/Hp/Desktop/OTONDE/180829_1547_Anna.mp3time=12000)

R: ----- [00:00:13-7](http://localhost:2300/file=C:/Users/Hp/Desktop/OTONDE/180829_1547_Anna.mp3time=13700)

I: -----? And do you have children? [00:00:18-1](http://localhost:2300/file=C:/Users/H)

R: I have four children [00:00:20-5](%22h)

I: Are you married? [00:00:22-6](http://localhost:2300/file=C:/Users/Hp/Desktop/OTONDE/180829_1547_Anna.mp3time=22600)

R: (Nod head to agree)

I: And what is your highest level of education? [00:00:27-1](http://localhost:2300/file=C:/Users/Hp/Desktop/OTONDE/180829_1547_Anna.mp3time=27100)

R: Form four [00:00:28-4](http://localhost:2300/file=C:/Users/Hp/Desktop/OTONDE/180829)

I: What about your work, what do you do mostly? 00:00:33-3

R: Business [00:00:33-9](http://localhost:2300/file=C:/Users/Hp/Desktop/OTONDE/180829_1547_Anna.mp3time=33900)

I: What kind of business do you do? [00:00:35-5](http://localhost:2300/file=C:/Users/Hp/Desktop/OTONDE/180829_1547_Anna.mp3time=35500)

R: Just different kind of business, today I do this, I keep changing I have no specific business. [00:00:41-7](http://localhost:2300/file=C:/Users/Hp/Desktop/OTONDE/180829_1547_Anna.mp3time=41700)

I: Is it in the same region -----, or where do you do it? 00:00:43-9

R: In Tanga [00:00:44-7](http://localhost:2300/file=C:/Users/Hp/Desktop/OTONDE/180829_1547_Anna.mp3time=44700)

I: And where you stay is it in ----- town? [00:00:48-4](http://localhost:2300/file=C:/Users/Hp/Desktop/OTONDE/180829_1547_Anna.mp3t)

R: ----- [00:00:49-4](http://localhost:2300/file=C:/Us)

I: From where you live, ----- to the nearest health centre, how long does it take to get to there? [00:00:55-7](http://localhost:2300/file=C:/Users/Hp/Desktop/OTONDE/180829_1547_Anna.mp3time=55700)

R: It can take ten to fifteen minutes. 00:01:58-8

I: Do you walk or go by bus? [00:01:01-0](http://localhost:2300/file=C:/Users/Hp/Desktop/OTONDE/180829_1547_Anna.mp3time=61)

R: You can walk, anyhow depends on you [00:01:05-7](http://localhost:2300/file=C:/)

I: Normally do you walk or take a bus? [00:01:09-8](http://localhost:2300/file=C:/Users/Hp/Desktop/OTONDE/180829_1547_Anna.mp3time=69800)

R: I normally take a motorbike or *bajaji* [00:01:11-9](http://localhost:2300/file=C:/Users/Hp/Desktop/OTONDE/180829_1547_Anna.mp3time=71900)

I: And how much do you pay? 00:01:12-8

R: Two thousand for *bajaji* and one thousand for a motorbike. [00:01:18-7](http://localhost:2300/file=C:/Users/Hp/Desktop/OTONDE/180829_1547_Anna.mp3time=78700)

I: How did you reach at -----? [00:01:24-0](http://localhost:2300/file=C:/Users/Hp/Desktop/OTONDE/180829_1547_Anna.mp3time=84000) [00:01:23-7](http://localhost:2300/file=C:/Users/Hp/Desktop/OTONDE/180829_1547_Anna.mp3time=83700)

R: I came after....... [00:01:26-7](http://localhost:2300/file=C:/Users/Hp/Desktop/OTONDE/180829_1547_Anna.mp3time=86700)

I: Did you come by car or what means of transport did you use? [00:01:31-4](http://lo)

R: I came by a car

I: Okay; can you tell me how your disease started? [00:01:39-6](http://localhost:2300/file=C:/Users/Hp/Desktop/OTONDE/180829_1547_Anna.mp3time=99600)

R: It was in the year -----, I started feeling pain in the breast, it was just paining. I went to the ----- hospital, and told them, they gave me medications, antibiotics, I took them but the situation didn’t change it was still painful. I went again after I completed the dose, and they changed the dose they gave me another one. [00:02:14-3](http://localhost:2300/file=C:/Users/Hp/Desktop/O)

I: Was it only pain or there was something else? [00:02:16-8](http://localhost:2300/file=C:/Users/Hp/Desktop/OTONDE/180829_1547_Anna.mp3time=136800)

R: It was only pain, I didn’t feel anything else, it was only the breast paining the whole body. I saw that the medications were not helping, I came here in -----, I went to -----. When I was coming to -----, I saw a lump coming from the armpit, there is when I was startled. I went to ----- hospital. I was surprised that the lump in the armpit was in the breast but when I touch to feel, I don’t feel it. [00:02:50-4](http://localhost:2300/file=C:/Users/Hp/Desktop/OTONDE/180)

I: So when you touch you don’t see it [00:02:52-6](http://localhost:2300/file=C:/Users/Hp/Desktop/OTONDE/180829_1547_Anna.mp3time=172600)

R: Eee, when I reached -----, they screened me and they told me that they see signs of …you have swellings but these swellings have signs of cancer [00:03:06-9](http://localhost:2300/file=C:/Users/Hp/Desktop/OTONDE/180829_1547_Anna.mp3time=18)

I: That was in the year -----, the same year? 00:03:09-8

R: Mmmh so, I was to told to go to ----- for further check-up. I made arrangements of coming to -----. They took blood samples in the breast and in the armpit. They returned my results that I have cancer of the breast so, what I was supposed to do was to make follow up so that breast could be removed. Since I was poor, I couldn’t manage the costs. I struggled to get money for the investigations but I lacked money for surgery, it was every when I fight, I didn’t get enough money. . [00:04:06-7](http://localhost:2300/file=C:/Users/Hp/Desktop/OTONDE/180829_1547_Anna.mp3time=246700)

I: You were in Tanga or you were still here? [00:04:11-4](http://localhost:2300/file=C:/Users/Hp/Desktop/OTONDE/180829_1547_Anna.mp3time=251400)

R: I went back to -----, I went back, did some activities and got some money for the investigations about one hundred and sixty thousand shillings. I came for testing, and I was supposed to look for more money about seven hundred thousand shillings for the operation but I didn’t get, so I was informed that there is a certain kind of treatment that people are helped with and they get healed, so I was also going there. [00:04:40-2](%22http:)

I: What kind of treatment? [00:04:41-4](http://localhost:2300/file=C:/Users/Hp/Desktop/OTONDE/180829_1547_Anna.mp3time=281400)

R: Traditional treatment [00:04:42-2](http://localhost:2300/file=C:/Users/Hp/Desktop/OTONDE/180829_1547_Anna.mp3time=282200)

I: Traditional treatment, where was it? 00:04:46-2

R: Aaah I have taken some medications at different places, in -----, different places in ----- at a place there is a man called -----. There I have taken a lot of medications. And as I continued taking medications, they told me that as it continues to swell it might burst, and it will release the dirt in it and it will be okay. So, I continued taking the medications. [00:05:20-0](http://localhost:2300/file=C:/Users/Hp/Desktop/OTONDE/180829_1547_Anna.mp3time=320000)

I: While you were still seeing that it continues to swell? [00:05:21-9](http://localhost:2300/file=C:/Users/Hp/Desktop/OTONDE/180829_1547_Anna.mp3time=321900)

R: Eee it was swelling, and I was painful, I wasn’t happy. Since the year ----- I wasn’t happy until this year when it put on bed [00:05:37-6](%22http:/)

I: You also said that you went to different places, -----, any other place where you went to get the local medications? [00:05:45-0](http://localhost:2300/file=C:/Users/Hp/Desktop/OTONDE/180829_1547_Anna.mp3time=345000)

R: I went to ----- [00:05:47-3](http://localhost:2300/file=C:/Users/Hp/Desktop/OTONDE/180829_1547_Anna.mp3time=347300)

I: ----- in -----? [00:05:48-2](http://localhost:2300/file=C:/Users/Hp/Desktop/OTON)

R: ----- in ----- [00:05:52-9](http://localhost)

I: What kind of medication were they giving you? [00:05:56-4](http://localhost:2300/file=C:/Users/Hp/Desktop/OTONDE/180829_1547_Anna.mp3time=356400)

R: The local herbs [00:05:58-5](http://localhost:2300/file=C:/Users/Hp/Desktop/OTONDE/180829_1547_Anna.mp3time=358500)

I: He told you that…..the first one, he told you that it will swell and release the dirt or? [00:06:05-4](%22http:/)

R: the first one told me that I should use the medications and the swelling will disappear with time. I used his medicines for three months, but the swelling was still there. I left and went to the other person because I was already confused. [00:06:25-6](%22http:)

I: That is why you looked into different places? [00:06:29-2](http://localhost:2300/file=C:/Users/Hp/Desktop/OTONDE/180829_1547_Anna.mp3time=389200)

R: Mmmh so I roamed here and there, when it reached on the fifth month, it was already full to an extent that I cannot sleep neither during the day, nor the night and the pain was taking place. I asked the one who was giving me the medicines and he told me to continue taking the medicines.

[00:06:54-6](http://localhost:2300/file=C:/Users/Hp/Desktop/OTONDE/180829_1547_Anna.mp3time=414600)

I: Who told you so? [00:06:57-1](http://localhost:2300/file=C:/Users/Hp/Desktop/OTONDE/180829_1547_Anna.mp3time=417100)

R: -----, and I continued to take the medications, later on it created something like a black thing all over the breast and then there were pores in it as if they were metallic in nature and down here it created something like as if I was burnt with fire. So, there was a medication that I was applying on that area, as I apply it there it continues to dig deeper and deeper [00:07:30-6](http://localhost:2300/file=C:/Users/Hp/Desktop/OTONDE/180829_1547_Anna.mp3time=450600)

I: Did he give it to you in person? [00:07:32-2](http://localhost:2300/file=C:/Users/Hp/Desktop/OTONDE/180829_1547_Anna.mp3time=452200)

R: Yes, and what he was telling me was the way it digs is how it eats away cancer, so let the meat be eaten away okay. So, pus, blood and sometimes I bleed about half a bucket, I get deficit of blood, and it continues that way. [00:07:53-6](http://localhost:2300/file=C:/Users/Hp/Desktop/OTONDE/180829_1547_Anna.mp3time=473600)

I: But he tells you that that is how the medication works? [00:07:57-1](http://localhost:2300/file=C:/Users/Hp/Desktop/OTONDE/180829_1547_Anna.mp3time=477100)

R: He tells me that is how It works, later on I saw that it was abnormal and not good, it became a wound, such that you cannot look at it, the pus is pouring out. I didn’t have peace and so I said let me go back to the hospital, if it is to cut it if possible then let me cut it. [00:08:22-7](http://localhost:2300/file=C:/Users/Hp/Desktop/OTONDE/1808)

I: So, the cost wasn’t that high at the traditional healer? [00:08:29-1](http://localhost:2300/file=C:/Users/Hp/Desktop/OTONDE/180829_1547_Anna.mp3time=509100)

R: Mmmhmmh, there are others who are too costly and others are not. [00:08:36-3](http://localhost:2300/file=C:/Users/Hp/Desktop/OTONDE/180829_1547_Anna.mp3time=516300)

I: How long did you take to be treated at the traditional healer? [00:08:40-8](http://localhost:2300/file=C:/Users/Hp/Desktop)

R: Two years now. [00:08:43-9](http://localho)

I: And do they say that there are such kind of people with similar illness that they have treated? [00:08:50-2](http://localhost:2300/file=C:/Users/Hp/Desktop/OTONDE/180829_1547_Anna.mp3time=530200)

R: they are there, many of them are healed, though I haven’t seen others [00:08:57-1](http://localhost:2300/file=C:/Users/Hp/Desktop/OTONDE/180829_1547_Anna.mp3time=537100)

I: What did he tell you that what exactly it was? [00:08:59-5](http://localhost:2300/file=C:/Users/Hp/Desktop/OTONDE/180829_1547_Anna.mp3time=539500)

R: Cancer [00:09:01-6](http://localhost:2300/file=C:/Users/Hp/Desktop/OTONDE/180829_1547_Anna.mp3time=541600)

I: He also said it was cancer? [00:09:03-4](http://localhost:2300/file=C:/Users/Hp/Desktop/OTONDE/180829_1547_Anna.mp3time=543400)

R: Mmmh [00:09:03-4](http://localhost:2300/file=C:/Users/Hp/Desktop/OTONDE/180829_1547_Anna.mp3time=543400)

I: AN, did you know the symptoms of cancer? [00:09:11-0](http://localhost:2300/file=C:/Users/Hp/Desktop/OTONDE/180829_1547_Anna.mp3time=551000)

R: No

I: You have never known until when you came for treatment at the hospital there is when the doctor informed you. [00:09:19-3](http://localhost:2300/file=C:/Users/Hp/Desktop/OTONDE/180829_1547_Anna.mp3time=559300)

R: Eee [00:09:20-2](http://localhost:2300/file=C:/Users/Hp/Desktop/OTONDE/180829_1547_Anna.mp3time=560200)

I: But before that you didn’t know and have you ever seen anyone who was suffering from this illness? 00 :09:27-4

R: Yes, I have seen. [00:09:29-8](http://localhost:2300/file=C:/Users/Hp/Desktop/OTONDE/180829_1547_Anna.mp3time=569800)

I: Were they relatives or? [00:09:31-7](http://localhost:2300/file=C:/Users/Hp/Deskto)

R: Aa aa they were neighbors and I see others in TV [00:09:37-8](http://localhost:2300/file=C:/Users/Hp/Desktop/OTONDE/180829_1547_Anna.mp3time=577800)

I: Did they recover when they were treated or?

I: Where were they treated? [00:09:54-9](http://localhost:2300/file=C:/Users/Hp/Desktop/OTONDE/180829_1547_Anna.mp3time=594900)

R: There is one person who is there till date, she came to stay at ----- [00:09:59-7](http://localhost:2300/file=C:/Users/Hp/Desktop/OTONDE/180829_1547_Anna.mp3time=599700)

I: So, when you knew that you too have the illness like theirs, did you ask maybe on the treatment at least from the one who is still there? [00:10:16-3](http://localhost:2300/file=C:/Users/Hp/Desktop/OTONDE/180829_1547_Anna.mp3time=616300)

R: Eee, No. [00:10:21-3](http://localhost:2300/file=C:/Users/Hp/Desktop/OTONDE/180829_1)

I: No, and if you knew you would be worried, that is why you didn’t want to know about it. You told me that the first problem is money but you personally know the treatment modalities, did that too restrain you? [00:10:43-2](http://localhost:2300/file=C:/Users/Hp/Desktop/OTONDE/180829_1547_Anna.mp3time=643200)

R: Eee, you know when you get a certain illness, worries, mmh until you come to accept that it will be a lot of work. [00:10:57-3](http://localhost:2300/file=C:/Users/Hp/Desktop/OTONDE/180829_1547_Anna.mp3time=657300)

I: Did the relatives know about the illness? [00:11:02-9](http://localhost:2300/file=C:/Users/Hp/Desktop/OTONDE/180829_1547_Anna.mp3time=662900)

R: They know [00:11:03-5](http://localhost:2300/file=C:/Users/Hp/Desktop/OTONDE/180829_1547_Anna.mp3time=663500)

I: And what did they say? [00:11:04-7](http://localhost:2300/file=C:/Users/Hp/Desktop)

R: Everyone remained silent [00:11:08-3](%22http:/)

I: So, there are those who advised you to get treatment from the hospital or? [00:11:13-8](http://localhost:2300/file=C:/Users/Hp/Desktop/OTONDE/180829_1547_Anna.mp3time=673800)

R: Eee but then... money [00:11:19-2](http://localhost:2300/file=C:/Users/Hp/Desktop/OTONDE/180829_1547_Anna.mp3time=679200)

I: And apart from money, apart from being treated by traditional medicine is there any other kind of treatment that you made follow up on? [00:11:30-1](http://localhost:2300/file=C:/Users/Hp/Desktop/OTONDE/180829_1547_Anna.mp3time=690100)

R: Mmmhmmh….? [00:11:30-8](http://localhost:2300/file=C:/Users/Hp/Desktop/OTONDE/180829_1547_Anna.mp3time=690800)

I: Maybe others go for prayers? [00:11:33-3](http://localhost:2300/file=C:/Users/Hp/Desktop/OTONDE/180829_1)

R: prayers I have…I didn’t make follow-up servants of God prayed for me. [00:11:41-2](http://localhost:2300/file=C:/Users/Hp/Desktop/OTONDE/180829_1547_Anna.mp3time=701200)

I: Servants of God tell you that, have they ever met such people and healed them? [00:11:47-8](http://localhost:2300/file=C:/Users/Hp/Desktop/OTONDE/180829_1547_Anna.mp3time=707800)

R: Eee, I see many of them being healed. [00:11:51-1](http://localhost:2300/file=C:/Users/Hp/Desktop/OTONDE/180829_1547_Anna.mp3tim)

I: What about the investigations, you told me that you have ever done investigations of the imaging is it mammography or ultrasound? [00:12:06-7](http://localhost:2300/file=C:/Users/Hp/Desktop/OTONDE/180829_1547_Anna.mp3time=726700)

R: In the past? [00:12:07-1](http://localhost:2300/file=C:/Users/Hp/Desktop/OTONDE/180829_1547_Anna.mp3time=727100)

I: Mmmh [00:12:07-5](http://localhost:2300/file=C:/Users/Hp/Desktop/OTONDE/180829_154)

R: Eee [00:12:08-7](http://localhost:2300/file=C:/Users/Hp/Desk)

I: You did it when you were still at home or? [00:12:11-5](http://localhost:2300/file=C:/Users/Hp/Desktop/OTONDE/180829_1547_Anna.mp3time=731500)

R: No here at ----- [00:12:12-6](http://localhost:2300/file=C:/Users/Hp/Desktop/OTONDE/180829_1547_Anna.mp3time=732600)

I: So, you didn’t get any investigation there? [00:12:14-9](http://localhost:2300/file=C:/Users/Hp/Desktop/OTONDE/180829_1547_Anna.mp3time=734900)

R: Aa aa I did the investigations here [00:12:16-9](http://localhost:2300/file=C:/Users/Hp/Desktop/OTONDE/180829_1547_Anna.mp3time=736900)

I: When you went to the hospital in ----- you didn’t do any investigations? [00:12:24-3](http://localhost:2300/file=C:/Users/Hp/Desktop/OTONDE/180829_1547_Anna.mp3time=744300)

R: Aah when I reached to that hospital, they only gave me medications and I left, they gave me antibiotics, I didn’t proceed with them [00:12:34-2](%22http:)

I: Then you came straight to -----. And how long did it take from the time you saw the first symptoms to the time you did the first investigation, the imaging [00:12:51-4](http://localhost:2300/file=C:/Users/Hp/Desktop/OTONDE/180829_1547_Anna.mp3time=7714)

R: Aaa I didn’t…just in the first days, I did the imaging, I did the imaging to prepare for the surgery. [00:13:02-5](http://localhost:2300/file=C:/Users/Hp/Desktop/OTONDE/180829_1547_Anna.mp3time=782500)

I: Okay….so you didn’t do it before [00:13:07-6](http://localhost:2300/file=C:/Users/Hp/Desktop/OTONDE/180829_1547_Anna.mp3time=787600)

R: It was only to prepare for the operation. [00:13:11-0](http://localhost:2300/file=C:/Users/Hp/Desktop/OTONDE/180829_1547_Anna.mp3time=791000)

I: Even when the lump was still small? [00:13:15-9](http://localhost:2300/file=C:/Users/Hp/Desktop/OTON)

R: Eee [00:13:16-5](http://localhost:2300/file=C:)

I: Also, did they do it for the sake of surgery...? [00:13:18-7](http://localhost:2300/file=C:/Users/Hp/Desktop/OTONDE/180829_1547_Anna.mp3time=798700)

R: Yes, at that time [00:13:20-4](http://localhost:2300/file=C:/Users/Hp/Desktop/OTONDE/180829_1547_Anna.mp3time=800400)

I: And any history from a relative having a similar problem like this? [00:13:27-3](http://localhost:2300/file=C:/Users/Hp/Desktop/OTONDE/180829_1547_Anna.mp3time=807300)

R: None [00:13:27-3](http://localhost:2300/file=C:/Users/Hp/Desktop/OTONDE/180829_1547_Anna)

I: You are the first [00:13:28-8](http://localhost:2300/file=C:/Users/Hp/Desktop/OTONDE/180829_1547_Anna.mp3time=808800)

R: Only I 00:13:30-1

I: And when they were treating you here, did they tell you what stage of cancer were you in? [00:13:37-6](http://localhost:2300/file=C:/Users/Hp/Desktop/OTONDE/180829_1547_Anna.mp3time=817600)

R: Second stage [00:13:39-1](http://localhost:2300/file=C:/Users/Hp/Desktop/OTONDE/180829_1547_Anna.mp3time=81910)

I: Second stage. And I see it took much time until when you started the treatment. What other problem do you think led to this apart from money, apart from the fact that you started with traditional medicine, what other thing led to this? [00:14:01-7](http://localhost:2300/file=C:/Users/Hp/Desktop/OTONDE/180829_1547_Anna.mp3time=841700)

R: Until I reached the second stage? [00:14:03-3](http://localhost:2300/file=C:/Users/Hp/Desktop/OTONDE/180829_1547_Anna.mp3time=843300)

I: And you came to discover [00:14:05-5](http://localhost:2300/file=C:/Users/Hp/Desktop/OTONDE/180829_1547_Anna.mp3time=845500)

R: Aaa currently as I told you, I was using medications, and the medications I was using are the ones which made me come back to the hospital to see if there are possibilities of … [00:14:27-7](%22http://)

I: You personally, how do you see your faith, do you think the hospital treatment will help you or the traditional treatment? [00:14:40-8](http://localhost:2300/file=C:/Users/Hp/Desktop/OTONDE/180829_1547_Anna.mp3time=880800)

R: Mmmh, I don’t know where I should direct my faith now [00:14:47-3](http://localhost:2300/file=C:/Users/Hp/Desktop/OTONDE/180829_1547_Anna.mp3time=887300)

I: So, you are like doing some trials if this will succeed? [00:14:53-4](http://localhost:2300/file=C:/Users/Hp/Desktop/OTONDE/180829_1547_Anna.mp3time=893400)

R: I am just here [00:14:55-9](http://localhost:2300/file=C:/Users/Hp/Desktop/OTONDE/180829_1547_Anna.mp3time=895900)

I: And what were you told were the treatment modalities of cancer? [00:14:59-0](http://localhost:2300/file=C:/Users/Hp/Desktop/OTONDE/180)

R: There is radiations, there is chemo, and also surgery [00:15:09-4](http://localhost:2300/file=C:/Users/Hp/Desktop/OTONDE/180829_1547_Anna.mp3time=909400)

I: Did you have any worries when they did surgery? [00:15:14-1](http://localhost:2300/file=C:/Users/Hp/Desktop/OTONDE/180829_1547_Anna.mp3time=914100)

R: Mmmh, I was worried [00:15:20-3](http://localhost:2300/file=C:/Users/Hp/Desktop/O)

I: Do you have a relative in -----? [00:15:23-5](%22)

R: Eee [00:15:24-6](http://localhost:2300/file=C:/Users/Hp/Desktop/OTONDE/180829_1547_Anna.mp3time=924600)

I: So, do you stay at your relative’s place? [00:15:26-7](http://localhost:2300/file=C:/Users/Hp/Desktop/OTONDE/180829_1547_Anna.mp3time=926700)

R: Aa aa, I came directly from ----- [00:15:32-0](http://localhost:2300/file=C:/Users/Hp/Desktop/OTONDE/180829_1547_Anna.mp3time=932000)

I: Is there any relative you live with here? [00:15:34-2](http://localhost:2300/file=)

R: Pardon? [00:15:35-3](%22h)

I: Did you come alone or with your relative? [00:15:37-5](http://localhost:2300/file=C:/Users/Hp/Desktop/OTONDE/180829_1547_Anna.mp3time=937500)

R: I came with my relative and there are other relatives here. [00:15:40-3](http://localhost:2300/file=C:/Users/Hp/Desktop/OTONDE/180829_1547_Anna.mp3time=940300)

I: Thank you for your time. Maybe if you have anything else to say? [00:16:01-1](http://localhost:2300/file=C:/Users/Hp/Desktop/OTONDE/180829_1547_Anna.mp3time=961100)

R: No, that is all [00:16:02-4](http://localhost:2300/file=C:/Users/Hp/Desktop/OTONDE/180829_1547_Anna.mp3time=962400)

I: Thank you for your explanations [00:16:06-4](http://localhost:2300/file=C:/Users/Hp/Desktop/OTONDE/180829_1547_Anna.mp3time=966400)

R: Okay [00:16:07-4](http://localhost:2300/file=C:/Users/Hp/Desktop/OTONDE/180829_1547_Anna.mp3time=967400)

**CASE 10**

I: Mama, what is your name? [00:00:01-1](http://localhost:2300/file=C:/Users/admin/Desktop/NSAJIGWA/180904_1444_01_Regina.mp3time=1100)

R: ----- [00:00:04-1](%22http)

I: Where are you coming from? [00:00:04-8](http://localhost:2300/file=C:/Users/admin/Desktop/NSAJIGWA/180904_1444_01_Regina.mp3time=4800)

R: I come from -----. [00:00:07-2](http://localhost:2300/file=C:/Users/admin/Desktop/NSAJIGWA/180904_1444_01_Regina.mp3time=7200)

I: ----- -----? [00:00:09-6](http://localhost:2)

R: Yes. [00:00:10-3](http://localhost:2300/file=C:/Users/admin/Desktop/NSAJIGWA/180904_1444_01_Regina.mp3time=10300)

I: What is your age? [00:00:11-3](http://localhost:2300/file=C:/Users/admin/Desktop/NSAJIGWA/180904_1444_01_Regina.mp3time=11300)

R: ----- [00:00:13-6](http://localhost:2300/file=C:/Users/admin/Desktop/NSAJIGWA/180904_1444_01_Regina.mp3time=13600)

I: And what is your marital status, do you have a husband and children [00:00:14-8](http://localhost:2300/file=)

R: Yes, I do have #00:00:1 [00:00:20-1](http://localhost:2300/file=C:/Users/admin/Desktop/NSAJIGWA/180904_1444_01_Regina.mp3time=20100)

I: What is your education level? [00:00:23-9](http://localhost:2300/file=C:/Users/admin/Desktop/NSAJIGWA/180904_1444_01_Regina.mp3time=23900)

R: Form 4 [00:00:26-8](http://localhost:2300/file=C:/Users/admin/Desktop/NSAJIGWA/180904_1444_01_Regina.mp3time=26800)

I: Form 4? [00:00:28-0](http://localhost:2300/file=C:/Users/admin/Desktop/NSAJIG)

R: Yes [00:00:28-7](http://localhost:2300/file=C:/Users/admin/Desktop/NSAJIGWA/180904_1444_01_Regina.mp3time=28700)

I: What work are you doing or that you were doing in the past [00:00:29-5](http://localhost:2300/file=C:/Users/admin/Desktop/NSAJIGWA/180904_1444_01_Regina.mp3time=29500)

R: I was a Veterinary officer [00:00:34-8](http://localhost:2300/file=C:/)

I: Veterinary officer? [00:00:38-9](http://localhost:2300/file=C:/Users/admin/Desktop/NSAJIGWA/180904_1444_01_Regina.mp3time=38900)

R: Yes [00:00:40-0](http://localhost:2300/file=C:/Users/admin/Desktop/NSAJIGWA/180904_1444_01_Regina.mp3time=40000)

I: Oh, okay [00:00:40-3](http://localhost:2300/file=C:/Users/admin/Desktop/NSAJIGWA/180904_1444_01_Regina.mp3time=40300)

I:But I opted to retire [00:00:41-9](http://localhost:2300/file=C:/User)

I: Oh! [00:00:45-1](http://localhost:2300/file=C:/Users/admin/Desktop/NSAJIGWA/180904_1444_01_Regina.mp3time=45100)

R: Yes [00:00:45-9](http://localhost:2300/file=C:/Users/admin/Desktop/NSAJIGWA/180904_1444_01_Regina.mp3time=45900)

I: Why did you opt to retire? [00:00:46-8](http://localhost:2300/file=C:/Users/admin/Desktop/NSAJIGWA/180904_1444_01_Regina.mp3time=46800)

R: I wanted to establish myself and prepare for life after retirement since there is an end to being a civil servant [00:00:48-5](http://localhost:2300/file=C:/Users/admin/Desktop/NSAJIGWA/180904_1444_01_Regina.mp3time=48500)

I: Mmh, [00:00:56-2](http://localhost:2300/file=C:/Users/admin/Desktop/NSAJIGWA/180904_1444_01_Regina.mp3time=56200)

R: Yes [00:00:57-0](http://localhost:2300/file=C:/Users/admin/Desktop/NSAJIGWA/180904_1444_01_Regina.mp3time=57000)

I: Okay, so do you have a health seeking behavior like going to hospital for a checkup even when you are not sick [00:00:57-5](http://localhost:2300/file=C:/Users/admin/Desktop/NSAJIGWA/180904_1444_01_Regina.mp3time=57500)

R: Seldom [00:01:09-3](http://localhost:2300/file=C:/Users/admin/Desktop/NSAJIGWA/180904_1444_01_Regina.mp3time=69300)

I: Seldom? [00:01:11-1](http://localhost:2300/file=C:/Users/admin/Desktop/NSAJIGWA/180904_1444_01_Regina.mp3time=71100)

R: Yes [00:01:11-9](http://localhost:2300/file=C:/Users/admin/Desktop/NSAJIGWA/180904_1444_01_Regina.mp3time=71900)

I: And what is the estimated distance from where you live at ----- to hospital [00:01:13-0](http://localhost:2300/file=C:/Users/admin/Desktop/NSAJIGWA/180904_1444_01_Regina.mp3time=73000)

R: At first it was far since I was in the village but for now I am just nearby, approximately 2km [00:01:21-1](http://localhost:2300/file=C:/Users/admin/Desktop/NSAJIGWA/180904_1444_01_Regina.mp3time=81100)

I: Mmh [00:01:30-3](http://localhost:2300/file=C:/Users/admin/Desktop/NSAJIGWA/180904_1)

R: Yes [00:01:31-0](http://localhost:2300/fil)

I: How do you go to hospital, do you board a car or [00:01:31-8](http://localhost:2300/file=C:/Users/admin/Desktop/NSAJIGWA/180904_1444_01_Regina.mp3time=91800)

R: Either way it is possible, on foot, or boarding tricyclic motorcycles or by motorcycle [00:01:34-0](http://localhost:2300/file=C:/Users/admin/Desktop/NSAJIGWA/180904_)

I: Okay [00:01:41-9](http://localhost:2300/fi)

R: Yes [00:01:42-6](http://localhost:2300/file=C:/Users/admin/Desktop/NSAJIGWA/180904_1444_01_Regina.mp3time=102600)

I: And, how to you get informed on health related issues , through mobile phones or radio, [00:01:43-7](http://localhost:2300/file=C:/Users/admin/Desktop/NSAJIGWA/180904_1444_01_Regina.mp3time=103700)

R: Radio and TV [00:01:54-1](http://localhost:2300/file=C)

I: Do you use mobile phone? [00:01:57-3](http://localhost:2300/file=C:/Users/admin/Desktop/NSAJIGWA/180904_1444_01_Regina.mp3time=117300)

R: Mobile phone? [00:01:59-6](http://localhost:2300/file=C:/Users/admin/Desktop/NSAJIGWA/180904_1444_01_Regina.mp3time=119600)

I: Yes [00:02:01-0](http://localhost:2300/file=C:/Users/admin/Desktop/NS)

R: Not so often with mobile phone [00:02:02-6](http://localhost:2300/file=C:/Users/admin/Desktop/NSAJIGWA/180904_1444_01_Regina.mp3time=122600)

I: Mmh [00:02:04-6](http://localhost:2300/file=C:/Users/admin/Desktop/NSAJIGWA/180904_1444_01_Regina.mp3time=124600)

R:Yes [00:02:04-3](http://localhost:2300/file=C:/Users/admin/Desktop/NSAJIGWA/180904_1444_01_Regina.mp3time=124300)

I: Are you an internet user? [00:02:05-5](http://localhost:2300/file=C:/)

R: No. [00:02:07-7](http://localhost:2300/file=C:/Users/admin/Desktop/NSAJIGWA/180904_1444_01_Regina.mp3time=127700)

I: Mmh,Can you explain to me in short on how all things started since when you were told that you are sick to when you started treatment [00:02:08-7](http://localhost:2300/file=C:/Users/admin/Desktop/NSAJIGWA/180904)

R: In short, I had an accident on this hand [00:02:24-7](http://localhost:2300/file=C:/Users/admin/Desktop/NSAJIGWA/180904_1444_01_Regina.mp3time=144700)

I: Mmh [00:02:31-4](http://localhost:2300/file=C:/Users/admin/Desktop/NSAJIGWA/180904_1444_01_Regina.mp3time=151400)

R: When I had an accident I was kept on POP and I had it for a long time about 8months [00:02:32-0](http://localhost:2300/file=C:/Users/admin/Desktop/NSAJIGWA/180904_1444_01_Regina.mp3time=152000)

I: Mmh [00:02:36-9](http://localhost:2300/file=C:/Users/admin/Desktop/NSAJIGWA/180904_1444_01_Regina.mp3time=156900)

R: Because I had an accident I got fractured and metals were kept on me, [00:02:36-8](http://localhost:2300/file=C:/Users/admin/Desktop/NSAJIGWA/180904_144)

I: Mmh [00:02:41-2](http://localhost:2300/file=C:/Users/admin/Desktop/NSAJIGWA/180904_1444_01_Regina.mp3time=161200)

R: Unfortunately, while I was going for metal removal I got another challenge so I had another fracture so I had another POP and so I placed my hand to chest level for a long time [00:02:41-6](http://localhost:2300/file=C:/Users/admin/Desktop/NSAJIGWA/180904_1444_01_Regina.mp3time=161600)

I:Mmh [00:02:58-0](http://localhost:2300/file=C:/Users/admin/Desktop/NSAJIGWA/180904_1444_01_Regina.mp3time=178000)

R: You see, [00:02:58-5](http://localhost:2300/file=C:/Users/admin/Desktop/NSAJIGWA/180904_1444_01_Regina.mp3time=178500)

I: Yes [00:02:59-6](http://localhost:2300/file=C:/Users/admin/Desktop/NSAJIGWA/180904_1444_01_Regina.mp3time=179600)

R: So when I came for POP removal [00:03:00-1](http://localhost:2300/file=C:/Users/admin/Desktop/NSAJIGWA/180904_1444_01_Regina.mp3time=180100)

I: Mmh [00:03:03-9](http://localhost:2300/file=C:/Users/admin/Desktop/NSAJIGWA/180904_1444_01_Regina.mp3time=183900)

R: That’s when I noticed a small swelling [00:03:04-8](http://localhost:2300/file=C:/Users/admin/Desktop/NSAJIGWA/180904_1444_01_Regina.mp3time=184800)

I: Mmh [00:03:07-6](http://localhost:2300/file=C:/Users/admin/Desktop/NSAJIGWA/180904_1444_01_Regi)

R: A small one here on the breast [00:03:08-1](%22http:)

I: Mmh [00:03:10-3](http://localhost:2300/file=C:/Users/admin/Desktop/NSAJIGWA/180904_1444_01_Regina.mp3time=190300)

R: So I went to hospital and the doctor that was there told me that it wasn’t a problem [00:03:10-8](http://localhost:2300/file=C:/Users/admin/Desktop/NSAJIGWA/180904_1444_01_Regina.mp3time=190800)

I: Mmh [00:03:17-6](http://localhost:2300/file=C:/Users/admin/Desktop/NSAJIGWA/180904_1444_01_Regina.mp3time=197600)

R: Yes, that’s not a problem so go proceed with your works. [00:03:18-2](http://localhost:2300/file=C:/Users/admin/Desktop/NSAJIGWA/180904_1444_01_Regina.mp3t)

I: Mmh [00:03:21-3](http://localhost:2300/file=C:/Users/admin/Desktop/NSAJIGWA/180904_1444_01_Regina.mp3time=201300)

R: But when I stayed for sometime I returned to hospital for the second time then I said why is it progressing is size? [00:03:21-9](http://localhost:2300/f)

I: Mmh [00:03:27-6](http://localhost:2300/file=C:/Users/admin/Desktop/NSAJIGWA/180904_1444_01_Regina.mp3time=207600)

R: I met with another doctor [00:03:28-4](http://localhost:2300/file=C:/Users/admin/Desktop/NSAJIGWA/180904_1444_01_Regina.mp3time=208400)

I: Mmh [00:03:30-1](http://localhost:2300/file=C:/Users/admin/Desktop/NSAJIGWA/180904_1444_01_Regina.mp3time=210100)

R: And he told me, no, you should take a step [00:03:30-6](http://localhost:2300/file=C:/Users/admin/Desktop/NSAJIGWA/180904_1444_01_Regina.mp3time=210600)

I: Mmh [00:03:32-2](http://localhost:2300/file=C:/Users/admin/Desktop/NSAJIGWA/180904_1444_01_Regina.mp3time=212200)

R: Yes, I will give you these medications to slow the progress then he asked if it was aching then I replied, no, then he insisted that I should take a step [00:03:32-9](%22http://)

I: Mmh [00:03:40-3](http://localhost:2300/file=C:/Users/admin/Desktop/NSAJIGWA/180904_1444_01_Regina.mp3time=220300)

R: So I went to ----- Hospital [00:03:41-0](http://localhost:2300/file=C:/Users/admin/Desktop/NSAJIGWA/180904_1444_01_Regina.mp3time=221000)

I: Mmh [00:03:44-0](http://localhost:2300/file=C:/Users/admin/Desktop/NS)

R: After going to -----, doctor examined me and noticed that I had a high blood pressure [00:03:45-1](http://localhost:2300/file=C:/Users/admin/Desktop/NSAJIGWA/180904_1444_01_Regina.mp3time=225100)

I: Mmh #00:03:5 [00:03:57-9](http://localhost:2300/file=C:/Users/admin/Desktop/NSAJIGWA/180904_1444_01_Regina.mp3time=237900)

R: I had a systolic of 200 and something [00:03:58-4](http://localhost:2300/file=C:/Users/admin/Deskt)

I: While on examination? [00:03:59-8](http://localhost:2300/file=C:/Users/admin/Desktop/NSAJIGWA/180904_1444_01_Regina.mp3time=239800)

R: Yes [00:04:01-3](http://localhost:2300/file=C:/Users/admin/Desktop/NSAJIGWA/180904_1444_01_Regina.mp3time=241300)

I: Okay [00:04:01-8](http://localhost:2300/file=C:/Users/admin/Desktop/NSAJIGWA/180904_1444_01_Regina.mp3time=241800)

R: So he said that they won’t do anything, He could have taken me to theatre but for now deal with the high blood pressure , with such high blood pressure you can fall by anytime [00:04:02-5](http://localhost:2300/file=C:/Users/admin/Desktop/NSAJIGWA/180904_1444_01_Regina.mp3time=242500)

I: Okay [00:04:11-8](http://localhost:2300/file=C:/Users/admin/Desktop/NSAJIGWA/180904_1444_01_Regina.mp3time=251800)

R: It is too high [00:04:12-1](http://localhost:2300/file=C:/Users/admin/Desktop/NSAJIGWA/180904_1444_01_Regin)

I: Mmh [00:04:12-9](http://localhost:2300/file=C:/User)

R: He gave me pills to us for a month [00:04:13-4](http://localhost:2300/file=C:/Users/admin/Desktop/NSAJIGWA/180904_1444_01_Regina.mp3time=253400)

I: Mmh [00:04:15-9](http://localhost:2300/file=C:/Users/admin/Desktop/NSAJIGWA/180904_1444_01_Regina.mp3time=255900)

R: Once I was done with the medication I decided that I better go to ----- Referral Hospital [00:04:16-0](http://localhost:2300/file=C:/User)

I: So, you never wanted to go back to there [00:04:24-4](http://localhost:2300/file=C:/Users/admin/Desktop/NSAJIGWA/180904_1444_01_Regina.mp3time=264400)

R: I never wanted to go back there because even with the expenses at ----- I had someone to host me so in case of any challenges then there is someone to help [00:04:25-6](http://localhost:2300/file=C:/Users/admin/Desktop/NSAJIGWA/180904_1444_01_Regina.mp3time=265600)

I: Okay [00:04:34-1](http://localhost:2300/file=C:/Users/admin/Desktop/NSAJIGWA/180904_1444_01_Regina.mp3time=274100)

R: Yes, so when I arrived there high blood pressure was still a problem, it hadn’t been lowered [00:04:34-8](http://localhost:2300/file=C:/Users/admin/Desktop/NSAJIGWA/180904_1444_01_Regina.mp3time=274800)

I: Mmh [00:04:39-2](http://localhost:2300/file=C:/Users/admin/Desktop/NSAJIGWA/180904_1444_01_Regina.mp3time=279200)

R: Yes, so I stayed for like 3 days ,then after that that’s when they started investigations [00:04:39-5](http://localhost:2300/file=C:/Users/admin/Desktop/NSAJIGWA/180904_1444_01_Regina.mp3time=279500)

I: Investigations? [00:04:44-0](http://localhost:2300/file=C:/Users/admin/Desktop/NSAJIGWA/180904_1444_01_Regina.mp3time=284000)

R: Yes, investigations. At ----- they said that they will deal with the hypertension and going to referral hospital they gave me medications and they said that I should start attending clinic everyday [00:04:45-0](http://localhost:2300/file=C:/Users/admin/Desktop/NSAJIGWA/180904_1444_01_Regina.mp3time=285000)

I: For hypertension? [00:04:59-8](http://localhost:2300/file=C:/Users/admin/Desktop/NSAJIGWA/180904_1444_01_Regina.mp3time=299800)

R: Yes, [00:05:01-3](http://localhost:2300/file=C:/Users/admin/Desktop/NSAJIGW)

I: Okay [00:05:01-8](http://local)

R: The blood pressure was too high, systolic was something like [00:05:02-3](http://localhost:2300/file=C:/Users/admin/Desktop/NSAJIGWA/180904_1444_01_Regina.mp3time=302300)

I: Okay [00:05:06-6](http://localhost:2300/file=C:/Users/admin/Desktop/NSAJIGWA/180904_1444_01_Regina.mp3time=306600)

R: After that I started doing investigations and I was ordered to do ultrasound [00:05:07-1](http://localhost:2300/file=C:/Users/admin/Desktop/NSAJIGWA/180904_1444_01_Regina.mp3time=307100)

I:Mmh [00:05:13-4](http://localhost:2300/file=C:/Users/admin/Desktop/NSAJIGWA/180904_1444_01_Regina.mp3time=313400)

R: After going for ultrasound I was referred to ----- hospital [00:05:14-0](http://localhost:2300/file=C:/Users/admin/Desktop/NSAJIGWA/180904_1444_01_Regina.mp3time=31400)

I: The one in -----? [00:05:20-0](http://localhost:2300/file=C:/Users/admin/Desktop/NSAJIGWA/180904_1444_01_Regina.mp3time=320000)

R: Yes, at ----- hospital and biopsy was taken [00:05:21-3](http://localhost:2300/file=C:/Users/admin/Desktop/NSAJIGWA/180904_1444_01_Regina.mp3time=321300)

I: Mmh [00:05:27-0](http://localhost:2300/file=C:/Users/admin/Desktop/NSAJIGWA/180904_1444_01_Regina.mp3time=327000)

R: Yes, that was in May [00:05:27-5](http://localhost:2300/file=C:/Users/admin/Desktop/NSAJI)

I: May? [00:05:29-8](http://lo)

R: Yes [00:05:30-5](http://localhost:2300/file=C:/Users/admin/Desktop/NSAJIGWA/180904_1444_01_Regina.mp3time=330500)

I: What time had passed since you first saw the swelling [00:05:31-1](http://localhost:2300/file=C:/Users/admin/Desktop/NSAJIGWA/180904_1444_01_Regina.mp3time=331100)

R: It was in January when I saw it for the first time [00:05:34-7](%22h)

I: So it was five months now [00:05:36-7](http://localhost:2300/file=C:/Users/admin/Desktop/NSAJIGWA/180904_1444_01_Regina.mp3time=336700)

R: Yes, so they had taken a biopsy , when they took biopsy it was on ----- [00:05:38-1](http://localhost:2300/file=C:/Users/admin/Desktop/NSAJIGWA/180904_1444_01_R)

I: Mmh [00:05:49-6](http://localhost:2300/file=C:/Users/admin/Desktop/NSAJIGWA/180904_1444_01_Regina.mp3time=349600)

R: So, whenever I asked for biopsy results they would tell me that they are not out yet, then later was informed that the results were out and that I should go [00:05:50-1](http://localhost:2300/file=C:/Users/admin/Deskt)

I: Mmh [00:06:02-5](%22ht)

R: So, I went, when I arrived, I was given my results and was told to take them to doctor, after taking them to doctor he said that he couldn’t read the results well since the biopsy taken wasn’t adequate. That was on Wednesday so they decided that I go on Friday for admission and when I went on Friday I do not remember the exact date since it was so sudden [00:06:03-1](http://localhost:2300/file=C:/Users/admin/Desktop/NSAJIGWA/180904_1444_01_Regina.mp3time=363100)

I: Mmh [00:06:26-5](http://localhost:2300/file=C:/Users/admin/Desktop/NSAJIG)

R: So I got admitted and on Saturday morning I was among the people who were on the list of going to theatre, and I was well prepared to theatre [00:06:26-9](http://localhost:2300/file=C:/Users/admin/Desktop/NSAJIGWA/180904_1444_01_Regina.mp3time=386900)

I: Mmh [00:06:36-5](http://localhost:2300/file=C:/Users/admin/Desktop/NSAJIGWA/180904_1444_01_Regina.mp3t)

R: Everything was well prepared, when they wanted to start working on me they discussed amongst themselves and I didn’t understand what they were discussing since it’s not my profession [00:06:37-0](http://localhost:2300/file=C:/Users/admin/Desktop/NSAJIGWA/180904_1444_01_Regina.mp3time=397000)

I: Mmh [00:06:45-3](http://localhost:2300/file=C:/Users/admin/Desktop/NSAJIGWA/180904_1444_01_Regina.mp3time=405300)

R:They asked me on who my doctor was, I mentioned him, so they called him and it seemed that they wanted to use something but I couldn’t understand the terminologies [00:06:45-8](http://localhost:2300/file=C:/Users/adm)

I: Mmh [00:06:54-6](http://localhost:2300/file=C:/Users/admin/Desktop/NSAJIGWA/180904_1444_01_Regina.mp3time=414600)

R: So I didn’t get to understand them just that they told me to return to the ward[00:06:55-1](http://localhost:2300/file=C:/Users/admin/Desktop/NSAJIGWA/180904_1444_01_Regina.mp3time=415100)

I: So before telling you, didn’t they tell you the reason they took you to theatre didn’t they tell what the problem was [00:07:00-4](http://localhost:2300/file=C:/Users/admin/Desktop/NSAJIGWA/180904_1444_01_Regina.mp3time=420400)

R: Theatre? [00:07:05-8](http://localhost:2300/file=C:/Users/admin/Desktop/NSAJIGWA/180904_1444_01_Regina.mp3time=425800)

I: Yes [00:07:06-3](http://localhost:2300/file=C:/Users/admin/Desktop/NSAJIGWA/180904_1444_01_Regina.mp3time=426300)

R: They took me to theatre for swelling removal [00:07:06-7](http://localhost:2300/file=C:/U)

I: And did they tell you what the swelling was [00:07:08-7](http://localhost:2300/file=C:/Users/admin/Desktop/NSAJIGWA/180904_1444_01_Regina.mp3time=428700)

R: They didn’t mention, no one ever mentioned [00:07:10-6](http://localhost:2300/file=C:/Users/admin/Desktop/NSAJIGWA/180904_1444_01_Regina.mp3time=430600)

I: Mmh [00:07:13-8](http://localhost:2300/file=C:/Users/admin/Desktop/NSAJIGWA/180904_1444_01_Regina.mp3time=433800)

R: Yes, I guess they wanted to mention it through the sample results [00:07:14-8](http://localhost:2300/file=C:/Users/admin/Desktop/NSAJIGWA/180904_1444_01_Regina.mp3time=434800)

I: Okay [00:07:18-2](http://localhost:2300/file=C:/Users/admin/Desktop/NSAJIGWA/180904_1444_01_Regina.mp3time=43820)

R: Of which there were no results, so what then can they mention, I think that is where the stammering begun [00:07:18-7](http://localhost:2300/file=C:/Users/admin/Desktop/NSAJIGWA/180904_1444_01_Regina.mp3time=438700)

I: Yes [00:07:26-4](http://localhost:2300/file=C:/Users/admin/Desktop/NSAJIGWA/180904_1444_01_Regina.mp3time=446400)

R: So, it was decided that I should get back to the ward , and that was on Saturday, when Monday reached around visiting hours in the afternoon, my relatives came and inquired on what was going on concerning treatment [00:07:26-9](http://localhost:2300/file=C:/Users/admin/Desktop/NSAJIGWA/180904_1444_01_Regina.mp3time=446900)

I: Mmh [00:07:38-3](http://localhost:2300/file=C:/Users/admin/Desktop/N)

R: So after my relatives asked it seemed that staying in the hospital is the system and someone can’t share an information that is not sure of [00:07:39-0](http://localhost:2300/file=C:/Users/admin/Desktop/NSAJIGWA/180904_1444_01_Regina.mp3time=459000)

I: Mmh [00:07:50-9](http://localhost:2300/file=C:/Users/admin/Deskt)

R: So we decided that we get the referral letter and move forward to higher level [00:07:51-4](http://localhost:2300/file=C:/Users/admin/Desktop/NSAJIGWA/180904_1444_01_Regina.mp3time=471400)

I: You mean coming to -----? [00:07:56-7](http://localhost:2300/file=C:/Users/admin/Desktop/NSAJIGWA/180904_1444_01_Regina.mp3time=476700)

R: Yes, so was given letter that’s when we came to -----, and at ----- I stayed for a long time due to time taking investigations because my first investigation was done ----- [00:07:57-7](http://localhost:2300/file=C:/Users/admin/Desktop/NSAJIGWA/180904_1444_01_Regina.mp3time=477700)

I: July? [00:08:15-4](http://localhost:2300/file=C:/Users/admin/Desktop/NSAJIGWA/180904_1444_01_Regina.mp3time=495400)

R: Yes, and the results came back this August don’t remember the exact date ,so even with the results they take too long and the patient keeps on been worried and not knowing what is happening but at the end I got operated on [00:08:16-0](http://localhost:2300/file=C:/Users/admin/Desktop/NSAJIGWA/180904_1444_01_Regina.mp3time=)

I: Mmh [00:08:36-4](http://localhost:2300/file=C:/Users/admin/De)

R: Yes, when we arrived on ----- [00:08:37-0](http://localhost:2300/file=C:/Users/admin/Desktop/NSAJIGWA/180904_1444_01_Regina.mp3time=517000)

I: Mmh [00:08:41-5](http://localhost:2300/file=C:/Users/admin/Desktop/NSAJIGWA/180904_1444_01_Regina.mp3time=521500)

R: The results came out on dates of ----- [00:08:41-9](%22http:)

I: A little time has passed [00:08:45-0](http://localhost:2300/file=C:/Users/admin/Desktop/NSAJIGWA/180904_1444_01_Regina.mp3time=525000)

R: Yes, they take too long and someone keeps on being hurt and worried you can die even before getting your biopsy results because you might think that you are having a serious issue that doctors can’t treat because we are used to other illnesses like malaria that you get tested and you receive results after a short while, but for this was different because I stayed for a whole month since I arrived here on June and on reaching here thinking that this will be your last hope but you come and stay again without any medication to ----- [00:08:45-8](http://localhost:2300/file=C:/Users/admin/Desktop/NSAJIGWA/180904_1444_01_Regina.mp3time=525800)

I: Mmh [00:09:07-2](http://localhost:2300/file=C:/Users/admin/Desktop/NSAJIGWA/180904_1444_01_Regina.mp3time=547200)

R: It disturbs a lot, and I do not know what is the problem whether it is the kind of treatment or what, I don’t understand [00:09:07-5](http://localhost:2300/file=C:/Users/admin/Desktop/NSAJIGWA/180904_1444_01_Regina.mp3t)

I: But for now, have you known already, are you told on what is the problem is. [00:09:12-7](http://localhost:2300/file=C:/Users/admin/Desktop/NSAJIGWA/180904_1444_01_Regina.mp3time=552700)

R: Yes, they already informed me [00:09:16-8](http://localhost:2300/file=C:/Users/admin/Desktop/NSAJIGWA/180904_1444_01_Regina.mp3time=556800)

I: Mmh [00:09:21-3](http://localhost:2300/file=C:/Users/admin/Desktop/NSAJIGWA/180904_1444_01_Regina.mp3time=561300)

R: That it is breast cancer [00:09:21-8](http://localhost:2300/file=C:/Us)

I: Mmh [00:09:23-7](http://localhost:2300/file=C:/Users/admin/Desktop/NSAJIGWA/180904_1444_01_Regina.mp3time=563700)

R: Yes [00:09:24-2](http://localhost:2300/file=C:/Users/admin/Desktop/NSAJIGWA/180904_1444_01_Regina.mp3time=564200)

I: Did you think of it before? Maybe when you first saw the swelling and decided to go to hospital [00:09:25-2](http://localhost:)

R: When I first saw it I went to hospital with similar thoughts but I didn’t have enough information about it [00:09:37-4](http://localhost:2300/file=C:/Users/admin/Desktop/NSAJIGWA/180904_1444_01_Regina.mp3time=577400)

I: Mmh [00:09:42-5](http://localhost:2300/file=C:/Users/admin/Desktop/NSAJIGWA/180904_1444_01_Regina.m)

R: About the course of the disease [00:09:42-9](http://lo)

I:Mmh [00:09:49-4](http://localhost:2300/file=C:/Users/admin/Desktop/NSAJIGWA/180904_1444_01_Regina.mp3time=589400)

R: Yes, but I knew on the existence of breast cancer, or uterine cancer I heard about them or any other cancer,I used to hear about them [00:09:49-0](http://localhost:2300/file=C:/Users/admin/Deskto)

I: Mmh [00:09:55-2](http://localhost:2300/file=C:/Users/admin/Desktop/NSAJIGWA/180904_1444_01_Regina.mp3time=595200)

R: Yes [00:09:55-6](http://localhost:2300/file=C:/Users/admin/Desktop/NSAJIGWA/180904_1444_01_Regina.mp3time=595600)

I: Okay [00:09:56-7](http://localhost:2300/file=C:/Users/admin/Desktop/NSAJIGWA/180904_1444_01_Regina.mp3time=596700)

R: Yes, and the one that reminded me is the first person who told me that it wasn’t a problem , I think if he could have alerted me like the second person, the first one had done me wrong [00:09:57-7](http://localhost:2300/file=C:/Users/admin/Desktop/NSAJIGWA/180904_1444_01_Regina.mp3time=597700)

I: So, when he told you to go back you truly went back and proceeded with your works as usual [00:10:07-4](http://localhost:2300/file=C:/Users/admin/Desktop/NSAJI)

R: Yes, he said that it wasn’t a problem [00:10:10-4](http://localhost:2300/file=C:/Users/admin/Desktop/NSAJIGWA/180904_1444_01_Regina.mp3time=610400)

I: Mmh [00:10:11-8](http://localhost:2300/file=C:/Users/admin/Desktop/NSAJIGWA/180904_1444_01_Regina.mp3time=611800)

R: Yes, and because he is only human then I do forgive him because he was supposed to alert me that I should take a step just as what the other person did to me [00:10:12-4](http://localhost:2300/file=C:/Users/admin/Desktop/NSAJIGWA/180904_1444_01_Regina.mp3time=612400)

I: Mmh [00:10:21-2](http://localhost:2300/file=C:/Users/admin/Desktop/NSAJIGWA/180904_1444_01_Regina.mp3time=621200)

R: So we had similar ideas and instead of telling me that I should go to a hospital where I can get quick results he allowed me to go back home [00:10:21-5](http://localhost:2300/file=C:/Users/admin/Desktop/NSAJIGWA/180904_1444_01_Regina.mp3time=621500)

I: Okay [00:10:35-5](http://localhost:2300/file=C:/Users/admin/Desktop/NSAJIGWA/180904_1444_01_Regina.mp3time=635500)

R: Yes [00:10:36-3](http://localhost:2300/file=C:/Users/admin/Desktop/NSAJIGWA/180904_1444_01_Regina.mp3time=636300)

I:And you told me that you did an ultrasound in ----- or was it in ----- hospital [00:10:36-9](http://localhost:2300/file=C:/Use)

R: No, in ----- [00:10:43-5](http://localhost:2300/file=C:/Users/admin/Desktop/NSAJIGWA/180904_1444_01_Regina.mp3time=643500)

I: -----? [00:10:44-5](http://localhost:2300/file=C:/Users/admin/Desktop/NSAJIGWA/180904_1444_01_Regina.mp3time=644500)

R: Yes [00:10:45-1](http://localhost:2300/file=C:/Users/admin/Desktop/NSA)

I: So didn’t you do any investigation in -----? [00:10:45-4](http://localhost:2300/file=C:/Users/admin/Desktop/NSAJIGWA/180904_1444_01_Regina.mp3time=645400)

R: Yes, I didn’t do any investigation apart from dealing with hypertension [00:10:46-9](http://localhost:2300/file=C:/Users/admin/Desktop/NSAJIGWA/180904_1444_01_Regina.mp3time=646900)

I: Oh [00:10:50-7](http://localhost:2300/file=C:/Users/admin/Desktop/NSAJI)

R: Yes, he said that due to hypertension he cant do anything since it was too high, that they might loose me anytime if left unattended [00:10:51-4](http://localhost:2300/file=C:/Users/admin/Desktop/NSAJIGWA/180904_1444_01_Regina.mp3time=651400)

I: Inaudible [00:10:57-6](http://localhost:2300/file=C:/Users/admin/Desktop/NSAJIGWA/180904_1444_01_Re)

R: Yes, [00:10:58-6](http://localhost:2300/file=C:/U)

I: Do you think you are a bit late to start getting treatment [00:10:59-1](http://localhost:2300/file=C:/Users/admin/Desktop/NSAJIGWA/180904_1444_01_Regina.mp3time=659100)

R: I can’t tell since am not a professional, and I do not know how the stages are but to an extent that an organ is removed it’s possible that I am late [00:11:03-1](http://localhost:2300/file=C:/Users/admin/Desktop/NSAJIGWA/180904_1444_01_Regina.mp3time=663100)

I: A bit late [00:11:17-5](http://localhost:2300/file=C:/Users/admin/Desktop/NSAJIGWA/180904_1444_01_Regina.mp3time=677500)

R: Yes [00:11:18-1](http://localhost:2300/file=C:/Users/admin/Desktop/NSAJIGWA/180904_1444_01_Regina.mp3time=678100)

I: And when you were told that you have breast cancer, were you about the stage [00:11:18-5](http://localhost:2300/file=C:)

R: They didn’t tell [00:11:23-6](http://localhost:2300/file=C:/Users/admin/Desktop/NSAJIGWA/180904_1444_01_Regina.mp3time=683600)

I: They didn’t mention? [00:11:24-5](http://localhost:2300/file=C:/Users/admin/Desktop/NSAJIGWA/180904_1444_01_Regina.mp3time=684500)

R: Yes [00:11:25-3](http://localhost:2300/file=C:/Users/admin/Desktop/NSAJIGWA/180904_1444_01_Regina.mp3time=685300)

I: And, we see that time has passed since you noticed about the swelling and that was in January and you tolerated to September that is when you got treated , so what could be reasons that contributed you to delay seeking for health [00:11:25-8](http://localhost:2300/file=C:/Users/admin/Desktop/NSAJIGWA/180904_1444_01_Regina.mp3time=685800)

R: Since I do not know, they probably were helping me once they weigh out hypertension and this [00:11:49-9](http://localhost:2300/file=C:/Users/admin/Desktop/NSAJIGWA/180904_1444_01_Regina.mp3time=709900)

I: Mmh [00:11:58-3](http://localhost:2300/file=C:/Users/admin/Desktop/NSAJIGWA/180904_1444_01_Regina.mp3time=718300)

R: Yes, but If the doctor from ----- could have said that I use the medications for few days Then return, it could have been better, but he said that I should use them for a month And days pass by, but I can still thank the doctor from ----- because he said that we should at least be communicating every day for updates so the one from ----- had helped me compared to the problem and he already knew that we have to rush , [00:12:09-0](http://localhost:2300/file=C:/Users/admin/Desktop/NSAJIGWA/180904_1444_01_Regina.mp3time=729000)

I: Was that in Mbeya? [00:12:23-9](http://localhost:2300/file=C:/Users/admin/Desktop/NSAJIGWA/180904)

R: Yes, that was in -----, but the worst that happened is, The person that took inadequate biopsy since am not a professional, just imagine that the sample stays for all those days and return with no results [00:12:28-9](http://localhost:2300/file=C:/Users/admin/Desktop/NSAJIGWA/180904_1444_01)

I: Mmh [00:12:40-2](http://localhost:2300/file=C)

R: Yes [00:12:40-5](http://localhost:2300/file=C:/Users/admin/Desktop/NSAJIGWA/180904_1444_01_Regina.mp3time=760500)

I: Was that biopsy taken in -----? [00:12:41-3](http://localhost:2300/file=C:/Users/admin/Desktop/NSAJIGWA/180904_1444_01_Regina.mp3time=761300)

R: Yes [00:12:42-3](http://localhost:2300/file=C:/Users/admin/Desktop/NSAJIGWA/180904_1444_01_Regina.mp3time=762300)

I: But was sent to ----- [00:12:43-1](http://localhost:2300/file=C:/Users/admin/Desktop/)

R: Is it not that they normally send it ----- where do they normally send then? [00:12:44-2](http://localhost:2300/file=C:/Users/admin/Desktop/NSAJIGWA/180904_1444_01_Regina.mp3time=764200)

I: They do histology over there [00:12:45-9](http://localhost:2300/file=C:/Users/admin/Desktop/NSAJIGWA/180904_1444_01_Regina.mp3time=765900)

R: I don’t know, do they do histology over there? [00:12:46-6](http://loca)

I:I think so [00:12:48-5](http://localhost:2300/file=C:/Users/admin/Desktop/NSAJIGWA/180904_1444_01_Regina.mp3time=768500)

R: Do they do it at moment? [00:12:50-0](http://localhost:2300/file=C:/Users/admin/Desktop/NSAJIGWA/180904_1444_01_Regina.mp3time=770000)

I: Am not so sure [00:12:51-5](http://localhost:2300/file=C:/Users/admin/Desktop/NSAJIGWA/180904_1444_01_Regina.mp3time=771500)

R: I don’t know [00:12:53-4](http://localhost:2300/file=C:/Users/admin/Desktop/NSAJIGWA/180904_1444_01_Regina.mp3time=773400)

I: But they said that they sent it to ----- [00:12:55-0](http://localhost:2300/file=C:/Users/admin/Desktop/NSAJIGWA/180904_1444_01_Regina.mp3time=775000)

R: I shouldn’t say that they sent the biopsy, they just told me that I will get my results after 3 to 4 weeks. Yes, So I do not your procedure as professionals [00:13:01-7](http://localhost:2300/file=C:/Users/admin/Desktop/NSAJIGWA/180904_1444_01_Regina.mp3time=781700)

I: Okay [00:13:06-0](http://localhost:2300/file=C:/Users/admin/Desktop/)

R: Yes, so that one too took time. Yes, and another thing and this is to others since to me it has already happened try to talk to the patient if you think that there is a problem, talk to the patient concerning the situation and give an advice on not to waste time for treatment and let the patient decide on his own [00:13:11-9](http://localhost:2300/file=C:/Users/admin/Desktop/NSAJIGWA/180904_1444_01_Regina.mp3time=791900)

I: Mmh [00:13:46-9](http://localhost:2300/file=C:/Users/admin/Desktop/NSAJIGWA/180904_1444_01_Regina.mp3time=826900)

R: Yes, [00:13:47-5](http://localhost:2300/file=C:/Users/admin/Desktop/NSAJIGWA/180904_1444_01_Regina.mp3time=827500)

I: Okay [00:13:48-5](http://localhost:2300/file=C:/Users/admin/Desktop/N)

R: Because for my case they didn’t do me good, since I took myself to hospital. But they are the ones who failed to give me the results and to today I still have the documents. It was just a matter of them saying that they are thinking of something and in order for the disease not to spread we need to take a step, so what are your views? And on that day my relatives had escorted me to hospital so it wasn’t a difficult issue, we could just sit and discuss. My husband was there and my other relatives, and since they are professionals, they should tell us on what they are thinking in regard to symptoms and for patients’ benefits should advice on what should be done to the patient then check on patient’s and family views. I think that was a right thing to do rather than just sending me here because when I arrived here I started having lots of cycles [00:14:35-4](http://localhost:2300/file=C:/Users/admin/Desktop/NSAJIGWA/180904_1444_01_Regina.mp3time=875400)

I: Here in Dar? [00:14:41-6](http://localhost:2300/file=C:/Users/admin/Desktop/NSAJIGWA/180904_1444_01_Regina.mp3time=881600)

R: There are a lot of procedures and are time taking , you can imagine since ----- to now that’s when am getting back the results, now I don’t understand whether it’s the type of disease or what [00:14:41-8](http://localhost:2300/file=C:/Users/admin/Desktop/NSAJIGWA/180904_1444_01_Regina.mp3time=881800)

I: With a lot of procedures what do you do mostly? [00:15:00-6](http://localhost:2300/file=C:/Use)

R: A lot of procedures are with investigations, for example I took a biopsy on -----. I was told to come back on ----- but it was not possible. The reason was when biopsy was taken it was not taken to the laboratory [00:15:11-6](http://localhost:2300/file=C:/Us)

I: Okay [00:15:18-1](http://localhost:2300/file=C:/Users/admin/Desktop/NSAJIGWA/180904_1444_01_Regina.mp3time=918100)

R: After ten days that is when they called me, if it truly was a mistake why did they take too long to call me back while they had my phone number and keeping in mind, I am still sick. Yes, that means they had my phone number and if there was anything that went wrong it was just a matter of them calling me back. So, they remained silent till when they called me on ----- at evening hours, on ----- we came and paid for what we were to pay then went to the main laboratory, but with the laboratory again I don’t know whether that is the system or the type of disease or maybe there are no reagents. Because it took a lot of time to get the results [00:15:56-1](http://localhost:2300/file=C:/Users/admin/Des)

I: From the -----? [00:15:59-6](http://localhost:2300/file=C:/Users/admin/Desktop/NSAJIGWA/180904_1444_01_Regina.mp3time=959600)

R: Yes, I do not know whether there a lot of patients or , because it takes a long time and the patient keeps on getting hurt [00:16:00-8](http://localhost:2300/file=C:/Users/admin/Desktop/NSAJIGWA/180904_)

I: Getting hurt? [00:16:08-4](http://loca)

R: Yes, so if there will be amendments on that I will be more glad [00:16:08-9](http://localhost:2300/file=C:/Users/admin/Desktop/NSAJIGWA/180904_1444_01_Regina.mp3time=968900)

I: Okay [00:16:14-6](http://localhost:2300/file=C:/Users/admin/Desktop/NSAJIGWA/180904_1444_01_Regina.mp3time=974600)

R: But they do take a long time [00:16:14-9](http://localhost:2300/file)

I: Mmh [00:16:16-6](http://localhost:2300/file=C:/Users/admin/Desktop/NSAJIGWA/180904_1444_01_Regina.mp3time=976600)

R: Yes [00:16:17-4](http://localhost:2300/file=C:/Users/admin/Desktop/NSAJIGWA/180904_1444_01_Regina.mp3time=977400)

I: And, what about getting referral, were there no challenges? [00:16:17-9](http://localhost:2300/file=C:/Users/admin/Desktop/NSAJIGWA/180904_1444_01_Regina.mp3time=977)

R: Refferal to here? 00:16:22-4

I: Yes [00:16:23-7](http://localhost:2300/file=C:/Users/admin/Desktop/NSAJIGWA/180904_1444_01_Regina.mp3time=983700)

R: No, there were no challenges with referral just that the treatments are way too slow [00:16:24-2](http://localhost:2300/file=C:/Users/admin/Desktop/NSAJIGWA/180904_1444_01_Regina.mp3time=984200)

I: Are they slow? [00:16:30-7](http://localhost:2300/file=C:/Users/admin/Desktop/NSAJIGWA/180904_1444_01_Regina.mp3time=990700)

R: Yes [00:16:30-9](http://localhost:2300/file=C:/Users/admin/Desktop/NSAJIGWA/180904_1444_01_Regina.mp3time=990900)

I: And do you use a health insurance card on doing payment 00:16:31-5

R: Yes [00:16:35-5](http://localhost:2300/file=C:/Users/admin/Desktop/NSAJIGWA/180904_1444_01_Regina.mp3time=995500)

I: And did you know the kind of treatment pertaining this disease, when you noticed that you have a swelling [00:16:39-0](http://localhost:2300/file=C:/Users/admin/Desktop/NSAJIGWA/180904)

R: Yes [00:16:46-3](http://localhost:2300/file=C:/Users/admin/Desktop/NSAJIGWA/180904_1444_01_Regina.mp3time=1006300)

I: Didn’t you have a feeling that the kind of treatment could involve breast removal? [00:16:46-6](http://localhost:2300/file=C:/Users/admin/Desktop/NSAJIGWA/180904_1444_01_Regina.mp3time=1006600)

R: I only felt that there would be swelling removal because even my colleagues who had swellings, swelling were removed [00:16:53-4](http://localhost:2300/file=C:/Users/admin/Desktop/NSAJIGWA/180904_1444_01_Regina.mp3time=1013400)

I: Mmh [00:17:00-7](http://localhost:2300/file=C:/Users/admin/Desktop/NSAJIGWA/180904_1444_01_Regina.mp3time=1020700)

R: Yes, so I do not know what comes next after removing the swelling [00:17:03-6](http://localhost:2300/file=C:/Users/admin/Desktop/NSAJIGWA/180904_1444_01_Regina.mp3time=1023600)

I: Mmh [00:17:06-8](http://localhost:2300/file=C:/Users/admin/Desktop/NSAJIGWA/180904_1444_01_Regina.mp3time=1026800)

R: Yes [00:17:07-0](http://localhost:2300/file=C:/Users/admin/Desktop/NSAJIGWA/180904_1444_01_Regina.mp3time=1027000)

I: What about treatment involving breast removal [00:17:07-8](http://localhost:2300/file=C:/Users/admin/Desktop/NSAJIGWA/180904_1444_01_Regina.mp3time=1027800)

R: Mmh, I ever heard about it from people. Yes, and even doctor spoke about it [00:17:14-6](http://localhost:2300/file=C:/Users/admin/Desktop/NSAJIGWA/180904_1444_01_Regina.mp3time=1034600)

I: Did the doctor tell you about it? [00:17:16-3](http://localhost:2300/f)

R: Yes [00:17:17-4](http://localhost:2300/file=C:/Users/admin/Desktop/NSAJIGWA/180904_1444_01_Regina.mp3time=1037400)

I: And weren’t you scared if that was the kind of treatment [00:17:18-2](http://localhost:2300/file=C:/Users/admin/Desktop/NSAJIGWA/180904_1444_01_Regina.mp3time=1038200)

R: Fear must be there [00:17:23-6](http://localhost:2300/file=C:/Users/admin/Desktop/NSAJIGWA/180904_1444_0)

I:But it never made you feel that the swelling was a normal thing [00:17:27-2](http://localhost:2300/file=C:/Users/admin/Desktop/NSAJIGWA/180904_1444_01_Regina.mp3time=1047200)

R: So, I weighed, If I pretend that it’s a normal thing or not a normal thing, because the society knows about it and its side effects and how it spreads, I knew that it could be breast as per today but tomorrow could advance to lung. What if it spreads to liver, if it wasn’t something that spread then it could have been better [00:17:47-8](http://localhost:2300/file=C:/Users/admin/Desktop/NSAJIGWA/180904_14)

I: And what about during the treatment phase, didn’t you use local herbs for treatment since we meet with people undergoing treatment other than hospital-based treatment, so how about yourself, how was it? [00:18:02-8](http://localhost:2300/file=C:/Users/admin/Desktop/NSAJIGWA/180904_1444_01_Regina.mp3time=1082800)

R: For my side I also did it, I used both hospital-based treatment and non-hospital based. After not knowing my fate from here. Because whenever you go there are a lot of procedures on treatment and becomes difficult for a normal civilian to afford hence a lot of people will die due to expenses and someone could be from upcountry and becomes difficult to afford accommodation. And when you arrive here there would be investigations that would take up to two months, so someone would wait for all that while only waiting for an investigation, Most people will die and it is possible that they are already dead , because investigations take a long time. Again, there comes a time where you are to do radiotherapy so that imposes some other cost [00:18:56-6](http://localhost:2300/file=C:/Users/admin/Desktop/NSAJIGWA/180904_1444_01_Regina.mp3time=1136600)

I: Mmh [00:19:01-4](http://localhost:2300/file=C:/Users/admin/Desktop/NSAJIGWA/180904_1444_01_Regina.mp3time=1141400)

R: Yes, so it is possible that there a lot of people from villages and upcountry [00:19:01-7](http://localhost:2300/file=C:/Users/admin/Desktop/NSAJIGWA/180904_1444_01_Regina.mp3time=1141700)

I: Abscond treatment, [00:19:05-8](http://localhost:2300/file=C:/Users/admin/Desktop/NSAJIGWA/180904_1444_01_Regina.mp3time=1145800)

R: Yes, they abscond treatment , they just decide that come what may, so they run to an alternative therapy and lastly they die [00:19:06-9](http://localhost:230)

I: Did you get alternative therapy from ----- or? [00:19:13-6](http://localhost:2300/file=C:/Users/admin/Desktop/NSAJIGWA/180904_1444_01_Regina.mp3time=1153600)

R: Yes, here in ----- [00:19:16-9](%22ht)

I: Is it available in -----? [00:19:18-1](http://localhost:2300/file=C:/Users/admin/Desktop/NSAJIGWA/180904_1444_01_Regina.mp3time=1158100)

R: Because while I was there, I depended on you mostly. When I arrived here, for two months there were no medications, how could I survive? By the way I am thankful to the blood pressure because I could have been dead by now due to hypertension [00:19:23-3](http://localhost:2300/file=C:/Users/admin/Desktop/NSAJIGWA/180904_1444_01_Regina.mp3time=1163300)

I: No, [00:19:34-1](http://localhost:2300/file=C:/Users/admin/Desktop/NSAJIGWA/180904_1444_01_Regina.mp3time=1174100)

R: Yes, because I have never gotten such kind of illness [00:19:35-6](%22)

I: Are you normally hypertensive since then? [00:19:39-7](http://localhost:2300/file=C:/Users/admin/Desktop/NSAJIGWA/180904_1444_01_Regina.mp3time=1179700)

R: No, not since then [00:19:41-2](http://localhost:2300/file=C:/Users/admin/Desktop/NSAJIGWA/180904_1444_01_Regina.mp3time=1181200)

I: Or it started during this time [00:19:43-0](http://localhost:2300/file=C:/Users/admin/Desktop/NSAJIGWA/180904_1444_01_Regina.mp3time=1183000)

R: There was a time that I fell ill in year -----, but later on I controlled it. It erupted again this time [00:19:52-6](http://localhost:2300/file=C:/Users/admin/Desktop/NSAJIGWA/180904_1444_01_Regina.mp3time=1192600)

I: Do you have relatives in -----? [00:19:55-2](http://localhost:2300/fil)

R: I do have them? [00:19:57-4](http://localhost:2300/file=C:/Users/admin/Desktop/NSAJIGWA/180904_1444_01_Regina.mp3time=1197400)

I: Mmh [00:19:58-8](http://localhost:2300/file=C:/Users/admin/Desktop/NSAJIGWA/180904_1444_01_Regina.mp3time=1198800)

R: Yes [00:19:59-3](http://localhost:2300/file=C:/Users/admin/Desktop/NSAJIGWA/180904_1444_01_Regina.mp3time=1199300)

I: Because I wanted to know on the type of alternative therapy that you used to get, I would like to know them [00:19:59-7](http://localhost:2300/file=C:/Users/admin/Desktop/NSAJIGWA/180904_1444_01_Regina.mp3time=1199700)

R: You should just make efforts on your hospital treatment, you guys are too slow , you are taking too long, it is a very dangerous disease but I don’t know you only want us to die. The treatment is too slow and I do not know why, just imagine since March to today that’s when am getting treatment. [00:20:23-0](http://local)

I: There are things that we have truly observed, that we made delays [00:20:31-4](http://localhost:2300/file=C:/Users/admin/Desktop/NSAJIGWA/180904_1444_01_Regina.mp3time=1231400)

R: Yes, you really delay us [00:20:37-5](http://localhost:2300/file=C:/Users/admin/Desktop/NSAJIGWA/180904_1444_01_Regina.mp3time=12)

I: And that is why we are trying to get information from patients and doing some research [00:20:38-7](http://localhost:2300/file=C:/Users/admin/Desktop/NSAJIGWA/180904_1444_01_Regina.mp3time=1238700)

R: So just imagine I do have a relative but I have become a burden to him, I do have a family and with my condition I could engage in any activity and earn money because it is not a disease that can make me sleep all day. But if you are at your host’s place you can’t be productive, and all what you are waiting for are cancer results [00:20:57-5](http://localhost:2300/file=C:/Users/admin/Desktop/NSAJIGWA/180904_1444_01_)

I: And with treatment apart from local herbs, is there anything that you have done to help you with treatment [00:21:26-7](http://localhost:2300/file=C:/Users/admin/Desktop/NSAJIGWA/180904_1444_01_Regina.mp3time=1286700)

R: No, there isn’t, first before going for alternative therapy is after seeing that there is no help from you, so if there is no help I wonder if it will come the time I am dying because ever since I reported to hospital in -----, results are taking too many procedures to be out, why is it so? My thinking is that you should educate people but your people should also be saying [00:21:56-4](%22http://)

I: Mmh [00:22:01-2](http://localhost:2300/file=C:/Users/admin/Desktop/NSAJIGWA/180904_1444_01_Regina.mp3time=1321200)

R: Yes, for example if someone could have alerted me to rush for treatment earlier, unfortunately I went to hospital and someone told me that it wasn’t a problem. Yes, so you become less worried and you proceed with your works , and mind you it is a medical doctor who told me that It wasn’t a problem and I didn’t go to a witchdoctor, I only went to hospital for that [00:22:08-2](http://localhost:2300/file=C:/Users)

I: Mmh [00:22:20-8](http://localhost:2300/file=C:/Users/admin/Desktop/NSAJIGWA/180904_1444_01_Regina.mp3time=1340800)

R: Yes, so I do not understand whether he wasn’t understanding or what. A doctor saying that this is not a problem [00:22:27-3](http://localhost:2300/file=C:/Users/admin/Desktop/NSAJIGWA/180904_1444_01_Regina.mp3time=13)

I: Mmh [00:22:29-8](http://localhost:2300/file=C:/Users/admin/Des)

R: Yes, so I am just human what then can I say, I would say maybe it’s just due to frictional forces from the fracture and POP [00:22:30-5](http://localhost:2300/file=C:/Users/admin/Desktop/NSAJIGWA/180904_1444_01_Regina.mp3time=1350500)

I: After having a fracture? [00:22:36-6](http://localhost:2300/file=C:/Users/admin/Desktop/NSAJIGWA/180)

R: Yes, because I had POP for long, it’s possible that if it was on this side then he couldn’t have said anything, this other hand is the one that has got the effect. Now a problem has occurred while my hand is still aching to moment [00:22:48-3](http://localhost:2300/file=C:/Users/admin/Desktop/NSAJIGWA/180904_1444_01_Regina.mp3time=1368300)

I: Is it still aching? [00:22:52-5](http://localhost:2300/file=C:/Users/admin/Desktop/NSAJIGWA/180904_1444_01_Regina.mp3time=1372500)

R: So personally that is what is confusing me, if it was this side I guess I could have left him because even those who fail are there [00:22:52-8](http://l)

I: Mmh [00:22:58-8](http://localhost:2300/file=C:/Users/admin/Desktop/NSAJIGWA/180904_1444_01_Regina.mp3time=1378800)

R: Yes [00:22:59-3](http://localhost:2300/file=C:/Users/admin/Desktop/NSAJIGWA/180904_1444_01_Regina.mp3time=1379300)

I: In your family is there anyone who got a similar problem? [00:23:00-3](http://localhost:2300/file=C:/Users/admin/Desktop/NSAJIGWA)

R: No one [00:23:03-6](http://loc)

I: No one? [00:23:04-2](http://localhost:2300/file=C:/Users/admin/Desktop/NSAJIGWA/180904_1444_01_Regina.mp3time=1384200)

R: Yes [00:23:05-0](http://localhost:2300/file=C:/Users/admin/Desktop/NSAJIGWA/180904_1444_01_Regina.mp3time=1385000)

I: Maybe there are relatives or your people that you heard about [00:23:05-6](http://localhost:2300/file=C:/Users/admin/Desktop/NSAJIGWA/180904_1444_01_Regina.mp3time=1385600)

R: I have never heard [00:23:08-2](http://localhost:2300/file=C:/Users/admin/Desktop/NSAJIGWA/180904_1444_01_Regina.mp3time=1388200)

I: How did you know about cancer disease? [00:23:10-6](http://localhost:2300/file=C:/Users/admin/Desktop/NSAJIGWA/180904_1444_01_Regina.mp3time=1390600)

R: I heard about breast cancer from one of my customers , he underwent mastectomy a year before last year [00:23:14-3](http://localhost:2300/file=C:/Users/admin/Desktop/NSAJIGWA/180904_1444_01_Regina.mp3time=1394300)

I: Mmh [00:23:26-0](http://localhost:2300/file=C:/Users/admin/Desktop/NSAJIGWA/180904_1444_01_Regina.mp3time=1406000)

R: Yes, she was my customer, but on uterine cancer I witnessed it from three people [00:23:27-0](http://localhost:2300/file=)

I: Mmh [00:23:35-9](http://localhost:2300/file=C:/Users/admin/Desktop/NSAJIGWA/180904_1444_01_Regina.mp3time=1415900)

R: Yes [00:23:36-4](http://localhost:2300/file=C:/Users/admin/Desktop/NSAJIGWA/180904_1444_01_Regina.mp3time=1416400)

I: And you said that your customer had a similar problem? [00:23:37-0](http://localhost:2300/file=C:/Users/admin/Desktop/NSAJIGWA/180904_1444_01_Regina.mp3time=1417)

R: Yes [00:23:41-1](http://localhost:2300/file=C:/Users/admin/Desktop)

I: Did she undergo mastectomy? [00:23:41-5](http://localhost:2300/file=C:/Users/admin/Desktop/NSAJIGWA/180904_1444_01_Regina.mp3time=1421500)

R: Yes, she was operated on [00:23:42-6](http://localhost:2300/file=C:/Users/admin/Desktop/NSAJIGWA/180904_1444_01_Regina.mp3time=1422600)

I: Were the treatments same to these? [00:23:44-2](http://localhost:2300/file=C:/Users/admin/Desktop/NSAJIGWA/180904_14)

R: She did both, hospital based and alternative therapy [00:23:50-7](http://localhost:2300/file=C:/Users/admin/Desktop/NSAJIGWA/180904_1444_01_Regina.mp3time=1430700)

I: Mmh [00:23:54-8](http://localhost:2300/file=C:/Users/admin/Desktop/NSAJIGWA/180904_1444_01_Regina.mp3time=1434800)

R: And she is doing well to date [00:23:54-8](http://localhost:2300/fi)

I: Okay [00:23:56-7](http://localhost:2300/file=C:/Users/admin/Desktop/NSAJIGWA/180904_1444_01_Regina.mp3time=1436700)

R: Yes, but the rest that I do know about have died [00:23:57-9](%22htt)

I: And were they receiving hospital treatment? [00:24:04-2](http://localhost:2300/file=C:/Users/admin/Desktop/NSAJIGWA/180904_1444_01_Regina.mp3time=1444200)

R: Yes, maybe if someone absconds willingly, for example for uterine cancer many people from villages die , I was thinking if the government would take a step of educating people out there. [00:24:06-9](http://localhost:2300/file=C:/Users/admin/Desktop/NSAJIGWA/180904_1444_01_Regina.mp3time=1446900)

I: Are people not aware? [00:24:21-5](http://localhost:2300/file=C:/Users/admin/Desktop/NSAJIGWA/18)

R: Yes, I don’t think so [00:24:22-5](http://localhost:2300/file=C:/Users/admin/Desktop/NSAJIGWA/180904_1444_01_Regina.mp3time=1462500)

I: (Inaudible) [00:24:23-0](http://localhost:2300/file=C:/Users/admin/Desktop/NSAJIGWA/180904_1444_01_Regina.mp3time=1463000)

R: Yes, or maybe people haven’t understood well about cancer disease since they say it’s better you suffer from HIV/AIDS because cancer disease is too silent, it is not so obvious at first and not being perceived as a threat. [00:24:24-1](http://localhost:2300/file=C:/Users/admin/Desktop/NSAJIGWA/180904_1444_01_Regina.mp3tim)

I: Very true [00:24:35-7](http://localhost:2300/file=C:/Users/a)

R: Yes, for now you better suffer from HIV/AIDS that cancer disease , and many people do have cancer disease and again maybe I do not know if people are educated on causes of cancer disease [00:24:36-1](http://localhost:2300/file=C:/Users/admin/Desktop/NSA)

I: Mmh [00:24:45-9](http://l)

R: Yes, knowing at least on what one should use or should do, because I attended at -----one day, if people from villages could get such education they could be knowledgeable [00:24:46-5](http://localhost:2300/file=C:/Users/admin/)

I: Mmh [00:24:57-9](http://localhost:2300/file=C:/Users/admin/Desktop/NSAJIGWA/180904_1444_01_Regina.mp3time=1497900)

R: Yes, from Monday to Thursday at ----- [00:24:58-3](http://localhost:2300/file=C:/Users/admin/Desktop/NSAJIGWA/180904_1444_01_Regina.mp3time=1498300)

I: They do educate [00:25:02-5](http://localhost:2300/file=C:/Users/adm)

R: Yes, they do educate people before they start giving treatment [00:25:03-1](http://localhost:2300/file=C:/Users/admin/Desktop/NSAJIGWA/180904_1444_01_Regina.mp3time=1503100)

I: Okay [00:25:04-5](http://localhost:2300/file=C:/Users/admin/Desktop/NSAJIGWA/180904_1444_01_Regina.mp3time=1504500)

R: So that is in -----, would people from village be knowledgeable? [00:25:05-2](http://localhost:2300/file=C)

I: So, you also got a better understanding on cancer disease [00:25:07-7](http://localhost:2300/file=C:/Users/admin/Desktop/NSAJIGWA/180904_1444_01_Regina.mp3time=1507700)

R: Yes, a lot of understanding, [00:25:11-3](http://localhost:2300/file=C:/Users/admin/Desktop/NSAJIGWA/180904_1444_01_Regina.mp3time=1511300)

I: So after being sick then it became much better in terms of knowledge acquired on cancer [00:25:12-1](http://localhost:2300/file=C:/Users/admin/Desktop/NSAJIGWA/180904_1444_01_Regina.mp3time=1512100)

R: Yes, since I am only here I have nowhere to go [00:25:13-6](http://localhost:2300/file=C:/Users/admin/Desktop/NSAJIGWA/180904_1444_01_Regina.mp3time=1513600)

I: Mmh [00:25:16-0](http://localhost:2300/file=C:/Users/admin/Desktop/NSAJIGWA/180904_1444_01_Regina.mp3tim)

R: Yes, so someone said that we should go to ----- because they do give education on cancer disease , then we went, if such kind of information could be spread to most people a lot of Tanzanians could be [00:25:16-4](http://localhost:2300/file=C:/Users/admin/Desktop/NSAJIGWA/180904_1444)

I: Mmh [00:25:25-4](http://localhost:2300/f)

R: Unfortunately, you are only waiting for a person from ----- to educate, so do you think a person from ----- would wait for a person from -----? [00:25:25-7](http://localhost:2300/file=C:/Users/admin/Desktop/NSAJIGWA/180904_1444_01_Reg)

I: It’s a challenge [00:25:30-1](http://localhost:230)

R: Why shouldn’t such kind of information be on health centres and reach out to people out there at least to make them aware [00:25:30-5](http://localhost:2300/file=C:/Users/admin/Desktop/NSAJIGWA/180904_1444_01_Regina.mp3time=1530500)

I: Making them aware to at least get to know the symptoms [00:25:36-5](%22)

R: Not only symptoms, even before one gets symptoms, that on what to eat because sometimes is on what we eat [00:25:40-2](http://localhost:2300/file=C:/Users/admin/Desktop/NSAJIGWA/180904_1444_01_Regina.mp3time=1540200)

I: Mmh [00:25:46-6](http://localhost:2300/file=C:/Users/admin/Desktop/NSAJIGWA/18)

R: Yes, from the food that we eat, someone should be educated on, plus the symptoms generally should be educated on everything and the health sector should take it into a serious note. Why is it that with HIV/AIDS you really educated people? When HIV/AIDS first got into this country you educated on people and so it was on one’s decision whether to abstain or to engage. But with cancer disease I do not know if there such a thing, or maybe it is because I do not attend to clinics [00:26:05-7](http://localhost:2300/file=C:/Users/admin/Desktop/N)

I: The services are mostly delivered through radio so we are also asking ourselves as to why they are not reaching out to people [00:26:09-8](http://localhost:2300/file=C:/Users/admin/Desktop/NSAJIGWA/180904_1444_01_Regina.mp3time=1569800)

R: Mmh [00:26:18-8](%22http)

I: But again I have seen that you are among the few who sees symptoms and starts making follow-ups on treatment , what do you think was the cause that made you seek for medical care right after you saw the swelling [00:26:19-3](%22http)

R: Me seeing the swelling and starting to look for medical care was after seeing my customer [00:26:35-1](http://localhost:2300/file=C:/Users/admin/Desktop/NSAJIGWA/180904_1444_01_Regina.mp3time=1595100)

I: Mmh [00:26:41-7](http://localhost:2300/file=C:/Users/admin/Desktop/NSAJIGWA/180904_1444_01_Regin)

R: But I wasn’t knowledgeable about breast cancer [00:26:42-0](http://localhost:2300/file=C:/Users/admin/Desktop/NSAJIGWA/180904_1444_01_Regina.mp3time=1602000)

I: Mmh [00:26:45-3](http://localhost:2300/file=C:/Users/admin/Desktop/NSAJIGWA/180904_1444_01_Regina.mp3time=1605300)

R: Yes, I wasn’t knowledgeable and when I saw the swelling and didn’t understand it, I just decided to go to hospital, and by then my husband wasn’t there and I didn’t ask anyone at home about it, I had to ask the doctor so that it doesn’t turn out to being a problem. Surprisingly, we all had similar thoughts that it is not a problem, he examined me and told me that it is not a problem so I went out with joy, not knowing that it was a problem. [00:27:00-1](http://localhost:2300/file=C:/Users/admin/Desktop/NSAJIGWA/180904_1444_01_Regina.mp3time=1620100)

I: But at least you met with your customer who had a similar disease [00:27:15-8](http://localhost:2300/file=C:/Users/admin/Desktop/NSAJIGWA/180904_1444_01_Regina.mp3time=1635800)

R: Yes, it alerted me to some extent that she said it was breast cancer let me rush to check, but the one who said that it is not a problem [00:27:22-1](http://localhost:2300/file=C:/Users/admin/Desktop/NSAJIGWA/180904_1444_01_Regina.mp3time=1642100)

I: And, we have seen that cancer disease treatment are different from other disease treatment, do you think such kind of treatment is a challenge that makes people not get treatment on time? [00:27:33-3](http://localhost:2300/file=C:/Users/admin/Desktop/NSAJIGWA/180904_1444_01_Regina.mp3time=1653300)

R: It is a challenge [00:27:50-7](http://localhost:2300/file=C:/Users/admin/Desktop/NSAJIGWA/180904_1444_01_Regina.mp3time=1670700)

I: Mostly in what manner [00:27:53-5](http://localhost:2300/file=C:/Users/ad)

R: Suppose a normal civilian is to come from village to ----- waiting for radiotherapy for different cycles, and that civilian is just a peasant who isn’t no longer productive and he/she has not relatives in Dar so with all those expenses there comes a time someone absconds treatment [00:27:56-6](http://localhost:2300/file=C:/Users/admin/Desktop/NSAJIGWA/180904_1444_01_Regina.mp3time=1676600)

I:Mmh [00:28:09-3](http://localhost:2300/file=C:/Users/admin/Desktop/NSAJIGWA/180904_1444_01_Regina.mp3time=1689300)

R: So not every person has a relative here in ----- [00:28:09-7](http://localhost:2300/file=C:/Users/admin/Desktop/NSAJIGWA/180904_1444_01_Regina)

I:Mmh [00:28:13-2](http://localhost:2300/file=C:/Users/a)

R: And not all can tolerate to that extent [00:28:13-8](http://localhost:2300/file=C:/Users/admin/Desktop/NSAJIGWA/180904_1444_01_Regina.mp3time=1693800)

I:Yes [00:28:16-7](http://localhost:2300/file=C:/Users/admin/Desktop/NSAJIGWA/180904_1444_01_Regina.mp3time=1696700)

R: For my case, I came here since end of June till date [00:28:17-3](http://localhost:2300/file=C:/Users/admin/Desktop/NSAJIGWA/)

I: What improvement should be done to this group of people who do not have relatives in ----- [00:28:27-5](http://localhost:2300/file=C:/Users/admin/Desktop/NSAJIGWA/180904_1444_01_Regina.mp3time=1707500)

R: Just as the government does to other things then should also consider them. If the government would construct them a place to reside in, they should be prioritized , imagine someone coming from ----- , because they say that treatment is from only three places for the whole country, so maybe the zonal hospitals should be enabled to do perform radiotherapy and some other cancer treatments [00:28:43-0](http://localhost:2300/file=C:/Users/admin/Desktop/NSAJIGWA/180904_1444_01_Regina.mp3time=1723000)

I: Mmh [00:28:59-7](http://localhost:2300/file=C:/Users/admin/Desktop/NSAJIGWA/180904_1444_01_Regina.mp3time=1739700)

R: Yes, so that people from each zone would have a common centre for treatment. For example for people from ----- and ----- maybe they get treated at -----, but I heard for a whole country its only in here [00:29:04-9](http://localhost:2300/file=C:/Users/admin/Desktop/NSAJIGWA/180904_1444_01_Regina.mp3time=1744900)

I: Only here [00:29:12-0](http://localhost:2300/file=C:/Users/admin/Desktop/NSAJIGWA/180904_1444_01_Regina.mp3time=1752000)

R: Yes [00:29:12-4](http://localhost:2300/file=C:/Users/admin/Desktop/NSAJIGWA/180904_1444_01_Regina.mp3time=1752400)

I: ----- at most [00:29:13-4](http://localhost:2300/file=C:/Users/admin/Desktop/NSAJIGWA/180904_1444_01_Regina.mp3time=1753400)

R: Eeh, -----[00:29:15-1](http://localhost:2300/file=C:/Users/admin/Desktop/NSAJIGWA/180904_1444_01_Regina.mp)

I: Have you ever done investigation like mammography?, we do have an investigation that detects lesions at early stages , have you personally ever done it [00:29:16-0](http://localhost:2300/file=C:/Users/admin/Desktop/NSAJIGWA/180904_1444_01_Regina.mp3time=1756000)

R: I am first hearing it from you [00:29:30-5](http://localhost:2300/file=C:/Users/admin/Desktop/NSAJIGWA/180904)

I: Apart from ultrasound, where did you do ultrasound? [00:29:31-9](http://localhost:2300/file=C:/Users/admin/Desktop/NSAJIGWA/180904_1444_01_Regina.mp3time=1771900)

R: So, how is that? [00:29:35-7](http://localhost:2300/file=C:/Users/admin/Desktop/NSAJIGWA/180904_1444_01_Regina.mp3time=1775700)

I: It is an investigation that examines [00:29:39-2](http://localhost:2300/file=C:/Users/admin/Deskto)

R: So, you only go there? [00:29:42-6](http://localhost:2300/file=C:/Users/admin/Desktop/NSAJIGWA/180904_1444_01_Regina.mp3time=1782600)

I: You go and check even when the swelling is small [00:29:44-0](http://localhost:2300/file=C:/Users/admin/Desktop/NSAJIGWA/180904_1444_01_Regina.mp3time=1784000)

R: Mmh [00:29:47-4](http://localhost:2300/file=C:/Users/admin/Desktop/NSAJIGWA/180904_1444_01_Regina.mp3time=1787400)

I: Even when you cannot feel it by touch [00:29:47-8](http://localhost:2300/file=C:/Users/admin/Desktop/NSAJIGWA/180904_1444_01_Regina.mp3time=1787800)

R: Mmh [00:29:50-0](http://localhost:2300/file=C:/Users/admin/Desktop/NSAJIGWA/180904_1444_01_Regina.mp3time=1790000)

I: It only checks if someone has a problem, it does early screening [00:29:50-4](http://localhost:2300/file=C:/Users/admin/Desktop/NSAJIGWA/180904_1444_01_Regina.mp3time=179)

R: Where is it found? [00:29:58-9](http://localhost:2300/file=C:)

I: It is in places like ----- [00:30:01-4](http://localhost:2300/file=C:/Users/admin/Desktop/NSAJIGWA/180904_1444_01_Regina.mp3time=1801400)

R: Do ----- have it? [00:30:04-5](http://localhost:2300/file=C:/Users/admin/Desktop/NSAJIGWA/180904_1444_01_Regina.mp3time=1804500)

I: Mmh, I know ----- has it [00:30:05-4](http://localhost:2300/file=C:/Users/admin/Desktop/NSAJIGWA/180904_1444_01_R)

R: What about here? [00:30:07-0](http://localhost:)

I: I guess it is also here but am not so certain [00:30:09-4](http://localhost:2300/file=C:/Users/admin/Desktop/NSAJIGWA/180904_1444_01_Regina.mp3time=1809400)

R: So let’s say that it is found in some hospitals [00:30:12-2](http://localhost:2300/file=C:/Users/admin/Desktop/NSAJIGWA/180904_1444_)

I: Yes, it is found in some hospitals [00:30:14-8](http://localhost:2300/file=C:/Users/admin/Desktop/NSAJIGWA/180904_1444_01_Regina.mp3time=1814800)

R: So, you only go and tell them, [00:30:15-9](http://localhost:2300/file=C:/Users/admin/Desktop/NSAJIGWA/180904_1444_01_Regina.mp3time=1815900)

I: That you came for checkup, screening [00:30:18-2](http://localhost:2300/file=C:/Users/admin/Desktop/NSAJIGWA/180904_1444_01_Regina.mp3time=1818200)

R: Does it screen any type of cancer at early stages [00:30:20-0](http://localhost:2300/file=C:/Users/admin/Desktop/NSAJIGWA/180904_1444_01_Regina.mp3time=1820000)

I: Mostly for breast [00:30:23-0](http://localhost:2300/file=C:/Users/admin/Desktop/NSAJIGWA/180904_1444_01_Regina.mp3time=1823000)

R: For breast? [00:30:24-4](http://localhost:2300/file=C:/Users/admin/De)

I: Yes, that is for breast [00:30:25-1](http://localhost:2300/file=C:/Users/admin/Desktop/NSAJIGWA/180904_1444_01_Regina.mp3time=1825100)

R: I never knew about it, I am hearing it first from you [00:30:26-3](http://localhost:2300/file=C:/Users/admin/Desktop/NSAJIGWA/180904_1444_01_Regina.mp3time=1826300)

I: Okay [00:30:32-1](http://localhost:2300/file=C:/Users/admin/Desktop/NSAJIGWA/180904_1444_01_Regina.mp3time=1832100)

R: Yes 00:30:32-5

I: Okay -----, I think I mostly wanted to learn about challenges and reasons that makes a patient delay seeking for health and getting treatment, because I spoke to you and you have given me the source to many that is happening [00:30:51-4](http://localhost:2300/file=C:/Users/admin/Desktop/NSAJIGWA/180904_1444_01_Regina.mp3time=1851400)

R: Mmh [00:31:08-3](http://localhost:2300/file=C:/Users/admin/Desktop/NSAJIGWA/180904_1444_01_Regina.mp3time=1868300)

I: We will work on them [00:31:08-9](http://localhost:2300/file=C:/Users/admin/Desktop/NSAJIGWA/180904_1444_01_Regina.mp3time=1868900)

R: Okay, [00:31:12-6](http://localhost:2300/file=C:/Users/admin/Desktop/NS)

I: They might not be fitting for you since you already have a problem but as we get these information they become helpful [00:31:13-2](http://localhost:2300/file=C:/Users/admin/Desktop/NSAJIGWA/180904_1444_01_Regina.mp3time=1873200)

R: Okay [00:31:22-4](http://localhost:2300/file=C:/Users/admin/Desktop/NSAJIGWA/180904_1444_01_Regina.mp3time=1882400)

I: Maybe if you have something else that you want to add, [00:31:23-0](http://localhost:2300/file=C:/Users/admin/Desktop/NSAJIGWA/180904_1444_01_Regina.mp3time=1883000)

R: Are you referring to me? [00:31:27-4](http://localhost:2300/file=C:/Users/admin/Desktop/NSAJIGWA/180904_1444_01_Regina.mp3time=1887400)

I: Yes [00:31:28-9](http://localhost:2300/file=C:/Users/admin/Desktop/N)

R: Personally to where I have reached I do not know the stage of the disease , is it a secret or are they going to tell me some other day, because I do not know my stage and I heard that there is stage one to four [00:31:29-5](http://localhost:2300/file=C:/Users/admin/Desktop/NSAJI)

I: Mmh [00:31:42-7](http://lo)

R: What are those stages? [00:31:43-2](http://localhost:2300/file=C:/Users/admin/Desktop/NSAJIGWA/180904_1444_01_Regina.mp3time=1903200)

I: I guess they will tell you, but it’s all about the stage that the disease has reached [00:31:49-6](http://localhost:2300/file=C:/Users/admin/Desktop/NSAJIGWA/180904_1444_01_Regina.mp3time=1909600)

R: Disease stage? [00:31:53-2](http://localhost:2300/file=C:/Users/admin/Desktop/NSAJIGWA/180904_1444_01_Regina.mp3time=1913200)

I: Yes [00:31:54-0](http://localhost:2300/file=C:/Users/admin/Desktop/NSAJIGWA/180904_1444_01_Regina.mp3time=1914000)

R: Mmh, okay [00:31:54-3](%22)

I: Okay, thank you ----- [00:31:57-8](http://localhost:2300/file=C:/Users/admin/Desktop/NSAJIGWA/180904_1444_01_Regina.mp3time=1917800)

R: I only request that you keep on helping others 00:31:59-7

**CASE 11**

I: Mmh sister what’s your name? [00:00:03-7](http://localhost:2300/file=C:/Users/USER/Desktop/EXTRAS/godfrey/New%20folder/180905_1032_Amina.mp3time=3700)

R: My name is ----- [00:00:05-1](http://localhost:2300/file=C:/Users/USER/Desktop/EXTRAS/godfrey/New%20folder/180905_1032_Amina.mp3time=5100)

I: Where are you from -----? [00:00:06-7](http://localhost:2300/file=C:/Users/USER/Desktop/EXTRAS/godfrey/New%20folder/180905_1032_Amina.mp3time=6700)

R: I am from -----[00:00:08-4](http://localhost:2300/file=C:/Users/USER/Desktop/EXTRAS/godfrey/New%20folder/180905_1032_Amina.mp3time=8400)

I: Where? [00:00:08-8](http://localhost:2300/file=C:/Users/USER/Desktop/EXTRAS/godfrey/New%20folder/180905_1032_Amina.mp3time=8800)

R: ----- because they say that after you cross the railway, you’re then heading to ----- street. [00:00:17-2](http://localhost:2300/file=C:/Users/USER/Desktop/EXTRAS/godfrey/New%20folder/180905_1032_Amina.mp3time=17200)

I: Ahaa and how old are you? [00:00:20-3](http://localhost:2300/file=C:/Users/USER/Desktop/EXTRAS/godfrey/New%20folder/180905_1032_Amina.mp3time=20300)

R: -----[00:00:21-3](http://localhost:2300/file=C:/Users/USER/Desktop/EXTRAS/godfrey/New%20folder/180905_1032_Amina.mp3time=21300)

I: And do you have a family, do you have children, do you have a husband? [00:00:25-1](http://localhost:2300/file=C:/Users/USER/Desktop/EXTRAS/godfrey/New%20folder/180905_1032_Amina.mp3time=25100)

R: I don’t have a husband or children. [00:00:26-8](http://localhost:2300/file=C:/Users/USER/Desktop/EXTRAS/godfrey/New%20folder/180905_1032_Amina.mp3time=26800)

I: Ahaa what is your occupation, what do you do? [00:00:29-8](http://localhost:2300/file=C:/Users/USER/Desktop/EXTRAS/godfrey/New%20folder/180905_1032_Amina.mp3time=29800)

R: At first, I was selling buns but ever since this problem happened to me, I have failed to knead the dough, I currently cannot do that, I have stopped. [00:00:38-7](http://localhost:2300/file=C:/Users/USER/Desktop/EXTRAS/godfrey/New%20folder/180905_1032_Amina.mp3time=38700)

I: And to what highest level have you studied? [00:00:43-3](http://localhost:2300/file=C:/Users/USER/Desktop/EXTRAS/godfrey/New%20folder/180905_1032_Amina.mp3time=43300)

R: I have studied to standard seven. [00:00:45-1](http://localhost:2300/file=C:/Users/USER/Desktop/EXTRAS/godfrey/New%20folder/180905_1032_Amina.mp3time=45100)

I: From where you live to the nearest hospital, how long does it take to reach there? [00:00:53-7](http://localhost:2300/file=C:/Users/USER/Desktop/EXTRAS/godfrey/New%20folder/180905_1032_Amina.mp3time=53700)

R: Fifteen minutes. [00:00:55-2](http://localhost:2300/file=C:/Users/USER/Desktop/EXTRAS/godfrey/New%20folder/180905_1032_Amina.mp3time=55200)

I: Do you go by bus or do you walk? [00:00:57-2](http://localhost:2300/file=C:/Users/USER/Desktop/EXTRAS/godfrey/New%20folder/180905_1032_Amina.mp3time=57200)

R: I walk. [00:00:58-2](http://localhost:2300/file=C:/Users/USER/Desktop/EXTRAS/godfrey/New%20folder/180905_1032_Amina.mp3time=58200)

I: Okay. Now, can you tell me how it started, how you noticed the problem until you came to the hospital. Can you narrate that to me? [00:01:09-7](http://localhost:2300/file=C:/Users/USER/Desktop/EXTRAS/godfrey/New%20folder/180905_1032_Amina.mp3time=69700)

R: My problem started as a small boil, it started down here. When I showed it to my relatives, they advised me to go to the hospital. And really that night when I slept it busted. [00:01:24-1](http://localhost:2300/file=C:/Users/USER/Desktop/EXTRAS/godfrey/New%20folder/180905_1032_Amina.mp3time=84100)

I: When was that? [00:01:26-4](http://localhost:2300/file=C:/Users/USER/Desktop/EXTRAS/godfrey/New%20folder/180905_1032_Amina.mp3time=86400)

R: It was in ----- something like that. [00:01:34-3](http://localhost:2300/file=C:/Users/USER/Desktop/EXTRAS/godfrey/New%20folder/180905_1032_Amina.mp3time=94300)

I: This year? [00:01:34-7](http://localhost:2300/file=C:/Users/USER/Desktop/EXTRAS/godfrey/New%20folder/180905_1032_Amina.mp3time=94700)

R: This year yes. [00:01:35-5](http://localhost:2300/file=C:/Users/USER/Desktop/EXTRAS/godfrey/New%20folder/180905_1032_Amina.mp3time=95500)

I: So, it is like five months have passed now. [00:01:37-8](http://localhost:2300/file=C:/Users/USER/Desktop/EXTRAS/godfrey/New%20folder/180905_1032_Amina.mp3time=97800)

R: Mmmh. After I saw that boil, I showed it to my relatives and they advised me to go to the hospital. I went to ----- hospital located at -----; I think it is very well known. After I reached at ----- hospital, I met the doctor and he checked me he saw the swelling which was like an abscess, and it surprised him. He gave me the transfer to -----. [00:02:08-1](http://localhost:2300/file=C:/Users/USER/Desktop/EXTRAS/godfrey/New%20folder/180905_1032_Amina.mp3time=128100)

I: Had it already busted? [00:02:11-1](http://localhost:2300/file=C:/Users/USER/Desktop/EXTRAS/godfrey/New%20folder/180905_1032_Amina.mp3time=131100)

R: Yes, it had already busted, after I headed to -----, I met the doctor and he told me to go and have an ultrasound or X-ray so that they can give me a transfer to come here so that they can get to know what it is. Unfortunately, I didn’t have enough money so I had to return home. After I returned home, I stayed there and the problem was growing big, and around June after the Eid festival I had to start the challenge once more. I went back to ----- and then to -----, I met the doctor on the same day and he told me to go and have a quick ultrasound. [00:03:04-6](http://localhost:2300/file=C:/Users/USER/Desktop/EXTRAS/godfrey/New%20folder/180905_1032_Amina.mp3time=184600)

I: Ultrasound again? [00:03:05-6](http://localhost:2300/file=C:/Users/USER/Desktop/EXTRAS/godfrey/New%20folder/180905_1032_Amina.mp3time=185600)

R: Yes [00:03:06-2](http://localhost:2300/file=C:/Users/USER/Desktop/EXTRAS/godfrey/New%20folder/180905_1032_Amina.mp3time=186200)

I: You had done it in the beginning, right? [00:03:07-3](http://localhost:2300/file=C:/Users/USER/Desktop/EXTRAS/godfrey/New%20folder/180905_1032_Amina.mp3time=187300)

R: I hadn’t done it, I failed to do it, as I didn’t have cash at hand, when I got cash there is when I went, after I went, I was checked and they gave me my results and I went back to the doctor. Doctor told me that it is seen that in my breast there are signs of cancer. So, they told me that since they don’t have further investigations to do, they have to give me transfer to -----. [00:03:33-8](http://localhost:2300/file=C:/Users/USER/Desktop/EXTRAS/godfrey/New%20folder/180905_1032_Amina.mp3time=213800)

I: What month was it? [00:03:36-1](http://localhost:2300/file=C:/Users/USER/Desktop/EXTRAS/godfrey/New%20folder/180905_1032_Amina.mp3time=216100)

R: What is this month? [00:03:39-2](http://localhost:2300/file=C:/Users/USER/Desktop/EXTRAS/godfrey/New%20folder/180905_1032_Amina.mp3time=219200)

I: This is September. [00:03:41-3](http://localhost:2300/file=C:/Users/USER/Desktop/EXTRAS/godfrey/New%20folder/180905_1032_Amina.mp3time=221300)

R: It is the last month. When I came to -----, we faced some challenges on the part of investigations, but on the day that I arrived there were no much challenges, I was checked by the doctor on the same day and he told me that I had to pay for the bed so that I can be admitted for further investigations. So, we were on the queue and paid fifteen thousand and we went in the ward and I was then admitted. [00:04:17-8](http://localhost:2300/file=C:/Users/USER/Desktop/EXTRAS/godfrey/New%20folder/180905_1032_Amina.mp3time=257800)

I: Do you pay cash or you use insurance? [00:04:19-8](http://localhost:2300/file=C:/Users/USER/Desktop/EXTRAS/godfrey/New%20folder/180905_1032_Amina.mp3time=259800)

R: I don’t have an insurance, we paid cash. [00:04:23-0](http://localhost:2300/file=C:/Users/USER/Desktop/EXTRAS/godfrey/New%20folder/180905_1032_Amina.mp3time=263000)

I: Okay. [00:04:23-9](http://localhost:2300/file=C:/Users/USER/Desktop/EXTRAS/godfrey/New%20folder/180905_1032_Amina.mp3time=263900)

R: we came inside. And honestly, I didn’t get any challenges. I met with a doctor whose name I don’t know until when I see him there is when I may know him. He attended me from the day I was admitted, at night until five or six in the morning he took a piece of tissue and discharged me home to go and wait for the results. When we got the results, I was told to have a chest x-ray and ultrasound of the abdomen because the disease may spread to the chest or abdomen.

[00:05:11-9](http://localhost:2300/file=C:/Users/USER/Desktop/EXTRAS/godfrey/New%20folder/180905_1032_Amina.mp3time=311900)

I: So, there is when you started treatment? [00:05:15-2](http://localhost:2300/file=C:/Users/USER/Desktop/EXTRAS/godfrey/New%20folder/180905_1032_Amina.mp3time=315200)

R: I haven’t yet started treatment; I have come for the treatment process. We went there and did every investigation and I was given a date to return to the hospital, we came for the results and we went to see the doctor for anesthesia, and other doctors, and we came back here, and on Tuesday is when I got a bed, and I am currently waiting if it is today or tomorrow I will go for surgery of the swelling. Yes, so, I didn’t go through many challenges, I thank God for that.

[00:05:54-4](http://localhost:2300/file=C:/Users/USER/Desktop/EXTRAS/godfrey/New%20folder/180905_1032_Amina.mp3time=354400)

I: Okay, and when you saw it for the first time, you said it was like an abscess? [00:05:59-6](http://localhost:2300/file=C:/Users/USER/Desktop/EXTRAS/godfrey/New%20folder/180905_1032_Amina.mp3time=359600)

R: yes, like an abscess about this size. [00:06:01-6](http://localhost:2300/file=C:/Users/USER/Desktop/EXTRAS/godfrey/New%20folder/180905_1032_Amina.mp3time=361600)

I: Yes, and what did you do thereafter? [00:06:03-4](http://localhost:2300/file=C:/Users/USER/Desktop/EXTRAS/godfrey/New%20folder/180905_1032_Amina.mp3time=363400)

R: I didn’t to a thing, it was just a small abscess and we said to ourselves, “why has this abscess hardened, it has not ripened yet.” And until now when you touch the swelling, there are certain places that have hardened. [00:06:17-9](http://localhost:2300/file=C:/Users/USER/Desktop/EXTRAS/godfrey/New%20folder/180905_1032_Amina.mp3time=377900)

I: How long did it take until you told your relatives? [00:06:21-7](http://localhost:2300/file=C:/Users/USER/Desktop/EXTRAS/godfrey/New%20folder/180905_1032_Amina.mp3time=381700)

R: On the same day that I noticed I called my relatives, and informed them. We didn’t take it seriously because it wasn’t painful. I continued doing my activities, you know how we neglect things eeh. It wasn’t painful, it is just a normal abscess. [00:06:39-7](http://localhost:2300/file=C:/Users/USER/Desktop/EXTRAS/godfrey/New%20folder/180905_1032_Amina.mp3time=399700)

I: How long did you stay before you…. [00:06:43-7](http://localhost:2300/file=C:/Users/USER/Desktop/EXTRAS/godfrey/New%20folder/180905_1032_Amina.mp3time=403700)

R: Aaah we finished some months. [00:06:45-3](http://localhost:2300/file=C:/Users/USER/Desktop/EXTRAS/godfrey/New%20folder/180905_1032_Amina.mp3time=405300)

I: Months? [00:06:45-9](http://localhost:2300/file=C:/Users/USER/Desktop/EXTRAS/godfrey/New%20folder/180905_1032_Amina.mp3time=405900)

R: Mmmh yes, without knowing that the more we delay the more the problem worsens, but I thank God when they told me that it didn’t spread on the whole breast it is only confined to the muscles, it is something to really thank God for. I pray that it doesn’t spread so that I may get… [00:07:01-6](http://localhost:2300/file=C:/Users/USER/Desktop/EXTRAS/godfrey/New%20folder/180905_1032_Amina.mp3time=421600)

I: Did they tell you what stage it was? [00:07:04-0](http://localhost:2300/file=C:/Users/USER/Desktop/EXTRAS/godfrey/New%20folder/180905_1032_Amina.mp3time=424000)

R: They didn’t tell me the stage but they told me that my problem is in the muscles, so what they will do is they will do surgery outside the breast, where the problem is. [00:07:23-6](http://localhost:2300/file=C:/Users/USER/Desktop/EXTRAS/godfrey/New%20folder/180905_1032_Amina.mp3time=443600)

I: Okay. [00:07:25-5](http://localhost:2300/file=C:/Users/USER/Desktop/EXTRAS/godfrey/New%20folder/180905_1032_Amina.mp3time=445500)

R: Yes. [00:07:25-7](http://localhost:2300/file=C:/Users/USER/Desktop/EXTRAS/godfrey/New%20folder/180905_1032_Amina.mp3time=445700)

I: And before you started coming to the hospital, is there any kind of treatment you went through, the time you were waiting? [00:07:33-3](http://localhost:2300/file=C:/Users/USER/Desktop/EXTRAS/godfrey/New%20folder/180905_1032_Amina.mp3time=453300)

R: I have never used any kind of treatment apart from taking Panadol to relieve pain and until now I use Panadol. [00:07:40-0](http://localhost:2300/file=C:/Users/USER/Desktop/EXTRAS/godfrey/New%20folder/180905_1032_Amina.mp3time=460000)

I: Did you know anything about breast cancer? [00:07:42-3](http://localhost:2300/file=C:/Users/USER/Desktop/EXTRAS/godfrey/New%20folder/180905_1032_Amina.mp3time=462300)

R: I didn’t know but I used to hear about it. [00:07:46-0](http://localhost:2300/file=C:/Users/USER/Desktop/EXTRAS/godfrey/New%20folder/180905_1032_Amina.mp3time=466000)

I: You used to hear that... [00:07:48-8](http://localhost:2300/file=C:/Users/USER/Desktop/EXTRAS/godfrey/New%20folder/180905_1032_Amina.mp3time=468800)

R: I used to hear it from the television but I never understood but I am currently learning because I am having that problem now. [00:07:57-9](http://localhost:2300/file=C:/Users/USER/Desktop/EXTRAS/godfrey/New%20folder/180905_1032_Amina.mp3time=477900)

I: So, what do you think actually pushed you to go to the hospital and start treatment. Because maybe there are other things like other alternative sources of medication which are conducted in the streets, didn’t you think of such kind of stuffs? [00:08:12-0](http://localhost:2300/file=C:/Users/USER/Desktop/EXTRAS/godfrey/New%20folder/180905_1032_Amina.mp3time=492000)

R: I didn’t use alternative treatment. After I left home, my eldest brother told me to go to the hospital. What I did is, I went to the hospital, at -----. After he referred me to ----- there is when I and my brother went together to -----. [00:08:33-8](http://localhost:2300/file=C:/Users/USER/Desktop/EXTRAS/godfrey/New%20folder/180905_1032_Amina.mp3time=513800)

I: So, the greatest push was from the family or is there anything else that pushed you to go to the hospital? [00:08:39-1](http://localhost:2300/file=C:/Users/USER/Desktop/EXTRAS/godfrey/New%20folder/180905_1032_Amina.mp3time=519100)

R: I myself is a person who is so scared. I cannot lie to you, I am a very scared person, my family is the one that gave me the whole support until I reached here. [00:08:50-4](http://localhost:2300/file=C:/Users/USER/Desktop/EXTRAS/godfrey/New%20folder/180905_1032_Amina.mp3time=530400)

I: So, maybe you were also scared to tell them because I see that it took you a long time to seek medical treatment. So, I want to know what happened in between? [00:09:03-9](http://localhost:2300/file=C:/Users/USER/Desktop/EXTRAS/godfrey/New%20folder/180905_1032_Amina.mp3time=543900)

R: The challenge is money. [00:09:04-5](http://localhost:2300/file=C:/Users/USER/Desktop/EXTRAS/godfrey/New%20folder/180905_1032_Amina.mp3time=544500)

I: It is mostly money? [00:09:05-2](http://localhost:2300/file=C:/Users/USER/Desktop/EXTRAS/godfrey/New%20folder/180905_1032_Amina.mp3time=545200)

R: Mmmh, we are not the challenge, it is money. [00:09:08-6](http://localhost:2300/file=C:/Users/USER/Desktop/EXTRAS/godfrey/New%20folder/180905_1032_Amina.mp3time=548600)

I: Or knowing that this problem has… [00:09:10-5](http://localhost:2300/file=C:/Users/USER/Desktop/EXTRAS/godfrey/New%20folder/180905_1032_Amina.mp3time=550500)

R: I knew the problem of cancer is a disease that is so scary in Tanzania or the world. It is the disease that is mostly feared, but to be honest, money was the greatest challenge. [00:09:32-0](http://localhost:2300/file=C:/Users/USER/Desktop/EXTRAS/godfrey/New%20folder/180905_1032_Amina.mp3time=572000)

I: And you told me that you came for the ultrasound? [00:09:37-0](http://localhost:2300/file=C:/Users/USER/Desktop/EXTRAS/godfrey/New%20folder/180905_1032_Amina.mp3time=577000)

R: Yes [00:09:37-8](http://localhost:2300/file=C:/Users/USER/Desktop/EXTRAS/godfrey/New%20folder/180905_1032_Amina.mp3time=577800)

I: What about the mammography investigation? [00:09:40-1](http://localhost:2300/file=C:/Users/USER/Desktop/EXTRAS/godfrey/New%20folder/180905_1032_Amina.mp3time=580100)

R: What is mammography? [00:09:41-5](http://localhost:2300/file=C:/Users/USER/Desktop/EXTRAS/godfrey/New%20folder/180905_1032_Amina.mp3time=581500)

I: Mammography, is another investigation resembling….it is also an imaging, was that done to you? [00:09:48-5](http://localhost:2300/file=C:/Users/USER/Desktop/EXTRAS/godfrey/New%20folder/180905_1032_Amina.mp3time=588500)

R: I didn’t do any other imaging investigation. What was done to me was that of ultrasound of the chest at -----, and I came to do an abdominal ultrasound here and chest x-ray at -----. [00:10:06-3](http://localhost:2300/file=C:/Users/USER/Desktop/EXTRAS/godfrey/New%20folder/180905_1032_Amina.mp3time=606300)

I: Okay. [00:10:06-0](http://localhost:2300/file=C:/Users/USER/Desktop/EXTRAS/godfrey/New%20folder/180905_1032_Amina.mp3time=606000)

R: Yes [00:10:06-7](http://localhost:2300/file=C:/Users/USER/Desktop/EXTRAS/godfrey/New%20folder/180905_1032_Amina.mp3time=606700)

I: And how long did it take ever since you started feeling sick and get those investigations? [00:10:12-2](http://localhost:2300/file=C:/Users/USER/Desktop/EXTRAS/godfrey/New%20folder/180905_1032_Amina.mp3time=612200)

R: I t can be approximately, since April until now, how many months can it be? [00:10:17-5](http://localhost:2300/file=C:/Users/USER/Desktop/EXTRAS/godfrey/New%20folder/180905_1032_Amina.mp3time=617500)

I: It is five months. [00:10:18-8](http://localhost:2300/file=C:/Users/USER/Desktop/EXTRAS/godfrey/New%20folder/180905_1032_Amina.mp3time=618800)

R: So, it is five months eeh. [00:10:22-3](http://localhost:2300/file=C:/Users/USER/Desktop/EXTRAS/godfrey/New%20folder/180905_1032_Amina.mp3time=622300)

I: Yes, five months I guess. [00:10:25-9](http://localhost:2300/file=C:/Users/USER/Desktop/EXTRAS/godfrey/New%20folder/180905_1032_Amina.mp3time=625900)

R: Mmmh [00:10:26-3](http://localhost:2300/file=C:/Users/USER/Desktop/EXTRAS/godfrey/New%20folder/180905_1032_Amina.mp3time=626300)

I: Do you know about the services like -----, female doctors who go treating people, have you ever come across them? [00:10:34-8](http://localhost:2300/file=C:/Users/USER/Desktop/EXTRAS/godfrey/New%20folder/180905_1032_Amina.mp3time=634800)

R: I have never come across them. [00:10:36-3](http://localhost:2300/file=C:/Users/USER/Desktop/EXTRAS/godfrey/New%20folder/180905_1032_Amina.mp3time=636300)

I: And what do you know about cancer? [00:10:42-8](http://localhost:2300/file=C:/Users/USER/Desktop/EXTRAS/godfrey/New%20folder/180905_1032_Amina.mp3time=642800)

R: About cancer? [00:10:49-4](http://localhost:2300/file=C:/Users/USER/Desktop/EXTRAS/godfrey/New%20folder/180905_1032_Amina.mp3time=649400)

I: Mmmh [00:10:49-9](http://localhost:2300/file=C:/Users/USER/Desktop/EXTRAS/godfrey/New%20folder/180905_1032_Amina.mp3time=649900)

R: Yes, for now I can say I know a bit because I already have cancer and I know cancer exists and it is a very scary disease in Tanzania or let me say the whole world. So, I have accepted that cancer exists and it has already affected me, what should I do? [00:11:11-3](http://localhost:2300/file=C:/Users/USER/Desktop/EXTRAS/godfrey/New%20folder/180905_1032_Amina.mp3time=671300)

I: In your family, is there any other person who has ever got a similar disease? [00:11:15-8](http://localhost:2300/file=C:/Users/USER/Desktop/EXTRAS/godfrey/New%20folder/180905_1032_Amina.mp3time=675800)

R: No, I disagree, I am the first. [00:11:17-3](http://localhost:2300/file=C:/Users/USER/Desktop/EXTRAS/godfrey/New%20folder/180905_1032_Amina.mp3time=677300)

I: You are the first? [00:11:17-9](http://localhost:2300/file=C:/Users/USER/Desktop/EXTRAS/godfrey/New%20folder/180905_1032_Amina.mp3time=677900)

R: Yes [00:11:18-6](http://localhost:2300/file=C:/Users/USER/Desktop/EXTRAS/godfrey/New%20folder/180905_1032_Amina.mp3time=678600)

I: Maybe is there any relative or friend who has ever got this disease? [00:11:20-7](http://localhost:2300/file=C:/Users/USER/Desktop/EXTRAS/godfrey/New%20folder/180905_1032_Amina.mp3time=680700)

R: I don’t have any relatives, or friends who have got this disease, I am the only one. [00:11:25-4](http://localhost:2300/file=C:/Users/USER/Desktop/EXTRAS/godfrey/New%20folder/180905_1032_Amina.mp3time=685400)

I: Have you ever heard even to a close friend? [00:11:29-4](http://localhost:2300/file=C:/Users/USER/Desktop/EXTRAS/godfrey/New%20folder/180905_1032_Amina.mp3time=689400)

R: To my close relatives, nobody has ever had the disease. [00:11:34-0](http://localhost:2300/file=C:/Users/USER/Desktop/EXTRAS/godfrey/New%20folder/180905_1032_Amina.mp3time=694000)

I: Because we meet so many people who come to the hospital because maybe she had somebody who was suffering from this illness and that pushed her to go to the hospital. [00:11:46-3](http://localhost:2300/file=C:/Users/USER/Desktop/EXTRAS/godfrey/New%20folder/180905_1032_Amina.mp3time=706300)

R: No, nobody pushed me. The ones who pushed me are my brothers. To be honest, after I showed them the swelling and for everyday that the swelling was growing and it was hardening, so my brothers advised me to go to the hospital, and they are the ones who challenged me, and I thank them for where we have reached. [00:12:19-3](http://localhost:2300/file=C:/Users/USER/Desktop/EXTRAS/godfrey/New%20folder/180905_1032_Amina.mp3time=739300)

I: So, did you think that it could be cancer since the beginning, or you didn’t know that? [00:12:28-4](http://localhost:2300/file=C:/Users/USER/Desktop/EXTRAS/godfrey/New%20folder/180905_1032_Amina.mp3time=748400)

R: I didn’t know anything. [00:12:30-4](http://localhost:2300/file=C:/Users/USER/Desktop/EXTRAS/godfrey/New%20folder/180905_1032_Amina.mp3time=750400)

I: And did you know maybe what the treatment was? [00:12:33-1](http://localhost:2300/file=C:/Users/USER/Desktop/EXTRAS/godfrey/New%20folder/180905_1032_Amina.mp3time=753100)

R: I knew it was an abscess and they will just resect it. I just knew it was an abscess and there are some people who were telling me that there are abscesses that are tissue like and they are always hard and they don’t ripen quickly. And it is true it took time until it busted to release the dirt.

[00:12:57-0](http://localhost:2300/file=C:/Users/USER/Desktop/EXTRAS/godfrey/New%20folder/180905_1032_Amina.mp3time=777000)

I: And what else apart from money contributed to the delay of treatment?

[00:13:07-1](http://localhost:2300/file=C:/Users/USER/Desktop/EXTRAS/godfrey/New%20folder/180905_1032_Amina.mp3time=787100)

R: Mmmh? [00:13:07-3](http://localhost:2300/file=C:/Users/USER/Desktop/EXTRAS/godfrey/New%20folder/180905_1032_Amina.mp3time=787300)

I: Do you think there is anything else apart from money, you told me that money is the only thing that contributes to the delay of getting treatment. A long time had passed. [00:13:17-9](http://localhost:2300/file=C:/Users/USER/Desktop/EXTRAS/godfrey/New%20folder/180905_1032_Amina.mp3time=797900)

R: What I can say is my own fear because until I reached here is that I was somehow pushed until I reached here. [00:13:28-0](http://localhost:2300/file=C:/Users/USER/Desktop/EXTRAS/godfrey/New%20folder/180905_1032_Amina.mp3time=808000)

I: What were you scared of? [00:13:39-9](http://localhost:2300/file=C:/Users/USER/Desktop/EXTRAS/godfrey/New%20folder/180905_1032_Amina.mp3time=819900)

R: Eh? [00:13:40-3](http://localhost:2300/file=C:/Users/USER/Desktop/EXTRAS/godfrey/New%20folder/180905_1032_Amina.mp3time=820300)

I: What were you scared of? [00:13:41-7](http://localhost:2300/file=C:/Users/USER/Desktop/EXTRAS/godfrey/New%20folder/180905_1032_Amina.mp3time=821700)

R: I was scared because I used to hear that those with this disease don’t get healed and I had so much pressure. I thought about my three children, when I come here, I will be admitted and sometimes I will stay for long because there are those who are admitted at ----- and they don’t recover. So, it was giving me a bit of a challenge, and I said, “if I go, I won’t return.” But I thank God my relatives have tried their best to encourage me until they brought me here where I am. I am just waiting for the doctor’s possibility as they have said.

[00:14:29-1](http://localhost:2300/file=C:/Users/USER/Desktop/EXTRAS/godfrey/New%20folder/180905_1032_Amina.mp3time=869100)

I: And were you scared after being told that... [00:14:33-5](http://localhost:2300/file=C:/Users/USER/Desktop/EXTRAS/godfrey/New%20folder/180905_1032_Amina.mp3time=873500)

R: I got scared after being told that the illness is cancer. I was scared and I couldn’t sleep, you can sleep this way and…

[00:14:48-4](http://localhost:2300/file=C:/Users/USER/Desktop/EXTRAS/godfrey/New%20folder/180905_1032_Amina.mp3time=888400)

I: And wake up. Didn’t you try asking from other people what they do? [00:14:53-1](http://localhost:2300/file=C:/Users/USER/Desktop/EXTRAS/godfrey/New%20folder/180905_1032_Amina.mp3time=893100)

R: I wasn’t asking people to be honest because I didn’t want people to know that I have the illness. In the environment that I live I didn’t want anyone to know that I have the problem. In the end of the day I think it is what made me delay to come to the hospital. First, I was really scared until I came to the hospital it had already busted and release pus. [00:15:20-0](http://localhost:2300/file=C:/Users/USER/Desktop/EXTRAS/godfrey/New%20folder/180905_1032_Amina.mp3time=920000)

I: Why didn’t you want people to know, don’t you think they might have helped you? [00:15:25-9](http://localhost:2300/file=C:/Users/USER/Desktop/EXTRAS/godfrey/New%20folder/180905_1032_Amina.mp3time=925900)

R: Do people in the street help each other? There is no helping rather than laughing at one another, that a certain lady has faced the trouble, that is what we street people have, not that somebody to encourage you, but they mostly come and say I have seen so and so, she has this terrible disease that cannot be cured. So, people tell you it is better to have AIDS you will be cured rather than this disease. There is when you get the fear. [00:15:58-5](http://localhost:2300/file=C:/Users/USER/Desktop/EXTRAS/godfrey/New%20folder/180905_1032_Amina.mp3time=958500)

I: So, you were even scared of coming to the hospital? [00:16:02-1](http://localhost:2300/file=C:/Users/USER/Desktop/EXTRAS/godfrey/New%20folder/180905_1032_Amina.mp3time=962100)

R: yes, you must be scared, but I thank God that my relatives pushed me until I came here, I am so grateful, most especially I thank my young brother. He helped me until I reached here, apart from my brothers, my young brother also played a big role, he is the one who helped me until I reached here where I am. [00:16:25-8](http://localhost:2300/file=C:/Users/USER/Desktop/EXTRAS/godfrey/New%20folder/180905_1032_Amina.mp3time=985800)

I: Who is your young brother, what work does he do? [00:16:27-7](http://localhost:2300/file=C:/Users/USER/Desktop/EXTRAS/godfrey/New%20folder/180905_1032_Amina.mp3time=987700)

R: Pardon? [00:16:28-4](http://localhost:2300/file=C:/Users/USER/Desktop/EXTRAS/godfrey/New%20folder/180905_1032_Amina.mp3time=988400)

I: Does he have any understanding on these issues? [00:16:31-7](http://localhost:2300/file=C:/Users/USER/Desktop/EXTRAS/godfrey/New%20folder/180905_1032_Amina.mp3time=991700)

R: He doesn’t have any understanding, he just pushed me to come to the hospital. [00:16:35-9](http://localhost:2300/file=C:/Users/USER/Desktop/EXTRAS/godfrey/New%20folder/180905_1032_Amina.mp3time=995900)

I: Is there no any relative who told you maybe there is alternative treatment from the people they came across? [00:16:42-0](http://localhost:2300/file=C:/Users/USER/Desktop/EXTRAS/godfrey/New%20folder/180905_1032_Amina.mp3time=1002000)

R: No. [00:16:43-0](http://localhost:2300/file=C:/Users/USER/Desktop/EXTRAS/godfrey/New%20folder/180905_1032_Amina.mp3time=1003000)

I: No? [00:16:43-9](http://localhost:2300/file=C:/Users/USER/Desktop/EXTRAS/godfrey/New%20folder/180905_1032_Amina.mp3time=1003900)

R: mmh [00:16:46-3](http://localhost:2300/file=C:/Users/USER/Desktop/EXTRAS/godfrey/New%20folder/180905_1032_Amina.mp3time=1006300)

I: What about your work, didn’t it contribute to the delay? That because you were scared to waste time and what about normal works? [00:16:58-0](http://localhost:2300/file=C:/Users/USER/Desktop/EXTRAS/godfrey/New%20folder/180905_1032_Amina.mp3time=1018000)

R: No. [00:16:58-9](http://localhost:2300/file=C:/Users/USER/Desktop/EXTRAS/godfrey/New%20folder/180905_1032_Amina.mp3time=1018900)

I: Didn’t you think maybe that this swelling will finish on its own? [00:17:04-5](http://localhost:2300/file=C:/Users/USER/Desktop/EXTRAS/godfrey/New%20folder/180905_1032_Amina.mp3time=1024500)

R: This has already fully developed; it is not something to finish on its own. It is a must that it should be attended by the medical treatment as the specialists claim. [00:17:16-3](http://localhost:2300/file=C:/Users/USER/Desktop/EXTRAS/godfrey/New%20folder/180905_1032_Amina.mp3time=1036300)

I: Okay, and on the part of hospital, concerning health service providers is there any challenge that made you delay to come? [00:17:28-1](http://localhost:2300/file=C:/Users/USER/Desktop/EXTRAS/godfrey/New%20folder/180905_1032_Amina.mp3time=1048100)

R: No. [00:17:28-9](http://localhost:2300/file=C:/Users/USER/Desktop/EXTRAS/godfrey/New%20folder/180905_1032_Amina.mp3time=1048900)

I: No? [00:17:30-1](http://localhost:2300/file=C:/Users/USER/Desktop/EXTRAS/godfrey/New%20folder/180905_1032_Amina.mp3time=1050100)

R: Yes. [00:17:30-5](http://localhost:2300/file=C:/Users/USER/Desktop/EXTRAS/godfrey/New%20folder/180905_1032_Amina.mp3time=1050500)

I: You just had .... [00:17:31-2](http://localhost:2300/file=C:/Users/USER/Desktop/EXTRAS/godfrey/New%20folder/180905_1032_Amina.mp3time=1051200)

R: I reached here and got the service. [00:17:33-7](http://localhost:2300/file=C:/Users/USER/Desktop/EXTRAS/godfrey/New%20folder/180905_1032_Amina.mp3time=1053700)

I: What about the first hospitals that you were attended? [00:17:37-1](http://localhost:2300/file=C:/Users/USER/Desktop/EXTRAS/godfrey/New%20folder/180905_1032_Amina.mp3time=1057100)

R: The first hospital was ----- I went there, the doctor checked me, and as he suspected he had to discharge me to -----. When I went to -----, I told them my problem and then I went for the ultrasound and they also gave me a transfer to come to -----. And then we started the process until I reached here where I have reached, it is really something to thank God for. [00:18:24-2](http://localhost:2300/file=C:/Users/USER/Desktop/EXTRAS/godfrey/New%20folder/180905_1032_Amina.mp3time=1104200)

I: Do you pray? [00:18:26-6](http://localhost:2300/file=C:/Users/USER/Desktop/EXTRAS/godfrey/New%20folder/180905_1032_Amina.mp3time=1106600)

R: Yes, I ask God to help me every day. [00:18:30-3](http://localhost:2300/file=C:/Users/USER/Desktop/EXTRAS/godfrey/New%20folder/180905_1032_Amina.mp3time=1110300)

I: Okay sister. Do you have anything you would like to say and it may not be not be to you but to other people, something that makes other people delay to seek medical treatment. You have told me many things; do you have any others to add on? [00:18:54-8](http://localhost:2300/file=C:/Users/USER/Desktop/EXTRAS/godfrey/New%20folder/180905_1032_Amina.mp3time=1134800)

R: Yes, I would like to ask women who feel any difference in their bodies not to stay quiet, they should go to the hospital to get early treatment, mmh only that. [00:19:06-0](http://localhost:2300/file=C:/Users/USER/Desktop/EXTRAS/godfrey/New%20folder/180905_1032_Amina.mp3time=1146000)

I: Okay, thank you -----. [00:19:08-4](http://localhost:2300/file=C:/Users/USER/Desktop/EXTRAS/godfrey/New%20folder/180905_1032_Amina.mp3time=1148400)

R: Okay. [00:19:08-8](http://localhost:2300/file=C:/Users/USER/Desktop/EXTRAS/godfrey/New%20folder/180905_1032_Amina.mp3time=1148800)

**CASE 12**

I: What is your name [00:00:00-0](http://localhost:2300/file=C:/Users/admin/Desktop/NSAJIGWA/180826_1142_Julita.mp3time=0)

R: My name is ----- [00:00:01-4](http://localhost:2300/file=C:/Users/admin/Desktop/NSAJIGWA/180826_1142_Julita.mp3time=1400)

I: -----? [00:00:02-5](http://localhost:2300/file=C:/Users/admin/Desktop/NSAJIGWA/180826_1142_Julita.mp3time=2500)

R: Yes, ----- [00:00:03-2](http://localhost:2300/file=C:/Users/admin/Desktop/NSAJIGWA/180826_1142_Julita.mp3time=3200)

I: Where do you come from [00:00:04-2](http://localhost:2300/file=C:/Users/admin/Desktop/NSAJIGWA/180826_1142_Julita.mp3time=4200)

R: I live here in -----, residing at ----- [00:00:07-3](http://localhost:2300/file=C:/Users/admin/Desktop/NSAJIGWA/180826_1142_Julita.mp3time=7300)

I: Okay [00:00:11-9](http://localhost:2300/file=C:/Users/admin/Desktop/NSAJIGWA/180826_1142_Julita.mp3time=11900)

R: Mmh [00:00:12-5](http://localhost:2300/file=C:/Users/admin/Desktop/NSAJIGWA/180826_1142_Julita.mp3time=12500)

I: And when were you born [00:00:13-1](http://localhost:2300/file=C:/Users/admin/Desktop/NSAJIGWA/180826_1142_Julita.mp3time=13100)

R: ----- [00:00:14-3](http://localhost:2300/file=C:/Users/admin/Desktop/NSAJIGWA/180826_1142_Julita.mp3time=14300)

I: What about your marital status [00:00:20-4](http://localhost:2300/file=C:/Users/admin/Desktop/NSAJIGWA/180826_1142_Julita.mp3time=20400)

R: Marital status? , [00:00:23-1](http://localhost:2300/file=C:/Users/admin/Desktop/NSAJIGWA/180826_1142_Julita.mp3time=23100)

I: Mmh [00:00:23-6](http://localhost:2300/file=C:/Users/admin/Desktop/NSAJIGWA/180826_1142_Julita.mp3time=23600)

R: No, am not married [00:00:25-1](http://localhost:2300/file=C:/Users/admin/Desktop/NSAJIGWA/180826_1142_Julita.mp3time=25100)

I: You have never been married or? [00:00:26-4](http://localhost:2300/file=C:/Users/admin/Desktop/NSAJIGWA/180826_1142_Julita.mp3time=26400)

R: I only cohabited but I have never been married [00:00:28-7](http://localhost:2300/file=C:/Users/admin/Desktop/NSAJIGWA/180826_1142_Julita.mp3time=28700)

I: And, do you have children [00:00:33-7](http://localhost:2300/file=C:/Users/admin/Desktop/NSAJIGWA/180826_1142_Julita.mp3time=33700)

R: Yes I do have, I had two children but one is already deceased [00:00:35-3](http://localhost:2300/file=C:/Users/admin/Desktop/NSAJIGWA/180826_1142_Julita.mp3time=35300)

I: Oh [00:00:41-3](http://localhost:2300/file=C:/Users/admin/Desktop/NSAJIGWA/180826_1142_Julita.mp3time=41300)

R: Mmh [00:00:41-9](http://localhost:2300/file=C:/Users/admin/Desktop/NSAJIGWA/180826_1142_Julita.mp3time=41900)

I: My condolences [00:00:42-8](http://localhost:2300/file=C:/Users/admin/Desktop/NSAJIGWA/180826_1142_Julita.mp3time=42800)

R: Thank you [00:00:43-6](http://localhost:2300/file=C:/Users/admin/Desktop/NSAJIGWA/180826_1142_Julita.mp3time=43600)

I: What level of education have you attained [00:00:44-4](http://localhost:2300/file=C:/Users/admin/Desktop/NSAJIGWA/180826_1142_Julita.mp3time=44400)

R: Grade 12 [00:00:47-8](http://localhost:2300/file=C:/Users/admin/Desktop/NSAJIGWA/180826_1142_Julita.mp3time=47800)

I: Grade 12? [00:00:50-1](http://localhost:2300/file=C:/Users/admin/Desktop/NSAJIGWA/180826_1142_Julita.mp3time=50100)

R: Yes [00:00:50-8](http://localhost:2300/file=C:/Users/admin/Desktop/NSAJIGWA/180826_1142_Julita.mp3time=50800)

I: Your times of having many grades in school [00:00:51-5](http://localhost:2300/file=C:/Users/admin/Desktop/NSAJIGWA/180826_1142_Julita.mp3time=51500)

R: I passed grade 8 [00:00:55-8](http://localhost:2300/file=C:/Users/admin/Desktop/NSAJIGWA/180826_1142_Julita.mp3time=55800)

I: What do you engage yourself with [00:01:01-0](http://localhost:2300/file=C:/Users/admin/Desktop/NSAJIGWA/180826_1142_Julita.mp3time=61000)

R: I used to work at ----- but currently I am retired [00:01:05-8](http://localhost:2300/file=C:/Users/admin/Desktop/NSAJIGWA/180826_1142_Julita.mp3time=65800)

I: -----, you said that you live at -----, what is the distance from where you live to hospital [00:01:12-0](http://localhost:2300/file=C:/Users/admin/Desktop/NSAJIGWA/180826_1142_Julita.mp3time=72000)

R: It’s not far [00:01:17-8](http://localhost:2300/file=C:/Users/admin/Desktop/NSAJIGWA/180826_1142_Julita.mp3time=77800)

I: Is it a walking distance [00:01:21-5](http://localhost:2300/file=C:/Users/admin/Desktop/NSAJIGWA/180826_1142_Julita.mp3time=81500)

R: No it’s a travelling distance but it’s not far [00:01:23-3](http://localhost:2300/file=C:/Users/admin/Desktop/NSAJIGWA/180826_1142_Julita.mp3time=83300)

I: Mmh [00:01:25-8](http://localhost:2300/file=C:/Users/admin/Desktop/NSAJIGWA/180826_1142_Julita.mp3time=85800)

R: Mmh [00:01:26-3](http://localhost:2300/file=C:/Users/admin/Desktop/NSAJIGWA/180826_1142_Julita.mp3time=86300)

I: And if you board a car, how much do you pay [00:01:26-9](http://localhost:2300/file=C:/Users/admin/Desktop/NSAJIGWA/180826_1142_Julita.mp3time=86900)

R: Since I don’t use one vehicle to get to hospital I board then stop then board again to it accounts to six or eight thousand [00:01:31-7](http://localhost:2300/file=C:/Users/admin/Desktop/NSAJIGWA/180826_1142_Julita.mp3time=91700)

I: So, you normally use a car [00:01:50-1](http://localhost:2300/file=C:/Users/admin/Desktop/NSAJIGWA/180826_1142_Julita.mp3time=110100)

R: Most of times due to my condition I use Uber for transport [00:01:51-9](http://localhost:2300/file=C:/Users/admin/Desktop/NSAJIGWA/180826_1142_Julita.mp3time=111900)

I: How did you get information about this disease did you know it prior or when you fell sick that’s when you knew about breast cancer [00:02:00-4](http://localhost:2300/file=C:/Users/admin/Desktop/NSAJIGWA/180826_1142_Julita.mp3time=120400)

R: Mmh, I knew about breast cancer but I never noticed it if it was cancer because I only saw something hard, like a scar not a typical swelling as the way they say it, it wasn’t like a gland, a swelling [00:02:12-3](http://localhost:2300/file=C:/Users/admin/Desktop/NSAJIGWA/180826_1142_Julita.mp3time=132300)

I: Where did you get the information from about this disease, is it television, radio or mobile phone [00:02:35-8](http://localhost:2300/file=C:/Users/admin/Desktop/NSAJIGWA/180826_1142_Julita.mp3time=155800)

R: In the past I used to go to ----- to do screening for cervical cancer and I ever checked for breast cancer but they said that there was no problem so I had no worries [00:02:48-1](http://localhost:2300/file=C:/Users/admin/Desktop/NSAJIGWA/180826_1142_Julita.mp3time=168100)

I: And do you know how to do a self-breast examination [00:03:01-2](http://localhost:2300/file=C:/Users/admin/Desktop/NSAJIGWA/180826_1142_Julita.mp3time=181200)

R: Yes, I knew how to do it and that’s why I said I expected to see a typical swelling but it wasn’t so I thought of something else ,but one day I had to explain it to a doctor that I have something on my breast and it feels hard but didn’t understand what it was [00:03:04-8](http://localhost:2300/file=C:/Users/admin/Desktop/NSAJIGWA/180826_1142_Julita.mp3time=184800)

I: Mmh [00:03:26-8](http://localhost:2300/file=C:/Users/admin/Desktop/NSAJIGWA/180826_1142_Julita.mp3time=206800)

R: So, they took a biopsy and told me that I have features of breast cancer [00:03:27-7](http://localhost:2300/file=C:/Users/admin/Desktop/NSAJIGWA/180826_1142_Julita.mp3time=207700)

I: Can you explain to me how the illness started to when you arrived here at ----- [00:03:38-4](http://localhost:2300/file=C:/Users/admin/Desktop/NSAJIGWA/180826_1142_Julita.mp3time=218400)

R: After I was told that it was cancer I never hesitated because doctor told me that it can spread so fast [00:03:50-5](http://localhost:2300/file=C:/Users/admin/Desktop/NSAJIGWA/180826_1142_Julita.mp3time=230500)

I: Oh  [00:04:04-6](http://localhost:2300/file=C:/Users/admin/Desktop/NSAJIGWA/180826_1142_Julita.mp3time=244600)

R: So I agreed without hesitation to undergo mastectomy 24/02/2017 [00:04:05-2](http://localhost:2300/file=C:/Users/admin/Desktop/NSAJIGWA/180826_1142_Julita.mp3time=245200)

I: 2017 [00:04:16-2](http://localhost:2300/file=C:/Users/admin/Desktop/NSAJIGWA/180826_1142_Julita.mp3time=256200)

R: Yes [00:04:17-3](http://localhost:2300/file=C:/Users/admin/Desktop/NSAJIGWA/180826_1142_Julita.mp3time=257300)

I: And what time had passed since you noticed changes in your breast [00:04:18-0](http://localhost:2300/file=C:/Users/admin/Desktop/NSAJIGWA/180826_1142_Julita.mp3time=258000)

R: I think it was like one year [00:04:23-5](http://localhost:2300/file=C:/Users/admin/Desktop/NSAJIGWA/180826_1142_Julita.mp3time=263500)

I: Since you noticed? [00:04:28-6](http://localhost:2300/file=C:/Users/admin/Desktop/NSAJIGWA/180826_1142_Julita.mp3time=268600)

R: Yes [00:04:29-8](http://localhost:2300/file=C:/Users/admin/Desktop/NSAJIGWA/180826_1142_Julita.mp3time=269800)

I: Oh, [00:04:30-2](http://localhost:2300/file=C:/Users/admin/Desktop/NSAJIGWA/180826_1142_Julita.mp3time=270200)

R: So after mastectomy at ----- hospital they sent me to ----- for clinic and I started clinics in May [00:04:30-9](http://localhost:2300/file=C:/Users/admin/Desktop/NSAJIGWA/180826_1142_Julita.mp3time=270900)

I: Mmh [00:04:49-9](http://localhost:2300/file=C:/Users/admin/Desktop/NSAJIGWA/180826_1142_Julita.mp3time=289900)

R: So I started both radiotherapy and chemotherapy [00:04:50-7](http://localhost:2300/file=C:/Users/admin/Desktop/NSAJIGWA/180826_1142_Julita.mp3time=290700)

I: Okay [00:04:56-7](http://localhost:2300/file=C:/Users/admin/Desktop/NSAJIGWA/180826_1142_Julita.mp3time=296700)

R: I did 25 cycles of radiotherapy and 6 cycles of chemotherapy, I was well but lates I developed a blister here. [00:04:57-5](http://localhost:2300/file=C:/Users/admin/Desktop/NSAJIGWA/180826_1142_Julita.mp3time=297500)

I: Mmh [00:05:08-6](http://localhost:2300/file=C:/Users/admin/Desktop/NSAJIGWA/180826_1142_Julita.mp3time=308600)

R: So, later on I returned to clinic and explained it to doctor and he told me that we should do investigations to know what it is [00:05:09-1](http://localhost:2300/file=C:/Users/admin/Desktop/NSAJIGWA/180826_1142_Julita.mp3time=309100)

I: Mmh [00:05:19-9](http://localhost:2300/file=C:/Users/admin/Desktop/NSAJIGWA/180826_1142_Julita.mp3time=319900)

R: After investigating it they told me that the disease is still on , so I went by with chemotherapy 8 cycles [00:05:20-3](http://localhost:2300/file=C:/Users/admin/Desktop/NSAJIGWA/180826_1142_Julita.mp3time=320300)

I: Was it at ----- or ocean road [00:05:28-2](http://localhost:2300/file=C:/Users/admin/Desktop/NSAJIGWA/180826_1142_Julita.mp3time=328200)

R: ----- [00:05:29-3](http://localhost:2300/file=C:/Users/admin/Desktop/NSAJIGWA/180826_1142_Julita.mp3time=329300)

I: -----? [00:05:30-6](http://localhost:2300/file=C:/Users/admin/Desktop/NSAJIGWA/180826_1142_Julita.mp3time=330600)

R: Yes, so I began another round of chemotherapy,8 cycles [00:05:31-2](http://localhost:2300/file=C:/Users/admin/Desktop/NSAJIGWA/180826_1142_Julita.mp3time=331200)

I: Before we continue, I would like to know more on things that caused a delay for you to come to hospital. You said once you noticed changes on your breast it took you one year that’s when you went to hospital , in between what where you upto [00:05:38-7](http://localhost:2300/file=C:/Users/admin/Desktop/NSAJIGWA/180826_1142_Julita.mp3time=338700)

R: I wasn’t worried about anything since I never felt pain [00:05:57-8](http://localhost:2300/file=C:/Users/admin/Desktop/NSAJIGWA/180826_1142_Julita.mp3time=357800)

I: Mmh [00:06:02-5](http://localhost:2300/file=C:/Users/admin/Desktop/NSAJIGWA/180826_1142_Julita.mp3time=362500)

R: So I wasn’t worried thinking that it’s a normal thing [00:06:03-3](http://localhost:2300/file=C:/Users/admin/Desktop/NSAJIGWA/180826_1142_Julita.mp3time=363300)

I: So you never did any investigations in that particular year [00:06:10-3](http://localhost:2300/file=C:/Users/admin/Desktop/NSAJIGWA/180826_1142_Julita.mp3time=370300)

R: No I didn’t [00:06:14-3](http://localhost:2300/file=C:/Users/admin/Desktop/NSAJIGWA/180826_1142_Julita.mp3time=374300)

I: And was the condition not progressing for all those months [00:06:15-6](http://localhost:2300/file=C:/Users/admin/Desktop/NSAJIGWA/180826_1142_Julita.mp3time=375600)

R: Not at all, I never felt any illness. [00:06:18-2](http://localhost:2300/file=C:/Users/admin/Desktop/NSAJIGWA/180826_1142_Julita.mp3time=378200)

I: So, by your opinion do you think you came early or late to hospital for treatment [00:06:24-6](http://localhost:2300/file=C:/Users/admin/Desktop/NSAJIGWA/180826_1142_Julita.mp3time=384600)

R: I can’t really say since they didn’t tell me the stage of the disease [00:06:33-8](http://localhost:2300/file=C:/Users/admin/Desktop/NSAJIGWA/180826_1142_Julita.mp3time=393800)

I: Didn’t you know that it is cancer? [00:06:42-1](http://localhost:2300/file=C:/Users/admin/Desktop/NSAJIGWA/180826_1142_Julita.mp3time=402100)

R: Yes, so it’s possible that I was late that’s why it recurred, I don’t really know . But after completing the 8 cycles chemotherapy three for each cycle then that was the end, When I began the treatment the wound laid open so since ----- I was taking care of the wound [00:06:45-1](http://localhost:2300/file=C:/Users/admin/Desktop/NSAJIGWA/180826_1142_Julita.mp3time=405100)

I: Was it at home or hospital [00:07:25-8](http://localhost:2300/file=C:/Users/admin/Desktop/NSAJIGWA/180826_1142_Julita.mp3time=445800)

R: I went to hospital and received medications [00:07:27-8](http://localhost:2300/file=C:/Users/admin/Desktop/NSAJIGWA/180826_1142_Julita.mp3time=447800)

I: Mmh [00:07:30-0](http://localhost:2300/file=C:/Users/admin/Desktop/NSAJIGWA/180826_1142_Julita.mp3time=450000)

R: Mmh [00:07:30-3](http://localhost:2300/file=C:/Users/admin/Desktop/NSAJIGWA/180826_1142_Julita.mp3time=450300)

I: Are there no other medications that you tried to use apart from the ones from the hospital [00:07:31-2](http://localhost:2300/file=C:/Users/admin/Desktop/NSAJIGWA/180826_1142_Julita.mp3time=451200)

R: No [00:07:35-7](http://localhost:2300/file=C:/Users/admin/Desktop/NSAJIGWA/180826_1142_Julita.mp3time=455700)

I: You know there a lot of talks going on out there about different medications [00:07:36-2](http://localhost:2300/file=C:/Users/admin/Desktop/NSAJIGWA/180826_1142_Julita.mp3time=456200)

R: No, so I only used medications from hospital though I saw the wound worsening [00:07:41-0](http://localhost:2300/file=C:/Users/admin/Desktop/NSAJIGWA/180826_1142_Julita.mp3time=461000)

I: Mmh [00:07:54-5](http://localhost:2300/file=C:/Users/admin/Desktop/NSAJIGWA/180826_1142_Julita.mp3time=474500)

R: I was told to use flagyl the powdery one but no response. So and the wound was whitish in colour and they told me that they can’t do anything about it or even scraping it because all cells around that region were already dead. [00:07:55-3](http://localhost:2300/file=C:/Users/admin/Desktop/NSAJIGWA/180826_1142_Julita.mp3time=475300)

I: Mmh [00:08:26-1](http://localhost:2300/file=C:/Users/admin/Desktop/NSAJIGWA/180826_1142_Julita.mp3time=506100)

R: So, since there are no surgeons at ----- so I tried to reach out to ----- so doctor took a biopsy and did a histology, they didn’t scrap it and was adviced to use honey [00:08:26-9](http://localhost:2300/file=C:/Users/admin/Desktop/NSAJIGWA/180826_1142_Julita.mp3time=506900)

I: Mmh: [00:08:50-9](http://localhost:2300/file=C:/Users/admin/Desktop/NSAJIGWA/180826_1142_Julita.mp3time=530900)

R: I kept on using honey till the wound progressed well [00:08:52-1](http://localhost:2300/file=C:/Users/admin/Desktop/NSAJIGWA/180826_1142_Julita.mp3time=532100)

R: I kept on doing dressing and since it was a big hole so started progressing well [00:08:56-2](http://localhost:2300/file=C:/Users/admin/Desktop/NSAJIGWA/180826_1142_Julita.mp3time=536200)

I: I would like to know before you started treatment , did you ever do any investigation like mammography or ultrasound [00:09:09-4](http://localhost:2300/file=C:/Users/admin/Desktop/NSAJIGWA/180826_1142_Julita.mp3time=549400)

R: No, [00:09:21-7](http://localhost:2300/file=C:/Users/admin/Desktop/NSAJIGWA/180826_1142_Julita.mp3time=561700)

I: You never did investigations [00:09:23-0](http://localhost:2300/file=C:/Users/admin/Desktop/NSAJIGWA/180826_1142_Julita.mp3time=563000)

R: Yes [00:09:23-9](http://localhost:2300/file=C:/Users/admin/Desktop/NSAJIGWA/180826_1142_Julita.mp3time=563900)

I: To when you started being treated here [00:09:24-4](http://localhost:2300/file=C:/Users/admin/Desktop/NSAJIGWA/180826_1142_Julita.mp3time=564400)

R: Mmh, After [00:09:27-3](http://localhost:2300/file=C:/Users/admin/Desktop/NSAJIGWA/180826_1142_Julita.mp3time=567300)

I: After what? [00:09:29-7](http://localhost:2300/file=C:/Users/admin/Desktop/NSAJIGWA/180826_1142_Julita.mp3time=569700)

R: After being diagnosed at ----- I started doing investigations [00:09:30-6](http://localhost:2300/file=C:/Users/admin/Desktop/NSAJIGWA/180826_1142_Julita.mp3time=570600)

I: Was chest Xray one of the investigations? [00:09:37-3](http://localhost:2300/file=C:/Users/admin/Desktop/NSAJIGWA/180826_1142_Julita.mp3time=577300)

R: Yes, I did ultrasound, ECHO, ECG [00:09:40-1](http://localhost:2300/file=C:/Users/admin/Desktop/NSAJIGWA/180826_1142_Julita.mp3time=580100)

I: Oh [00:09:48-5](http://localhost:2300/file=C:/Users/admin/Desktop/NSAJIGWA/180826_1142_Julita.mp3time=588500)

R: Yes [00:09:50-6](http://localhost:2300/file=C:/Users/admin/Desktop/NSAJIGWA/180826_1142_Julita.mp3time=590600)

I: What time had already passed by then [00:09:51-4](http://localhost:2300/file=C:/Users/admin/Desktop/NSAJIGWA/180826_1142_Julita.mp3time=591400)

R: Not much, I ddint take time since doctor adviced me to to make haste for operation so within one month I was operated on [00:09:56-9](http://localhost:2300/file=C:/Users/admin/Desktop/NSAJIGWA/180826_1142_Julita.mp3time=596900)

I: Aah, okay [00:10:10-5](http://localhost:2300/file=C:/Users/admin/Desktop/NSAJIGWA/180826_1142_Julita.mp3time=610500)

R: Yes [00:10:11-1](http://localhost:2300/file=C:/Users/admin/Desktop/NSAJIGWA/180826_1142_Julita.mp3time=611100)

I: Do you live with your relatives [00:10:11-9](http://localhost:2300/file=C:/Users/admin/Desktop/NSAJIGWA/180826_1142_Julita.mp3time=611900)

R: Yes, I do live with my relatives [00:10:15-7](http://localhost:2300/file=C:/Users/admin/Desktop/NSAJIGWA/180826_1142_Julita.mp3time=615700)

I: How have they contributed to encourage reach the hospital for treatment [00:10:18-4](http://localhost:2300/file=C:/Users/admin/Desktop/NSAJIGWA/180826_1142_Julita.mp3time=618400)

R: They really do help me [00:10:22-5](http://localhost:2300/file=C:/Users/admin/Desktop/NSAJIGWA/180826_1142_Julita.mp3time=622500)

I: Mmh [00:10:24-1](http://localhost:2300/file=C:/Users/admin/Desktop/NSAJIGWA/180826_1142_Julita.mp3time=624100)

R: Yes [00:10:24-8](http://localhost:2300/file=C:/Users/admin/Desktop/NSAJIGWA/180826_1142_Julita.mp3time=624800)

I: Do you use health insurance scheme [00:10:24-6](http://localhost:2300/file=C:/Users/admin/Desktop/NSAJIGWA/180826_1142_Julita.mp3time=624600)

R: Yes I do [00:10:27-3](http://localhost:2300/file=C:/Users/admin/Desktop/NSAJIGWA/180826_1142_Julita.mp3time=627300)

I: Did you have it from the start or after being sick [00:10:30-1](http://localhost:2300/file=C:/Users/admin/Desktop/NSAJIGWA/180826_1142_Julita.mp3time=630100)

R: No, my child did it for me [00:10:32-6](http://localhost:2300/file=C:/Users/admin/Desktop/NSAJIGWA/180826_1142_Julita.mp3time=632600)

I: After falling sick [00:10:35-2](http://localhost:2300/file=C:/Users/admin/Desktop/NSAJIGWA/180826_1142_Julita.mp3time=635200)

R: No, it was since before [00:10:37-8](http://localhost:2300/file=C:/Users/admin/Desktop/NSAJIGWA/180826_1142_Julita.mp3time=637800)

I: Before falling sick [00:10:39-1](http://localhost:2300/file=C:/Users/admin/Desktop/NSAJIGWA/180826_1142_Julita.mp3time=639100)

R: Yes, right after I got retired [00:10:40-1](http://localhost:2300/file=C:/Users/admin/Desktop/NSAJIGWA/180826_1142_Julita.mp3time=640100)

I: Mmh [00:10:46-0](http://localhost:2300/file=C:/Users/admin/Desktop/NSAJIGWA/180826_1142_Julita.mp3time=646000)

R: Yes [00:10:46-0](http://localhost:2300/file=C:/Users/admin/Desktop/NSAJIGWA/180826_1142_Julita.mp3time=646000)

I: So, while you were working you had one [00:10:47-1](http://localhost:2300/file=C:/Users/admin/Desktop/NSAJIGWA/180826_1142_Julita.mp3time=647100)

R: We weren’t using national health insurance fund, we were using from private sectors [00:10:49-7](http://localhost:2300/file=C:/Users/admin/Desktop/NSAJIGWA/180826_1142_Julita.mp3time=649700)

I: Private sectors? [00:10:53-7](http://localhost:2300/file=C:/Users/admin/Desktop/NSAJIGWA/180826_1142_Julita.mp3time=653700)

R: Yes [00:10:53-7](http://localhost:2300/file=C:/Users/admin/Desktop/NSAJIGWA/180826_1142_Julita.mp3time=653700)

I: Weren’t you scared maybe [00:10:55-3](http://localhost:2300/file=C:/Users/admin/Desktop/NSAJIGWA/180826_1142_Julita.mp3time=655300)

R: No [00:10:59-3](http://localhost:2300/file=C:/Users/admin/Desktop/NSAJIGWA/180826_1142_Julita.mp3time=659300)

I: You never had fear? [00:11:01-4](http://localhost:2300/file=C:/Users/admin/Desktop/NSAJIGWA/180826_1142_Julita.mp3time=661400)

R: No, I never had fear [00:11:01-8](http://localhost:2300/file=C:/Users/admin/Desktop/NSAJIGWA/180826_1142_Julita.mp3time=661800)

I: Okay [00:11:02-5](http://localhost:2300/file=C:/Users/admin/Desktop/NSAJIGWA/180826_1142_Julita.mp3time=662500)

R: Yes [00:11:02-5](http://localhost:2300/file=C:/Users/admin/Desktop/NSAJIGWA/180826_1142_Julita.mp3time=662500)

I: Apart from hospital services what else are you doing to help out with your treatment [00:11:03-7](http://localhost:2300/file=C:/Users/admin/Desktop/NSAJIGWA/180826_1142_Julita.mp3time=663700)

R: No, honestly I only rely on hospital and God [00:11:15-3](http://localhost:2300/file=C:/Users/admin/Desktop/NSAJIGWA/180826_1142_Julita.mp3time=675300)

I: Mmh [00:11:20-4](http://localhost:2300/file=C:/Users/admin/Desktop/NSAJIGWA/180826_1142_Julita.mp3time=680400)

R: Yes [00:11:20-4](http://localhost:2300/file=C:/Users/admin/Desktop/NSAJIGWA/180826_1142_Julita.mp3time=680400)

I: Do you also do prayers [00:11:20-9](http://localhost:2300/file=C:/Users/admin/Desktop/NSAJIGWA/180826_1142_Julita.mp3time=680900)

R: Yes, I do prayers [00:11:23-7](http://localhost:2300/file=C:/Users/admin/Desktop/NSAJIGWA/180826_1142_Julita.mp3time=683700)

I: The time you saw the hard scar on the breast what did you think was the reasons [00:11:25-8](http://localhost:2300/file=C:/Users/admin/Desktop/NSAJIGWA/180826_1142_Julita.mp3time=685800)

R: It wasn’t a scar as such [00:11:31-9](http://localhost:2300/file=C:/Users/admin/Desktop/NSAJIGWA/180826_1142_Julita.mp3time=691900)

I: Mmh [00:11:33-2](http://localhost:2300/file=C:/Users/admin/Desktop/NSAJIGWA/180826_1142_Julita.mp3time=693200)

R: It had a hard feel [00:11:33-7](http://localhost:2300/file=C:/Users/admin/Desktop/NSAJIGWA/180826_1142_Julita.mp3time=693700)

I: Why weren’t scared but instead you just stayed and watched [00:11:37-0](http://localhost:2300/file=C:/Users/admin/Desktop/NSAJIGWA/180826_1142_Julita.mp3time=697000)

R: (laughing), I really don’t know why I was never worried [00:11:41-5](http://localhost:2300/file=C:/Users/admin/Desktop/NSAJIGWA/180826_1142_Julita.mp3time=701500)

I: Mmh [00:11:48-2](http://localhost:2300/file=C:/Users/admin/Desktop/NSAJIGWA/180826_1142_Julita.mp3time=708200)

R: Yes [00:11:48-5](http://localhost:2300/file=C:/Users/admin/Desktop/NSAJIGWA/180826_1142_Julita.mp3time=708500)

I: You never told anyone about it? [00:11:49-1](http://localhost:2300/file=C:/Users/admin/Desktop/NSAJIGWA/180826_1142_Julita.mp3time=709100)

R: Yes, I didn’t but after being diagnosed I told my relatives that I have cancer so they never hesitated [00:11:51-6](http://localhost:2300/file=C:/Users/admin/Desktop/NSAJIGWA/180826_1142_Julita.mp3time=711600)

I: Mmh [00:12:01-8](http://localhost:2300/file=C:/Users/admin/Desktop/NSAJIGWA/180826_1142_Julita.mp3time=721800)

R: They only advised me and there was no other way [00:12:02-4](http://localhost:2300/file=C:/Users/admin/Desktop/NSAJIGWA/180826_1142_Julita.mp3time=722400)

I: Apart from removing it? [00:12:07-2](http://localhost:2300/file=C:/Users/admin/Desktop/NSAJIGWA/180826_1142_Julita.mp3time=727200)

R: Yes, apart from removing it as advised by the doctor [00:12:08-3](http://localhost:2300/file=C:/Users/admin/Desktop/NSAJIGWA/180826_1142_Julita.mp3time=728300)

I: Weren’t you scared after being told [00:12:11-7](http://localhost:2300/file=C:/Users/admin/Desktop/NSAJIGWA/180826_1142_Julita.mp3time=731700)

R: No, I wasn’t [00:12:13-6](http://localhost:2300/file=C:/Users/admin/Desktop/NSAJIGWA/180826_1142_Julita.mp3time=733600)

I: Mmh [00:12:14-1](http://localhost:2300/file=C:/Users/admin/Desktop/NSAJIGWA/180826_1142_Julita.mp3time=734100)

R: I wasn’t scared [00:12:14-7](http://localhost:2300/file=C:/Users/admin/Desktop/NSAJIGWA/180826_1142_Julita.mp3time=734700)

I: Okay [00:12:16-1](http://localhost:2300/file=C:/Users/admin/Desktop/NSAJIGWA/180826_1142_Julita.mp3time=736100)

R: Okay [00:12:18-0](http://localhost:2300/file=C:/Users/admin/Desktop/NSAJIGWA/180826_1142_Julita.mp3time=738000)

I: So you are the one who noticed it first [00:12:30-5](http://localhost:2300/file=C:/Users/admin/Desktop/NSAJIGWA/180826_1142_Julita.mp3time=750500)

R: Yes [00:12:32-6](http://localhost:2300/file=C:/Users/admin/Desktop/NSAJIGWA/180826_1142_Julita.mp3time=752600)

I: And any challenges that you faced during your referral to here? [00:12:38-3](http://localhost:2300/file=C:/Users/admin/Desktop/NSAJIGWA/180826_1142_Julita.mp3time=758300)

R: No, I never faced challenges with the fact that [00:12:47-9](http://localhost:2300/file=C:/Users/admin/Desktop/NSAJIGWA/180826_1142_Julita.mp3time=767900)

I: Getting referral ? [00:12:53-0](http://localhost:2300/file=C:/Users/admin/Desktop/NSAJIGWA/180826_1142_Julita.mp3time=773000)

R: Yes, I never faced challenges [00:12:53-8](http://localhost:2300/file=C:/Users/admin/Desktop/NSAJIGWA/180826_1142_Julita.mp3time=773800)

I: Do you have relatives who have ever suffered from the similar disease [00:13:02-5](http://localhost:2300/file=C:/Users/admin/Desktop/NSAJIGWA/180826_1142_Julita.mp3time=782500)

R: No, I have never heard [00:13:08-4](http://localhost:2300/file=C:/Users/admin/Desktop/NSAJIGWA/180826_1142_Julita.mp3time=788400)

I: Or maybe a friend [00:13:10-2](http://localhost:2300/file=C:/Users/admin/Desktop/NSAJIGWA/180826_1142_Julita.mp3time=790200)

R: I have never met with a friend or a neighbor or a relative with similar disease [00:13:16-3](http://localhost:2300/file=C:/Users/admin/Desktop/NSAJIGWA/180826_1142_Julita.mp3time=796300)

I: So, you have no experience of anyone who has ever suffered from similar problem [00:13:29-2](http://localhost:2300/file=C:/Users/admin/Desktop/NSAJIGWA/180826_1142_Julita.mp3time=809200)

R: No [00:13:36-0](http://localhost:2300/file=C:/Users/admin/Desktop/NSAJIGWA/180826_1142_Julita.mp3time=816000)

I: Has there ever been none? [00:13:36-8](http://localhost:2300/file=C:/Users/admin/Desktop/NSAJIGWA/180826_1142_Julita.mp3time=816800)

R: Yes [00:13:37-4](http://localhost:2300/file=C:/Users/admin/Desktop/NSAJIGWA/180826_1142_Julita.mp3time=817400)

I: Okay, because I wanted to know by your own opinion as to why people do delay reaching to hospital [00:13:38-4](http://localhost:2300/file=C:/Users/admin/Desktop/NSAJIGWA/180826_1142_Julita.mp3time=818400)

R: Honestly, reaching to hospital maybe there should be provision of education [00:13:55-8](http://localhost:2300/file=C:/Users/admin/Desktop/NSAJIGWA/180826_1142_Julita.mp3time=835800)

I: Mmh [00:14:01-2](http://localhost:2300/file=C:/Users/admin/Desktop/NSAJIGWA/180826_1142_Julita.mp3time=841200)

R: You know we Africans rarely do checkups [00:14:01-7](http://localhost:2300/file=C:/Users/admin/Desktop/NSAJIGWA/180826_1142_Julita.mp3time=841700)

I: Mmh [00:14:09-9](http://localhost:2300/file=C:/Users/admin/Desktop/NSAJIGWA/180826_1142_Julita.mp3time=849900)

R: So, you are sick but you keep on working regarding it as a normal condition till when you realize that its an advanced problem that’s when you rush to hospital [00:14:10-5](http://localhost:2300/file=C:/Users/admin/Desktop/NSAJIGWA/180826_1142_Julita.mp3time=850500)

I: Mmh [00:14:24-1](http://localhost:2300/file=C:/Users/admin/Desktop/NSAJIGWA/180826_1142_Julita.mp3time=864100)

R: Yes [00:14:24-6](http://localhost:2300/file=C:/Users/admin/Desktop/NSAJIGWA/180826_1142_Julita.mp3time=864600)

I: Okay, thank you so much [00:14:25-7](http://localhost:2300/file=C:/Users/admin/Desktop/NSAJIGWA/180826_1142_Julita.mp3time=865700)

R: Okay, you are welcome [00:14:31-5](http://localhost:2300/file=C:/Users/admin/Desktop/NSAJIGWA/180826_1142_Julita.mp3time=871500)

I: Maybe if there is anything else from you? [00:14:32-7](http://localhost:2300/file=C:/Users/admin/Desktop/NSAJIGWA/180826_1142_Julita.mp3time=872700)

R: No, I only have a request that you professionals should keep on educating us, so that many people become educated [00:14:36-5](http://localhost:2300/file=C:/Users/admin/Desktop/NSAJIGWA/180826_1142_Julita.mp3time=876500)

I: Mmh [00:14:45-5](http://localhost:2300/file=C:/Users/admin/Desktop/NSAJIGWA/180826_1142_Julita.mp3time=885500)

R: But for now many people tend to be understanding but in rural areas education on this hasn’t reached yet [00:14:45-8](http://localhost:2300/file=C:/Users/admin/Desktop/NSAJIGWA/180826_1142_Julita.mp3time=885800)

I: Mmh [00:14:53-5](http://localhost:2300/file=C:/Users/admin/Desktop/NSAJIGWA/180826_1142_Julita.mp3time=893500)

R: And, that people are very hesitant when it comes to going to hospital but instead do seek for other means first [00:14:53-9](http://localhost:2300/file=C:/Users/admin/Desktop/NSAJIGWA/180826_1142_Julita.mp3time=893900)

I: Mmh [00:15:03-4](http://localhost:2300/file=C:/Users/admin/Desktop/NSAJIGWA/180826_1142_Julita.mp3time=903400)

R: With lies that once you are on radiotherapy then you won’t be healed [00:15:04-3](http://localhost:2300/file=C:/Users/admin/Desktop/NSAJIGWA/180826_1142_Julita.mp3time=904300)

I: Mmh [00:15:13-3](http://localhost:2300/file=C:/Users/admin/Desktop/NSAJIGWA/180826_1142_Julita.mp3time=913300)

R: But I thank God that they investigated me and found that am cancer free 00:15:13-9

I: Okay [00:15:20-1](http://localhost:2300/file=C:/Users/admin/Desktop/NSAJIGWA/180826_1142_Julita.mp3time=920100)

R: Yes [00:15:20-3](http://localhost:2300/file=C:/Users/admin/Desktop/NSAJIGWA/180826_1142_Julita.mp3time=920300)

I:You said that the issue is education but we thought that’s for people outside the city and you are here in ----- and maybe you delayed getting education so what do you think is the problem [00:15:21-8](http://localhost:2300/file=C:/Users/admin/Desktop/NSAJIGWA/180826_1142_Julita.mp3time=921800)

R:I did not delay getting education on breast cancer ,maybe its just carelessness of not making follow-ups on health issues because when they started doing screening on breast cancer [00:15:41-3](http://localhost:2300/file=C:/Users/admin/Desktop/NSAJIGWA/180826_1142_Julita.mp3time=941300)

I: Mmh [00:15:58-3](http://localhost:2300/file=C:/Users/admin/Desktop/NSAJIGWA/180826_1142_Julita.mp3time=958300) [00:15:57-2](http://localhost:2300/file=C:/Users/admin/Desktop/NSAJIGWA/180826_1142_Julita.mp3time=957200)

R: I started clinic at -----, it was in year ----- [00:15:57-8](http://localhost:2300/file=C:/Users/admin/Desktop/NSAJIGWA/180826_1142_Julita.mp3time=957800)

I: Where you being treated at -----? [00:16:04-0](http://localhost:2300/file=C:/Users/admin/Desktop/NSAJIGWA/180826_1142_Julita.mp3time=964000)

R: Yes. We were being treated and done screening on cervical cancer, I did not do screening on breast cancer [00:16:05-8](http://localhost:2300/file=C:/Users/admin/Desktop/NSAJIGWA/180826_1142_Julita.mp3time=965800)

I: Why did you first think about cervical cancer [00:16:20-9](http://localhost:2300/file=C:/Users/admin/Desktop/NSAJIGWA/180826_1142_Julita.mp3time=980900)

R: They used to say that they are checking and whenever you check you find that nothing is wrong, while there is a problem since I thought that I would find a typical swelling but this one was more of a scar [00:16:24-9](http://localhost:2300/file=C:/Users/admin/Desktop/NSAJIGWA/180826_1142_Julita.mp3time=984900)

I: Oh [00:16:46-3](http://localhost:2300/file=C:/Users/admin/Desktop/NSAJIGWA/180826_1142_Julita.mp3time=1006300)

R: Yes, so I thought I didn’t have a problem [00:16:48-1](http://localhost:2300/file=C:/Users/admin/Desktop/NSAJIGWA/180826_1142_Julita.mp3time=1008100)

I: Okay -----, thank you [00:16:54-9](http://localhost:2300/file=C:/Users/admin/Desktop/NSAJIGWA/180826_1142_Julita.mp3time=1014900)

R: Okay, you are welcome [00:16:57-2](http://localhost:2300/file=C:/Users/admin/Desktop/NSAJIGWA/180826_1142_Julita.mp3time=1017200)
